# Engineering the Photophysics of Cyanines by Chain C1′ Substituents

**DOI:** 10.1021/acs.joc.5c02283

**Published:** 2025-12-08

**Authors:** Ottavio Bedocchi, Jan Polena, Jana Okoročenkova, Petr Slavíček, Petr Klán

**Affiliations:** † Department of Chemistry, Faculty of Science, 37748 Masaryk University, 62500 Brno, Czech Republic; ‡ RECETOX, Faculty of Science, Masaryk University, 62500 Brno, Czech Republic; § Department of Physical Chemistry, University of Chemistry and Technology, Prague, 16628 Prague 6, Czech Republic

## Abstract

Cyanine dyes are widely used in bioimaging, sensing, optoelectronic, and medicinal applications due to their tunable photophysical properties. However, controlling their electronic structures and photophysical properties remains a challenge. Here we report a general synthetic route to pentamethine and heptamethine cyanines bearing C1′ chain substituents that allow substantial control of their electronic, photophysical, and photochemical properties. By varying the terminal heterocycle and introducing various substituents at the 1′-position, we investigated the role of symmetry breaking and its impact on bond length alternation (BLA) and out-of-plane rotation (OPR). Our analysis shows that OPR, coupled with BLA, suppresses or hypsochromically shifts the first absorption band, thereby significantly altering the absorption properties of the studied dyes. This effect is particularly pronounced in structures with different heterocyclic end groups and bulky or electron deficient substituents at the 1′-position. Through quantum chemical calculations and spectroscopic analyses, we demonstrate how these modifications can be used to tune optical properties of these dyes across the visible region, paving the way for their further customization.

## Graphical abstract

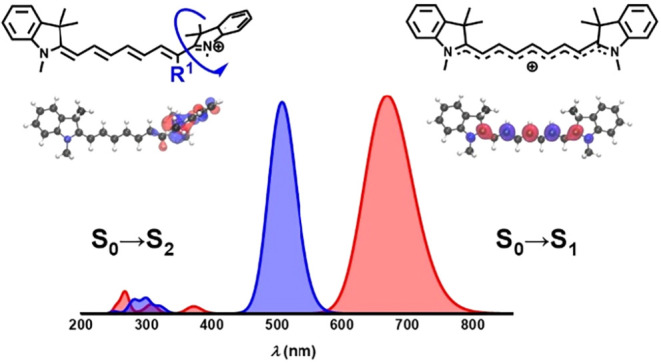

## Introduction

Cyanines are a diverse group of organic dyes characterized by the presence of a polymethine chain connecting two heteroatoms.^1^ They are classified based on the number of methine units in the chain, such as trimethine (Cy3), pentamethine (Cy5), and heptamethine (Cy7) cyanines. This distinctive conjugated system underlies their unique photophysical properties and chemical reactivity.^2^ Notably, cyanines have demonstrated significant applications in biology and medicine.^3,4^ For example, Cy7 derivatives have been employed for in vivo fluorescent imaging.^5,6^ It is possible to tune their properties by functionalizing the polyene chain of the dye. Chain-substituted cyanines have been developed for use in fluorescence microscopy,^7^ ROS (reactive oxygen species)^8^ or pH sensing,^9^ and as photoactivatable compounds.^10–12^

The photophysical properties of cyanine dyes, such as absorption and emission energies, fluorescence quantum yields, and solvent-dependent photophysics, are controlled by their molecular structure.^13–20^ The most straightforward strategy for modulating these properties is to change the number of methine units. However, more subtle design principles focus on structural modifications of the chain, often achieved through functionalization.^13,15,16,21–23^

Cyanine dyes are considered prototypical examples of nearly fully conjugated double-bond systems, where their electronic structure closely follows the particle-in-a-box model.^24^ The electronic properties and transitions can be fine-tuned through Peierls distortion associated with bond length alternation (BLA, Scheme 1).^25,26^ This distortion was demonstrated to be caused by increasing the chain length,^25,27^ functionalization,^23^ polarization by counterions,^28–30^ solvent polarity,^31^ or ion pairing.^28,32^

**Scheme 1:**
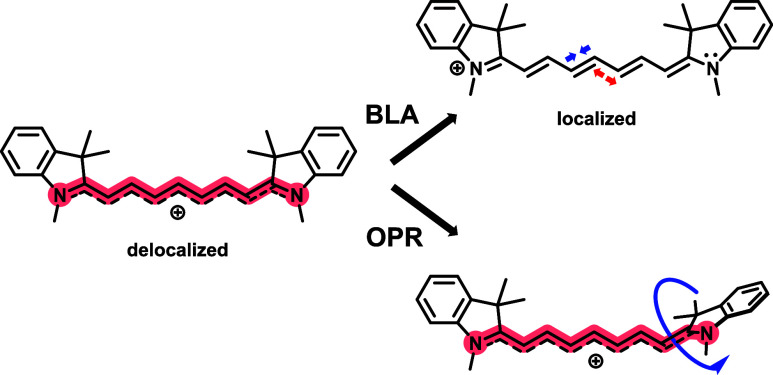
Schematic Representation of Bond Length Alternation of the Polymethine Chain (BLA) of Cyanine and Out-of-Plane Rotation of the Cyanine End Group (OPR)

Early theoretical works on hemicyanine dyes with the dimethyamino group on the end group showed that rotation around different chemical bonds, including those of the methine chain, leads to the formation of a TICT (twisted intramolecular charge transfer) state.^33^ Armitage, Yaron, and co-workers reported in their experimental-computational study that twisting about the monomethine bridge leads to a dark state that decays nonradiatively.^34^ It was also demonstrated that restraining the rotation of the CHO group in the *meso*-position of Cy5 in viscous or low-temperature media results in an increase in the fluorescence quantum yields.^35^ On the other hand, the length of C–N bond of amino group in an even position of the chain of cyanines has a double-bond character, its rotation is restricted,^36^ and its presence leads to a broken symmetry, nonplanar structure.^37^ Computational studies showed that the energy barriers of the C–C bond rotations strongly depend on the choice of substituents in the *meso*-position.^38^ We hypothesized that an important structural cyanine modification that could potentially change its electronic structure could be the forced distortion of the terminal heterocyclic rings out of the plane of the conjugated π-system, which we term out-of-plane rotation (OPR, Scheme 1). Significant electronic and structural deformations could be introduced by 1′-substituents next to the terminal heterocyclic rings, however, a reliable synthetic strategy for introducing the substituents was essentially unavailable.

The conventional approach to the synthesis of Cy5 and Cy7 derivatives involves the condensation of quaternary salts with malonaldehyde or glutaconaldehyde dianilide hydrochloride,^39–43^ respectively (Scheme 2A). Until recently, functionalization of the polymethine chain was primarily limited to the *meso*-position, the central chain carbon atom, achieved through nucleophilic substitution,^44^ or Pd-catalyzed coupling (Scheme 2B).^45^ General methods have been developed for the preparation of chain-substituted Cy7s, utilizing ring opening of pyridinium salt derivatives^46^ (Scheme 2C) and ring opening of furfural derivatives.^47^ Although these methods effectively functionalize the C3′, C4′, and C5′ chain positions, the possibilities for modifying the C1′ position remained very limited. 1′-Substituted trimethine cyanines (Cy3) have been reported,^48^ but examples of analogous 1′-substituted Cy5s are very limited.^49,50^ There is no general synthetic methodology for their preparation available. The synthesis of 1′-substituted Cy7 derivatives has not been reported.

**Scheme 2:**
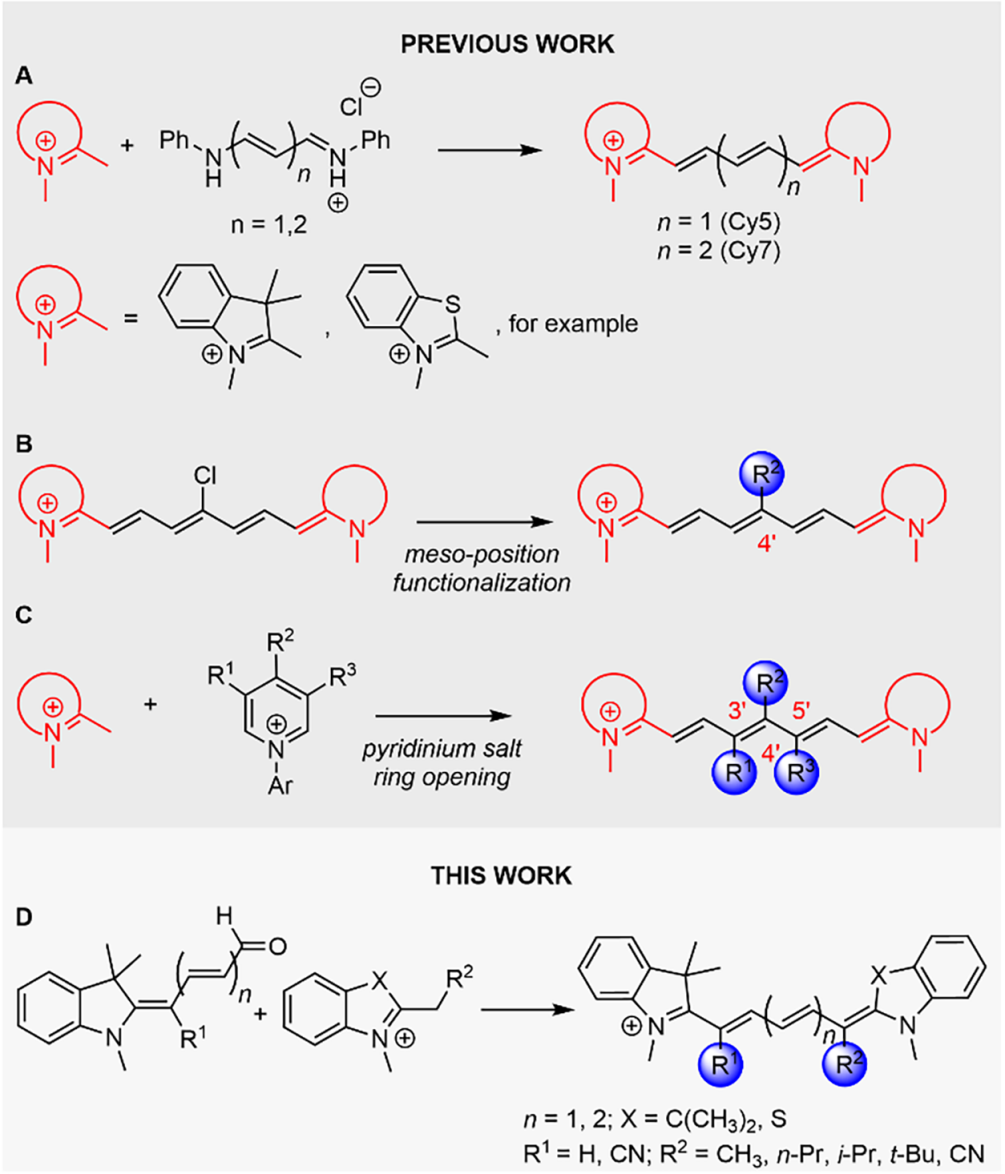
(A) Conventional Synthesis of Cy5 and Cy7 from Malonaldehyde or Glutaconaldehyde Dianilide Hydrochlorides; (B) Strategies for the Functionalization of the Cyanine *meso*-Position; (C) Synthesis of Chain Substituted Cy7s via Ring Opening of Pyridinium Salts; (D) Synthesis of 1′-Substituted Cyanines—This Work

Herein, we report a general approach for the functionalization of C1′ as well as both C1′ and C5′/C7′ positions of penta-/heptamethine cyanines by condensation of (1-methyl-1*H*-2-indol-2-ylidene)­but-2-enal or 6-(1-methyl-1*H*-indol-2-ylidene)­hexa-2,4-dienal derivatives with either substituted 1,3,3-trimethyl-2-methyleneindoline or 3-methyl-2-methylene-2,3-dihydrobenzo­[*d*]­thiazole as Fisher′s bases (Scheme 2D). Sixteen mono- and disubstituted cyanine derivatives bearing various electron-withdrawing (EWG), electron-donating (EDG), or sterically demanding 1′-substituents and the same or different heterocyclic ends have been prepared. The developed methodology utilized short reaction times under mild conditions and simple isolation. Furthermore, we present steady-state absorption and emission spectra of all synthesized derivatives along with measurements of singlet oxygen production efficiency, susceptibility to photooxygenation, and photostability. Experimentally found photophysical properties of the synthesized cyanines were systematically correlated with the changes in the electronic structure induced by structural modifications, rationalized using quantum chemical calculations. We demonstrate that substitution at the 1′-position of cyanine dyes allows a high level of control over their photophysical behavior with quantum-chemical analyses providing insight into the fundamental electronic changes.

## Results and Discussion

### Controlling Photophysics by Bond Length Alternation and Out-of-Plane Rotation

We begin by considering how structural modifications affect the electronic properties of cyanine dyes using quantum chemistry methods. Specifically, we focus on the impact of bond length alternation (BLA) and out-of-plane rotation (OPR, Scheme 1) induced by the 1′-position substituent or end heterocycle exchange. All calculated data presented here are based on semiempirical calculations at the IEF-PCM-ZINDO/S level of theory, assuming liquid-phase molecules optimized at the IEF-PCM-CAM-B3LYP/6–31+G** level of theory. Additional calculations using time-dependent density functional theory (TDDFT; Supporting Information) gave higher excitation energies, a well-described effect for cyanines.^13,51^ We also compare the results with more advanced SC-NEVPT2/cc-pVDZ calculations (Supporting Information). The mean absolute deviation (MAD) in vertical transition energies was 0.371 eV between IEF-PCM-ZINDO/S and IEF-PCM-TD-CAM-B3LYP/6–31+G** and 0.167 eV between IEF-PCM-ZINDO/S and SC-NEVPT2/cc-pVDZ (the deviations were calculated from the values in Table S2). Because the MADs were comparable between IEF-PCM-ZINDO/S and SC-NEVPT2/cc-pVDZ calculations, we decided to use the IEF-PCM-ZINDO/S approach for most of the calculations.

BLA reflects the Peierls distortion,^52^ a well-known phenomenon in cyanines.^25–27^ It arises spontaneously in some conjugated systems and can be artificially enforced in silico to investigate its impact on the electronic structure.^13^ Here, we analyzed the effects of BLA by applying controlled distortions to the fully symmetric Cy7 cyanine molecule. More specifically, we performed constrained optimizations in the ground state with frozen carbons on the polymethine chain. The distortion was imposed by varying the frozen bond lengths. First, we started with all bond lengths being identical (δ_BLA_= 0 Å), corresponding to the global minimal structure of the nonsubstituted cyanine Cy7. Then, we alternately prolonged/shortened the frozen bond lengths (Scheme 1) to scan across the BLA range δ_BLA_= 0.02–0.06 Å. The second structural modification, OPR (Scheme 1), describes the rotation of the terminal heterocyclic rings with respect to the plane of the conjugated π-system, changing the relative orientation of these groups, resulting in modification of the electronic transitions.

In the planar configuration (OPR = 0°), increasing BLA resulted in an increase in the S_0_ → S_1_ energy gap (Figure 1A). In addition, some spectral intensity was transferred to higher-energy transitions, particularly the S_0_ → S_3_ transition, which is consistent with expectations from the particle-in-a-box model.^24^ In contrast to BLA, the out-of-plane rotation introduced more pronounced excitation energy shifts, affecting both the S_0_ → S_1_ and S_0_ → S_2_ transitions (Figure 1B). While the S_0_ → S_1_ energy gap increased, the S_0_ → S_2_ gap decreased, resulting in a bathochromic shift of the corresponding absorption bands. Therefore, OPR induced a significant redistribution of intensity between the bands. In the planar configuration, the S_0_ → S_1_ transition is optically allowed, while the S_0_ → S_2_ transition remains forbidden (Figure 1B). As the structure became perpendicular, S_0_ → S_1_ lost intensity and S_0_ → S_2_ became more intense. This behavior arises from a shift in the character of the molecular orbitals involved in these transitions as the localized electronic excitation changes to a charge-transfer state.^25^ In a fully perpendicular orientation, the S_0_ → S_1_ transition is completely forbidden, confirming the critical role of OPR in the modulation of cyanine photophysics.

**Figure 1:**
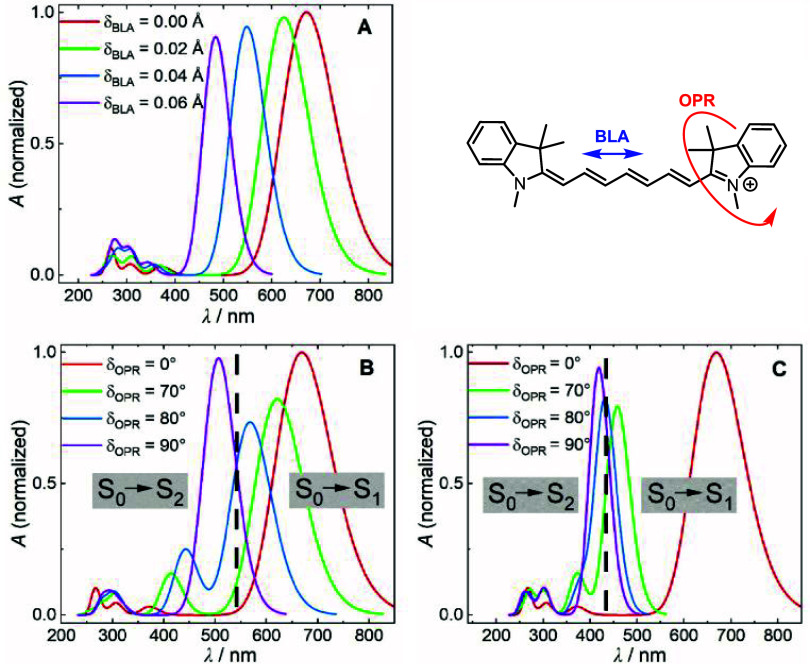
(A) Calculated UV/vis spectra of heptamethine cyanine in the planar conformation for different degrees of BLA: nonalternated (red), moderately alternated (green: BLA = 0.02 Å), and highly alternated (blue: BLA = 0.04 Å; violet: BLA = 0.06 Å). (B) Calculated UV/vis spectra of fully symmetric Cy7 as a function of OPR of the terminal heterocyclic group. The spectra correspond to different rotational states: planar orientation (red: OPR = 0°), highly rotated (green: OPR = 70°), near-perpendicular orientation (blue: OPR = 80°), and fully perpendicular orientation (violet: OPR = 90°). (C) Calculated UV/vis spectra of fully symmetric Cy7 as a function of OPR, incorporating OPR-induced BLA through constrained optimization. The color scheme follows the same convention as that in panel B.

The combined effect of OPR and BLA revealed a more complex relationship between structural changes and electronic properties. Because distortions of the terminal heterocycle can induce BLA, we performed partial optimizations with fixed OPR, while the other coordinates were allowed to relax (Figure 1C). The results showed that increasing OPR consistently induced BLA, leading to a distinct single-bond character between the C1′ carbon and the terminal heterocycle. This structural change hypsochromically shifted and weakened the first band while strengthening the second. Up to OPR = 80°, the intensities changed only slightly. From this point onward, there was a rapid increase in the S_0_ → S_2_ band intensity and a significant decrease in the S_0_→S_1_ band intensity, making the transition forbidden for the terminal group rotating to the perpendicular position.

The electronic origin of these intensity changes can be traced back to the nature of the S_0_ → S_2_ transitions. In the planar structure, the excited-state electron density remains localized on the polymethine chain. As OPR increases, the S_2_ electron density progressively shifts toward the terminal heterocycle (Figure 2), transforming the transition into an optically allowed charge transfer state.

**Figure 2:**
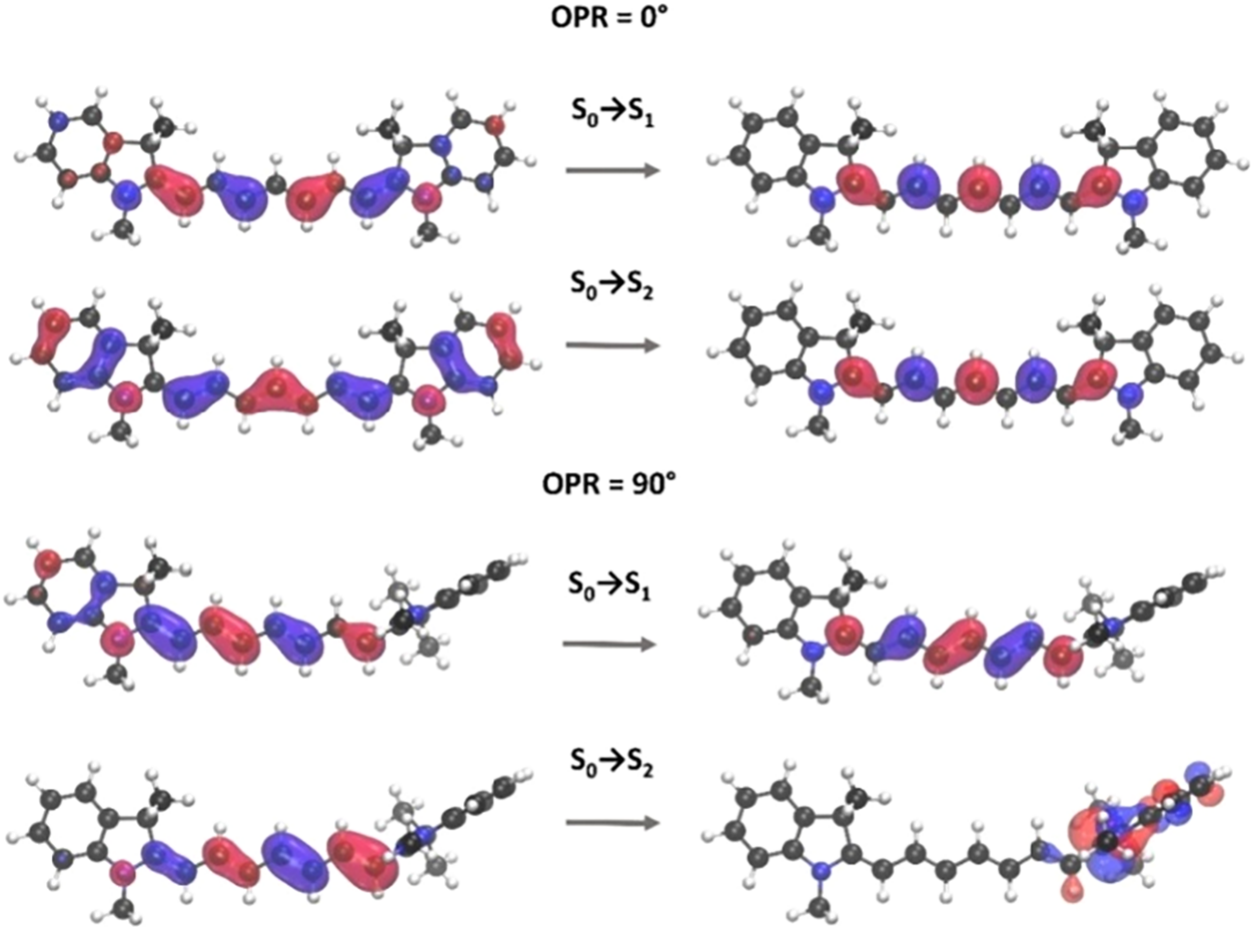
Natural Transition Orbitals (NTOs) characterizing S_0_ → S_1_ and S_0_ → S_2_ excitations for OPR = 0° (top) and OPR = 90° (bottom). The remaining coordinates were optimized. The orbitals were visualized using a contour threshold of 0.03.

Guided by these insights, we hypothesized that substituents on the polymethine chain, through their electronic and steric effects, could induce both OPR and BLA effects much more effectively than substituents at other chain positions, and it could be achieved without modifying the length of the polymethine chain. Therefore, we developed a synthesis method for 1′-substituted Cy7s and Cy5s and studied their physicochemical properties alongside quantum-chemical predictions.

### Synthesis

Reports on the synthesis of Cy3 and Cy5 derivatives bearing a substituent at the 1′-position are rare and nonexistent for Cy7. Wang and co-workers presented an elegant method for the direct functionalization of the 1′-position of Cy3 via an electrophilic substitution reaction^48^ (Scheme 3A). Unfortunately, this approach is not applicable to Cy5 and Cy7, where electrophilic substitution leads to the functionalization of the *meso*-position.^53^ An interesting approach was presented by Hamer and co-workers, who described the synthesis of 1′-substituted Cy5 via the reaction of 2-ethyl-benzothiazolium salts with β-ethoxyacraldehyde acetal^49^ in pyridine (Scheme 3B).

**Scheme 3:**
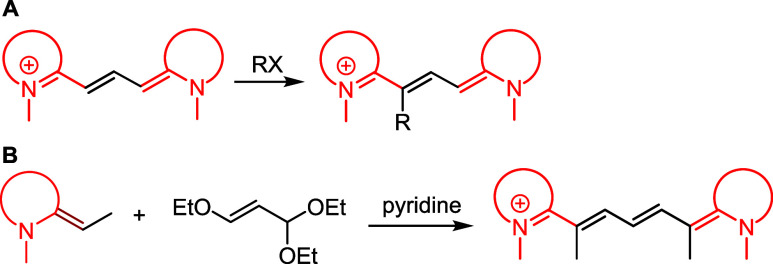
(A) Synthesis of 1′-Substituted Cy3 by Electrophilic Substitution; (B) Condensation of 2-Ethyl-benzothiazolium Salt with β-Ethoxyacraldehyde Acetal

In this work, we focused on the synthesis of Cy5/Cy7 derivatives with C1′ and C5′/C7′ substituents, such as methyl, *n*-propyl, *i*-propyl, *t*-butyl, and cyano groups (compounds **1**–**17**, Table 1; compound **1** is the parent Cy7 molecule). Two different heterocyclic end groups, indolenine and benzothiazole moieties, were used due to their prevalent use in biological applications.^10^

**Table 1:** Cyanine Derivatives Used and Prepared in This Work

cyanine	*n*	X	R^1^	R^2^	yield/%
**1**	1	C(CH_3_)_2_	H	H	n.a.^a^
**2**	1	S	H	H	46
**3**	1	C(CH_3_)_2_	Me	H	49
**4**	1	S	*n*-Pr	H	65
**5**	1	C(CH_3_)_2_	*i*-Pr	H	40
**6**	1	S	*i*-Pr	H	55
**7**	1	C(CH_3_)_2_	CN	H	51
**8**	1	S	CN	H	90
**9**	1	C(CH_3_)_2_	CN	CN	91
**10**	2	C(CH_3_)_2_	H	H	60
**11**	2	S	H	H	25
**12**	2	C(CH_3_)_2_	Me	H	75
**13**	2	S	*n*-Pr	H	35
**14**	2	S	*i*-Pr	H	64
**15**	2	S	*t*-Bu	H	25
**16**	2	C(CH_3_)_2_	CN	H	65
**17**	2	C(CH_3_)_2_	CN	CN	65

a Commercially available.

Initial attempts to prepare the 1′,5′-dimethyl Cy5 (**9**) and 1′,7′-dimethyl Cy7 (**17**) using commercially available dianilide hydrochloride derivatives of malonaldehyde (**18** *n* = 1) or glutaconaldehyde (**19**, *n* = 2), respectively, with 2-ethylideneindolium triflate (**21**),^54^ prepared by methylation from 2-*i*-butyl-3,3-dimethyl-3*H*-indole^54^ (**20**) under basic conditions (forming a Fischer’s base;^55^ Scheme 4A) and high temperature, resulted in the degradation of the starting material (Scheme 4B). Dianilide hydrochloride derivatives have already been used for the synthesis of unsubstituted Cy5 and Cy7.^41^ Fortunately, the use of Fisher’s base, 2-(1,3,3-trimethylindolin-2-ylidene)­acetonitrile (**22**), directly did afford the corresponding 1′,7′- and 1′,5′-cyano derivatives in high yields (Scheme 4B). **22** was prepared by the reaction of 1,3,3-trimethyl-2-methyleneindoline with sodium thiocyanate followed by basic hydrolysis.^56^ The good thermal stability of both **22** and the dianilide hydrochloride precursor in the absence of base probably afforded this compound without significant degradation.

**Scheme 4:**
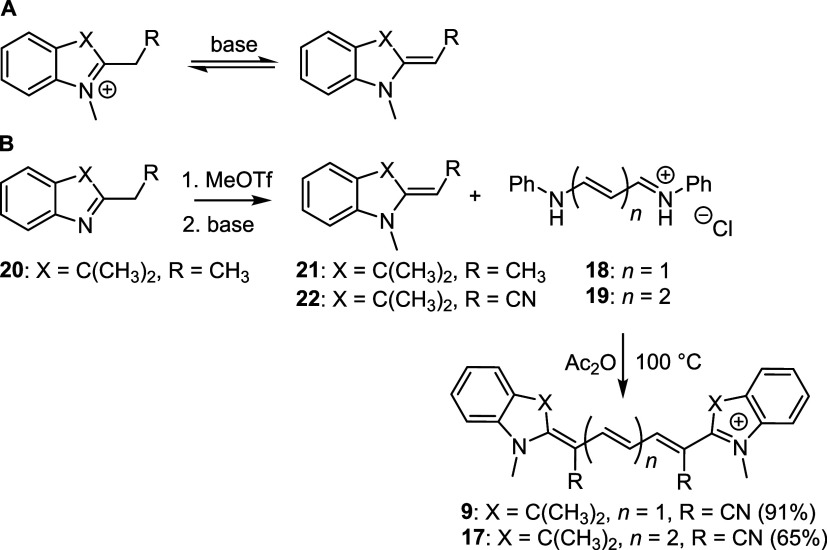
(A) Acid–Base Equilibrium of a Fisher’s Base; (B) Synthesis of Cyanines **9** and **17**

Then we turned our attention to indol-2-ylidene butenal **23**,^57^ as a precursor for the preparation of Cy5 (Scheme 5A). Previous studies have shown that 3-dimethylaminopropenal can undergo the Vilsmeier reaction to generate reactive intermediates,^58,59^ which can be trapped by electron-rich moieties, such as (3-hydroxypropenyl)-1-(toluene-4-sulfonyl)-1*H*-indole-5-carbonitrile^29^ and other indole derivatives.^60,61^ The conversion of enaminaldehydes to their iminium salts has been reported to give chloropentadiene iminium derivatives,^58,59,62^ and a similar strategy was used for the synthesis of 1′-cyano Cy3.^50^ Inspired by these findings, (indolin-2-ylidene)­butenal **23** was converted to the corresponding vinyl chloride that gave **8** in the presence of a Fischer’s base in high yield.

**Scheme 5:**
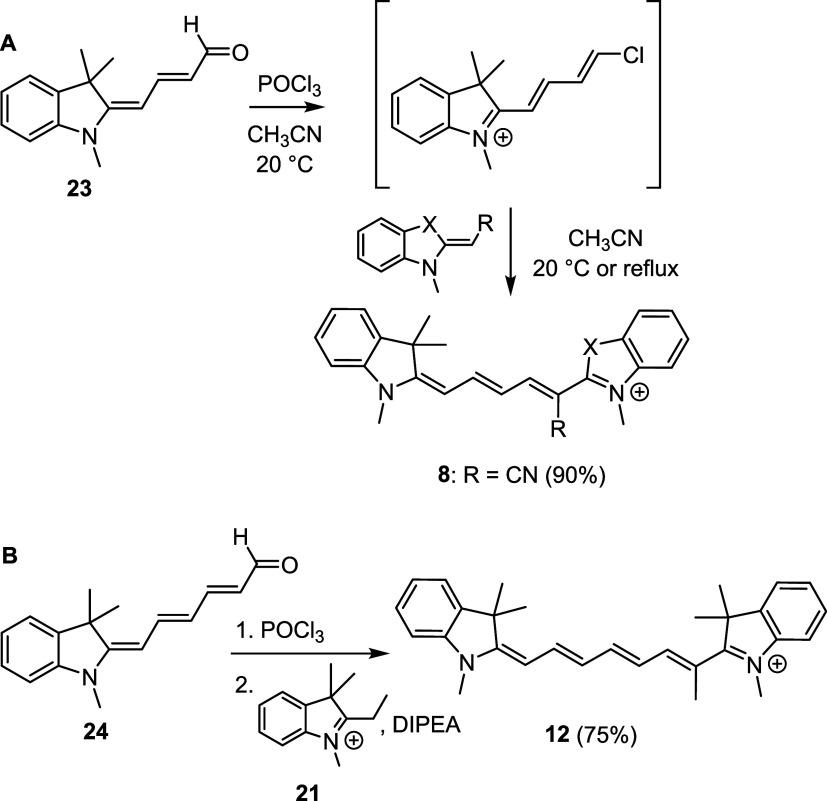
(A) Synthesis of 1′-Substituted Cy5s via a Vinyl Chloride Intermediate Using 2-Methylene Derivatives as Fisher’s Bases; (B) Synthesis of 1′-Substituted Cy7s Using Quaternary Salts in the Presence of a Base (Diisopropylethylamine, DIPEA)

In general, Fischer′s bases are difficult to manipulate due to their reactivity.^63^ For the preparation of monosubstituted Cy7 derivatives listed in Table 1, they were generated from the corresponding indolinium- and benzothiazolium-based quaternary salts (Scheme 4A). These salts were prepared from the corresponding 2-substituted 3,3-dimethyl-3*H*-indole or benzo­[*d*]­thiazole derivatives by *N*-methylation with methyl triflate in diethyl ether (Supporting Information). They are easy to purify and benchtop-stable for months. The generated Fisher’s base then reacts with a Vilsmeier intermediate to produce the desired cyanine (Scheme 5B). We optimized the synthesis of the reported 2-ethylidene indolium **21** ^54^ using bases of different strengths, AcONa, NaH, Et_3_N, and diisopropylethylamine (DIPEA), to find DIPEA as the most effective reagent. For 1′-methyl Cy7 **12**, for example, 2-ethylidene indolium **21** was added after the vinyl chloride intermediate was formed, and DIPEA was introduced dropwise to the reaction mixture. This immediately produced a dark blue-green solution indicating the successful formation of the Cy7 derivative. Overall, we prepared 13 new 1′-substituted Cy5 and Cy7 derivatives in good yields using this general procedure (Table 1, and Supporting Information). The main advantage of this method is the easy access not only to monosubstituted but also to disubstituted Cy5 and Cy7 with the same or different heterocyclic ends. Only the derivative **16** was prepared by lithiation of **22** with *n*-BuLi and subsequent condensation with the aldehyde **24**. After acidic treatment, **16** was obtained in good yield (Supporting Information).

### Absorption Properties

The parent unsubstituted Cy7 (**10**) features a rigid and planar structure.^64^ Its efficient and symmetric HOMO–LUMO orbital overlap, localized within the polyene system, is reflected in an absorption spectrum characterized by a narrow band at 740 nm. The presence of a secondary sub-band around 690 nm was attributed to vibronic transitions from the S_0_ state to higher vibrational levels of the S_1_ excited state.^17^ While it has been demonstrated that substituents on the cyanine chain in the 3′- and 4′-positions can influence the positions and shape of absorption maxima,^13^ we show in this work that the effects of 1′-substituents are significantly more pronounced in cyanines functionalized at the 1′-position. The absorption maxima of the prepared cyanines span in the wavelength ranges of approximately 400–700 nm for Cy5 and 400–750 nm for Cy7 derivatives (Figure 3 and Table 2), thus, some substituents induced significant hypsochromic shifts. The effects of bulky alkyl groups (**5**, **6**, **14**, and **15**) are much more pronounced than that of the electron-withdrawing cyano group. However, a hypsochromically shifted broader band with a maximum at 620 nm of the monocyano derivative **16** is changed to a narrow band at 704 nm in symmetric dicyano Cy7 derivative **17**, the spectrum of which closely resembles that of unsubstituted Cy7. The spectra of *i*-Pr- and *t*-Bu-substituted Cy5 and Cy7 possess two significant absorption bands of comparable intensity.

**Table 2:** UV–Vis Absorption and Emission Properties of 1′-Substituted Cyanines

cyanine	λ^abs^/nm^a^	λ_calc_^abs^/nm^b^	λ^em^/nm^a^	ε_max_/10^5^ ^c^	Φ_F_ ^d^
**1**	638	585	665	1.96	0.0165 ± 0.0100
**2**	640	594	671	2.86	0.205 ± 0.007
**3**	649	608	680	1.56	0.0098 ± 0.0010
**4**	628	571	679	0.78	0.0077 ± 0.0010
**5**	374, 652	349, 610	688	0.39	0.001 ± 0.000
**6**	375, 561	354, 568	679	0.30	0.001 ± 0.000
**7**	599	545	645	0.69	0.0471 ± 0.002
**8**	616	569	652	1.14	0.037 ± 0.003
**9**	610	564	671	1.06	0.007 ± 0.001
**10**	740	669	769	2.39	0.158 ± 0.021
**11**	744	671	780	1.58	0.091 ± 0.006
**12**	748	685	789	1.46	0.013 ± 0.000
**13**	638	635	790	0.51	0.052 ± 0.009
**14**	409, 533	382, 568	785	0.59	0.001 ± 0.000
**15**	433	413	771	0.45	0.001 ± 0.000
**16**	620	580	744	0.57	0.098 ± 0.014
**17**	704	641	744	1.50	0.0122 ± 0.0050

a Absorption (λ^abs^) and emission (λ^em^) maxima found in methanol at room temperature (see the Supporting Information for complete spectra). The concentrations were adjusted to have absorbance in the interval of 0.2–1.0. Two main absorption maxima are listed for some derivatives.

b Calculated vertical transition wavelengths (λ_calc_^abs^) at the IEF-PCM-ZINDO/S level of theory.

c Molar absorption coefficients, ε_max_/mol^–1^ dm^3^ cm^–1^, obtained in methanol.

d Fluorescence quantum yield (Φ_F_) was measured in methanol and obtained as an absolute value using an integrating sphere (λ^exc^ always at the long-wavelength λ^abs^ maximum). The concentrations were adjusted to have an absorbance below 0.15. The excitation wavelength was set at the absorption maxima. Averages of three independent measurements; standard deviation of the mean is given.

**Figure 3:**
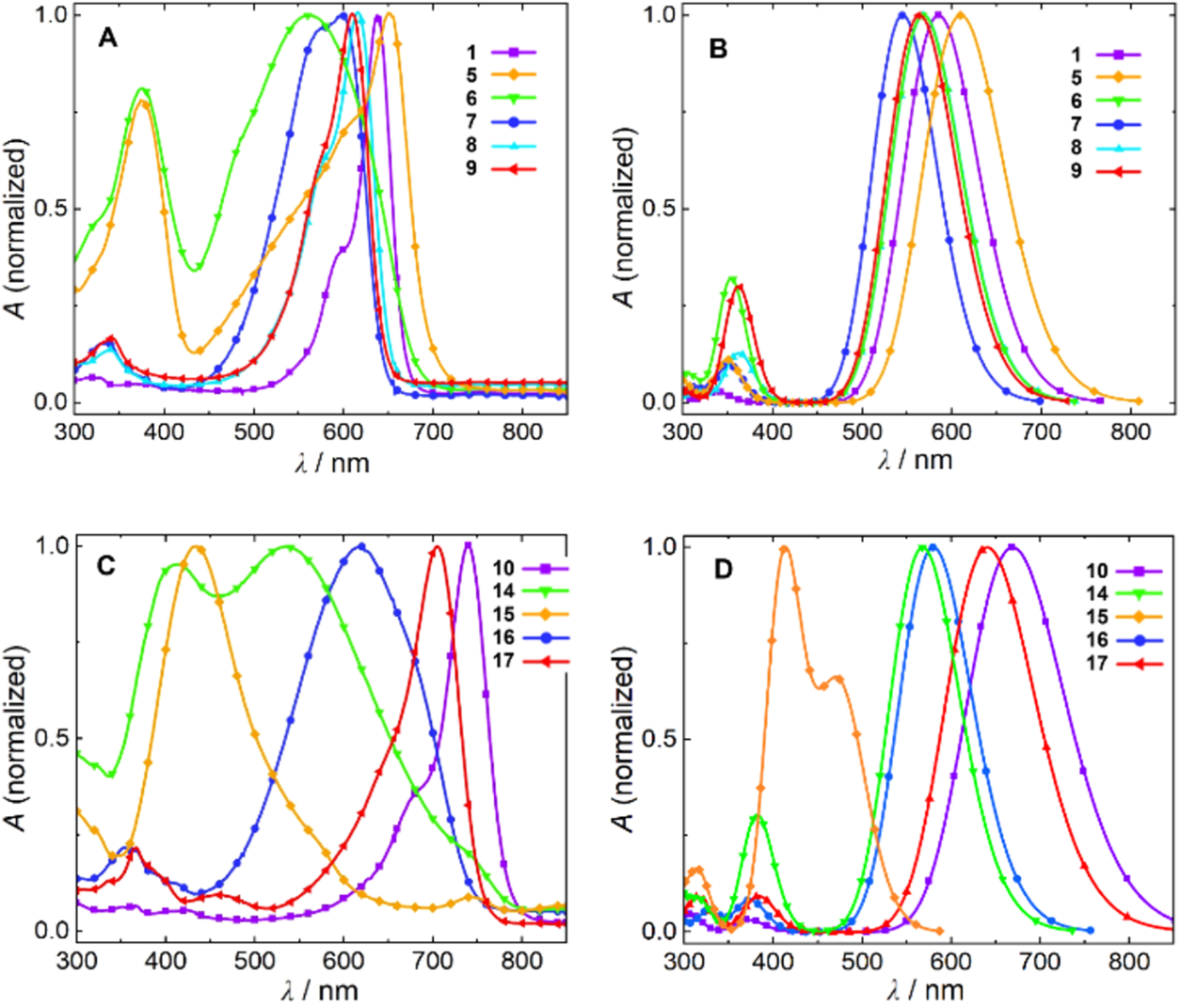
Absorption spectra of selected 1′-substituted Cy5 (A: experimental, B: calculated) and Cy7 (C: experimental, D: calculated) derivatives.

### Understanding the Absorption Spectra: Substitution Controls both OPR and BLA

Using CAM-B3LYP optimized structures in dielectric continuum, we connected substituent-driven symmetry breaking to the observed spectra (Table 2). We focused on two key modifications: variation of the heteroatom X in one of terminal heterocycle and the R^1^ substitution at the C1′ position.

The R^1^ substituents were responsible for three major structural and electronic modifications. First, they induced BLA, the extent of which depended on the electrophilicity of the substituent. Second, they influenced the bond angles at C1′, the carbon adjacent to the =C–C= moiety, due to steric effects, thereby introducing local geometric strain. Third, the R^1^ groups facilitated OPR of the terminal heterocyclic group along the C1′–C1 axis, further enhancing BLA. The effects of the substituent X (i.e., the end group) were generally smaller. These structural changes affect the absorption spectra. Indeed, the experimental and calculated absorption maxima for the Cy5 and Cy7 derivatives, presented in Figure 3A, showed good agreement. The calculated spectra lack vibronic structures, as the theoretical approach incorporates empirical broadening of the absorption wavelengths at the optimized molecular geometry.

### Electronic Effects in the 1′-Position

Substitution in the 1′-position with an electron-withdrawing cyano group induced two key geometrical effects. First, the electron-withdrawing nature of the CN group pulls the electron density, leading to BLA along the polymethine chain. For example, the CN-substituted Cy7 (16) displays a distinct alternating bond pattern (*r*_avg(singlebond)_ = 1.412 Å; *r*_avg(doublebond)_ = 1.377 Å), in contrast to the nearly uniform bond lengths observed for 10 (*r*_avg_ = 1.391 Å). Second, the relatively small size of the CN group induces only a modest OPR (15°). Both BLA and OPR contribute to the observed blue shift of the S_0_ → S_1_ absorption band. However, the spectral impact of the 15° OPR is small, as the shift induced by OPR becomes significant only at larger torsional angles (Figure 1). The BLA-induced shift exceeds that of an unsubstituted cyanine with the same imposed BLA (Figure S83), confirming the strong chain–CN coupling. Analogous spectral shifts were also observed for the unsubstituted and substituted Cy5s, **1** (*r*_avg_ = 1.392 Å) and **7** (*r*_avg(singlebond)_ = 1.415 Å; *r*_avg(doublebond)_ = 1.384 Å; OPR = 15°), respectively. The hypsochromic shift observed for the mono-CN derivatives disappears in 1′,7′-dicyano derivative 17 as the structure regains its symmetry (Figure 3).

### Electronic Effects of the Ending Group (X Substituent)

Cyanine bearing different terminal heterocycles, 2-methylene-indoline and 2-methylene-2,3-dihydrobenzo­[*d*]­thiazole, breaks the molecular symmetry and induces BLA along the polymethine chain. The symmetric structure has a bond between the central carbons with *r*_avg_ = 1.391 Å, while the asymmetric structure exhibits a minor alteration between single (*r*_avg_ = 1.400 Å) and double bonds (*r*_avg_ = 1.385 Å). Furthermore, the BLA induced in benzothiazole derivative is rather limited. The geometry-induced spectral changes are compensated by electronic effects, leaving the S_0_→S_1_ band maximum unchanged (Figure S83). The effect of the X substitution in the terminal heterocycle in Cy5s can also be investigated by comparing 1′-cyano Cy5 **7** (X = C­(CH_3_)_2_) with the analogous benzothiazole derivative **8** (X = S). The sulfur substitution induces BLA that compensates for the electrophilicity-driven BLA associated with the cyano group (Figure 3). As a result, **8** exhibits a decrease in BLA (*r*_avg(singlebond)_ = 1.409 Å; *r*_avg(doublebond)_ = 1.390 Å), thereby slightly reducing the S_0_ → S_1_ energy gap (Figure 3A). Overall, the spectral change is negligible in both theory and experiment.

### Steric Effects in the 1′-Position

Next, we discuss the effect of bulky 1′-substituents, namely, *i*-Pr (**14**) and *t*-Bu (**15**), where both OPR and BLA effects are pronounced (Figure 3 and Table 2). Both structures contain sulfur at the X position, which promotes minor BLA along the polymethine chain. The *i*-Pr group introduces OPR = 41°, whereas the *t*-Bu substituent triggers OPR = 79°. Such high OPR values induce non-negligible BLA, which enhances BLA induced by X substitution, resulting in *r*_avg (singlebond)_ = 1.431 Å and *r*_avg(doublebond)_ = 1.367 Å for **14** and *r*_avg(singlebond)_ = 1.446 Å and *r*_avg(doublebond)_ = 1.359 Å for **15**. The combination of both OPR and BLA effects contributes to a significant hypsochromic shift of the S_0_ → S_1_ absorption band. Because of extreme OPR, dual S_0_ → S_1_ and S_0_ → S_2_ bands are observed, with the latter dominating for the *t*-Bu derivative (Figure 3).

Next, we discuss the effect of polymethine chain length in Cy5 **6** and Cy7 **14**, bearing *i*-Pr as a substituent. The structures are comparable in the electronic ground state, exhibiting similar BLA (*r*_avg(singlebond)_ = 1.426 Å and *r*_avg(doublebond)_ = 1.373 Å for **6**), and OPR (δ_OPR_ = 42.6 ° and δ_OPR_ = 45.7 ° for **6** and **14**, respectively). Furthermore, the quantum-chemistry calculations suggest very similar spectra of those two derivatives (Figure 3B,D), indicating that polymethine chain length does not affect the spectral shape significantly. The spectra similarity originates from comparable BLA and OPR values. Nevertheless, the measured spectra differ (Figure 3A,B). We assume that vibronic effects play an important role in reshaping the spectra, which we do not take into account here. We show the scale of vibronic effects in Figure S83 for structure **5**.

Finally, we discuss the effects of the benzothiazole group on the structure of Cy5 bearing the same bulky 1′-*i*-Pr substituent. More pronounced BLA and OPR in **6** (*r*_avg(singlebond)_ = 1.426 Å, *r*_avg(doublebond)_ = 1.374 Å, OPR = 42°) result in a stronger hypsochromic shift of the first absorption band (Figure 3 and Table 2) and alternate more the polymethine chain than observed in indoline derivative **5** (*r*_avg(singlebond)_ = 1.405 Å, *r*_avg(doublebond)_ = 1.389 Å, OPR = 26°).

### Solvent Effects

Cyanine dyes were shown to exhibit small hypsochromic shifts in their absorption spectra with increasing solvent polarity,^65,66^ reflecting differential stabilization of the ground/excited state.^67^ Increasing the polarity of the solvent is also accompanied by a band broadening represented by the growth of the short-wavelength shoulder.^66,68^ It has been suggested that broad absorption bands in the blue region are related to the broken-symmetry ground state.^27,69,70^

Here, we recorded the absorption spectra of several 1′-substituted Cy5 and Cy7 derivatives. The solvent polarity played a significant role in the relative intensity ratio of the two major absorption bands of **5** (Figure 4A), where the band at ∼380 nm and the shoulder in the 500–600 nm region were more pronounced in polar solvents. This would be consistent with the partial charge-transfer character of the second transition, although the intensity change was not found in the calculated data, except for a small difference for **6** between dichloromethane and more polar solvents (Figures S85 and S86). A significant hypsochromic shift and the appearance of a new band at ∼550 nm were observed for **6** when dichloromethane was replaced by solvents of higher polarity (Figure 4B). This shift was also reproduced by calculations. We observed changing vibronic contributions in the first absorption band, indicating different structural changes in the S_1_ state in different solvents (Figure S83). Other cyanine derivatives, such as **4**, **15**, **14**, and **16**, also exhibited different absorption spectra in dichloromethane compared to those in more polar solvents (Figures S85 and 86).

**Figure 4:**
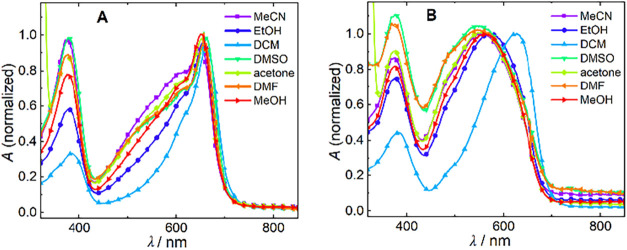
Absorption spectra of (A) **5** and (B) **6** in acetonitrile (MeCN), ethanol (EtOH), dichloromethane (DCM), dimethyl sulfoxide (DMSO), acetone, dimethylformamide (DMF), and methanol (MeOH).

### Emission Properties

The unsubstituted Cy7 (**10**) exhibits fluorescence with a quantum yield (Φ_F_) of approximately 0.16 in methanol at room temperature. This relatively low value is attributed to efficient nonradiative processes that compete with radiative deactivation of the excited state, with photoisomerization identified as the primary pathway for S_1_ state deactivation;^71^ the quantum yield can be tuned by some conformational constraints.^72^ The 1′-substituted dyes are less fluorescent than their unsubstituted counterparts, only the cyano derivatives **16** and **17** were somewhat more emissive (Φ_F_ < 0.1; Table 2). Cy7s have short fluorescence lifetimes (τ_F_), usually between 0.30 to 2 ns (e.g., τ_F_ of indocyanine green (ICG) in methanol is 0.52 ns^16^). τ_F_ of compounds **5**, **6**, and **14** in methanol was found to be biexponential for **14** with τ_1_ = 0.14 ± 0.01 ns and τ_2_ = 3.70 ± 0.32 ns (Figure 78A and Table S1). Therefore, we decided to investigate in more depth the emission properties of derivative **14**, which possesses two main absorption bands at 409 and 533 nm (Table 2). When measured at −78 and −173 °C, **14** showed τ_1_ = 1.11 and 1.09 ns and τ_2_ = 5.71 and 4.05 ns, respectively; thus, the value of τ_1_ prolonged by an order of magnitude at low temperatures. The emission bands of **14** obtained with excitation at 410/540 nm (λ^em^ ∼ 785) did not match that measured with the excitation at the tail of the absorption band (730 nm; λ^em^ ∼ 765 nm; Figure 5; see also an excitation–emission matrix of **14** in Figure S78B). The Φ_F_ in **14** at λ_exc_ = 410 nm (S_2_ state) was found negligible, whereas 0.04 was obtained at λ_exc_ = 730 nm. At low temperatures, all emission bands were found in the same region. In addition, different excitation spectra were obtained at different λ_em_ (Figure S78B), suggesting the presence of two distinct emissive species that contribute to the fluorescence emission. Indeed, the multiexponential fluorescence decay and temperature-dependent emission band shifts in unsubstituted polymethine dyes have been attributed to symmetrical and unsymmetrical relaxation pathways.^73,74^ This hypothesis was proven using variable-temperature nuclear magnetic resonance (NMR) to study conformational exchange processes.^75,76^ The ^1^H NMR spectra of **14** at room temperature showed a single species, whereas a mixture of two isomers was observed at −40 °C (Figure S78C). According to the computational calculations presented, the major isomer was attributed to the *Z*-isomer, which predicted significant geometric constraints for sterically demanding substituents. We conclude that the absorption bands at 410 and 540 nm belong to this isomer, although it exhibits only weak fluorescence. The minor *E*-isomer is responsible for the small absorption band at 730 nm; it is less twisted and therefore more emissive.

**Figure 5:**
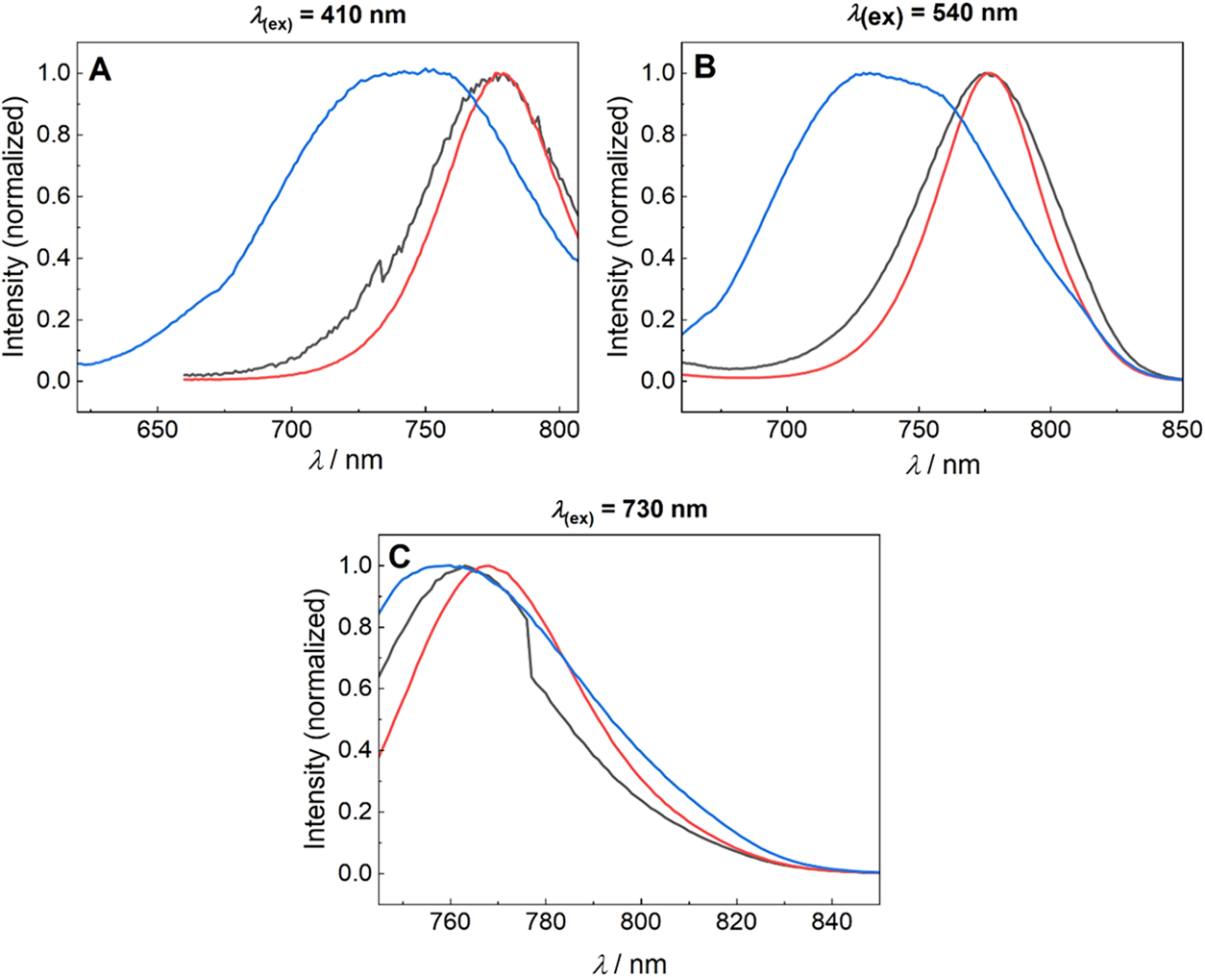
Emission spectra of **14** at 293 K (black line), 195 K (red line), and 100 K (blue line) at (A) λ_(ex)_ = 410 nm, (B) 540 nm, and (C) 730 nm.

The emission maxima of the cyanines studied were not significantly affected by the 1′-substituents as significantly as the absorption maxima (Table 2), increasing significantly Stokes shifts in some derivatives. The results are in good agreement with our quantum-chemical calculations (IEF-PCM-TD-CAM-B3LYP/6–31+G**). For example, the calculated emission wavelengths of the **14** and **15** derivatives bearing bulky substituents were 724 and 709 nm, respectively, which do not significantly differ from that of unsubstituted Cy7 (λ_em_ = 702 nm). The absence of a blue shift of the emission bands must be related to the structural planarization and the loss of BLA in the relaxed S_1_ state. For example, the *t*-Bu derivative **15** exhibits δ_OPR_ = 73.5° and 40.8° in the S_0_ and S_1_ states, respectively, and *r*_avg(singlebond)_ = 1.405 Å and *r*_avg(doublebond)_ = 1.400 Å in the S_1_ state. Therefore, BLA- and OPR-induced blue shift is effectively reduced, leaving the emission maximum similar to that of unsubstituted Cy7.

### Singlet Oxygen Production

Cyanine dyes can act as singlet oxygen (^1^O_2_) sensitizers in photodynamic therapy (PDT).^53,77^ Strategies to enhance ^1^O_2_ production from these dyes are well-documented, for example, by incorporating heavy atoms into the dye structure.^14,77^ Here, we determined the quantum yield of singlet oxygen production (Φ_Δ_) in methanol using diphenylisobenzofuran (DPBF) as a singlet oxygen trap (Table 3), with unsubstituted Cy7 serving as the reference ^1^O_2_ generator.^13^ Methylene blue (Φ_Δ_ = 0.49)^78^ was used as a reference ^1^O_2_ generator. For 1′-substituted cyanines, the Φ_Δ_ values were comparable to those of the unsubstituted dyes; in general, a lower ^1^O_2_ production was observed for Cy5 derivatives.

**Table 3:** Photochemical Properties of 1′-Substituted Cyanines^a^

Cy	Φ_Δ_/10^–3^ ^b^	Φ_dec_/10^–7^ ^c^
**1**	1.9 ± 0.3	3.7 ± 0.1
**2**	4.0 ± 0.5	14.0 ± 0.6
**3**	0.39 ± 0.06	4.4 ± 0.7
**4**	0.55 ± 0.05	12.5 ± 1.0
**5**	n.d.^d^	6.2 ± 0.6
**6**	n.d.^d^	56.4 ± 2.6
**7**	0.48 ± 0.06	2.6 ± 0.4
**8**	1.32 ± 0.10	0.8 ± 0.1
**9**	2.1 ± 0.2	3.1 ± 0.6
**10**	8.9 ± 0.1	31 ± 2
**11**	18.3 ± 2.1	120.0 ± 16.1
**12**	2.9 ± 0.4	32 ± 7
**13**	5.9 ± 0.5	190 ± 30
**14**	n.d.^d^	102.0 ± 5.4
**15**	n.d.^d^	n.d.^d^
**16**	5.2 ± 0.8	7.4 ± 0.3
**17**	6.3 ± 0.9	0.54 ± 0.10

a In methanol. The general cyanine structure is shown in Table 2.

b Quantum yields of the ^1^O_2_ production (Φ_Δ_) determined relative to that of methylene blue (Φ_Δ_ = 0.49)^79^ for Cy5 derivatives or **1** (Φ_Δ_ = 8.9 × 10^–3^)^13^ for Cy7 derivatives.

c Quantum yields of photodecomposition (Φ_dec_) calculated relative to **1** (Φ_dec_ = 3.7 × 10^–7^; see the Supporting Information) and **10** (Φ_dec_ = 3.1 × 10^–6^).^13^ Averages of three independent measurements; standard deviation of the mean is given.

d Not measured.

### (Photo)­stability

Photobleaching is a critical factor in the use of fluorophores for biological applications.^80^ Cyanines are known for their photosensitivity,^81^ creating a strong demand for cyanine derivatives with improved stability under irradiation.^29,82,83^ The photosensitization of ^1^O_2_ is considered to be the primary degradation pathway, leading to dioxetane formation and subsequent cleavage of the polyene chain.^10^ In this work, photodecomposition quantum yields (Φ_dec_) were determined for most derivatives in methanol (Table 3). In general, 1′-functionalized Cy5s showed the same or slightly lower photostability compared to their unsubstituted analogs. A similar trend was found for Cy7 derivatives, with one notable exception, **16**, which had four times lower Φ_dec_ than the unsubstituted Cy7. The synthesized compounds were shown to be stable in the dark in methanol solutions left overnight.

## Conclusions

Here, we present a versatile and efficient synthetic approach for introducing substituents at the C1′ chain position of pentamethine and heptamethine cyanines, which offers specific control over their structural and photophysical properties. By systematically varying the electronic and steric nature of these substituents, we uncovered two symmetry-breaking mechanisms—bond length alternation (BLA) and out-of-plane rotation (OPR)—that determine their optical nature. Our quantum-chemical and spectroscopic analyses revealed that OPR acts as an independent driver of BLA, particularly when the chain-end group dihedral angle increases. In this regime, OPR-induced BLA surpasses the BLA caused by electronic effects such as substituent electron deficiency, leading to a redistribution of bonding character along the polymethine chain. This deviation from the canonical cyanine conjugation explains the observed modulation of absorption properties and electronic transition intensities. The geometric distortion induced by OPR can effectively suppress the electronic effects of substituents, highlighting the delicate balance between their steric and electronic contributions.

Substitution at the 1′ position allows for tuning absorption spectra, fluorescence intensity, singlet oxygen generation, and photostability. For instance, electron-withdrawing cyano groups not only change the absorption spectrum but also affect excited-state lifetimes and resistance to photobleaching. This study establishes C1′ chain substitution as a powerful design handle for engineering cyanine dyes beyond traditional strategies and paves the way for the rational development of cyanines with tailored optical properties for various applications, for example in bioimaging, phototherapy, or optoelectronics. Selecting an appropriate absorption spectrum for fluorescence imaging offers a technological advantage. For example, two distinct absorption bands or broad absorption of a single dye can meaningfully advance the field of dye-sensitized solar cells. Furthermore, large Stokes shifts are valuable in bioimaging.

## Experimental Section

### Synthesis

#### Synthesis of Substituted Benzothiazoles and Indoles and Aldehyde Precursors

##### 2-*i*-Butyl-3,3-dimethyl-3*H*-indole (**20**)

The title compound was prepared according to the reported procedure.^54^ Phenylhydrazine (1.1 g; 10.2 mmol) and methyl-3-pentanone (1.13 g; 11.3 mmol) were dissolved in ethanol (10 mL). One drop of 35% HCl was added, and the solution was refluxed (heating mantle) for 2 h. The solvent was removed, and the crude mixture was added to acetic acid (10 mL). Zinc chloride (0.12 g; 0.09 mmol) was added, and the solution was refluxed for 2 h. After cooling the reaction mixture, a saturated solution of NaHCO_3_ was added, and the solution was extracted 2× with ethyl acetate (30 mL). Product was purified by silica gel chromatography (*n*-hexane/ethyl acetate, 10:1). Yellow oil. Yield: 1.2 g (68%). ^1^H NMR (300 MHz, *d*_4_-CD_3_OD) δ: 7.49 (m, 1H), 7.41–7.21 (m, 3H), 2.72–2.61 (m, 2H), 1.43–1.26 (m, 9H). The NMR data are consistent with those reported in the literature.^54^ 
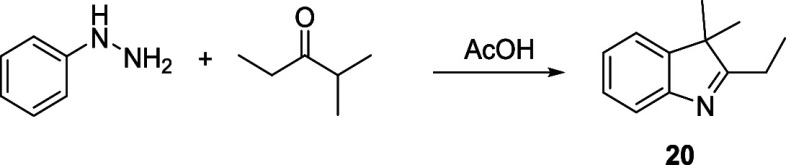


##### 2-Ethyl-1,3,3-trimethyl-3*H*-indol-1-ium Triflate (**21**)

The compound was prepared according to a known procedure.^54^ Indole **20** (0.5 g; 2.29 mmol) was dissolved in diethyl ether (15 mL). Methyl trifluoromethanesulfonate (0.71 g; 4.33 mmol) was added dropwise under stirring, and the reaction was stirred overnight under the N_2_ atmosphere at room temperature. The resulting precipitate was filtered off and washed several times with diethyl ether and *n*-pentane. White solid. Yield: 0.70 g (72%). ^1^H NMR (300 MHz, *d*_6_-DMSO) δ (ppm): 7.91 (m, 1H), 7.82 (s, 1H), 7.64 (s, 2H), 4.02 (s, 3H), 3.15 (m, 2H), 1.57 (s, 6H). The NMR data are consistent with those reported in the literature.^54^ 
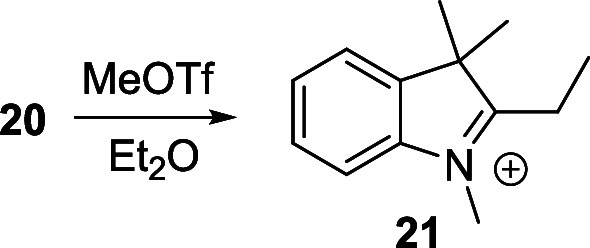


##### (*E*)-2-(1,3,3-Trimethylindolin-2-ylidene)­acetonitrile (**22**)

The title compound was prepared according to the reported procedure.^56^ Sodium thiocyanate (2.0 g; 25.4 mmol) was added to acetone (∼20 mL). Benzoyl chloride (3.5 g; 25.0 mmol) was added dropwise under vigorous stirring, a white precipitate was formed, and the reaction was refluxed (heating mantle) for 30 min. After cooling, 1,3,3-trimethyl-2-methyleneindoline (Fisher base; 4 g, 23.1 mmol) was added dropwise, and the reaction mixture turned deep red. After refluxing for 30 min and subsequent cooling, the reaction mixture was poured in water (∼250 mL) and 3× extracted with dichloromethane (∼30 mL). The solvent was removed under reduced pressure, and the crude product was dissolved in a 0.5 M sodium methoxide solution (200 mL) and refluxed for 2 h. After cooling, the solvent was removed under reduced pressure, and the product was purified using column chromatography (silica gel; *n*-hexane/ethyl acetate, 3:1). Red solid. Yield: 2.1 g (46%). ^1^H NMR (300 MHz, *d*_6_-DMSO) δ (ppm): 7.32 (m, 1H), 7.23 (m, 1H), 6.95 (m, 2H), 4.48 (s, 1H), 3.14 (s, 3H), 1.56 (s, 6H). The NMR data are consistent with those reported in the literature.^56^ 
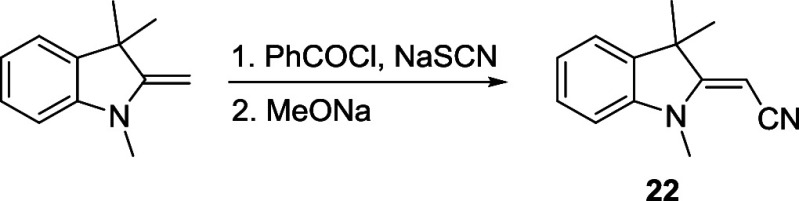


##### (*E*)-4-((*E*)-1,3,3-Trimethylindolin-2-ylidene)­but-2-enal (**23**)

The title compound was prepared using a modified reported procedure.^84^ 3-Dimethylaminoacrolein (2.8 g; 28.2 mmol) was added dropwise to acetic anhydride (12 mL) under stirring. After stirring for additional 20 min, a solution of 1,3,3-trimethyl-2-methyleneindoline (1 g, 5.8 mmol) in 5 mL of dry dichloromethane was added dropwise using a syringe pump over 2 h. The reaction was stirred overnight at room temperature. The reaction mixture was slowly added to a 10% sodium hydroxide solution (150 mL), and then, ethyl acetate (80 mL) was added, and the organic phase was extracted 2× with water. After the solvent was removed under reduced pressure, the product was purified by chromatography on silica gel (*n*-hexane/ethyl acetate, 3:1). Green solid. Yield: 0.56 g (43%). ^1^H NMR (300 MHz, *d*_6_-DMSO) δ (ppm): 9.45 (m, 1H), 7.85 (m, 1H), 7.36 (m, 1H), 7.23 (m, 1H), 6.97 (m, 2H), 5.84 (m, 1H), 5.67 (d, *J* = 12.7 Hz, 1H), 3.25 (s, 3H), 1.58 (s, 6H). The NMR data are consistent with those reported in the literature.^57^ 
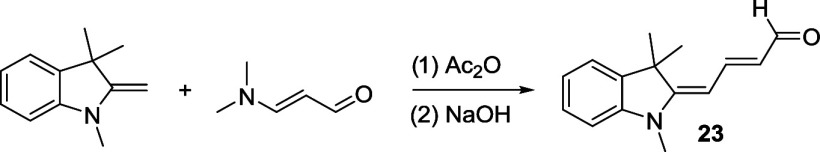


##### (2*E*,4*E*)-6-((*E*)-1,3,3-Trimethylindolin-2-ylidene)­hexa-2,4-dienal (**24**)

1,3,3-Trimethyl-2-methyleneindoline (0.6 mL; 3.46 mmol) was dissolved in acetone (250 mL). Under vigorous stirring, finely grinded pyrylium tetrafluoroborate^85^ (0.28 g; 1.67 mmol) was added. The reaction was stirred at room temperature for 4 h and sonicated every hour. After the solvent was removed, the crude material was dissolved in dichloromethane (30 mL) and washed with water 2×. Chromatography on silica gel (*n*-hexane/ethyl acetate, 4:1) afforded a red oil that was kept under high vacuum overnight. Red solid. Yield: 0.3 g (72%).^1^H NMR (500 MHz, *d*_6_-DMSO) δ (ppm): 9.41 (d, *J* = 10.0 Hz, 1H), 7.57–7.44 (m, 2H), 7.31 (m, 1H), 7.24–7.17 (m, 1H), 6.94–6.88 (m, 2H), 6.29–6.20 (m, 1H), 5.95–5.89 (m, 1H), 5.56 (d, *J* = 10.0 Hz, 1H), 3.20 (s, 3H), 1.56 (s, 6H). ^13^C­{^1^H} NMR (126 MHz *d*_6_-DMSO) δ (ppm): 192.9, 155.9, 142.3, 128.3, 125.1, 122.2, 121.9, 121.0, 107.8, 97.0, 46.3, 40.7, 39.7, 39.7, 39.5, 29.6, 28.3. (Figures S9–S10). HRMS (ESI^+^) *m*/*z*: calcd for C_17_H_19_NO [M + H]^+^ 254.1539, found 254.1538.
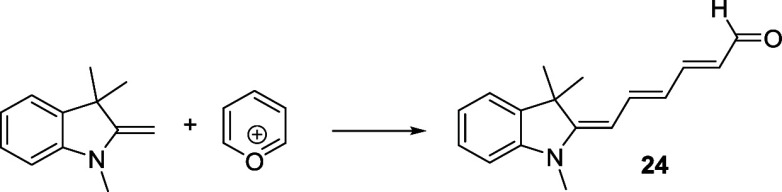


##### 2-Neopentylbenzo­[*d*]­thiazole (**25**)

The title compound was prepared according to the synthesis of 2-methylbenzothiazole.^86^ 2-Aminothiophenol (1.2 g; 9.6 mmol) and 2,2-dimethylbutanoic acid (1.36 g; 11.7 mmol) were dissolved in methanesulfonic acid (9 mL). After the addition of silica (2.6 g), the reaction was stirred at 140 °C (heating mantle) for 3 h. After cooling, the crude mixture was added dropwise to a saturated solution of NaHCO_3_ at 0 °C. The title product was extracted with ethyl acetate, and after the solvent was removed under reduced pressure, it was purified using column chromatography (silica gel; *n*-hexane/ethyl acetate, 10:1). Yellow solid. Yield: 0.3 g (20%). ^1^H NMR (300 MHz, *d*_6_-DMSO) δ (ppm): 8.01 (m, 2H), 7.44 (m, 2H), 2.99 (s, 2H), 1.03 (s, 9H). The NMR data are consistent with those reported in the literature.^87^ 
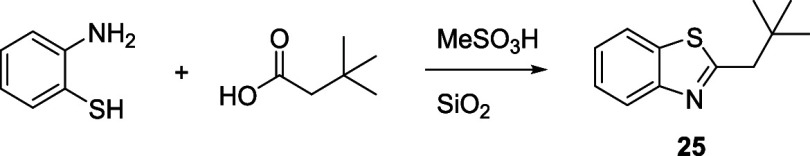


##### 2-*i*-Butylbenzo­[*d*]­thiazole (**26**)

The title compound was prepared by the procedure as in the synthesis of **24**. 2-Aminothiophenol (1.2 g; 9.6 mmol) and 3-methyl butyric acid (1.32 g; 11.7 mmol) were dissolved in methanesulfonic acid (9 mL). After the addition of silica (2.6 g), the reaction was stirred at 140 °C (heating mantle) for 3 h. After cooling, the crude mixture was added dropwise to a saturated solution of NaHCO_3_ at 0 °C. The mixture was extracted by ethyl acetate, the solvent was removed under reduced pressure, and the product was purified using column chromatography (silica gel; *n*-hexane/ethyl acetate, 10:1). Yellow oil. ^1^H Yield: 0.3 g (20%). ^1^H NMR (300 MHz, *d*_6_-DMSO) δ (ppm): 8.05 (m, 1H), 7.94 (m, 1H), 7.53–7.36 (m, 2H), 2.98 (d, *J* = 7.2 Hz, 2H), 2.17 (m, 1H), 0.98 (d, *J* = 6.6 Hz, 6H). The NMR data are consistent with those reported in the literature.^88^ 
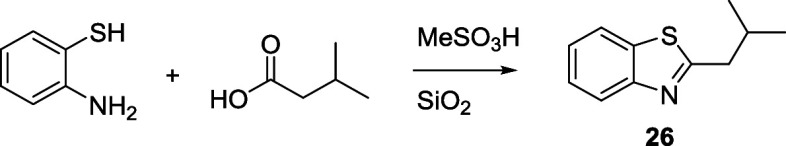


##### 2-Butylbenzo­[*d*]­thiazole (**27**)

The title compound was prepared according to the reported procedure.^89^ 2-Aminothiophenol (0.96 g; 7.7 mmol) was dissolved in toluene (10 mL). After 10 min, *n*-valeryl chloride (1 g; 8.3 mmol) was added dropwise under vigorous stirring. The reaction was stirred for 3 h at room temperature, and then ethyl acetate (30 mL) was added. The organic phase was washed 3× with water (∼50 mL). The solvent was removed under reduced pressure and the title product was purified using column chromatography (silica gel; *n*-hexane/ethyl acetate, 10:1). Yellow oil. Yield: 0.8 g (54%). ^1^H NMR (300 MHz, *d*_6_-DMSO) δ (ppm): 8.04 (d, *J* = 7.8 Hz, 1H), 7.93 (d, *J* = 7.8 Hz, 1H), 7.44 (m, 2H), 3.12 (m, 2H), 1.79 (m, 2H), 1.48–1.32 (m, 2H), 0.93 (t, *J* = 7.3 Hz, 3H). The NMR data are consistent with those reported in the literature.^89^ 
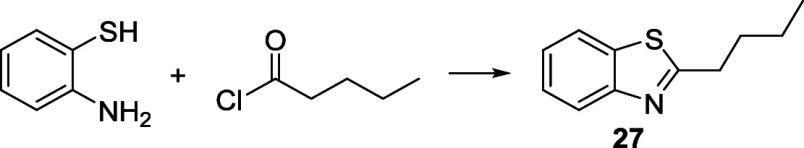


##### (*Z*)-2-(3-Methylbenzo­[*d*]­thiazol-2­(3*H*)-ylidene)­acetonitrile (**28**)

The title compound was prepared according to the reported procedure.^90^ 3-Methyl-2-methylthio-benzothazolium tosylate (0.8 g; 2.3 mmol) is dissolved in pyridine (6 mL). Cyanoacetic acid (0.23 g; 2.7 mmol) was added, followed by triethylamine (0.38 mL; 5.2 mmol). The reaction was stirred overnight at room temperature. The solvent was removed under reduced pressure, and water (∼30 mL) was added. The product was collected by filtration. Brown solid. Yield: 0.24 g (55%). ^1^H NMR (300 MHz, *d*_6_-DMSO) δ (ppm): 7.66 (m, 1H), 7.39–7.21 (m, 2H), 7.11 (s, 1H), 4.71 (s, 1H), 3.38 (s, 3H). The NMR data are consistent with those reported in the literature.^90^ 
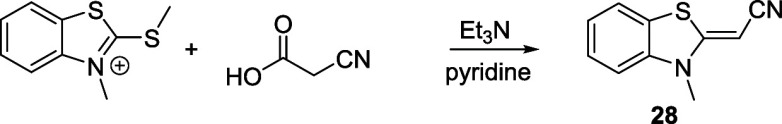


##### 2-Ethyl-3,3-dimethyl-3*H*-indole (**29**)

Phenylhydrazine (0.55 g; 5 mmol) and 2,5-dimethylhexan-3-one^91^ (0.78 g; 6.1 mmol) were dissolved in ethanol (10 mL). One drop of 35% HCl was added, and the solution was refluxed for 2 h. The solvent was removed, and the crude mixture was added to acetic acid (10 mL). ZnCl_2_ (0.12 g; 0.09 mmol) was added, and the solution was refluxed (heating mantle) for 2 h. After cooling the reaction mixture, a saturated solution of NaHCO_3_ was added, and the mixture was extracted 2× with ethyl acetate (30 mL). After the solvent was removed under reduced pressure, the product was purified by silica gel chromatography (*n*-hexane/ethyl acetate, 10:1). Yellow oil. Yield: 0.41 g (40%). ^1^H NMR (500 MHz *d*_6_-DMSO) δ (ppm): 7.46 (m, 1H), 7.41 (m, 1H), 7.27 (m, 1H), 7.17 (m, 1H), 2.40 (d, *J* = 6.5 Hz, 2H), 2.34 (m, 1H), 1.23 (s, 6H), 0.99 (d, *J* = 6.5 Hz, 6H). ^13^C­{^1^H} NMR (126 MHz, *d*_6_-DMSO) δ (ppm): 190.2, 154.1, 146.2, 127.7, 125.2, 121.9, 119.9, 53.8, 37.5, 25.7, 23.1, 22.9. (Figures S1 and S2). HRMS (ESI^+^) *m*/*z*: calcd for C_14_H_19_N [M + H]^+^ 202.1590; found 202.1588.
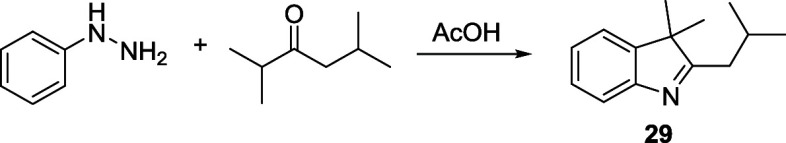


### Synthesis of Quaternary Salts

#### General Procedure for the Synthesis of Quaternary Salts

The corresponding heterocycle (1 equiv) was dissolved in diethyl ether (6 mL). Methyl trifluoromethanesulfonate (1.5 equiv) was added dropwise under vigorous stirring, and the reaction was stirred overnight under the N_2_ atmosphere at room temperature. The resulting precipitate was filtered off and washed several times with diethyl ether and *n*-pentane.

##### 3-Methyl-2-neopentylbenzo­[*d*]­thiazol-3-ium Triflate (**30**)

Prepared according to the general procedure above from **24** (0.3 g; 1.46 mmol) and methyl trifluoromethanesulfonate (0.34 g; 2.1 mmol). White solid. Yield: 0.3 g (55%). ^1^H NMR (500 MHz, *d*_6_-DMSO) δ (ppm): 8.45 (m, 1H), 8.29 (m, 1H), 7.93 (m, 1H), 7.84 (m, 1H), 4.32 (s, 3H), 3.57 (s, 2H), 1.13 (s, 9H). ^13^C­{^1^H} NMR (126 MHz, *d*_6_-DMSO) δ (ppm): 177.9, 142.4, 129.7, 129.4, 128.8, 124.6, 117.9, 42.3, 37.7, 34.8, 29.4. (Figures S3 and S4). HRMS (ESI^+^) *m*/*z*: calcd for C_13_H_18_NS^+^ [M–OTf^–^]^+^ 220.1154, found 220.1153.
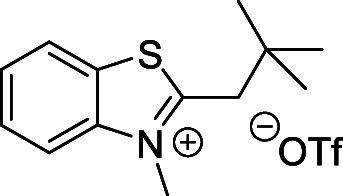


##### 2-*i*-Butyl-3-methylbenzo­[*d*]­thiazol-3-ium Triflate (**31**)

Prepared according to the general above procedure from **25** (0.3 g, 1.46 mmol) and methyl trifluoromethanesulfonate (0.34 g; 2.10 mmol). White solid. Yield: 0.4 g (72%). ^1^H NMR (500 MHz, *d*_6_-DMSO) δ (ppm): 8.45 (m, 1H), 8.31 (m, 1H), 7.92 (m, 1H), 7.83 (m, 1H), 4.27 (s, 3H), 3.46 (d, *J* = 7.1 Hz, 2H), 2.24 (m, 1H), 1.09 (d, *J* = 6.6 Hz, 6H). ^13^C­{^1^H} NMR (126 MHz, *d*_6_-DMSO) δ (ppm): 180.0, 142.3, 129.8, 129.2, 128.7, 124.8, 117.6, 38.4, 37.0, 29.0, 22.4. (Figures S5 and S6). HRMS (ESI^+^) *m*/*z* calcd for C_12_H_16_NS^+^ [M–OTf^–^]^+^ 206.0998, found 206.0998.
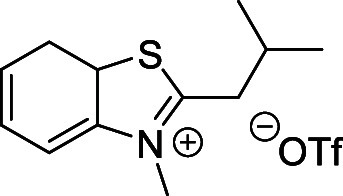


##### 2-Butyl-3-methylbenzo­[*d*]­thiazol-3-ium Triflate (**32**)

Prepared according to the general procedure above from **26** (0.8 g; 4.18 mmol) and methyl trifluoromethanesulfonate (1.02 g; 6.27 mmol). White solid. Yield: 1.21 g (81%). ^1^H NMR (300 MHz, DMSO) δ (ppm): 8.44 (d, *J* = 7.9 Hz, 1H), 8.31 (d, *J* = 7.9 Hz, 1H), 7.86 (m, 7.3 Hz, 2H), 3.49 (t, *J* = 7.3 Hz, 2H), 1.94–1.79 (m, 2H), 1.53 (m, 2H), 0.99 (t, *J* = 7.0 Hz, 3H). (Figure S6a). The compound was directly used in the next step. The NMR data are consistent with those reported in the literature.^92^ 
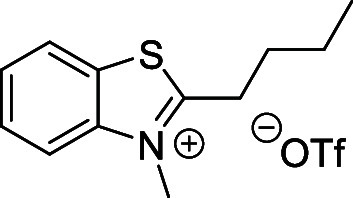


##### 2-*i*-Butyl-1,3,3-trimethyl-3*H*-indol-1-ium Triflate (**33**)

Prepared according to the general procedure above from **29** (0.41g; 2.0 mmol) and methyl trifluoromethanesulfonate (0.49 g; 3.0 mmol). Yellow solid. Yield: 0.63 g (86%). ^1^H NMR (500 MHz, *d*_6_-DMSO) δ (ppm): 7.94–7.89 (m, 1H), 7.85–7.81 (m, 1H), 7.67–7.63 (m, 2H), 4.08 (s, 3H), 3.09 (d, *J* = 7.5 Hz, 2H), 2.44–2.36 (m, 1H), 1.59 (s, 6H), 1.08 (d, *J* = 6.5 Hz, 6H). ^13^C­{^1^H} NMR (126 MHz, *d*_6_-DMSO) δ (ppm): 197.1, 142.6, 142.3, 130.1, 129.3, 123.5, 116.1, 55.2, 36.2, 35.7, 28.5, 23.3, 22.4. (Figures S7 and S8). HRMS (ESI^+^) *m*/*z* : calcd for C_15_H_22_N^+^ [M–OTf^–^]^+^ 216.1747, found 216.1744.
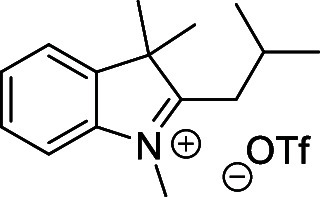


### Synthesis of Pentamethine Cyanines (Cy5)

#### General Procedure for the Synthesis of Pentamethine Cyanine Dyes from Quaternary Salts

An aldehyde (**22**; 1 equiv; 0.39 mM) was dissolved in acetonitrile (5 mL). After 10 min, POCl_3_ (44 μL; 0.47 mM, 1.2 equiv) was added dropwise under stirring. After 10 min, the corresponding quaternary salt (**20, 30**–**33**; 1.1–3 equiv) was added to the solution. A solution of *N*,*N*-diisopropylethylamine (DIPEA; 0.241 mL; 1.38 mM, 3.5 equiv) in acetonitrile (3 mL) was added dropwise over 20 min using a syringe pump. The solvent was removed, and a crude material was dissolved in dichloromethane (∼20 mL) and extracted several times with an HCl aqueous solution at pH = 4. The solvent was evaporated under reduced pressure, and the pure product was obtained by chromatography on silica gel (*n*-hexane/ethyl acetate, 1:1, then dichloromethane/methanol, 100:0 to 96:4). The product was obtained as a dark solid.
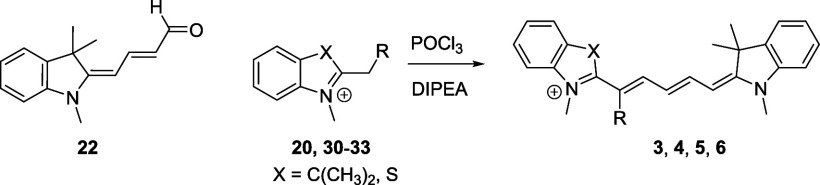


##### 1,3,3-Trimethyl-2-((1*E*,3*E*,5*Z*)-5-(3-methylbenzo­[*d*]­thiazol-2­(3*H*)-ylidene)­penta-1,3-dien-1-yl)-3*H*-indol-1-ium Triflate (**2**)

Prepared according to the general procedure above from **22** (0.060 g; 0.26 mmol) and 2,3-dimethylbenzo­[*d*]­thiazol-3-ium trifluoromethanesulfonate^93^ (0.18g; 0.57 mmol). Blue solid. Yield: 61 mg (41%). ^1^H NMR (500 MHz, *d*_6_-DMSO) δ (ppm): 8.14–8.03 (m, 3H), 7.86 (d, *J* = 8.2 Hz, 1H), 7.66 (t, *J* = 8.2 Hz, 1H), 7.51 (d, *J* = 4.8 Hz, 2H), 7.35 (m, 1H), 7.24 (d, *J* = 8.2 Hz, 1H), 7.14 (t, *J* = 7.3 Hz, 1H), 6.75 (d, *J* = 13.7 Hz, 1H), 6.50 (m, 1H), 6.06 (d, *J* = 13.3 Hz, 1H), 3.95 (s, 3H), 3.49 (s, 3H), 1.65 (s, 6H).(Figures S10b)­
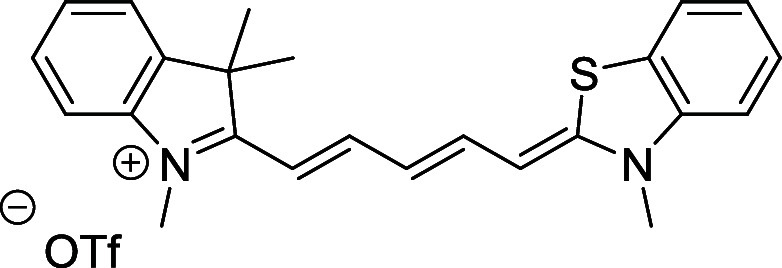


##### 1,3,3-Trimethyl-2-((2*E*,4*E*)-6-((*E*)-1,3,3-trimethylindolin-2-ylidene)­hexa-2,4-dien-2-yl)-3*H*-indol-1-ium Triflate (**3**)

Prepared according to the general procedure above from **22** (0.085 g; 0.37 mmol) and **20** (0.21g; 0.62 mmol). Blue solid. Yield: 0.1 g (49%). ^1^H NMR (500 MHz, *d*_6_-DMSO) δ (ppm): 8.13 (m, 1H), 7.91 (m, 1H), 7.63 (m, 1H), 7.55 (m, 1H), 7.46 (m, 2H), 7.39–7.32 (m, 2H), 7.29 (d, *J* = 10.0 Hz, 1H), 7.18 (m, 1H), 6.60 (m, 1H), 6.24 (d, *J* = 10.0 Hz, 1H), 3.85 (s, 3H), 3.53 (s, 3H), 2.23 (s, 3H), 1.67 (d, *J* = 12.0 Hz, 12H). ^13^C­{^1^H} NMR (126 MHz, *d*_6_-DMSO) δ (ppm): 180.0, 171.2, 153.5, 152.0, 144.0, 143.5, 141.8, 140.9, 128.9, 128.7, 126.2, 124.2, 122.6, 122.4, 121.1, 119.9, 112.5, 111.6, 110.6, 102.9, 51.6, 48.6, 37.9, 31.1, 27.6, 26.9, 15.5. (Figures S11–S13). HRMS (ESI^+^) *m*/*z* calcd for C_28_H_33_N_2_^+^ [M–OTf^–^]^+^ 397.2638, found 397.2638 (Figure S62).
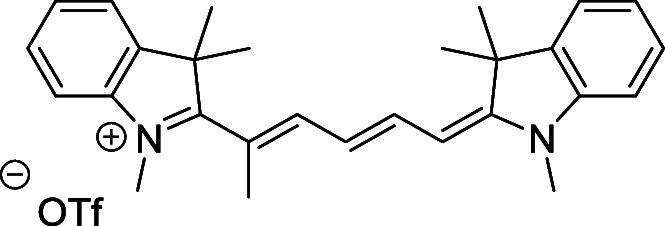


##### 3-Methyl-2-((4*E*,6*E*)-8-((*E*)-1,3,3-trimethylindolin-2-ylidene)­octa-4,6-dien-4-yl)­benzo­[*d*]­thiazol-3-ium Triflate (**4**)

Prepared according to the general procedure from **22** (0.085g; 0.37 mmol) and **32** (0.21g; 0.59 mmol). Blue solid. Yield: 170 mg (82%). ^1^H NMR (500 MHz, *d*_6_-DMSO) δ (ppm): 8.24 (d, *J* = 7.6 Hz, 1H), 8.08 (d, *J* = 7.6 Hz, 1H), 7.81 (d, *J* = 8.5 Hz, 2H), 7.66 (s, 2H), 7.42 (d, *J* = 6.6 Hz, 1H), 7.28 (t, *J* = 7.2 Hz, 1H), 7.14–7.00 (m, 2H), 6.52 (m, 1H), 5.97 (d, *J* = 12.3 Hz, 1H), 4.20 (s, 3H), 3.37 (s, 2H), 2.70 (s, 2H), 1.61 (s, 8H), 1.01 (m, 3H). ^13^C­{^1^H} NMR (126 MHz, *d*_6_-DMSO) δ (ppm): 173.2, 166.5, 148.6, 148.0, 147.9, 144.2, 143.7, 140.0, 129.4, 128.4, 127.3, 125.0, 124.0, 122.4, 119.9, 119.5, 117.3, 116.9, 116.2, 109.0, 99.7, 47.3, 34.2, 30.2, 28.1, 22.4, 14.2. (Figures S14–S17). HRMS (ESI^+^) *m*/*z* calcd for C_27_H_31_N_2_S^+^ [M–OTf^–^]^+^ 415.2002, found 415.2001 (Figure S63).
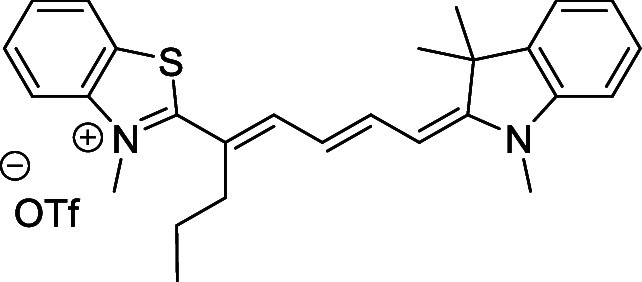


##### 1,3,3-Trimethyl-2-((3*E*,5*E*)-2-methyl-7-((*E*)-1,3,3-trimethylindolin-2-ylidene)­hepta-3,5-dien-3-yl)-3*H*-indol-1-ium Triflate (**5**)

Prepared according to the general procedure above from **22** (0.07g; 0.30 mmol) and **33** (0.20g; 0.54 mmol). Blue solid. Yield: 72 mg (40%). The compound was obtained as 6:1 mixture of *E* and *Z* isomers. ^1^H NMR (500 MHz, *d*_6_–DMSO) δ (ppm): 7.83 (t, *J* = 7.4 Hz, 2H), 7.63 (m, 2H), 7.49 (t, *J* = 12.8 Hz, 1H), 7.34 (d, *J* = 7.2 Hz, 1H), 7.25–7.18 (m, 2H), 6.92 (t, *J* = 7.2 Hz, 2H), 5.97 (t, *J* = 12.2 Hz, 1H), 5.69 (d, *J* = 12.4 Hz, 1H), 3.86 (s, 3H), 3.20 (s, 3H), 2.94 (dt, *J* = 13.7, 6.8 Hz, 1H), 1.63 (s, 6H), 1.55 (s, 6H), 1.30 (d, *J* = 6.7 Hz, 6H). ^13^C­{^1^H} NMR (126 MHz, *d*_6_–DMSO) δ (ppm): 151.0, 144.6, 142.6, 139.5, 129.2, 128.3, 127.9, 124.8, 123.2, 122.2, 121.6, 121.3, 117.7, 115.8, 108.0, 105.0, 103.4, 98.2, 54.7, 46.5, 37.3, 30.3, 29.9, 29.0, 28.6, 28.2, 26.0, 25.8, 24.4, 23.2. (Figures S18–S21). HRMS (ESI^+^) *m*/*z*: calcd for C_30_H_37_N_2_^+^ [M–OTf^–^]^+^ 425.2951, found 425.2954 (Figure S64).
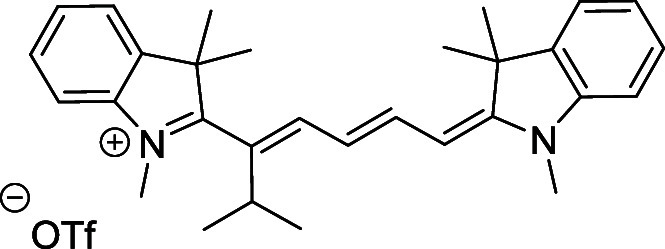


##### 3-Methyl-2-((3*E*,5*E*)-2-methyl-7-((*E*)-1,3,3-trimethylindolin-2-ylidene)­hepta-3,5-dien-3-yl)­benzo­[*d*]­thiazol-3-ium Triflate (**6**)

Prepared according to the general procedure above from **22** (0.060 g; 0.26 mmol) and **31** (0.15 g; 0.42 mmol). Blue solid. Yield: 80 mg (55%). ^1^H NMR (500 MHz, *d*_4_–CD_3_OD) δ (ppm): 8.14 (m, 2H), 7.77 (m, 2H), 7.36 (s, 1H), 7.16–7.06 (m, 2H), 6.98 (m, 1H), 6.85–6.66 (m, 2H), 5.50 (m, 2H), 4.11 (s, 3H), 2.95 (m, 4H), 1.49 (s, 6H), 1.23 (s, 6H). ^13^C NMR gave an inconsistent result due to the aggregation of the compound (Figures S22–S25). HRMS (ESI^+^) *m*/*z*: calcd for C_27_H_31_N_2_S^+^ [M–OTf^–^]^+^ 415.2202, found 415.2205 (Figure S65).
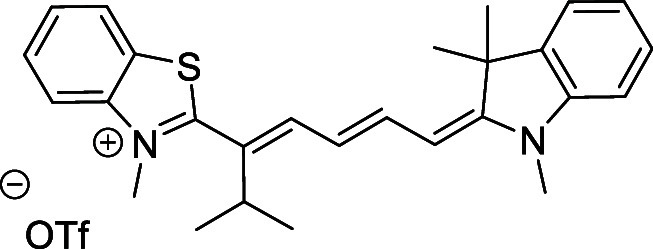


#### General Procedure for the Synthesis of Pentamethine Cyanine Dyes from Fisher’s Bases

Aldehyde **22** (1 equiv; 0.39 mM) was dissolved in acetonitrile (5 mL). After 10 min, POCl_3_ (44 μL; 0.47 mM, 1.2 equiv) was added dropwise under stirring. After 10 min, a heterocycle **27**–**30** (1–3 equiv) was added to the solution. The reaction mixture was stirred under reflux (heating mantle) until completion (monitored by HPLC). The solvent was removed under reduced pressure, and the crude product was dissolved in dichloromethane (∼20 mL) and extracted several times with an HCl aqueous solution (pH = 4). The solvent was removed and the pure compound was obtained using chromatography on silica gel (*n*-hexane/ethyl acetate, 1:1, then dichloromethane/methanol, 100:0 to 96:4). The product was obtained as a dark solid.
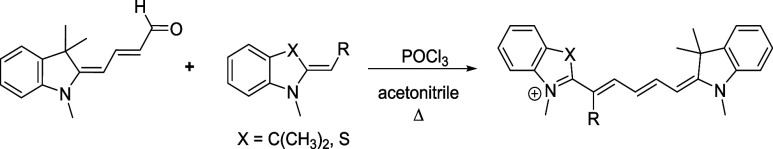


##### 2-((1*Z*,3*E*)-1-Cyano-5-((*E*)-1,3,3-trimethylindolin-2-ylidene)­penta-1,3-dien-1-yl)-1,3,3-trimethyl-3*H*-indol-1-ium chloride (**7**)

Prepared according to the general procedure above from **22** (0.1 g; 0.44 mmol) and **21** (0.11 g; 0.55 mmol). Blue solid. Yield: 0.1 g (51%). ^1^H NMR (500 MHz, *d*_4_–CD_3_OD) δ (ppm): 8.37 (m, 1H), 8.07 (d, *J* = 7.9 Hz, 1H), 7.55 (m, 1H), 7.51 (d, *J* = 7.9 Hz, 1H), 7.45 (t, *J* = 7.5 Hz, 1H), 7.38 (m, 2H), 7.32 (m, 1H), 7.17 (m, 2H), 6.83–6.71 (m, 2H), 3.81 (d, *J* = 6.9 Hz, 6H), 1.67 (d, *J* = 18.8 Hz, 12H). ^13^C­{^1^H} NMR (126 MHz, *d*_4_–CD_3_OD) δ (ppm): 179.9, 174.0, 156.2, 147.8, 143.5, 142.6, 142.1, 139.7, 128.8, 128.3, 127.7, 125.0, 123.3, 122.2, 121.8, 117.4, 112.9, 110.3, 109.5, 80.4, 51.1, 50.4, 34.4, 31.8, 25.5, 24.6. (Figures S26–S29). HRMS (ESI^+^) *m*/*z*: calcd for C_28_H_30_N_3_^+^ [M–OTf^–^]^+^ 408.2434, found 408.2433 (Figure S66).
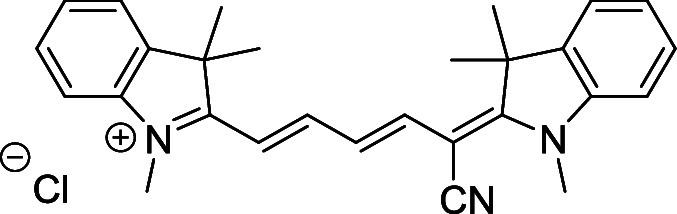


##### 2-((1*E*,3*E*)-1-cyano-5-((*E*)-1,3,3-trimethylindolin-2-ylidene)­penta-1,3-dien-1-yl)-3-methylbenzo­[*d*]­thiazol-3-ium chloride (**8**)

Prepared according to the general procedure above from **22** (0.075g; 0.33 mmol) and **27** (0.075g; 0.40 mmol). The product was isolated by precipitation after the addition of dichloromethane (5 mL) to the reaction mixture. Blue solid. Yield: 130 mg (90%). ^1^H NMR (500 MHz, *d*_4_–CD_3_OD) δ (ppm): 8.28 (m, 1H), 7.88 (m, 1H), 7.80 (m, 1H), 7.63 (m, 1H), 7.57–7.50 (m, 2H), 7.43–7.37 (m, 3H), 7.36–7.31 (m, 1H), 6.75–6.69 (m, 1H), 6.58 (m, 1H), 4.11 (s, 3H), 3.72 (s, 3H), 1.67 (s, 6H). ^13^C­{^1^H} NMR (126 MHz, *d*_4_–CD_3_OD) δ (ppm): 178.5, 166.6, 155.9, 155.8, 142.9, 142.3, 141.1, 128.7, 128.2, 126.9, 125.6, 125.3, 122.2, 121.5, 116.2, 113.5, 112.3, 107.1, 77.8, 50.5, 50.5, 35.4, 31.3, 25.8. (Figures S30–S33). HRMS (ESI^+^) *m*/*z*: calcd for C_25_H_24_N_3_S^+^ [M–OTf ^–^]^+^ 398.1685, found 398.1683 (Figure S67).
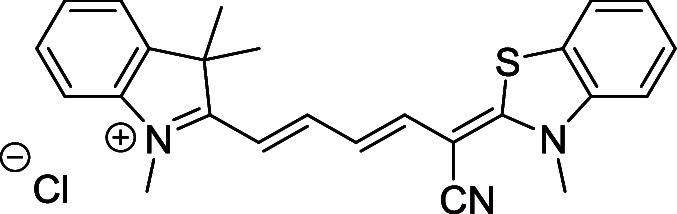


##### 2-((1*Z*,3*E*)-1,5-Dicyano-5-((*Z*)-1,3,3-trimethylindolin-2-ylidene)­penta-1,3-dien-1-yl)-1,3,3-trimethyl-3*H*-indol-1-ium chloride (**9**)

**21** (0.1 g, 0.50 mmol) and malonaldehyde dianilide hydrochloride (0.065 g, 0.25 mmol) were added to freshly distilled acetic anhydride (0.5 mL). The reaction mixture was heated to 95–100 °C (heating mantle) for approximately 30 min (monitored using a UV–vis spectrometer). After cooling, methanol (2 mL) was added, and then diethyl ether (∼15 mL) was added dropwise. Then the reaction mixture was kept at–20 °C overnight. A precipitate formed, was filtered off, and washed 3× with diethyl ether (∼ 5 mL) and *n*-pentane (∼ 5 mL). Blue solid. Yield: 107 mg (91%). ^1^H NMR (500 MHz, *d*_4_-CD_3_OD) δ (ppm): 8.62 (d, *J* = 12.6 Hz, 2H), 7.68–7.47 (m, 8H), 7.16 (t, *J* = 12.6 Hz, 1H), 4.14 (s, 6H), 1.82 (s, 12H). ^13^C­{^1^H} NMR (126 MHz, *d*_4_-CD_3_OD) δ (ppm): 178.3, 158.2, 142.8, 141.1, 128.8, 127.5, 122.1, 118.8, 115.4, 112.6, 86.0, 65.5, 52.3, 35.5, 24.0. (Figures S34 and S35). HRMS (ESI^+^) *m*/*z*: calcd for C_29_H_29_N_4_^+^ [M–OTf^–^]^+^ 433.2387, found 433.2388 (Figure S68).
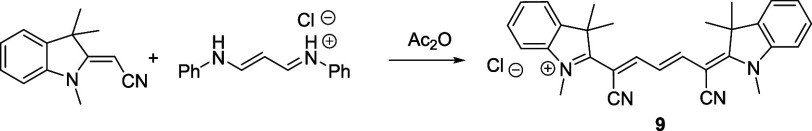


### Synthesis of Heptamethine Cyanines (Cy7)

#### General Procedure for the Synthesis of Heptamethine Cyanine Dyes from Quaternary Salts

Aldehyde **23** (1 equiv; 0.39 mM) was dissolved in acetonitrile (5 mL). After 10 min, POCl_3_ (44 μL; 0.47 mM, 1.2 equiv) was added dropwise under stirring. After 10 min, a heterocycle (**20**, **31**–**33**) (1.1–3 equiv) was added to the solution. Then, a solution of *N*,*N*-diisopropylethylamine (0.241 mL; 1.38 mM, 3.5 equiv) in acetonitrile (3 mL) was added dropwise over 20 min using a syringe pump. The solvent was removed, and the crude material was dissolved in dichloromethane (∼20 mL) and extracted several times with an HCl aqueous solution (pH = 4). The solvent was removed under reduced pressure and pure compound was obtained by chromatography on silica gel (*n*-hexane/ethyl acetate, 1:1, then dichloromethane/methanol, 100:0 to 96:4). The product was obtained as a dark solid.
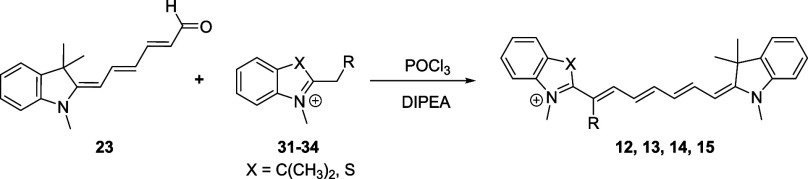


##### 3-Methyl-2-((1*E*,3*E*,5*E*)-7-((*E*)-1,3,3-trimethylindolin-2-ylidene)­hepta-1,3,5-trien-1-yl)-3l4-benzo­[*d*]­thiazole (**11**)

Prepared according to the general procedure shown above from **23** (50 mg; 0.19 mmol) and 2,3-dimethylbenzo­[*d*]­thiazol-3-ium trifluoromethanesulfonate^93^ (185 mg; 0.59 mmol). Green solid. Yield: 27 mg (25%). ^1^H NMR (500 MHz, *d*_4_-CD_3_OD) δ (ppm): 7.85 (m, 1H), 7.72–7.55 (m, 4H), 7.44 (t, *J* = 7.6 Hz, 1H), 7.27 (m, 2H), 7.19 (t, *J* = 7.7 Hz, 1H), 6.99–6.93 (m, 2H), 6.67 (d, *J* = 13.8 Hz, 1H), 6.46 (t, *J* = 12.5 Hz, 1H), 6.32 (m, 1H), 5.82 (d, *J* = 13.0 Hz, 1H), 3.89 (s, 3H), 3.31 (s, 3H), 1.54 (s, 6H). ^13^C­{^1^H} NMR (126 MHz, *d*_4_-MeOH) δ (ppm): 167.4, 153.5, 151.0, 146.5, 143.7, 142.1, 139.9, 128.4, 127.9, 126.3, 124.4, 122.5, 121.6, 119.1, 113.9, 108.4, 105.4, 99.9, 33.2, 29.1, 26.9. (Figures S36–S39). HRMS (ESI^+^) *m*/*z*: calcd for C_26_H_27_N_2_S^+^ [M–OTf^–^]^+^ 399.1889, found 399.1890 (Figure S69).
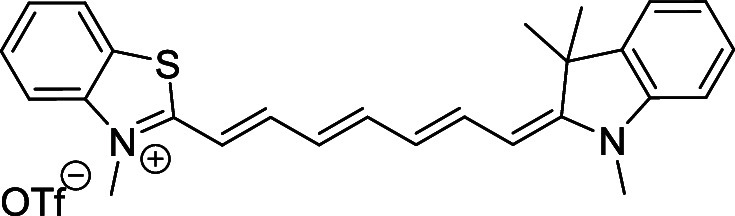


##### 1,3,3-Trimethyl-2-((2*E*,4*E*,6*E*)-8-((*E*)-1,3,3-trimethylindolin-2-ylidene)­octa-2,4,6-trien-2-yl)-3*H*-indol-1-ium Triflate (**12**)

Prepared according to the general procedure above from **23** (85 mg; 0.36 mmol) and **20** (190 mg; 0.56 mmol). Green solid. Yield: 143 mg (75%). ^1^H NMR (500 MHz, *d*_6_-DMSO) δ (ppm): 7.68–7.58 (m, 2H), 7.50 (m, 5H), 7.41 (m, 1H), 7.31 (m, 1H), 7.16 (m 1H), 7.08 (m, 1H), 6.56 (m, 1H), 6.46 (m, 1H), 5.97 (m, 1H), 3.86 (s, 3H), 3.42 (s, 3H), 2.24 (s, 3H), 1.62 (d, *J* = 26.9 Hz, 12H). ^13^C­{^1^H} NMR (126 MHz, *d*_6_-DMSO) δ (ppm): 150.1, 144.0, 143.8, 142.0, 140.4, 129.0, 128.6, 127.7, 127.1, 125.6, 123.1, 122.8, 122.5, 121.4, 119.9, 117.5, 113.4, 109.7, 104.5, 101.3, 87.6, 65.4, 52.3, 47.8, 37.8, 30.7, 27.9, 26.3, 15.6. (Figures S40–S43). HRMS (ESI^+^) *m*/*z*: calcd for C_30_H_35_N_2_^+^ [M–OTf^–^]^+^ 423.2795, found 423.2796 (Figure S70).
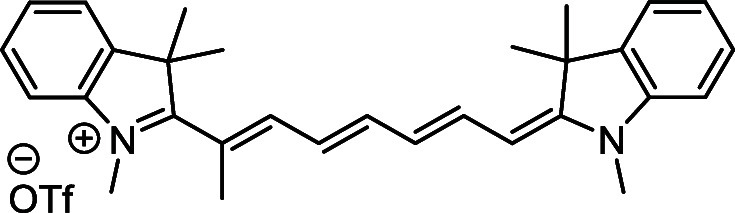


##### 3-Methyl-2-((4*E*,6*E*,8*E*)-10-((*E*)-1,3,3-trimethylindolin-2-ylidene)­deca-4,6,8-trien-4-yl)­benzo­[*d*]­thiazol-3-ium Triflate (**13**)

Prepared according to the general procedure above from **23** (80 mg; 0.31 mmol) and **32** (130 mg; 0.36 mmol). Green solid. Yield: 50 mg (26%). ^1^H NMR (500 MHz, *d*_3_-CD_3_CN) δ (ppm): 8.15 (m, 1H), 7.98 (m, 1H), 7.87 (m, 1H), 7.75 (m, 1H), 7.37 (s, 1H), 7.26 (m, 2H), 7.13 (d, *J* = 7.0 Hz, 2H), 6.96 (t, *J* = 7.0 Hz, 1H), 6.86 (m, 1H), 6.56 (s, 1H), 6.33 (s, 1H), 5.64 (d, *J* = 13.4 Hz, 1H), 4.16 (s, 3H), 3.25 (s, 3H), 2.76 (s, 2H), 1.65 (m, 2H), 1.59 (s, 6H), 1.03 (m, 3H). ^13^C­{^1^H} NMR (126 MHz, *d*_3_-CD_3_CN) δ (ppm): 175.9, 150.0, 145.2, 143.8, 139.9, 135.7, 130.2, 130.1, 128.6, 128.5, 128.5, 125.6, 124.9, 124.6, 124.0, 123.1, 122.2, 121.4, 121.0, 120.5, 116.8, 107.9, 98.1, 46.9, 39.6, 29.9, 28.0, 22.9, 13.6. (Figures S44 and S45). HRMS (ESI^+^) *m*/*z*: calcd for C_29_H_33_N_2_S^+^ [M–OTf^–^]^+^ 441.2359, found 441.2361 (Figure S71).
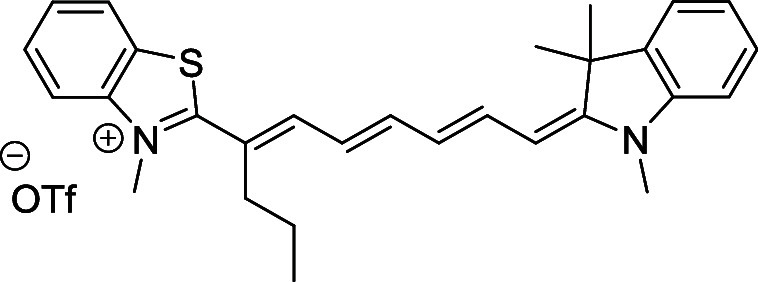


##### 3-Methyl-2-((3*E*,5*E*,7*E*)-2-methyl-9-((*E*)-1,3,3-trimethylindolin-2-ylidene)­nona-3,5,7-trien-3-yl)­benzo­[*d*]­thiazol-3-ium Triflate (**14**)

Prepared according to the general procedure above from **23** (100 mg; 0.39 mmol) and **31** (320 mg; 0.70 mmol). Dark solid. Yield: 150 mg (64%). ^1^H NMR (500 MHz, *d*_4_-CD_3_OD) δ (ppm): 8.20 (m, 1H), 8.12 (m, 1H), 7.86 (m, 1H), 7.75 (m, 1H), 7.06 (m, 3H), 6.83 (m, 2H), 6.76 (m, 1H), 6.65 (d, *J* = 7.8 Hz, 1H), 6.02 (s, 2H), 5.34 (d, *J* = 10.9 Hz, 1H), 4.11 (s, 3H), 3.08 (m, 4H), 1.45 (s, 6H), 1.20 (d, *J* = 6.9 Hz, 6H). ^13^C­{^1^H} NMR (126 MHz, *d*_4_-CD_3_OD) δ (ppm): 175.4, 144.7, 141.9, 138.8, 130.3, 129.7, 129.2, 128.6, 127.6, 126.3, 125.6, 123.6, 122.5, 121.7, 121.1, 120.6, 120.0, 119.1, 117.0, 115.3, 106.3, 41.8, 37.3, 27.9, 27.2, 23.8, 22.2, 21.7, 20.9.(Figures S46–S49). HRMS (ESI^+^) *m*/*z*: calcd for C_29_H_33_N_2_S^+^ [M–OTf^–^]^+^ 441.2359, found 441.2359 (Figure S72).
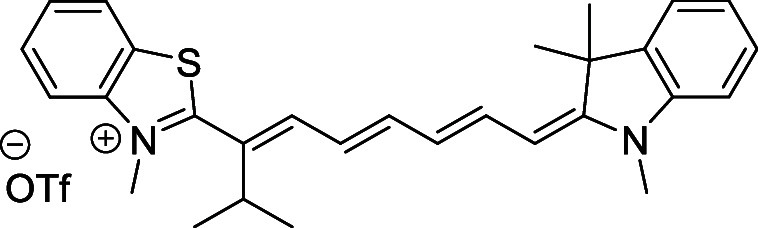


##### 2-((3*E*,5*E*,7*E*)-2,2-Dimethyl-9-((*E*)-1,3,3-trimethylindolin-2-ylidene)­nona-3,5,7-trien-3-yl)-3-methylbenzo­[*d*]­thiazol-3-ium Triflate (**15**)

Prepared according to the general procedure above from **23** (100 mg; 0.39 mmol) and **30** (260 mg; 0.70 mmol). Dark solid. Yield: 60 mg (25%). ^1^H NMR (500 MHz, *d*_4_-CD_3_OD) δ (ppm): 8.27 (m, 1H), 8.19 (m, 1H), 7.90 (m, 1H), 7.82 (m, 1H), 7.08–7.03 (m, 2H), 7.02–6.93 (m, 2H), 6.74 (m, 2H), 6.60 (d, *J* = 7.8 Hz, 1H), 5.84 (m, 1H), 5.35–5.22 (m, 2H), 4.13 (s, 3H), 2.98 (s, 3H), 1.44 (s, 6H), 1.22 (s, 9H). ^13^C­{^1^H} NMR (126 MHz, *d*_4_-CD_3_OD) δ (ppm): 175.0, 159.5, 144.7, 144.2, 138.7, 138.1, 136.0, 130.1, 129.9, 129.0, 128.3, 127.5, 123.8, 123.4, 121.7, 121.1, 119.7, 119.6, 117.4, 106.0, 96.1, 45.5, 36.7, 28.9, 28.2, 27.9, 27.2. (Figures S50–S53). HRMS (ESI^+^) *m*/*z* calcd for C_30_H_35_N_2_S^+^ [M–OTf^–^]^+^ 455.2515, found 455.2519 (Figure S73).
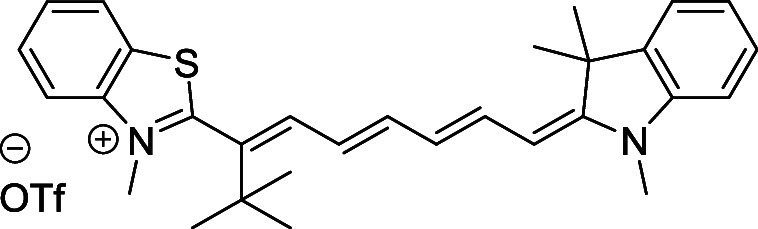


##### 2-((1*Z*,3*E*,5*E*)-1-Cyano-7-((*E*)-1,3,3-trimethylindolin-2-ylidene)­hepta-1,3,5-trien-1-yl)-1,3,3-trimethyl-3*H*-indol-1-ium chloride (**16**)

In a dry Schlenk flask, **21** (0.11g; 0.55 mmol) was added to dry tetrahydrofuran (1.6 mL). *n*-Butyllithium (120 μL of a 6 M *n*-hexane solution; 0.72 mmol) was added dropwise at −78 °C. After 15 min, a yellow precipitate appeared. **23** (50 mg; 0.2 mmol) was dissolved in dry tetrahydrofuran (0.4 mL) and the mixture was added dropwise. The reaction was stirred at −78 °C for 20 min and then at room temperature for 1 h. Dichloromethane was added (∼10 mL), and then water (3 mL) was added dropwise to quench the reaction. An organic phase was washed 2× with an HCl aqueous solution (pH = 3). The product was purified by chromatography on silica (*n*-hexane/ethyl acetate, 1:1, then dichloromethane/methanol, 100:0 to 96:4). Green solid. Yield: 60 mg (65%). ^1^H NMR (500 MHz, *d*_6_-DMSO) δ (ppm): 8.16 (m, 1H), 7.81–7.72 (m, 3H), 7.64–7.55 (m, 2H), 7.52 (t, *J* = 7.5 Hz, 2H), 7.37 (t, *J* = 7.7 Hz, 1H), 7.26 (d, *J* = 7.5 Hz, 1H), 7.19 (t, *J* = 7.4 Hz, 1H), 6.95 (d, *J* = 15.0 Hz, 1H), 6.81 (m, 1H), 6.55 (m, 1H), 3.91 (s, 3H), 3.77 (s, 3H), 1.68 (d, *J* = 24.5 Hz, 12H). ^13^C­{^1^H} NMR (126 MHz, *d*_6_-DMSO) δ (ppm): 179.7, 171.0, 155.3, 153.1, 144.2, 143.2, 142.5, 140.8, 139.5, 129.3, 129.3, 128.7, 128.5, 124.5, 118.6, 114.5, 112.6, 110.5, 79.5, 51.4, 49.8, 35.1, 33.6, 26.3, 26.0. (Figures S54–S57). HRMS (ESI^+^) *m*/*z*: calcd for C_30_H_32_N_3_^+^ [M–OTf^–^]^+^ 434.2591, found 434.2594 (Figure S74).
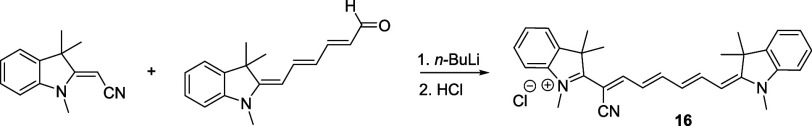


##### 2-((1*Z*,3*E*,5*E*)-1,7-dicyano-7-((*Z*)-1,3,3-trimethylindolin-2-ylidene)­hepta-1,3,5-trien-1-yl)-1,3,3-trimethyl-3*H*-indol-1-ium Chloride (**17**)

**21** (0.1 g, 0.50 mmol) and glutaconaldehydedianil hydrochloride (0.072 g, 0.25 mmol) were added to fresh distilled acetic anhydride (0.5 mL). The reaction was heated to 95–100 °C (heating mantle) for approximately 1 h (monitored by a UV–vis spectrometer). After cooling, methanol (2 mL) was added, and then diethyl ether (∼15 mL) was added dropwise. The flask was left at −20 °C overnight. The precipitate was filtered and washed 3× times with diethyl ether (∼5 mL) and *n*-pentane (∼5 mL). Blue solid. Yield: 82 mg (65%). ^1^H NMR (500 MHz, *d*_3_-CD_3_CN) δ (ppm): 8.25–8.08 (m, 3H), 7.57 (d, *J* = 7.5 Hz, 2H), 7.53 (t, *J* = 7.5 Hz, 2H), 7.48–7.42 (m, 4H), 6.97 (s, 2H), 3.99 (s, 6H), 1.75 (s, 12H).^13^C­{^1^H} NMR (126 MHz, *d*_3_-CD_3_CN) δ (ppm): 177.5, 163.2, 156.1, 143.7, 141.5, 129.5, 127.7, 124.0, 122.9, 116.5, 113.1, 86.3, 52.5, 36.9, 25.2. (Figures S58–S61). HRMS (ESI^+^) *m*/*z*: calcd for C_31_H_31_N_4_^+^ [M–OTf^–^]^+^ 459.2543, found 459.2546 (Figure S75).
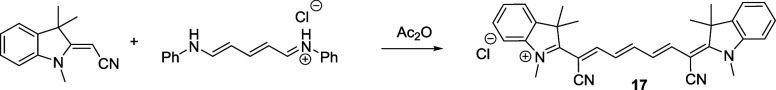


## Supplementary Material



## Data Availability

The data underlying this study are available in the published article and its Supporting Information.

## Notes

Materials and methods, ^1^H and ^13^C­{1H} NMR spectra, MS data, Absorption and emission spectra, Quantum-chemical calculations (PDF)
