# Supplementary material for: Engineering the Photophysics of Cyanines by Chain C1′ Substituents
Source: J Org Chem. 2025 Dec 8;90(50):17797–813. doi: 10.1021/acs.joc.5c02283 (PMC12723678; doi:10.1021/acs.joc.5c02283)
Supplement: Supplementary file 1 [file jo5c02283_si_001.pdf]

Supporting Information

Engineering the Photophysics of Cyanines by Chain C1´ Substituents

Ottavio Bedocchi,[†,‡,$] Jan Polena,[¶,$] Jana Okoročenkova,[†,‡]
Petr Slavíček,*[c] Petr Klán[†,‡,*]

[†] *Department of Chemistry, Faculty of Science, Masaryk University, 62500 Brno, Czech Republic.*

[‡] *RECETOX, Faculty of Science, Masaryk University, 62500 Brno, Czech Republic.*

[¶] *Department of Physical Chemistry, University of Chemistry and Technology, 16628 Prague 6, Czech Republic.*

[$] Those authors contributed equally to this work.

* Petr Slavíček: Petr.Slavicek@vscht.cz; Petr Klán: klan@sci.muni.cz

Contents

Materials and Methods S2
NMR Spectra S4
MS Data S67
Absorption and Emission Spectra S72
Quantum-Chemical Calculations S77
Calculated Absorption Spectra S86
References S100

Materials and Methods

Reagents and solvents of the highest purity available were used as purchased unless stated otherwise. Compounds **1**, **18**, and **19** were purchased from TCI chemicals and used without further purification. The synthetic procedures were performed under an ambient atmosphere unless stated otherwise. Column chromatography was performed using silica gel 60. ^{1}H NMR spectra were recorded on 300 or 500 MHz spectrometers and ^{13}C NMR were obtained on 125 MHz or 75 MHz instruments in $CDCl_3$, CD_3OD, or $(CD_3)_2SO$. ^{1}H chemical shifts are reported in ppm relative to tetramethylsilane (δ = 0.00 ppm) using the residual solvent signal as an internal reference. ^{13}C chemical shifts are reported in ppm with $CDCl_3$ (δ = 77.67 ppm), CD_3OD (δ = 49.30 ppm) or $(CD_3)_2SO$ (δ = 39.52 ppm) as internal references. Structural assignments were made using additional information from gHSQC and gHMBC experiments. The deuterated solvents were kept under a nitrogen atmosphere.

Absorption spectra were obtained with matched 1.0 cm quartz cuvettes using a UV-vis spectrometer. Molar absorption coefficients were determined from the absorption spectra (the average values were obtained from three independent measurements with solutions of different concentrations). Fluorescence was measured on an automated luminescence spectrometer in 1.0 cm quartz fluorescence cuvettes at 23 ± 1 °C; sample concentrations with an absorbance below 0.1 at the excitation wavelength were used. Fluorescence quantum yields were determined on a fluorimeter as absolute values using an integrating sphere. The quantum yields were measured five times and were averaged for each sample. The solution concentrations were adjusted to have absorbance below 0.15.

The exact masses of the synthesized compounds were obtained using a triple quadrupole electrospray ionization (ESI) mass spectrometer in a positive or negative mode coupled with direct inlet or liquid chromatography (nitrogen flow 5 L min^{-1}, gas temperature: 325 °C, nebulizer 45 psig, skimmer 65 V, V_{cap} –2500 V, fragmentor: 60 V; in methanol) and an atmospheric pressure chemical ionization (APCI) mass spectrometer in a positive or negative mode coupled with direct inlet or liquid chromatography (nitrogen flow 5 L min^{-1}, gas temperature: 325 °C, nebulizer 45 psig, skimmer 65 V, vaporizer 200 °C, fragmentor: 60 V; in dichloromethane).

Singlet Oxygen Production. Determination of the singlet oxygen production quantum yield (Φ_Δ): a solution of 1,3-diphenylisobenzofuran (DPBF; $c = 5 \times 10^{-5}$) and a Cy5 or Cy7 derivative ($c \sim 1 \times 10^{-5}$ M) in methanol was prepared. The stirred solution (3.0 mL) in a quartz cell (1 cm) was irradiated using LEDs at 730 nm for Cy7 and 638 nm for Cy5, and the UV-vis spectra were recorded periodically. The irradiation period was selected to reach a ~10% consumption of DPBF. The procedure was repeated three times. The decomposition of DPBF monitored at 411 nm was fitted with a pseudo-first-order rate law, and the singlet oxygen formation quantum yield (Φ_Δ) was calculated using that of unsubstituted Cy7 as a reference (Φ_Δ = 0.009)[1] for **12**, **13**, **14**, **15**, **16**, and **17**. Methylene blue (Φ_Δ = 0.49)[2] was used as a reference for compounds **2**, **3**, **4**, **7**, **8**, and **9**.

Photostability. The decomposition quantum yields (Φ_{dec}) of **12**, **13**, **14**, **15**, **16**, and **17** in an aerated methanol solution were determined using an LED source (λ_{irr} = 730 nm). The quantum yields were measured three times and were averaged for each sample. Unsubstituted (parent) Cy7

($\Phi_{dec} = 3.1 \times 10^{-6}$)[1] was used as a reference. The decomposition quantum yields of **2**, **3**, **4**, **5**, **6**, **7**, **8**, **9** and **14** in an aerated methanol solution were determined using an LED source ($\lambda_{irr} = 638$ nm). The Φ_{dec} values were measured three times and were averaged for each sample. The quantum yield of decomposition of unsubstituted (parent) Cy5 at 640 nm ($\Phi_{dec} = 3.7 \times 10^{-7}$) was determined using a photodiode and was used as a reference.

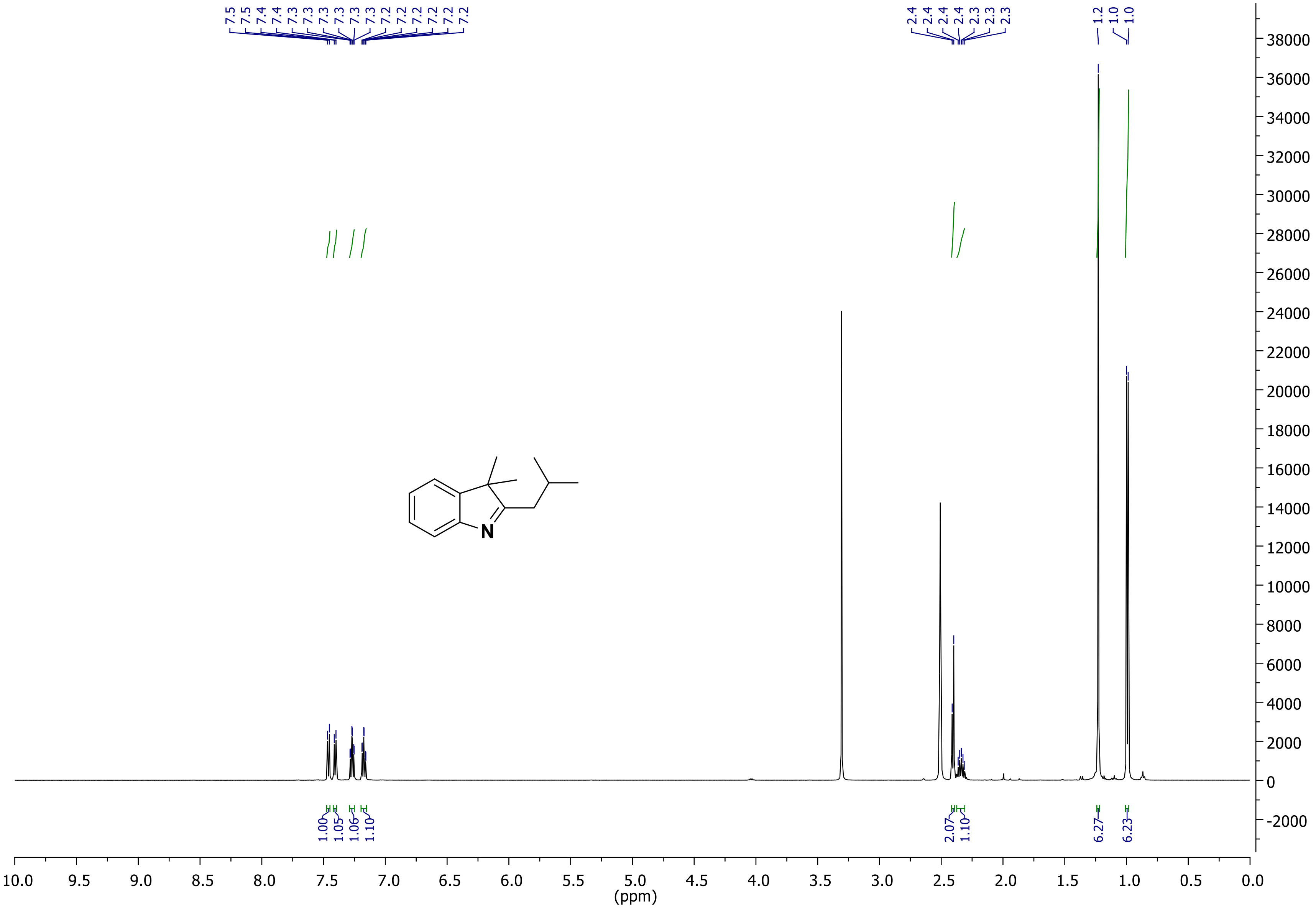

Figure S1. ^{1}H NMR (500 MHz, d_6-DMSO): **29**

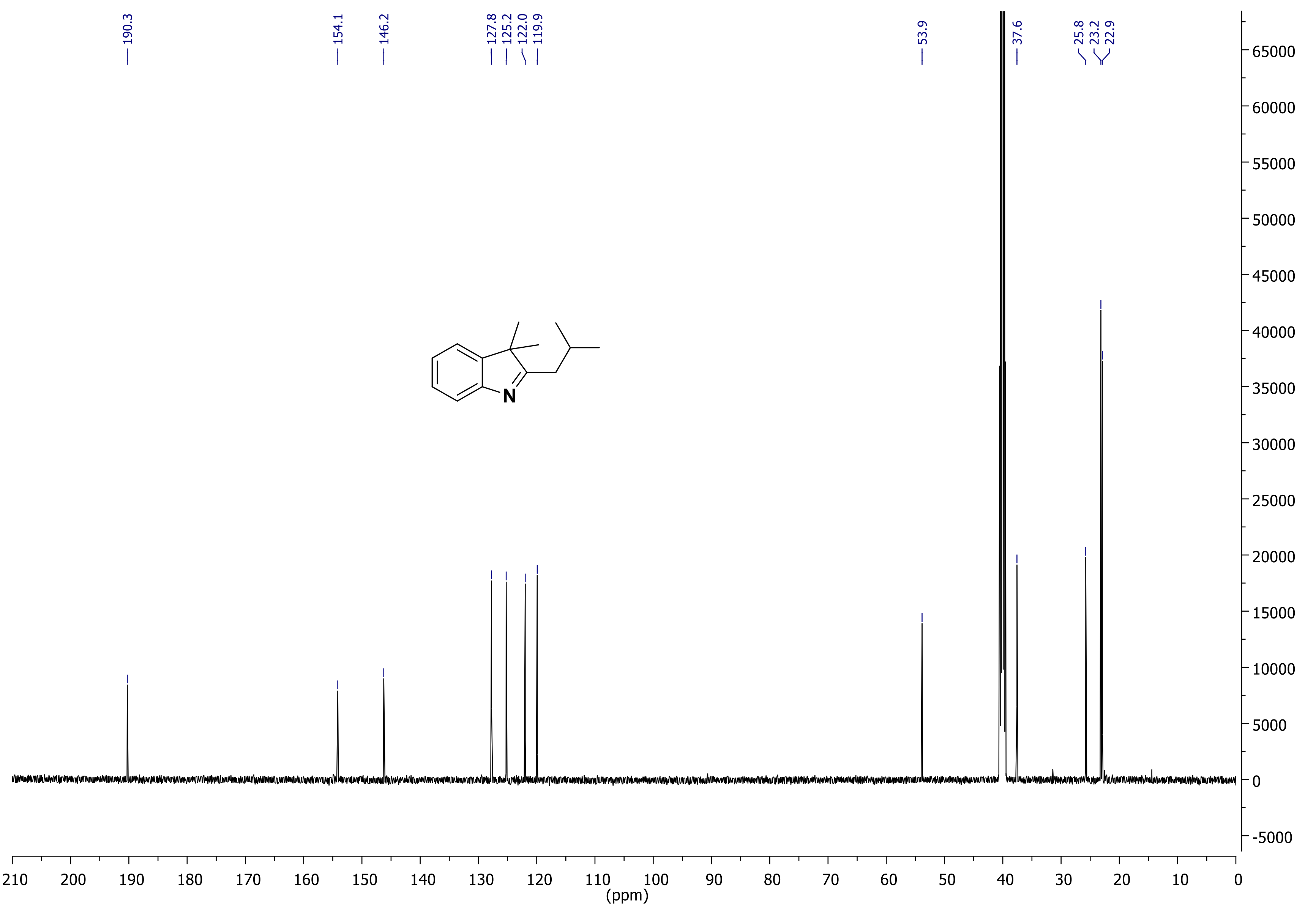

Figure S2. $^{13}C\{^{1}H\}$ NMR (126 MHz, d_6-DMSO): **29**

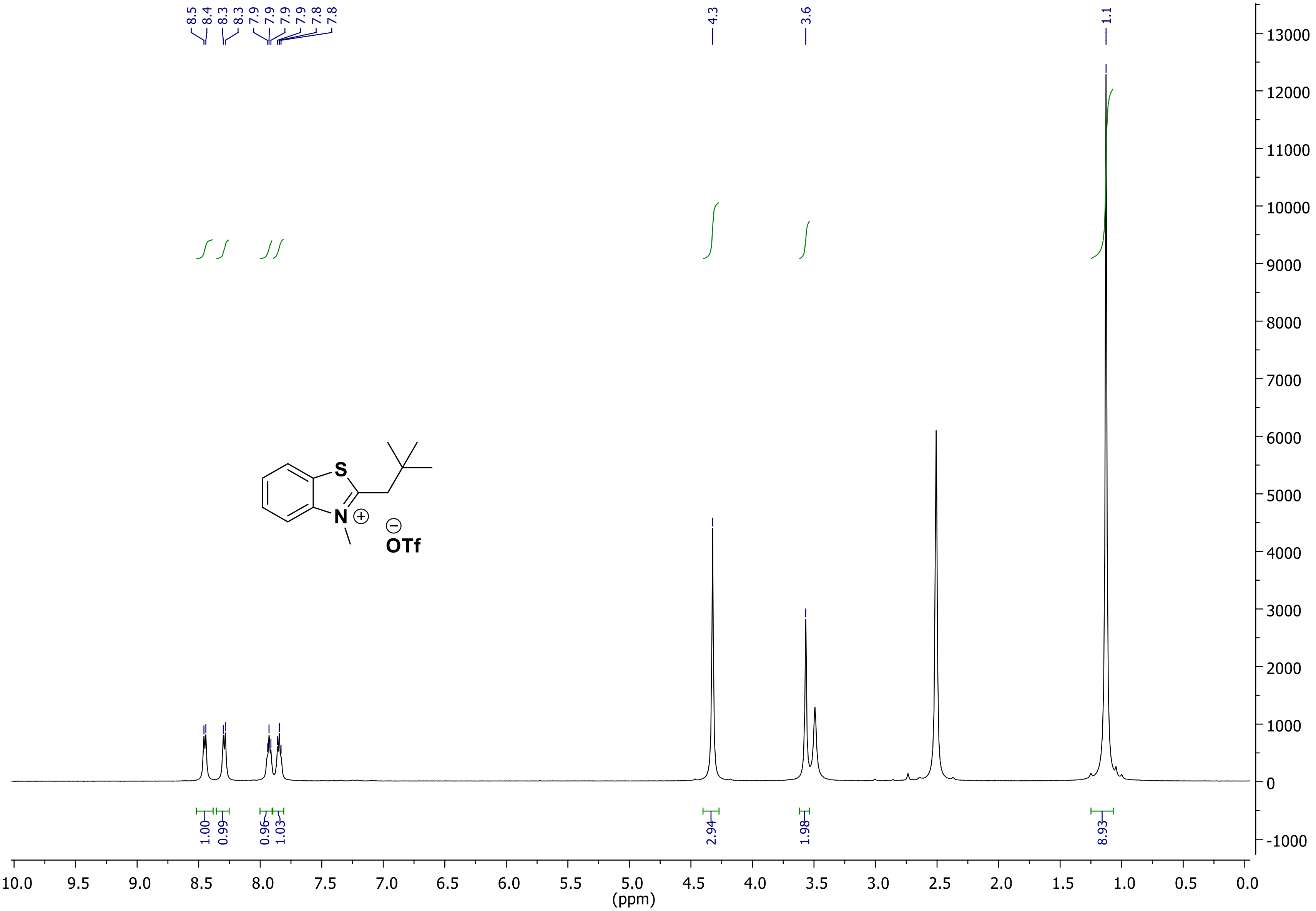

Figure S3. ^{1}H NMR (500 MHz, d_6-DMSO): **30**

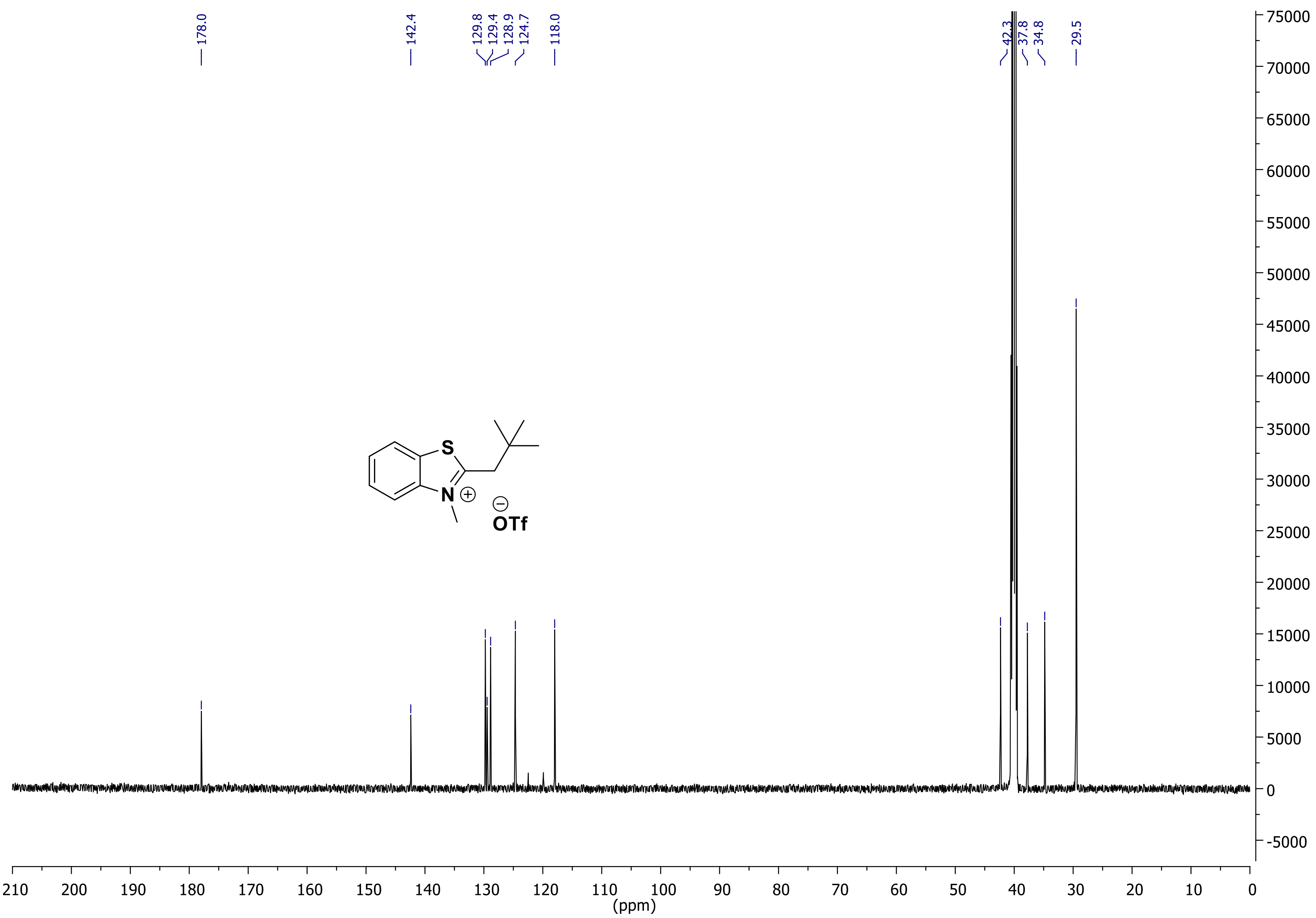

Figure S4. $^{13}C\{^{1}H\}$ NMR (126 MHz, d_6-DMSO): **30**

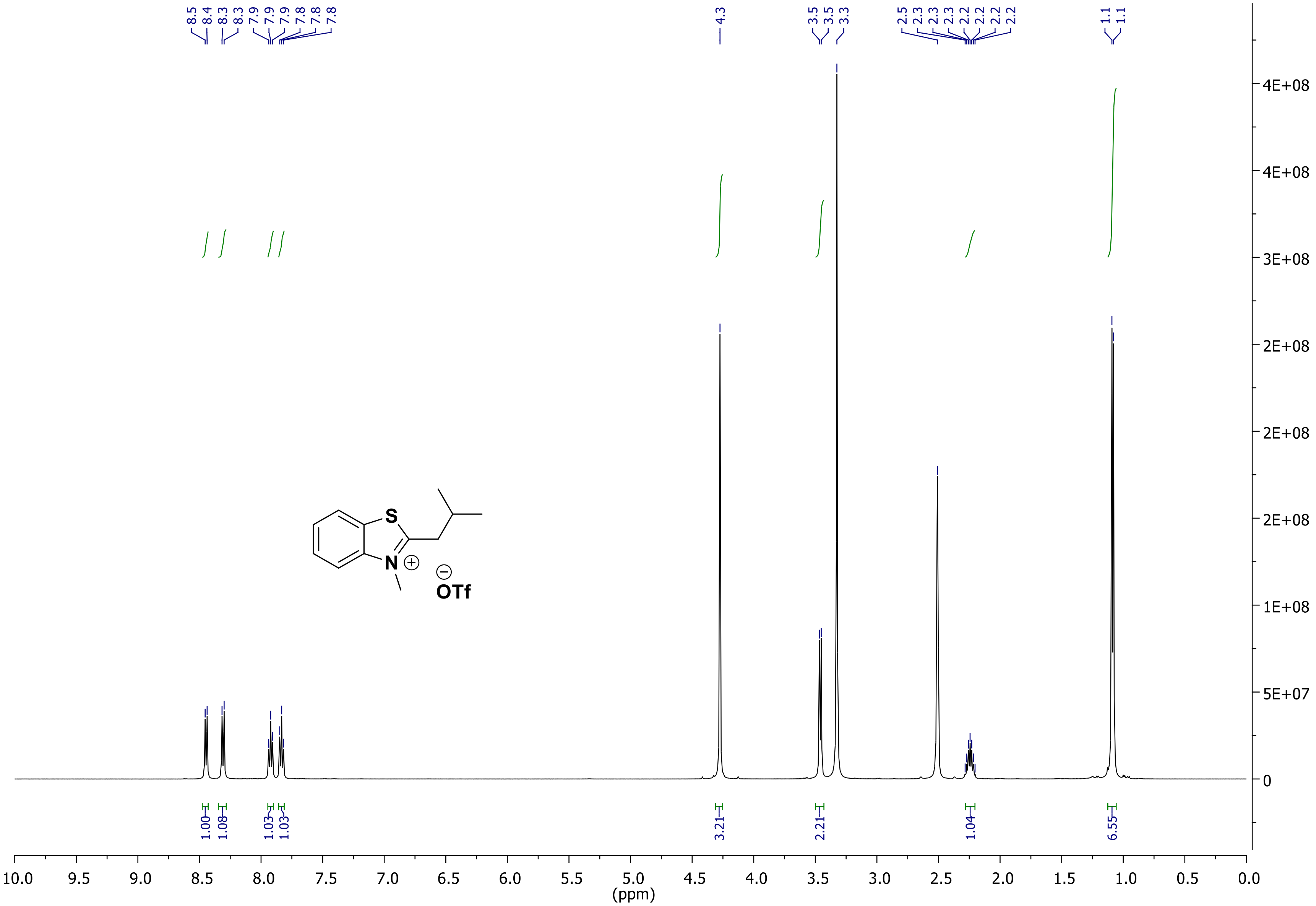

Figure S5. ^{1}H NMR (500 MHz, d_6-DMSO): **31**

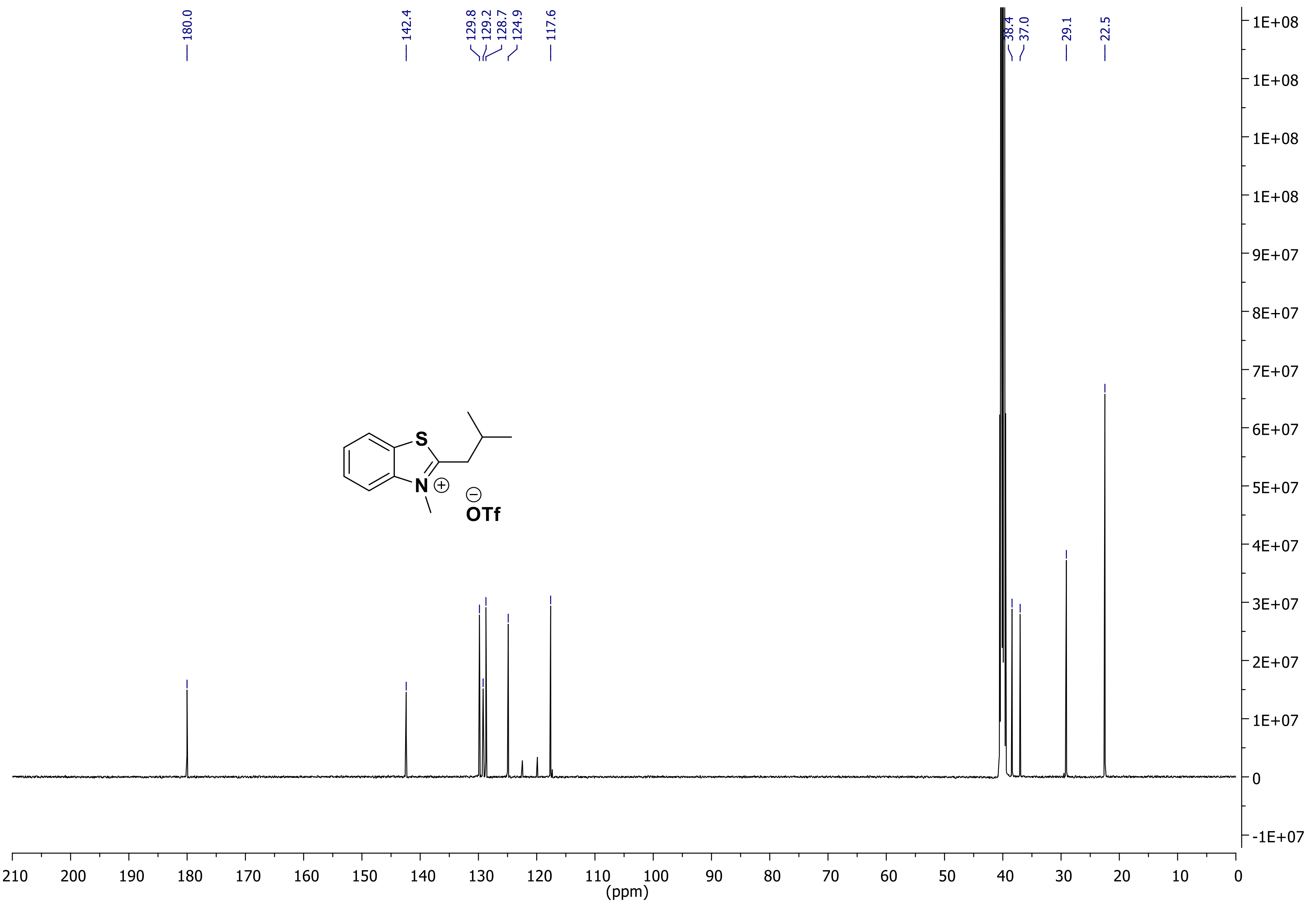

Figure S6. $^{13}C\{^1H\}$ NMR (126 MHz, *d*$_6$-DMSO): **31**

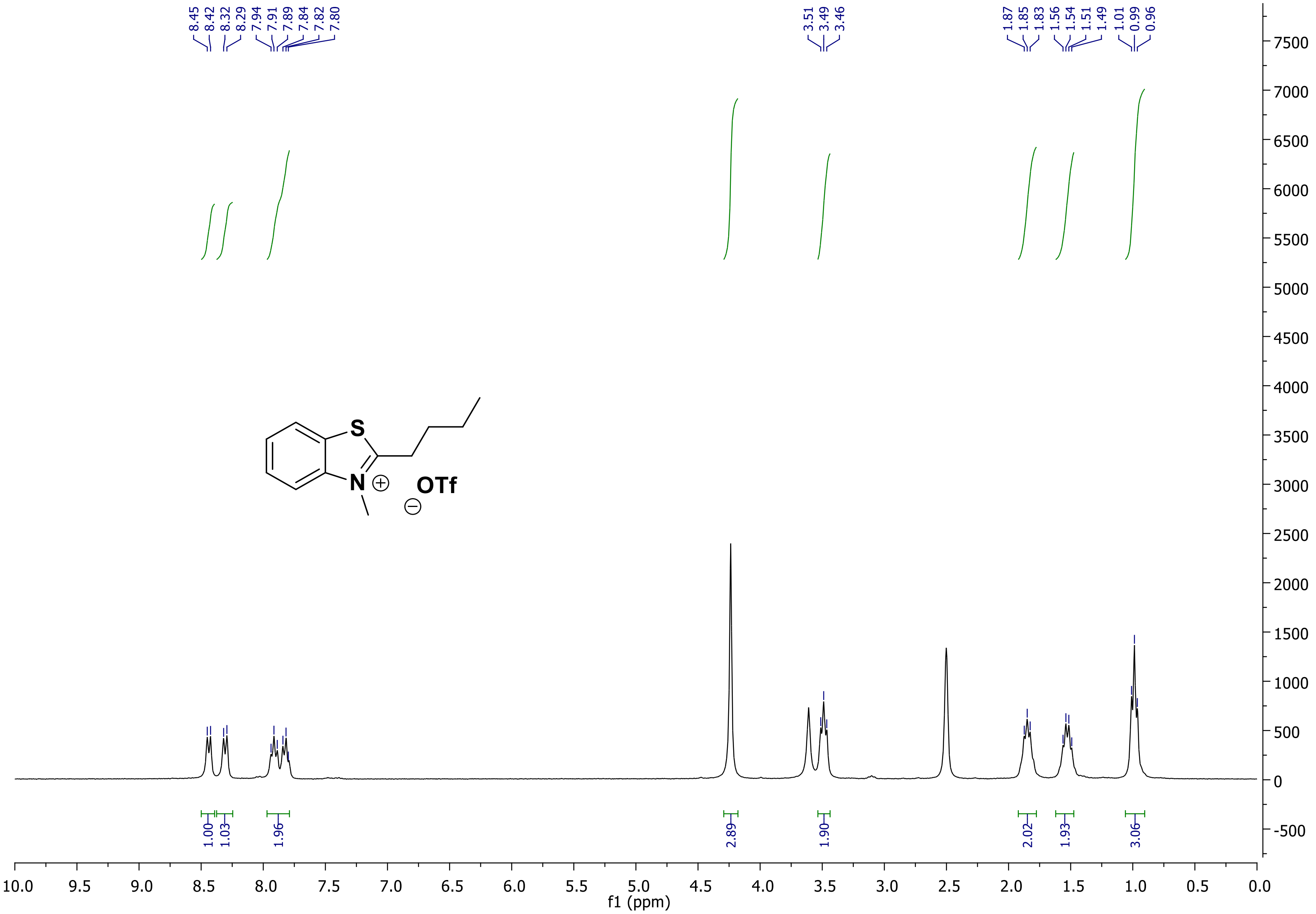

Figure S6a. ^{1}H NMR (500 MHz, d_6-DMSO): **32**

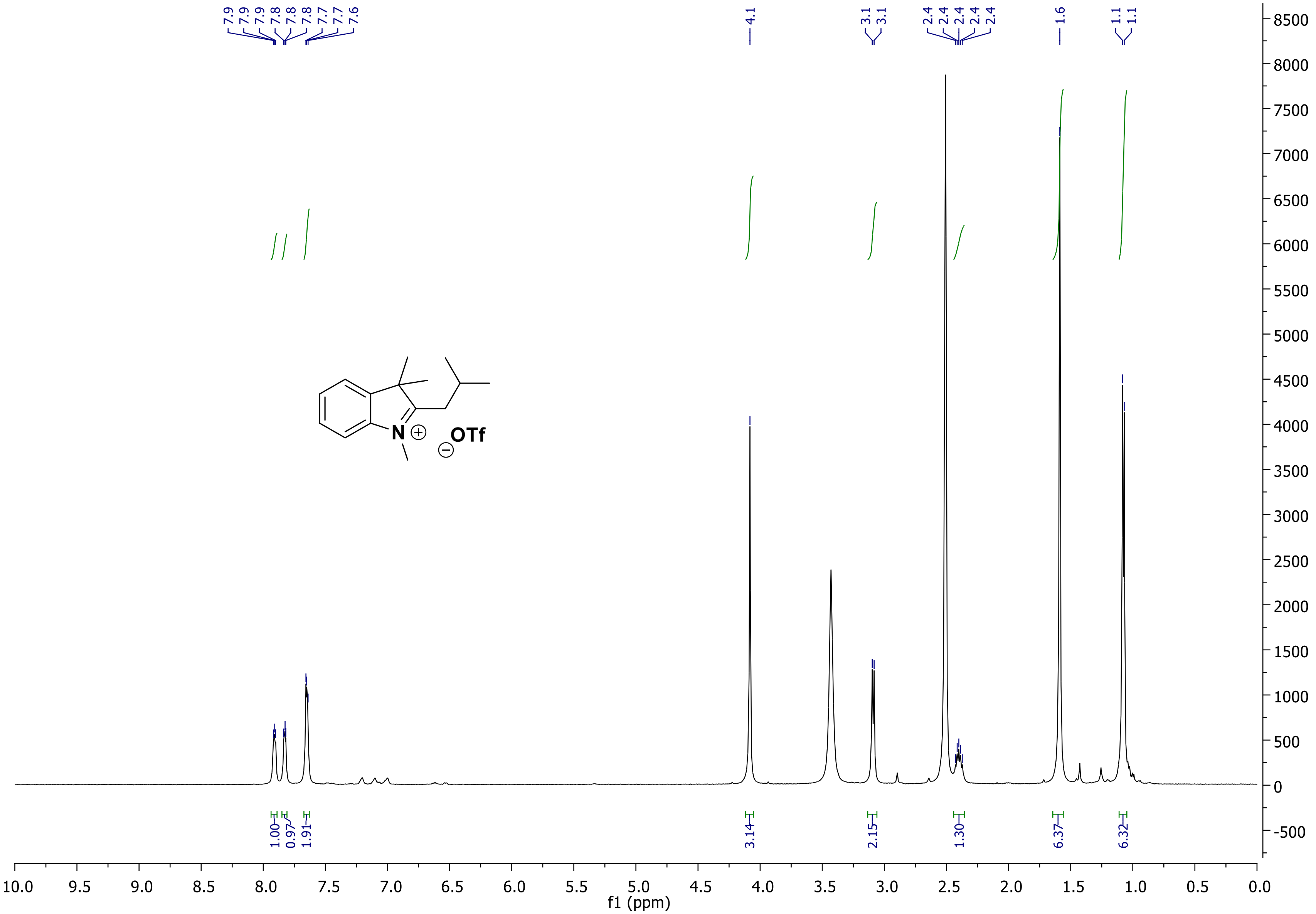

Figure S7. ^{1}H NMR (500 MHz, d_6-DMSO): **33**

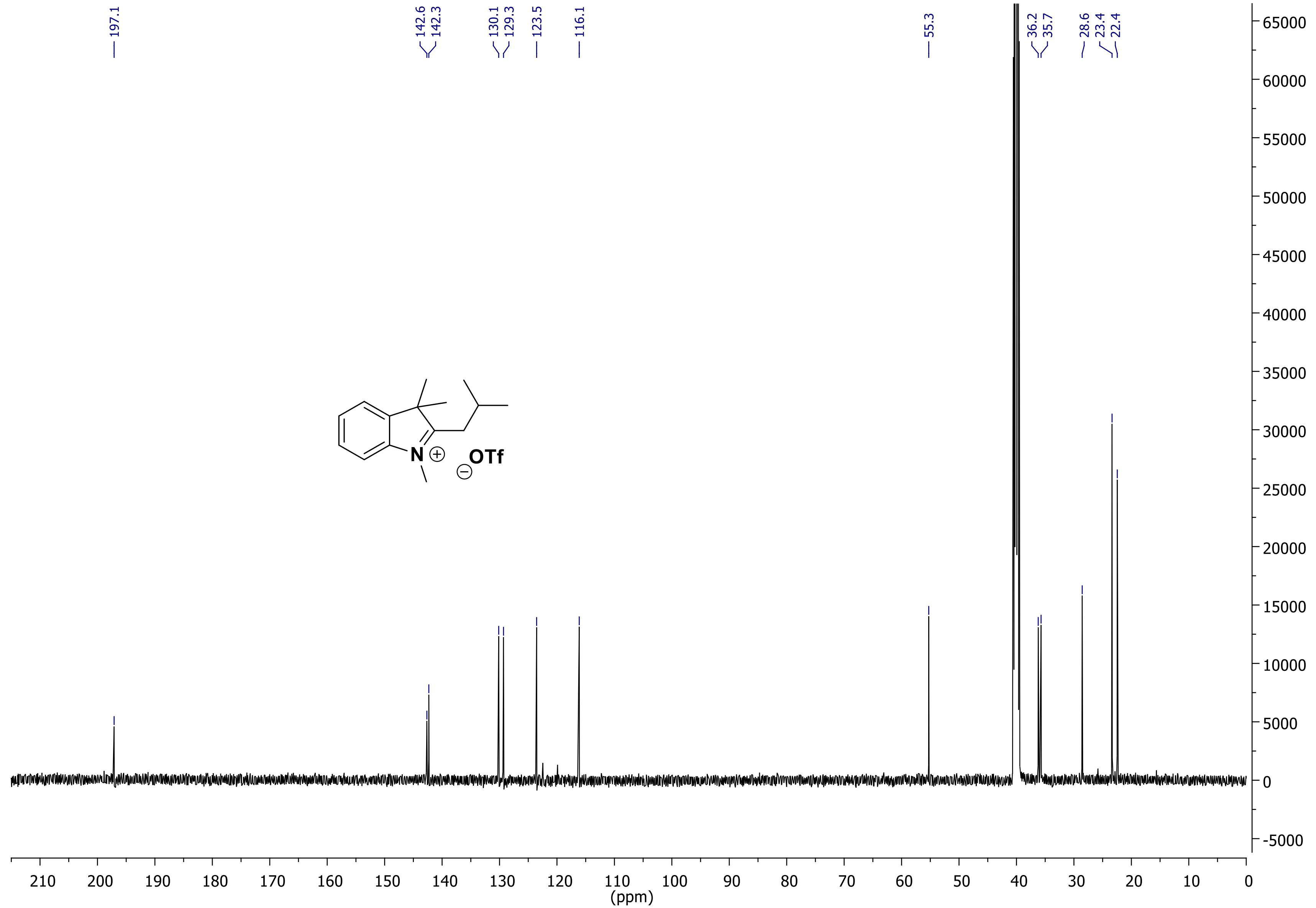

Figure S8. $^{13}C\{^{1}H\}$ NMR (126 MHz, d_6-DMSO): **33**

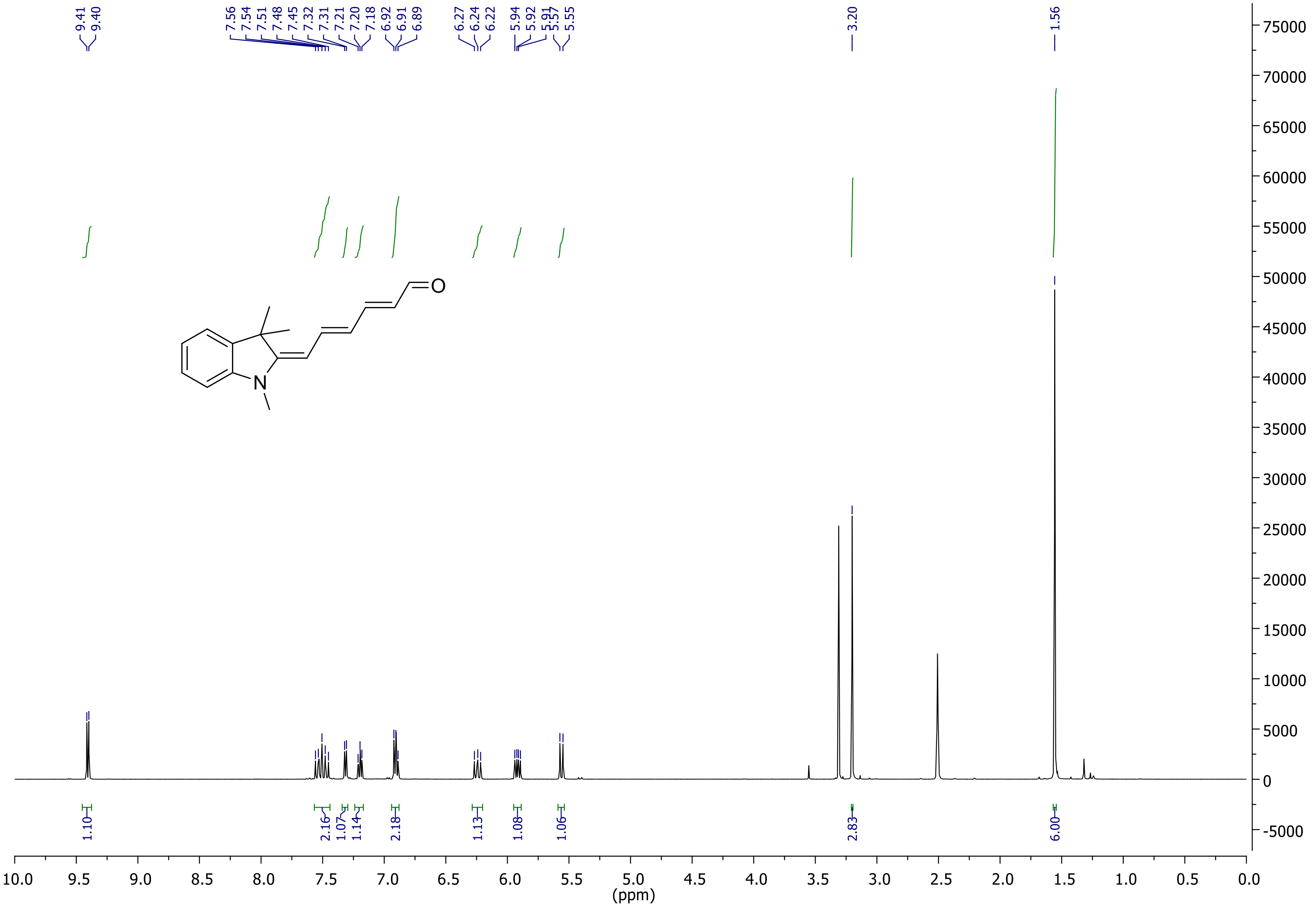

Figure S9. ^{1}H NMR (500 MHz, d_6-DMSO): **24**

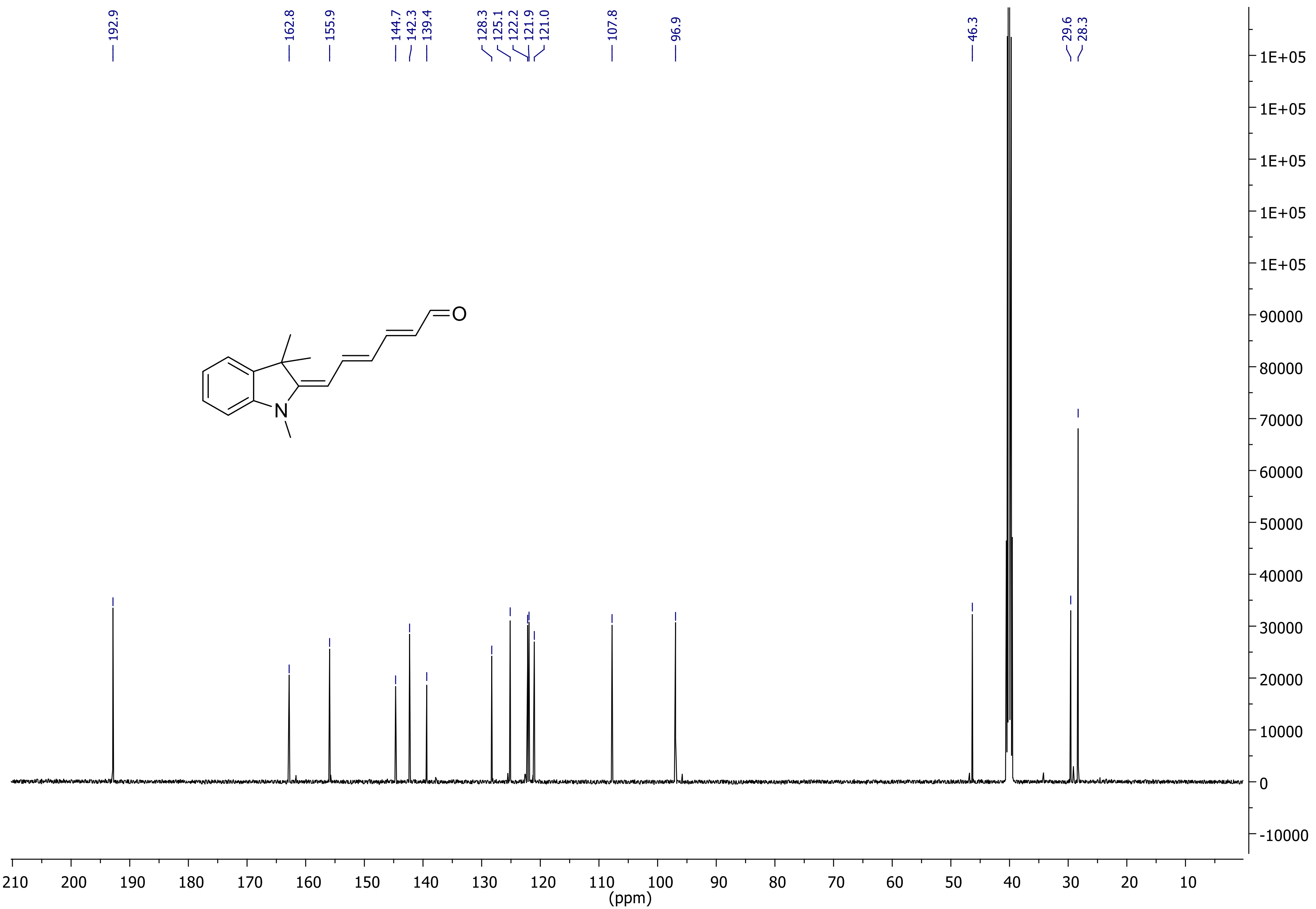

Figure S10. $^{13}C\{^{1}H\}$ NMR (126 MHz, d_6-DMSO): **24**

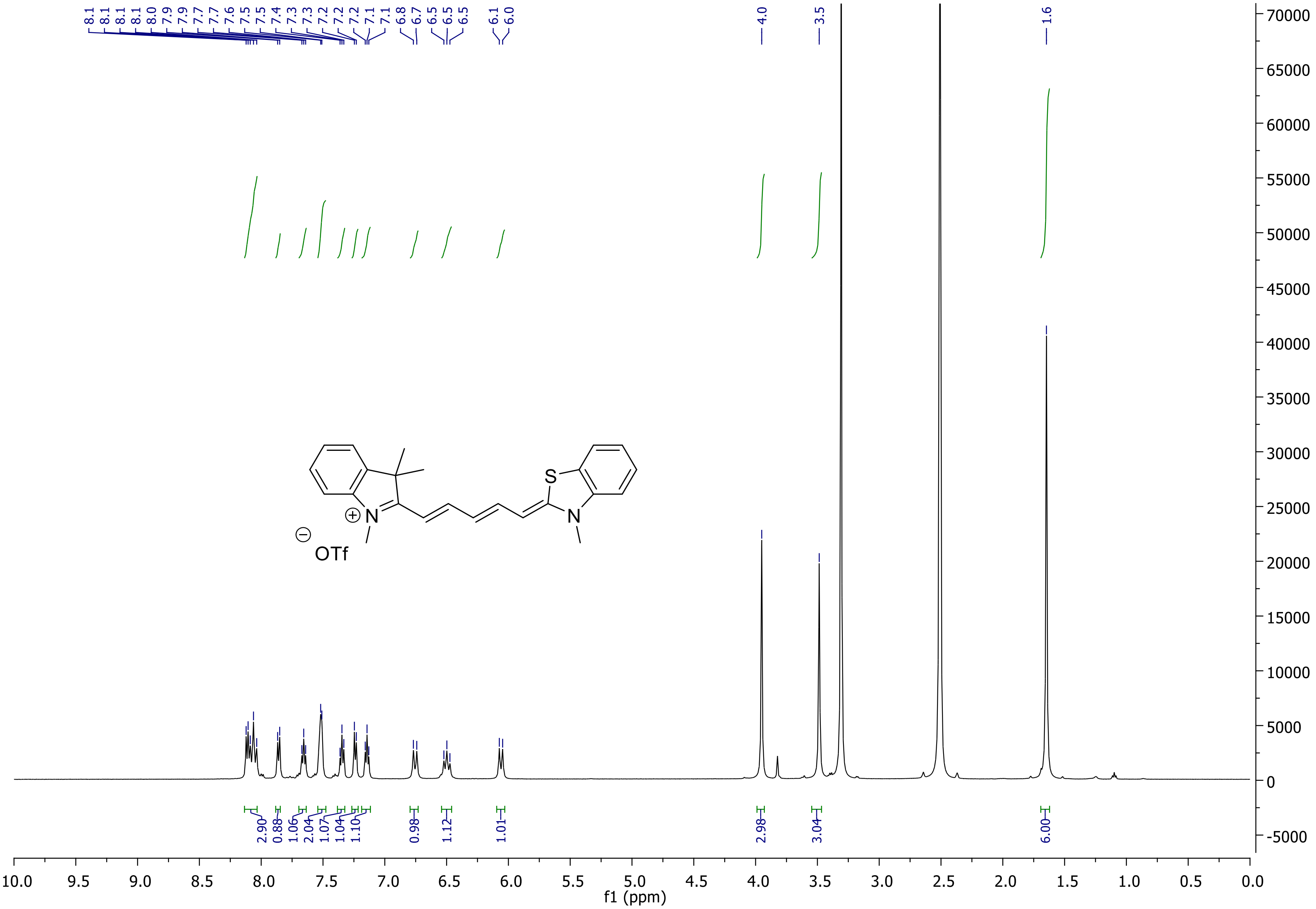

Figure S10b. ^{1}H NMR (500 MHz, d_6-DMSO): **2**

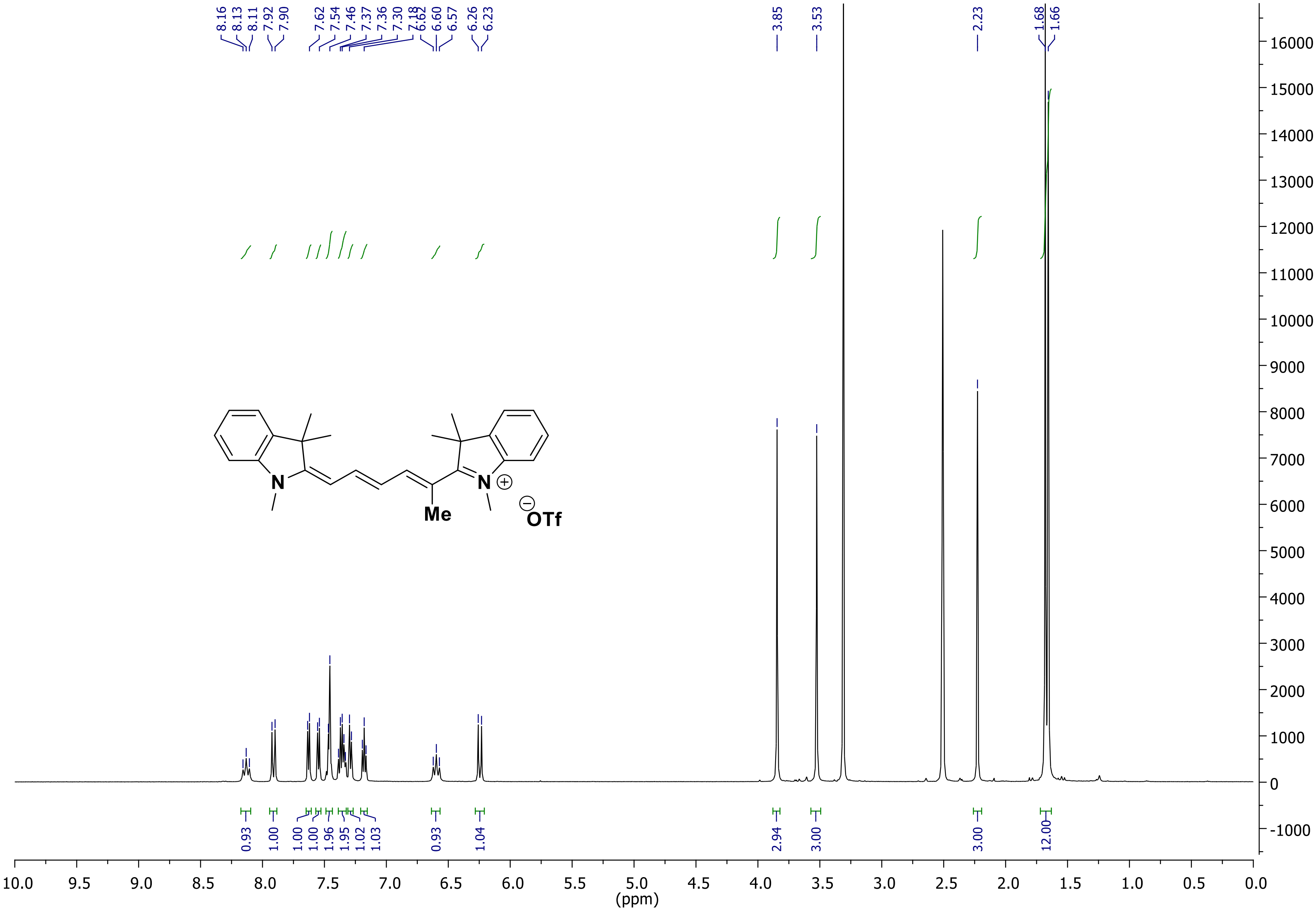

Figure S11. ^{1}H NMR (500 MHz, d_6-DMSO): **3**

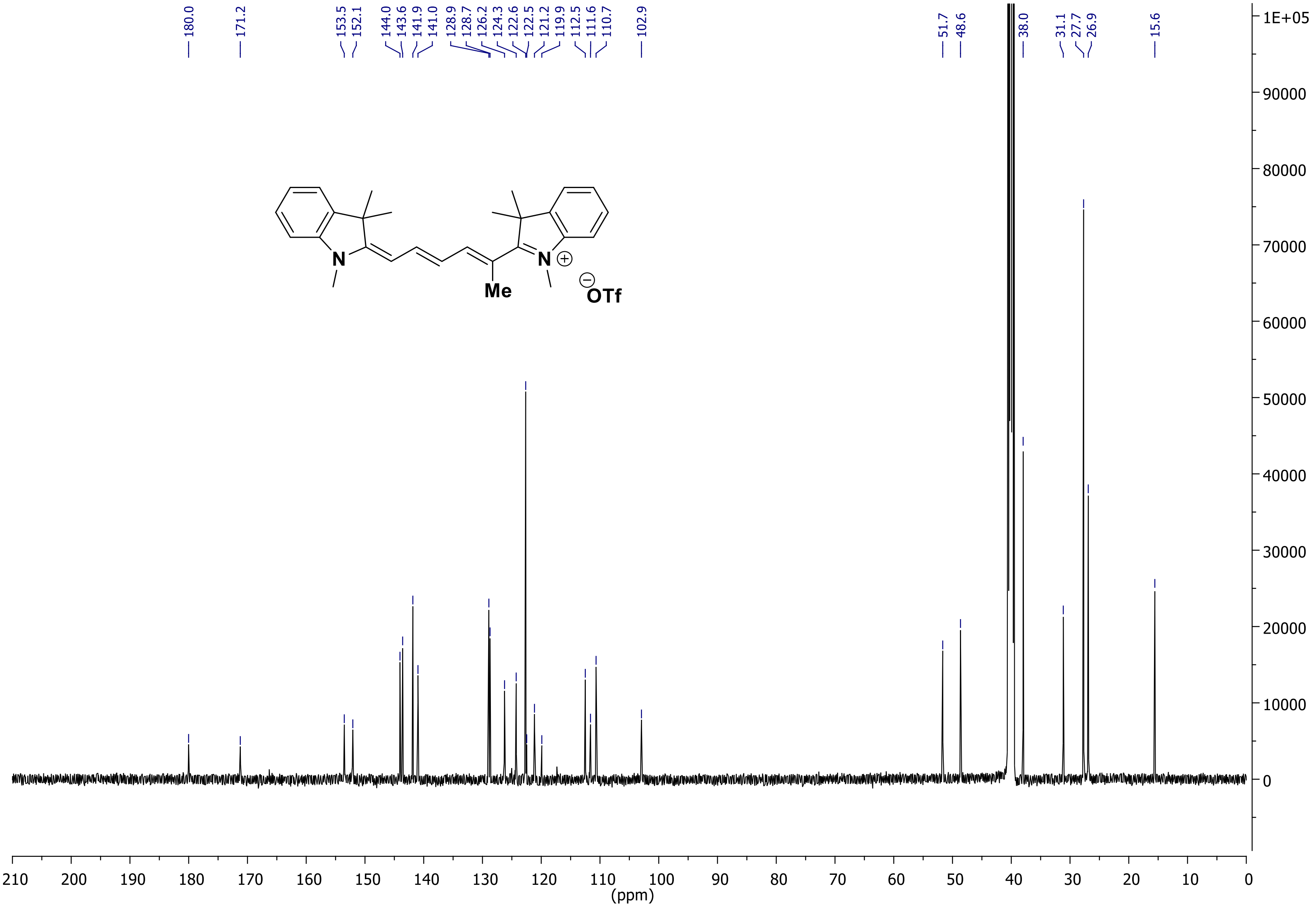

Figure S12. $^{13}C\{^{1}H\}$ NMR (126 MHz, d_6-DMSO): **3**

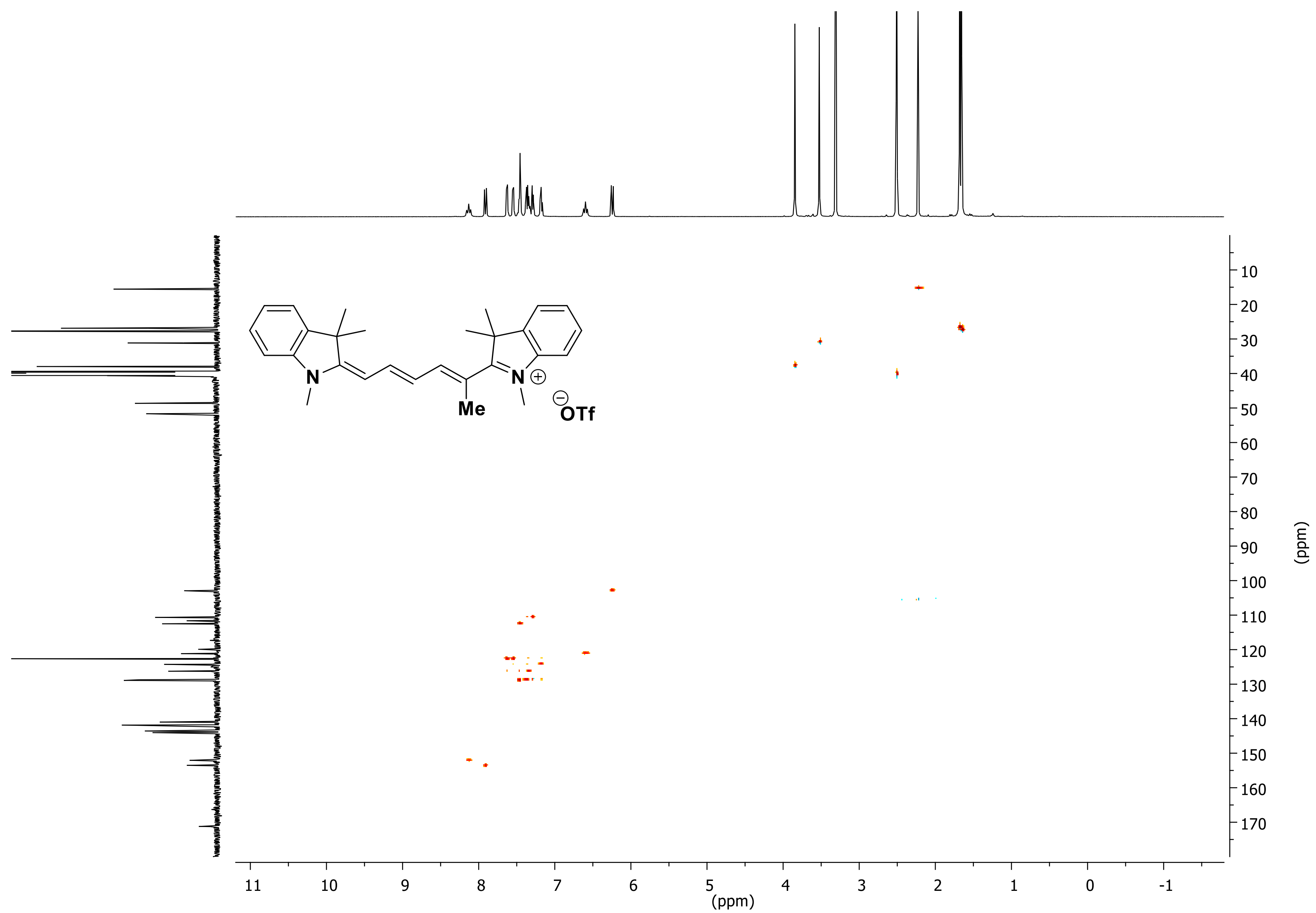

Figure S13. ^1H–$^{13}C\{^1H\}$ gHSQC (500 MHz, d_6–DMSO): **3**

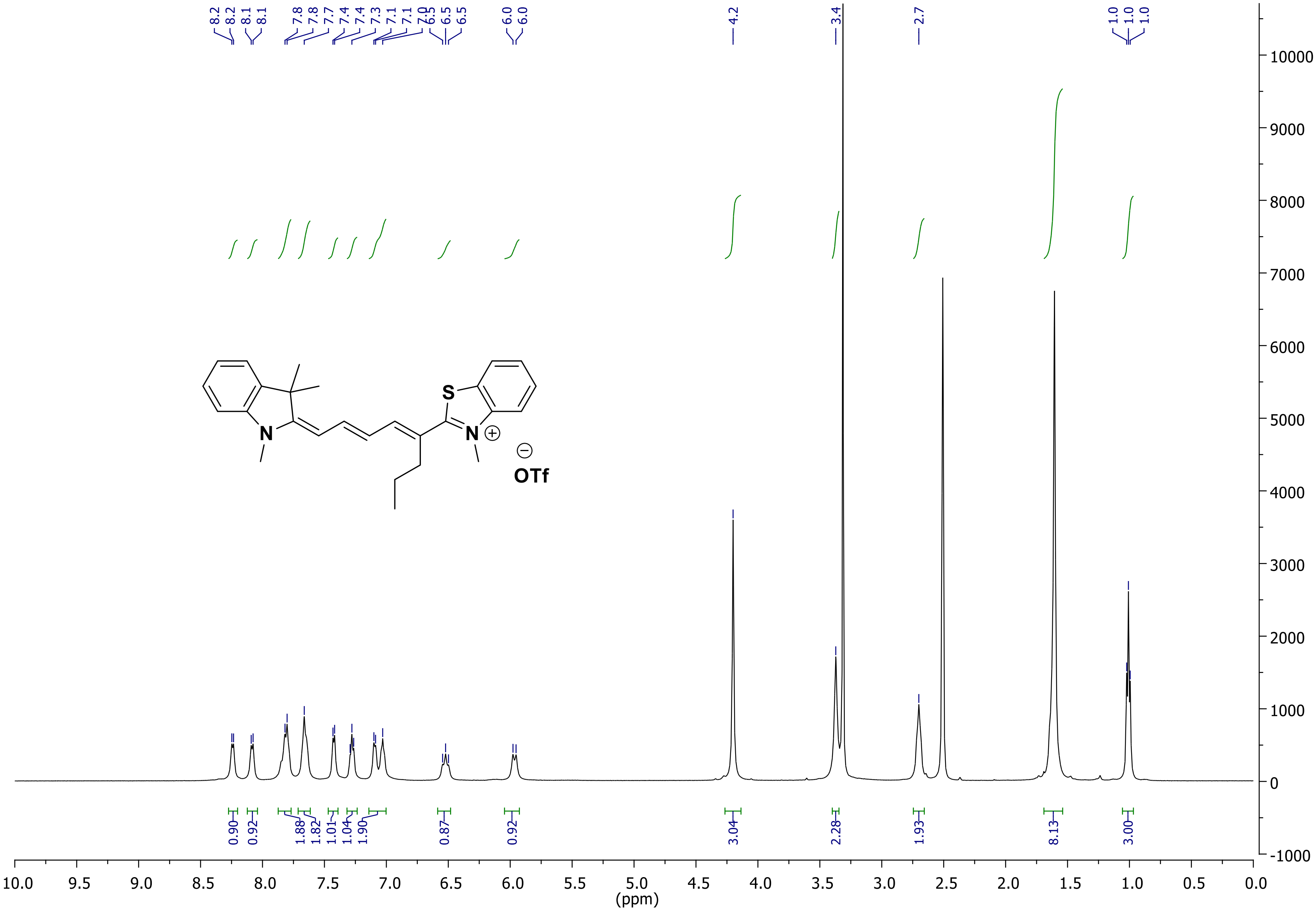

Figure S14. ^{1}H NMR (500 MHz, d_6-DMSO): **4**

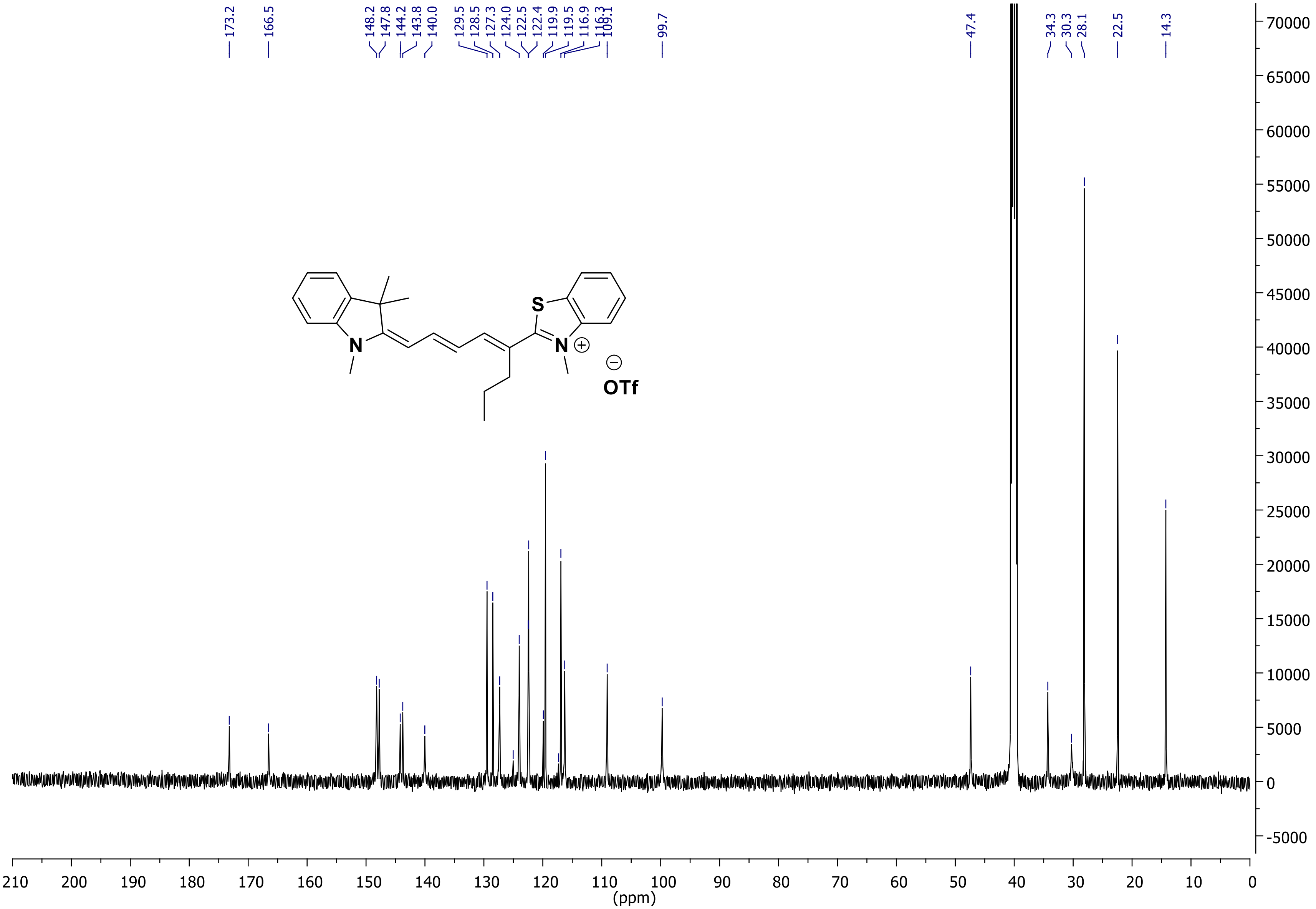

Figure S15. $^{13}C\{^{1}H\}$ NMR (126 MHz, d_6-DMSO): **4**

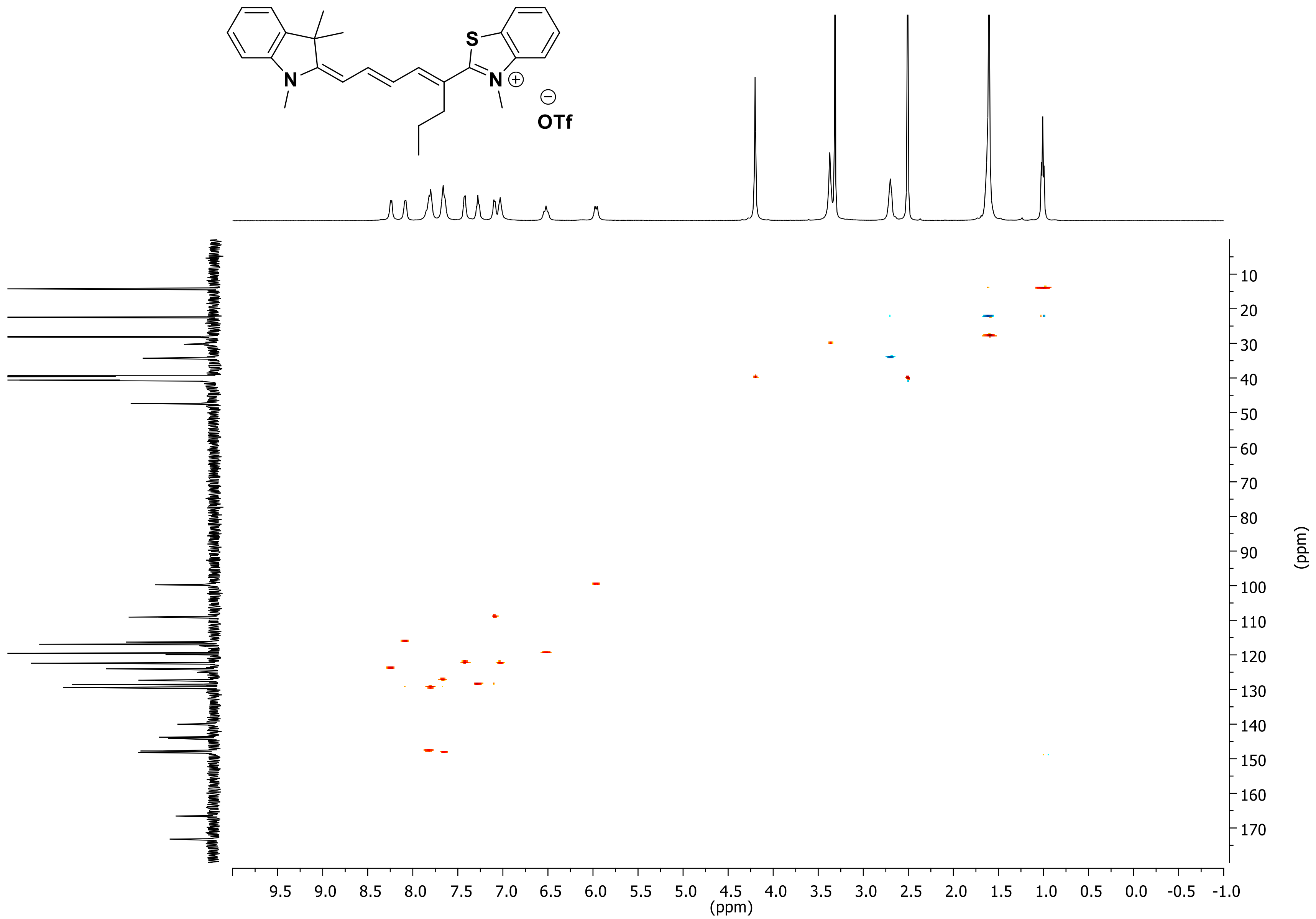

Figure S16. ^{1}H–$^{13}C\{^{1}H\}$ gHSQC (500 MHz, d_6–DMSO): **4**

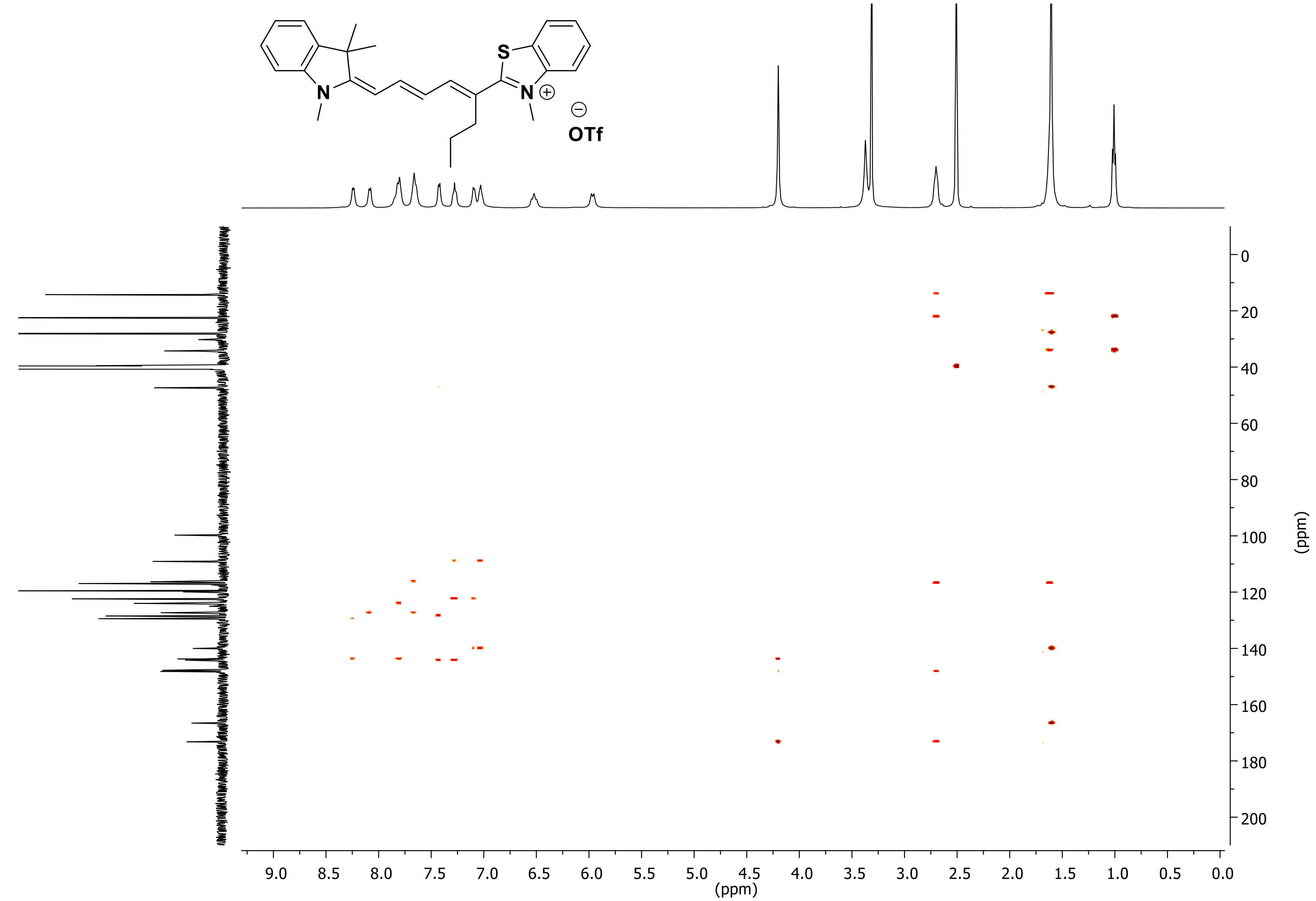

Figure S17. ^{1}H–$^{13}C\{^{1}H\}$ gHMBC (500 MHz, d_6–DMSO): **4**

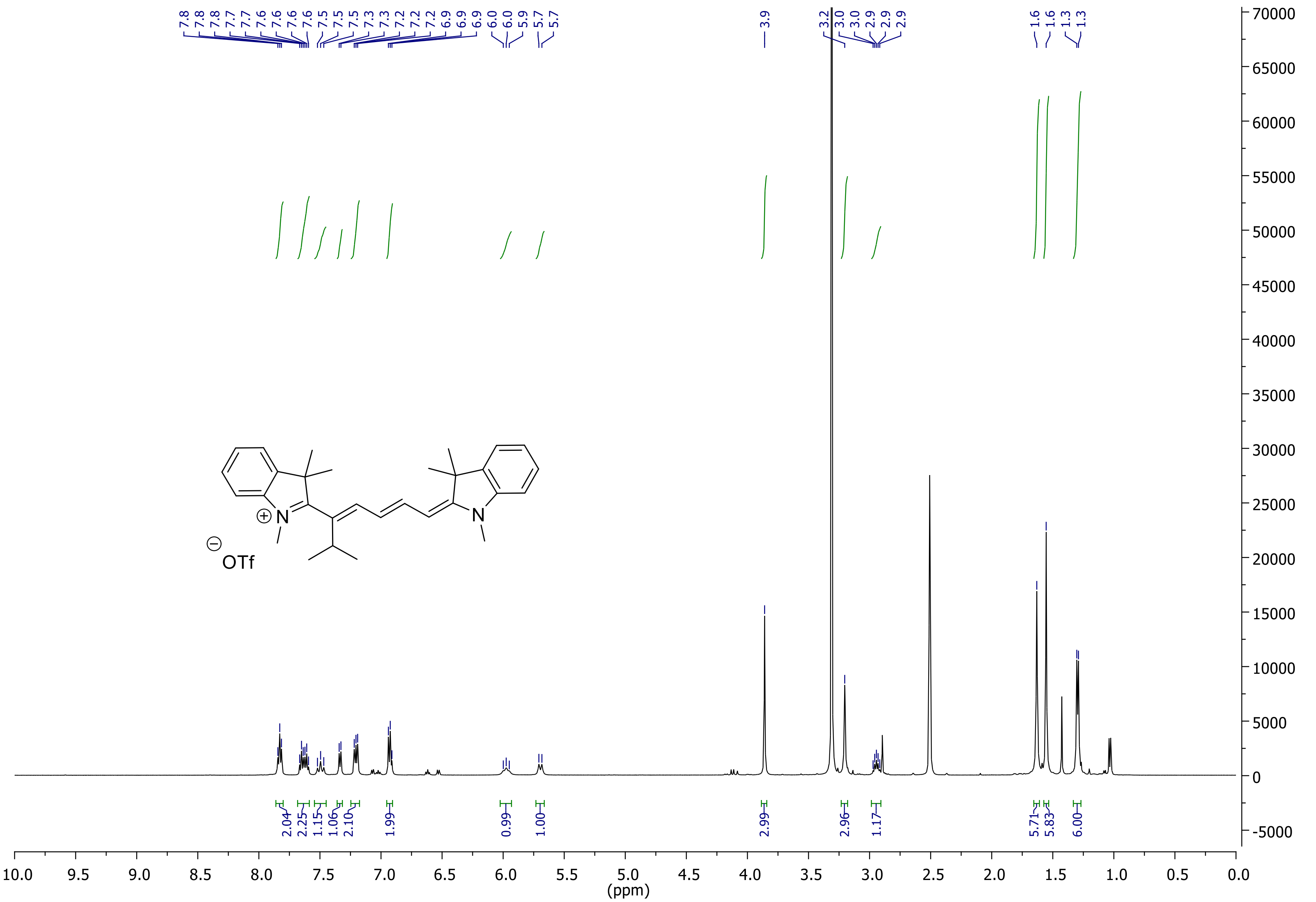

Figure S18. ^{1}H NMR (500 MHz, d_6–DMSO): **5**

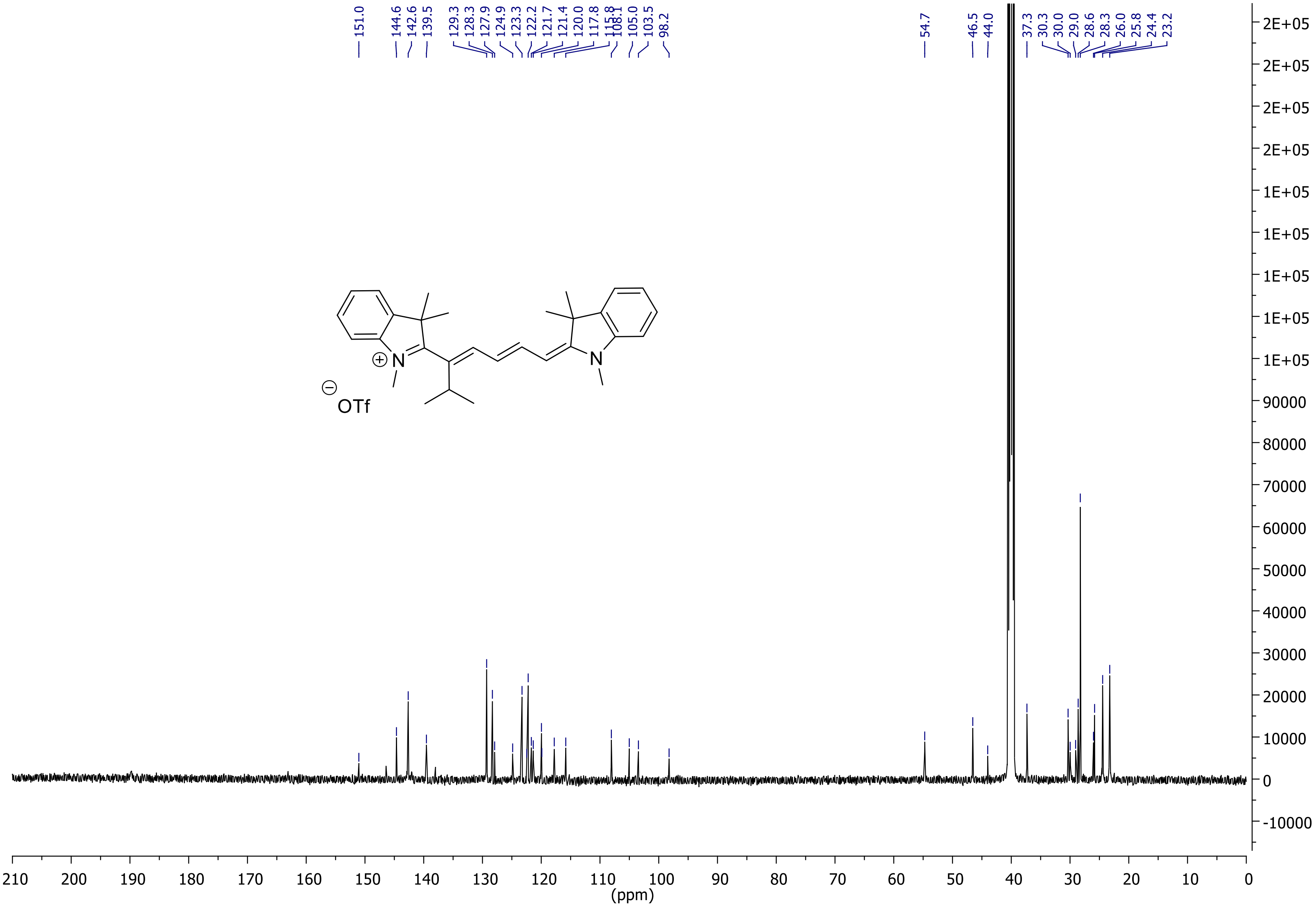

Figure S19. $^{13}C\{^{1}H\}$ NMR (126 MHz, d_6–DMSO): **5**

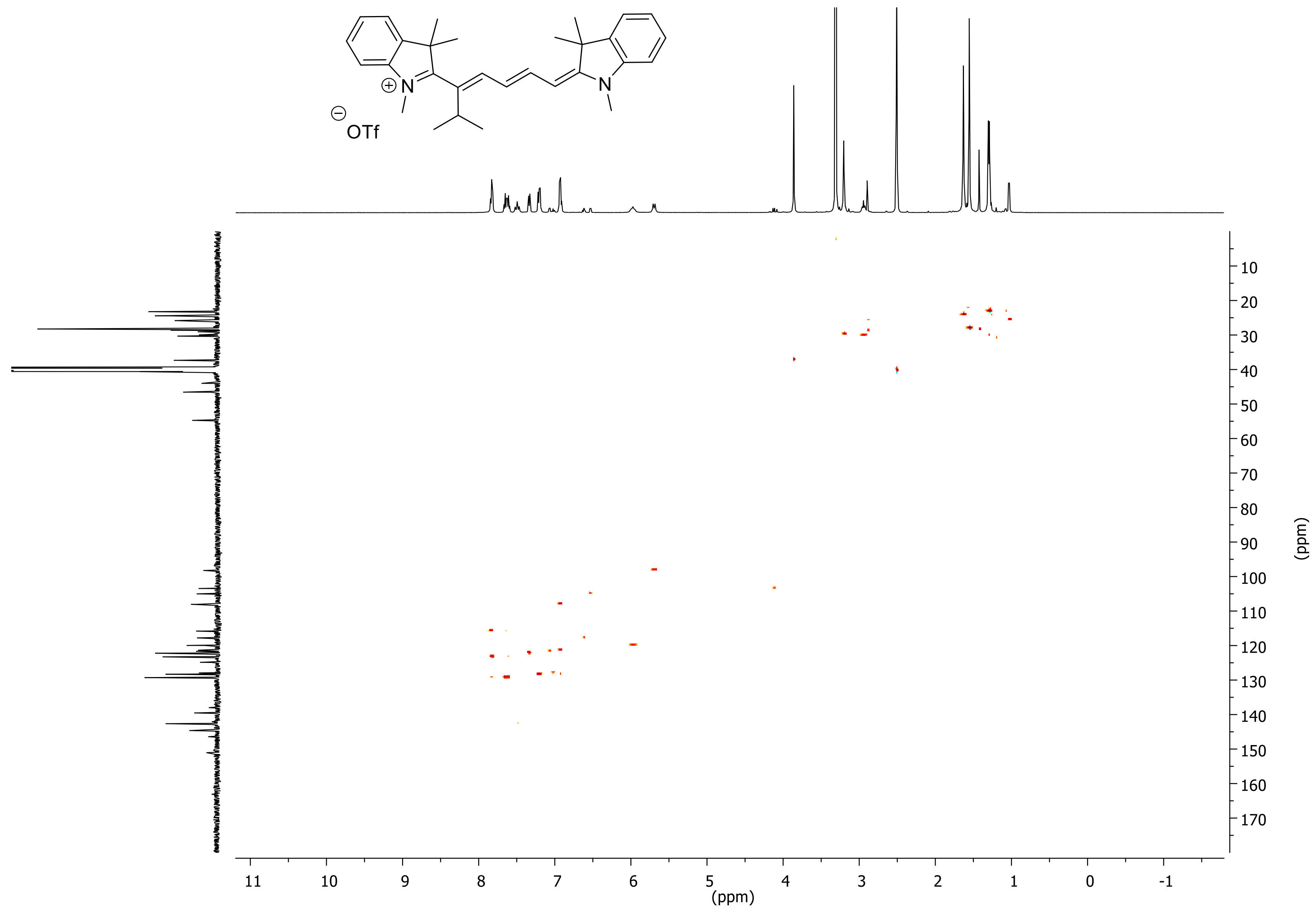

Figure S20. ^{1}H–$^{13}C\{^{1}H\}$ gHSQC (500 MHz, d_6–DMSO): **5**

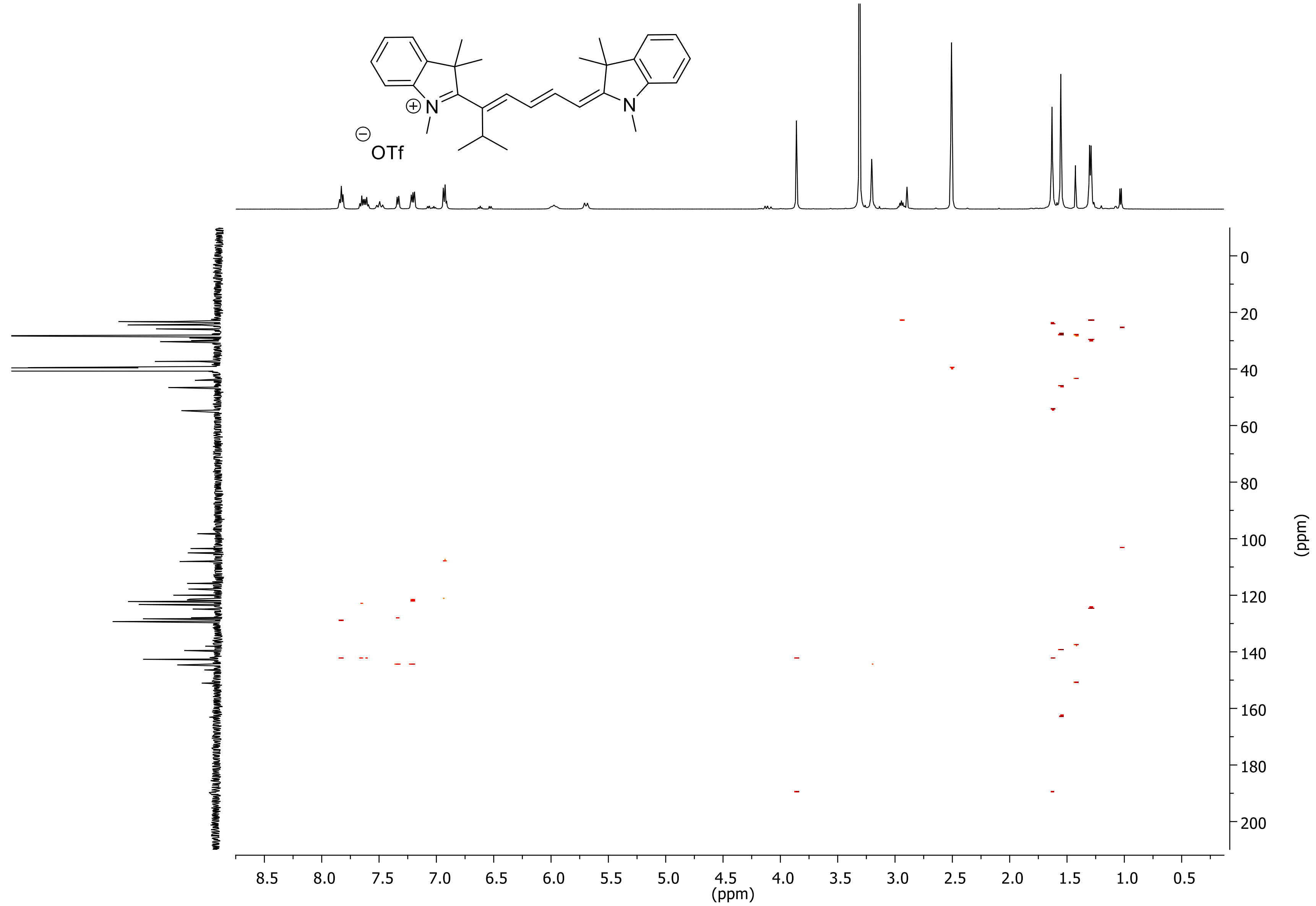

Figure S21. ^{1}H–$^{13}C\{^{1}H\}$ gHMBC (500 MHz, d_6–DMSO): **5**

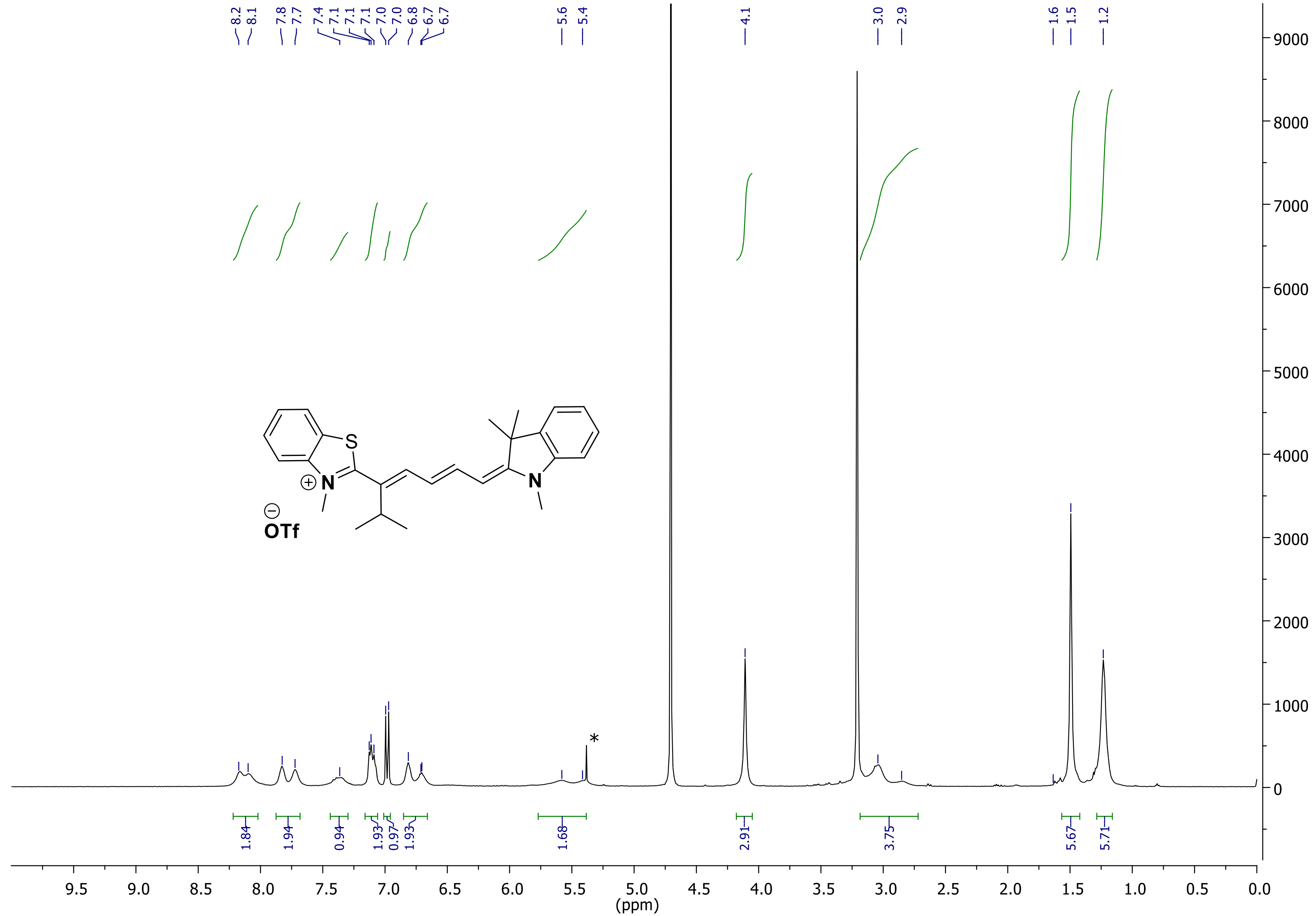

Figure S22. ^{1}H NMR (500 MHz, d_4–CD_3OD): **6** (*dichloromethane).

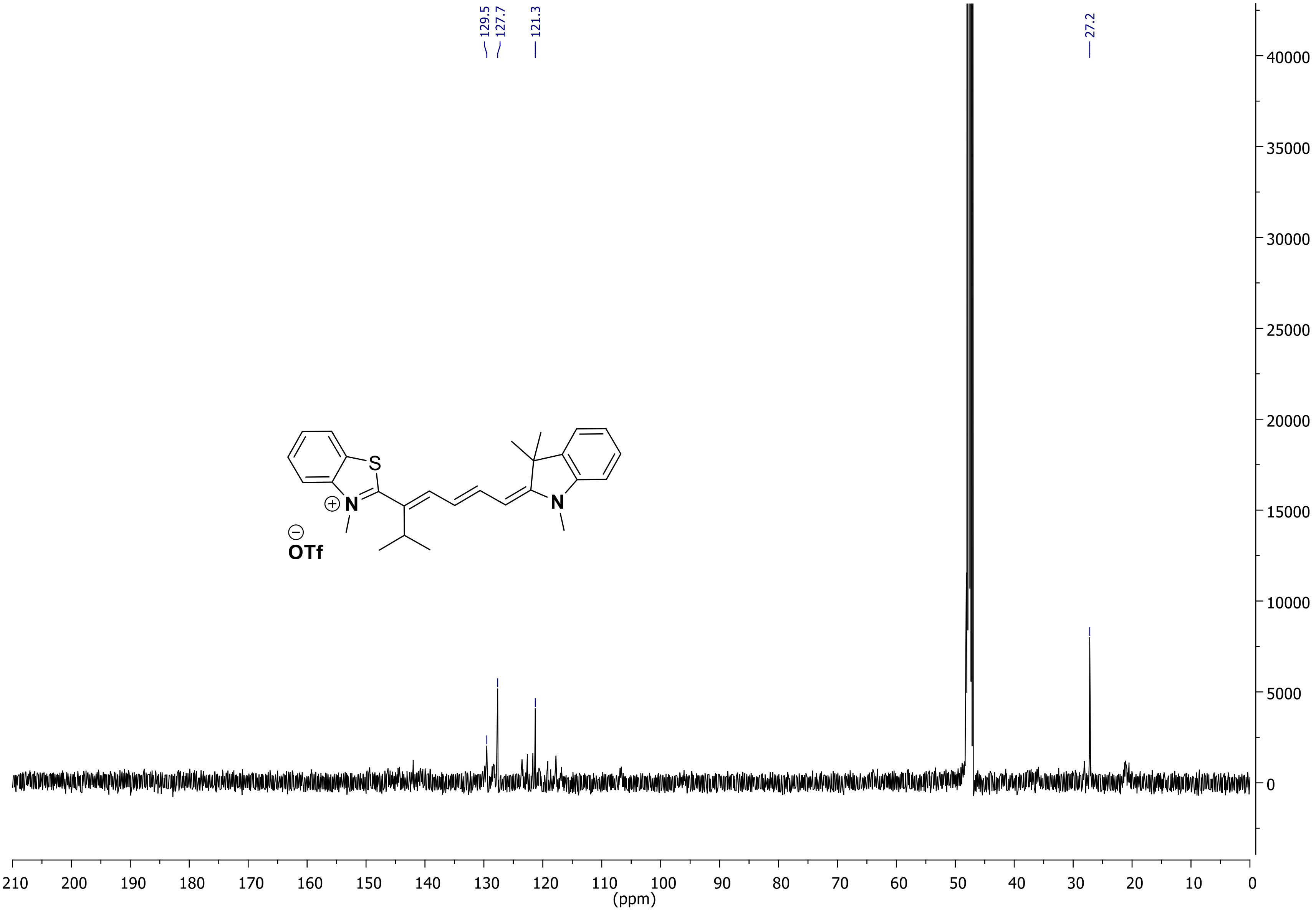

Figure S23. $^{13}C\{^{1}H\}$ NMR (126 MHz, d_4–CD_3OD): **6**

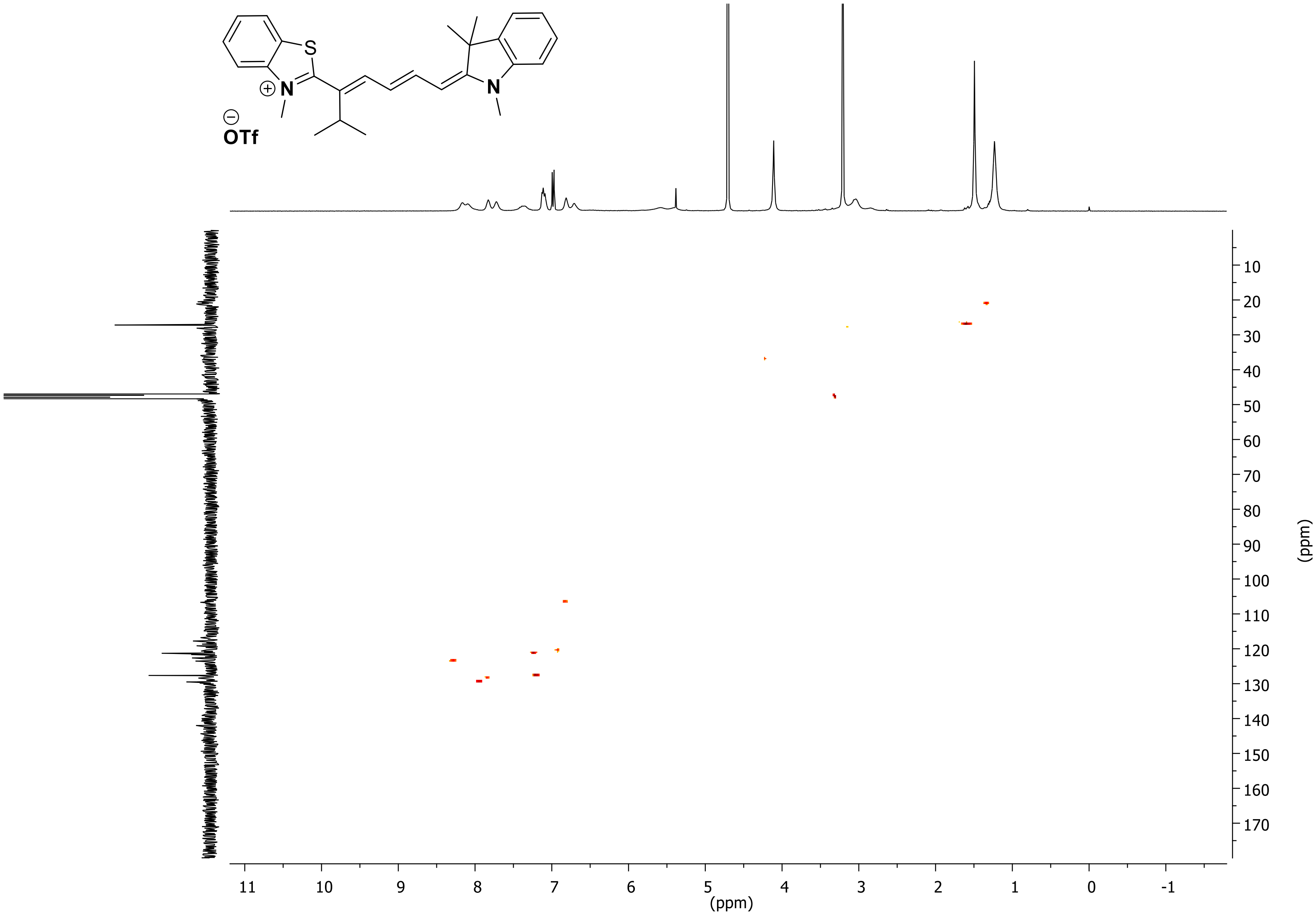

Figure S24. ^{1}H–$^{13}C\{^{1}H\}$ gHSQC (500 MHz, d_4–CD_3OD): **6**

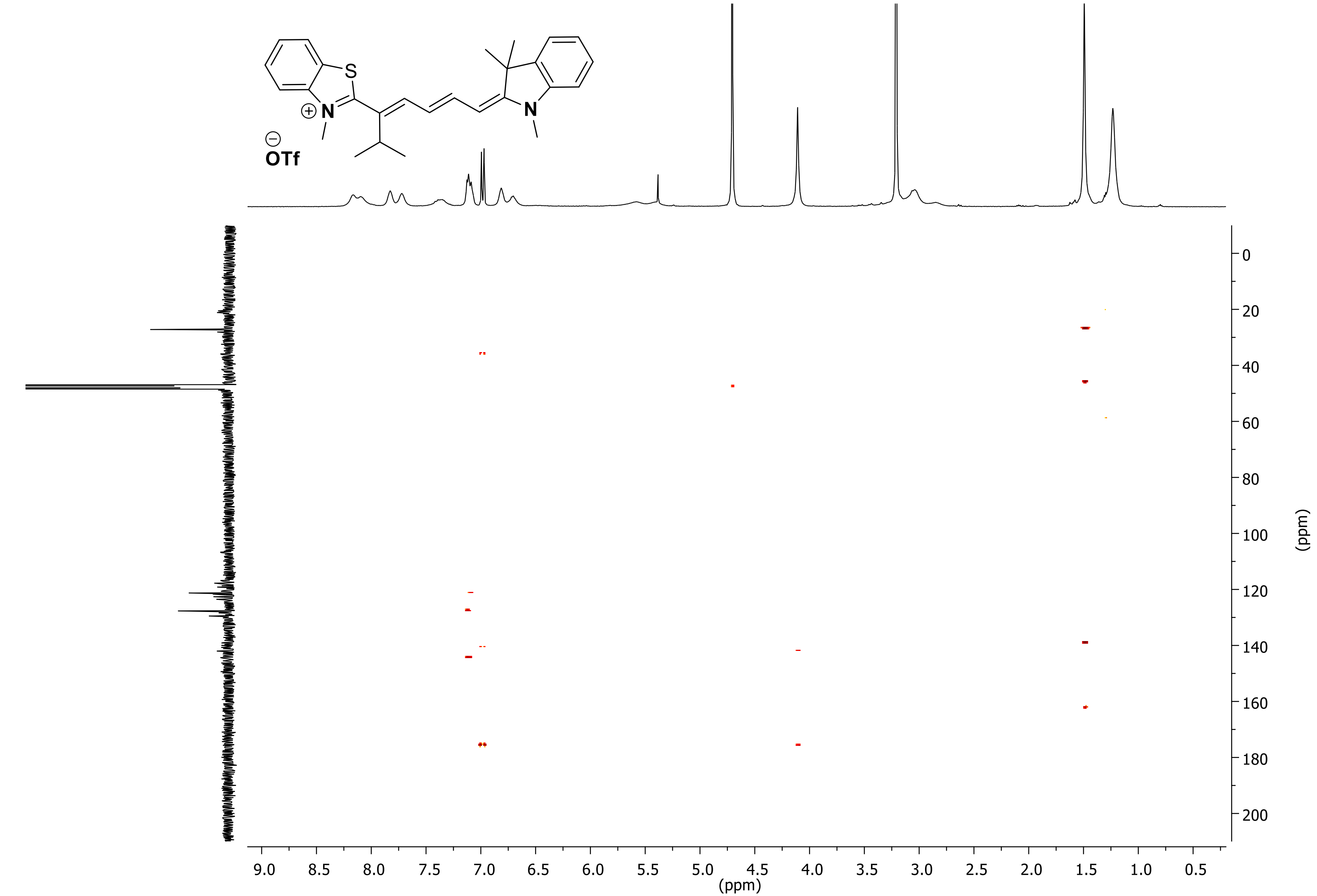

Figure S25. $^{1}H–^{13}C\{^{1}H\}$ gHMBC (500 MHz, d_4–CD_3OD): **6**

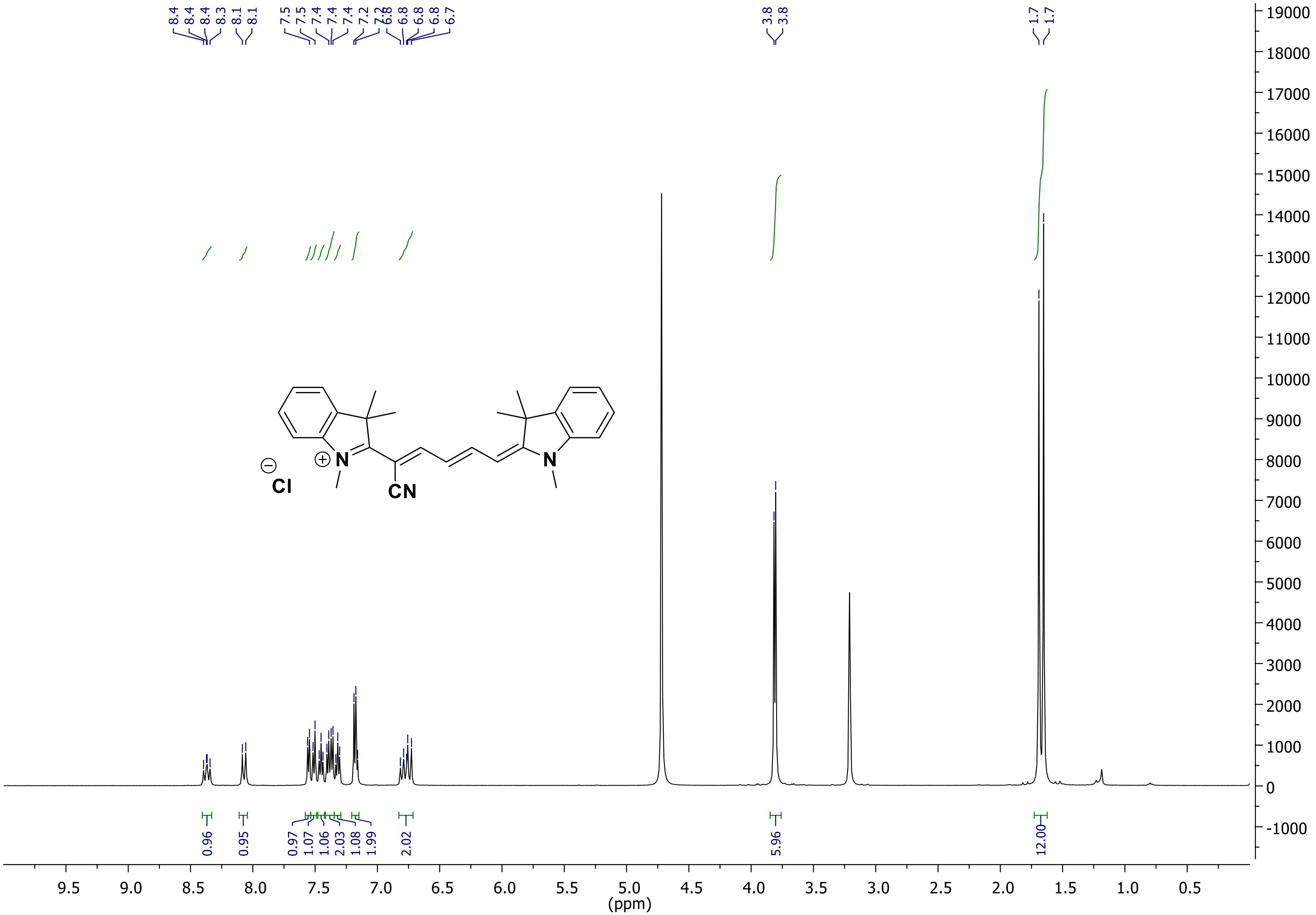

Figure S26. ^{1}H NMR (500 MHz, d_4–CD_3OD): **7**

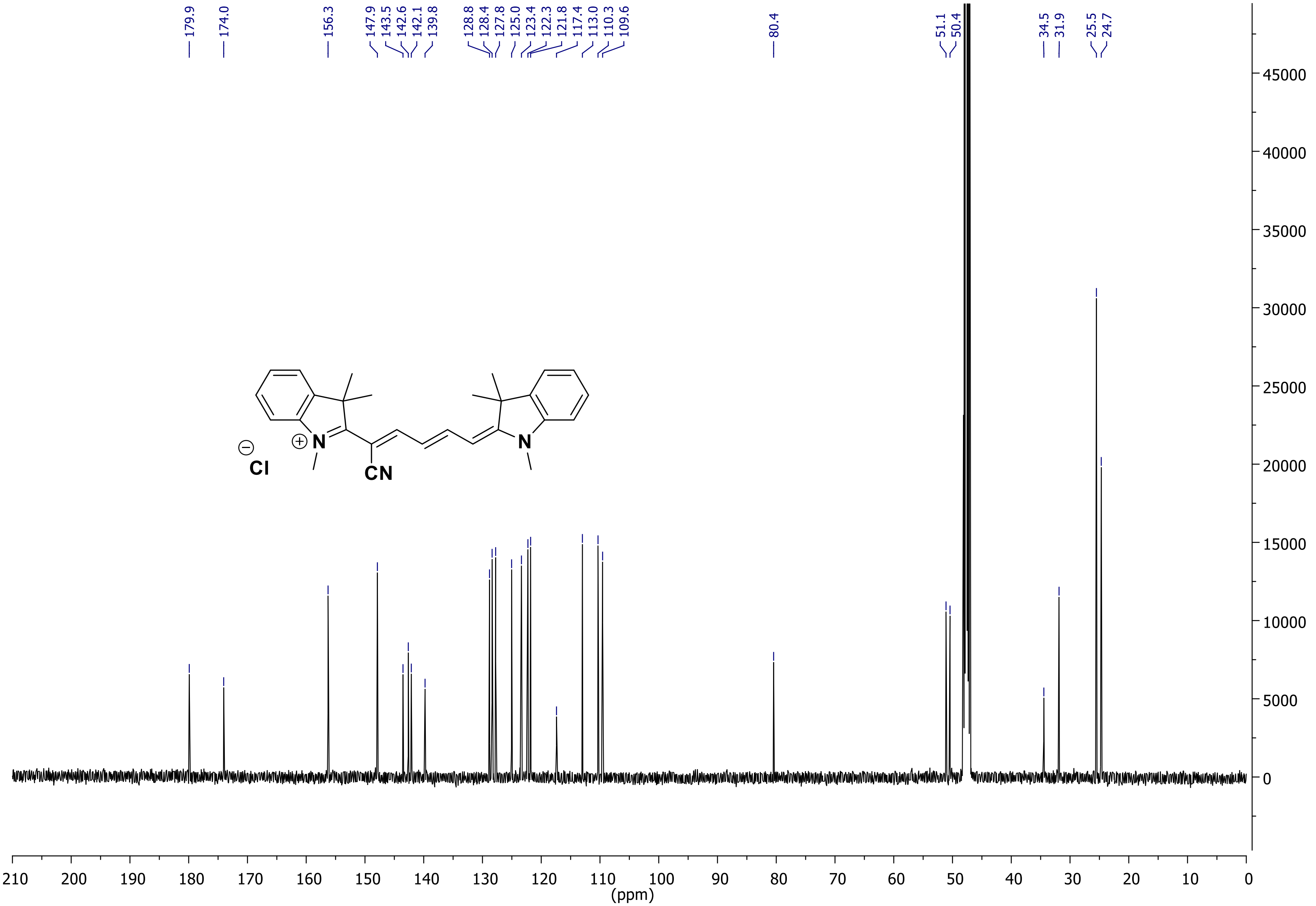

Figure 27. $^{13}C\{^{1}H\}$ NMR (126 MHz, d_4–CD_3OD): **7**

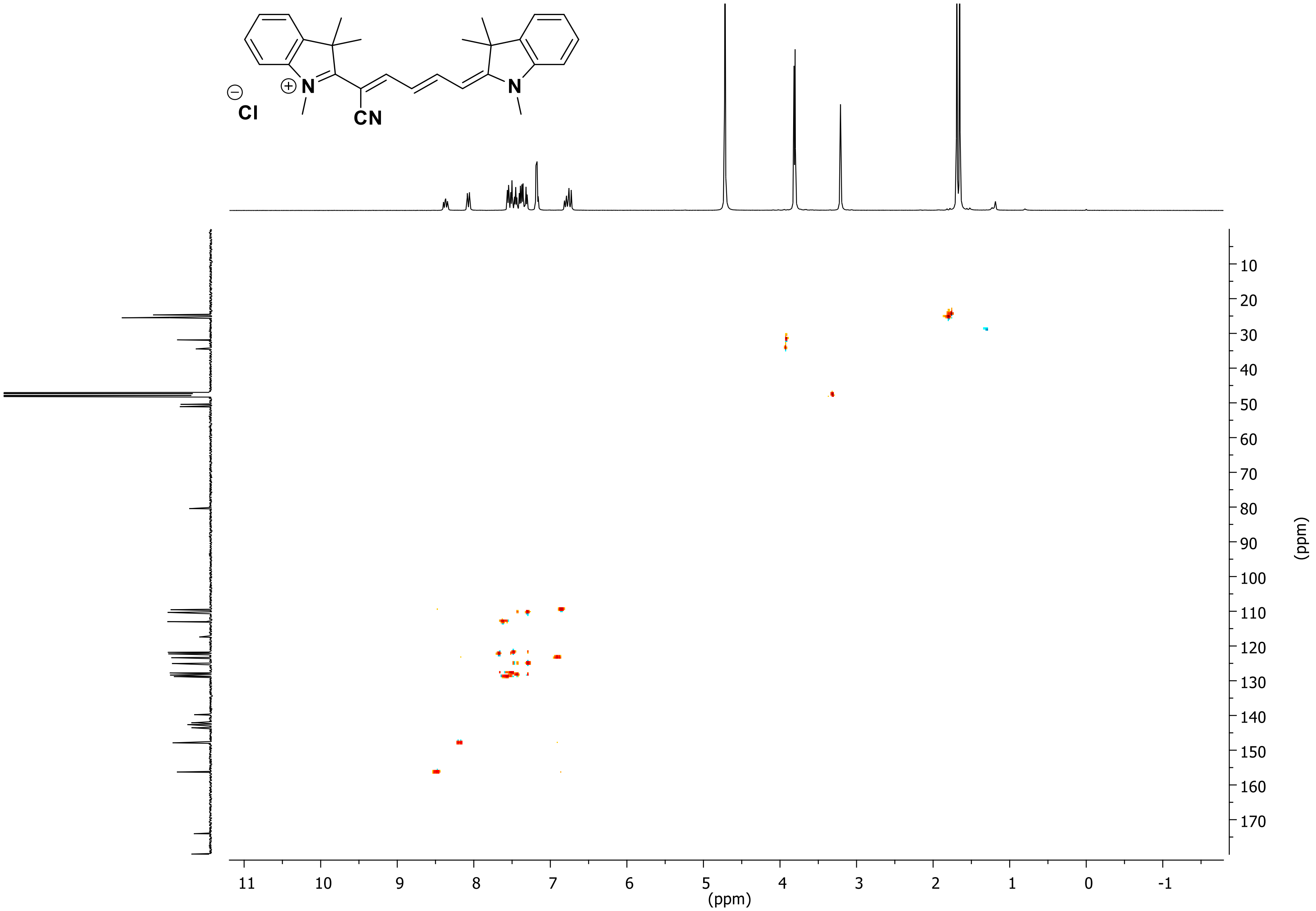

Figure S28. $^{1}H–^{13}C\{^{1}H\}$ gHSQC (500 MHz, d_4–CD_3OD): **7**

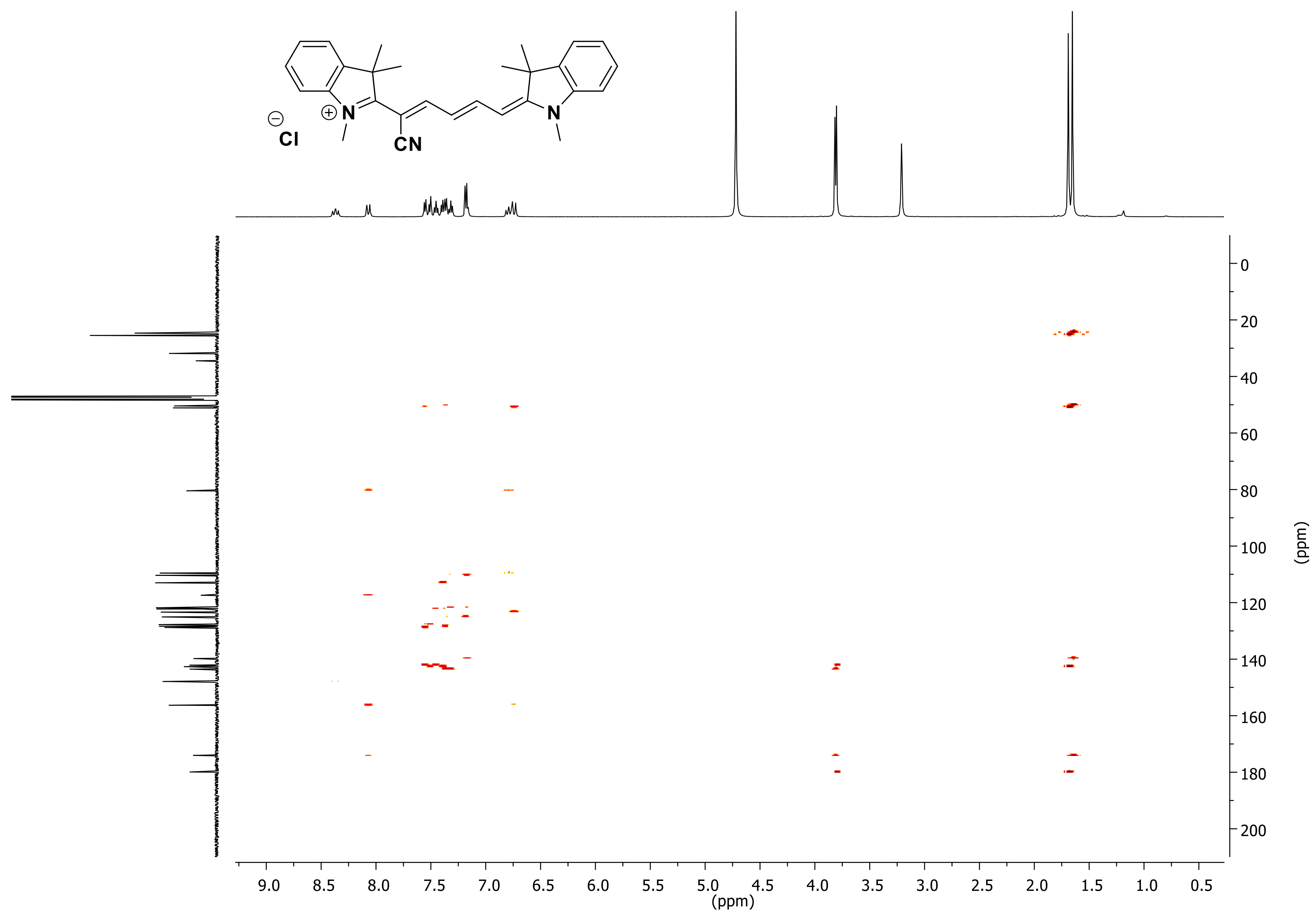

Figure S29. ^{1}H–$^{13}C\{^{1}H\}$ gHMBC (500 MHz, d_4–CD_3OD): **7**

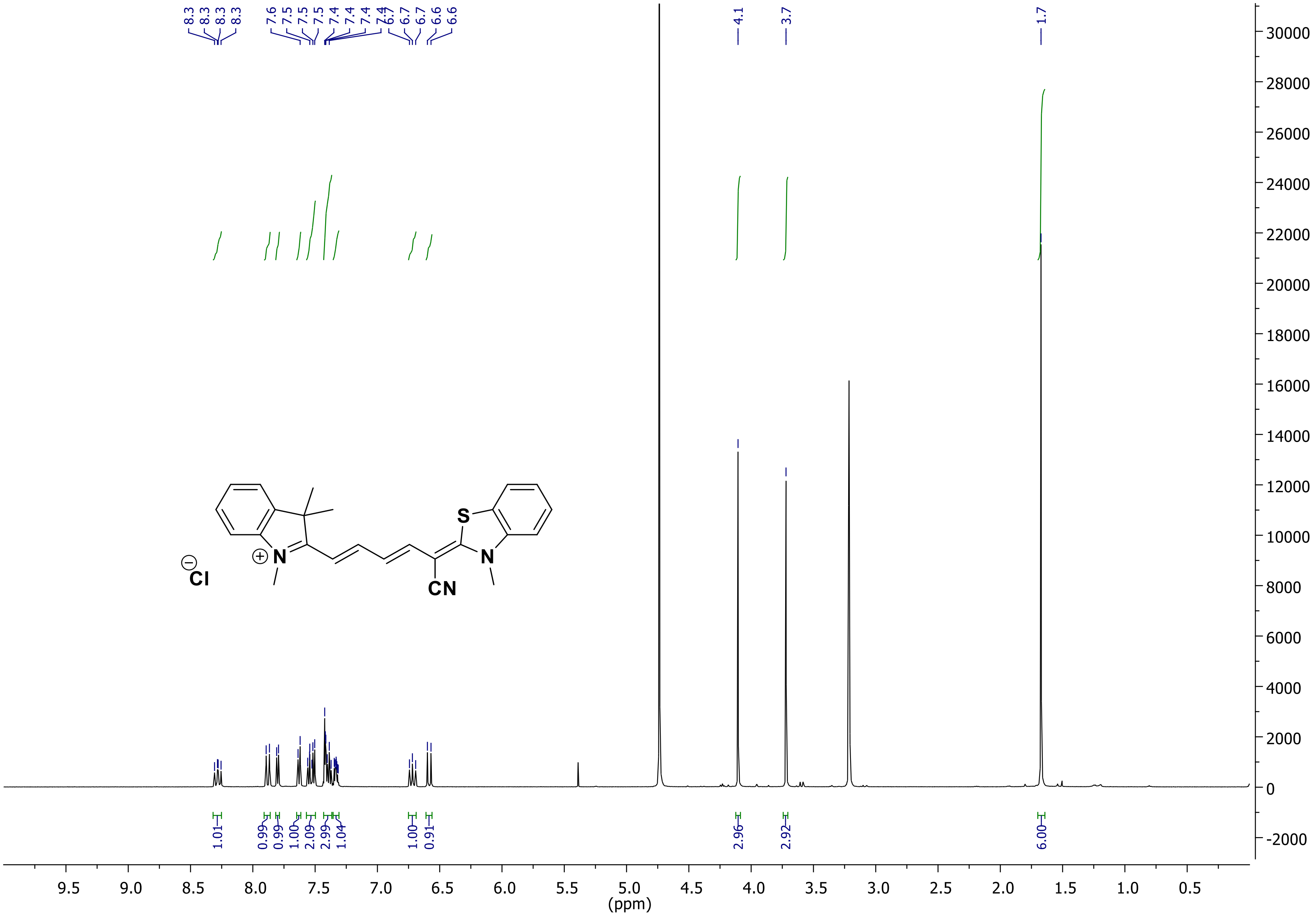

Figure S30. ^{1}H NMR (500 MHz, d_4-CD_3OD): **8**

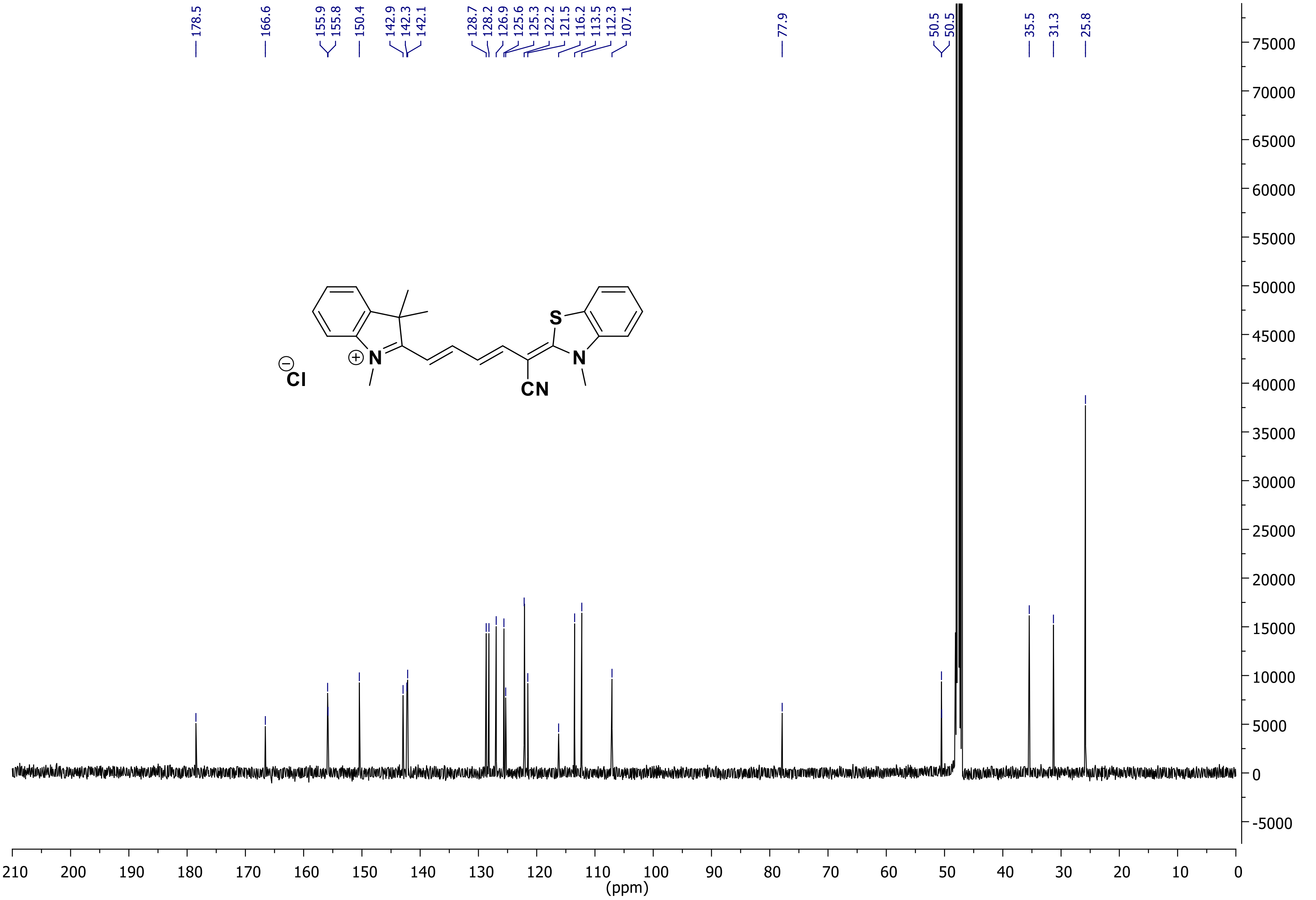

Figure S31. $^{13}C\{^1H\}$ NMR (126 MHz, d_4-CD_3OD): **8**

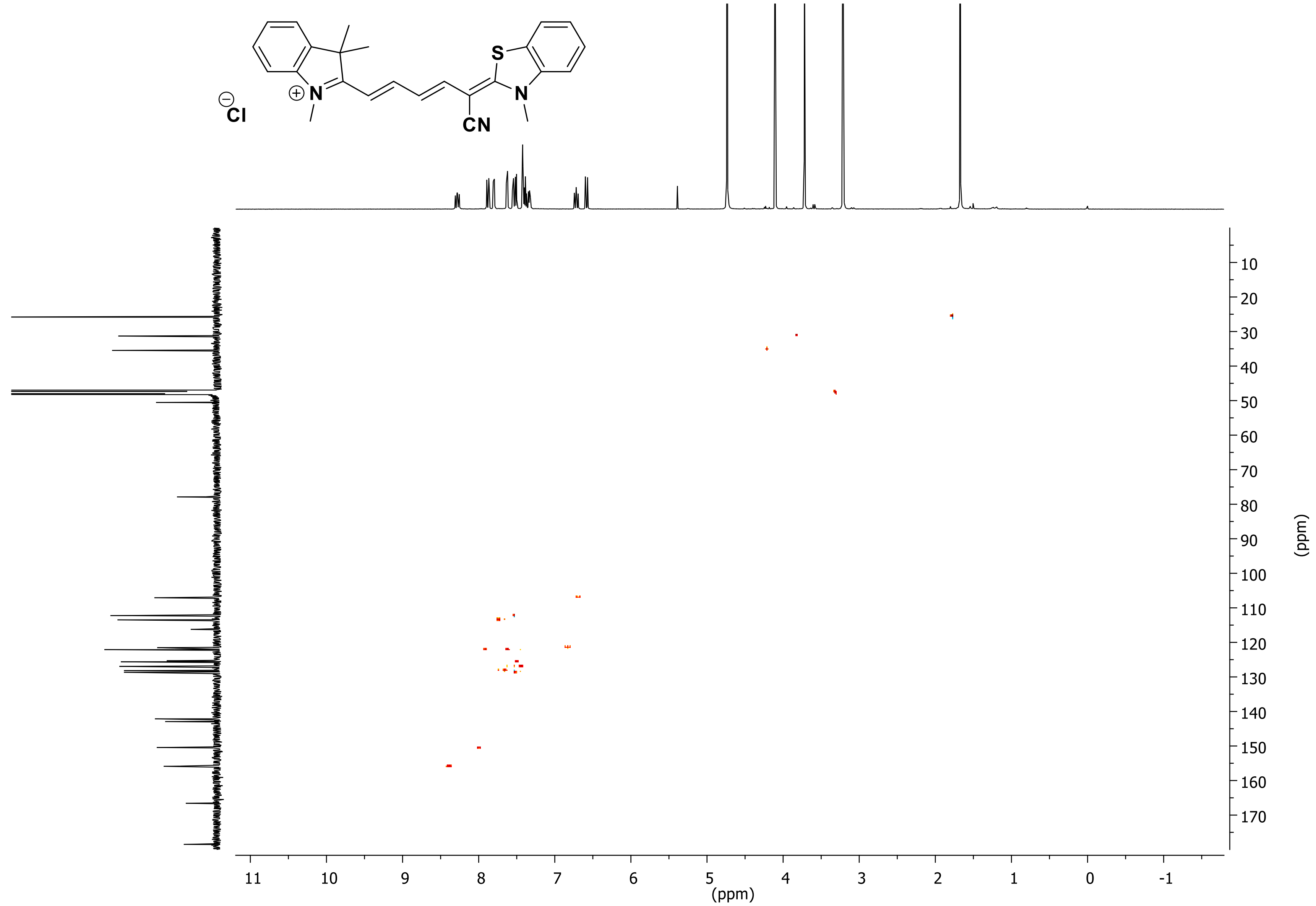

Figure S32. ^{1}H–$^{13}C\{^{1}H\}$ gHSQC (500 MHz, d_4-CD_3OD): **8**

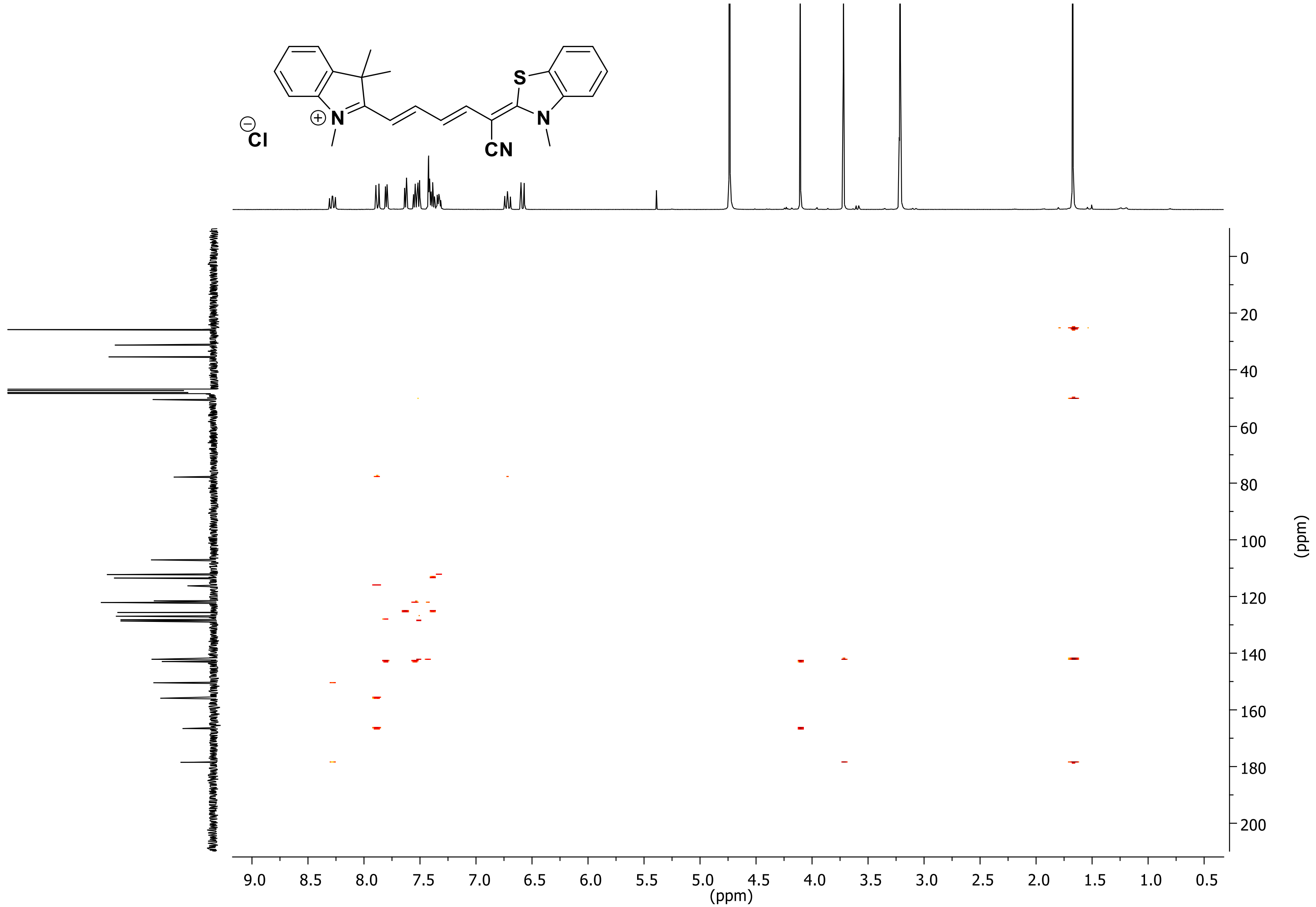

Figure S33. ^1H–$^{13}C\{^1H\}$ gHMBC (500 MHz, d_4-CD_3OD): **8**

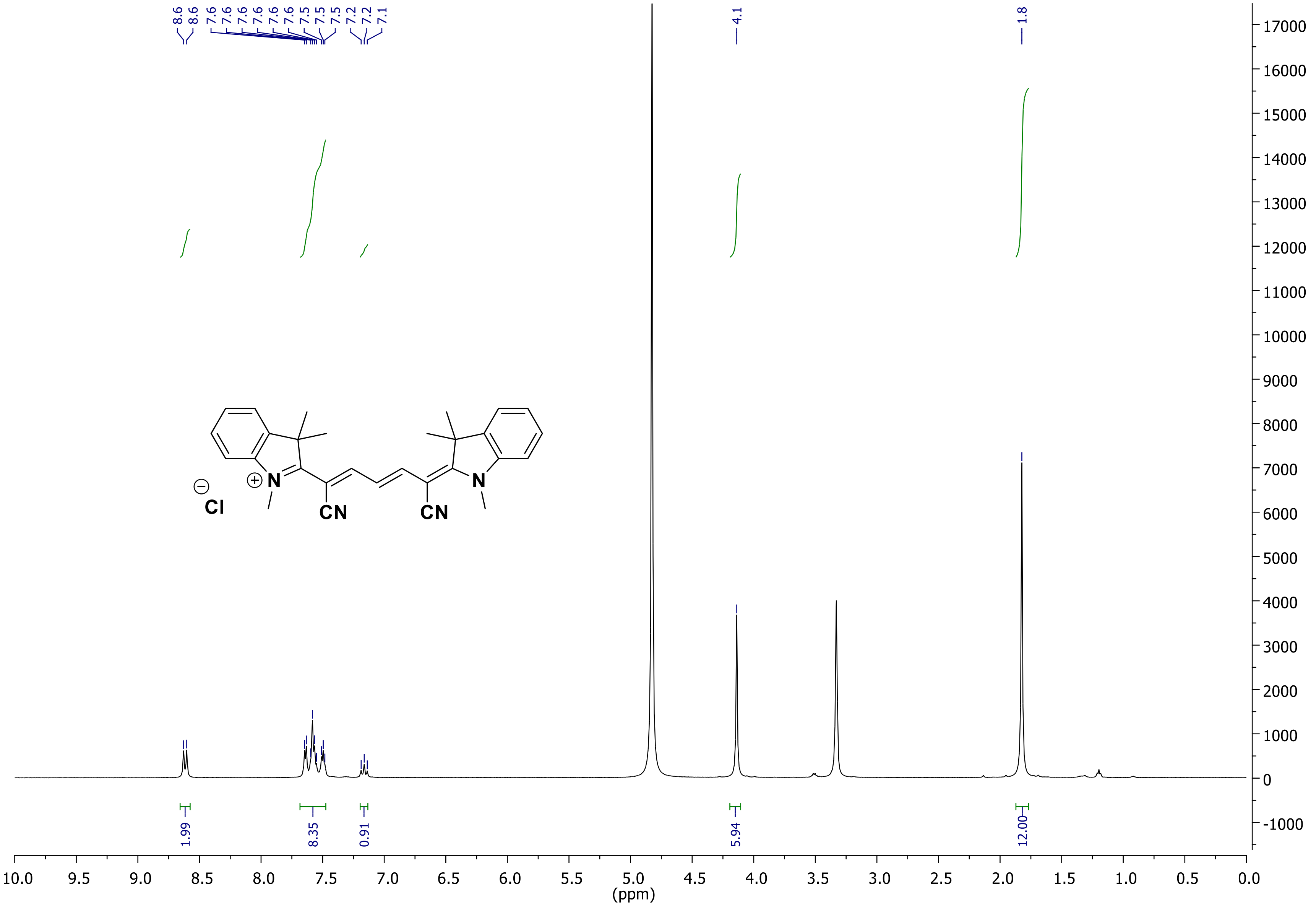

Figure S34. ^{1}H NMR (500 MHz, d_4-CD_3OD): **9**

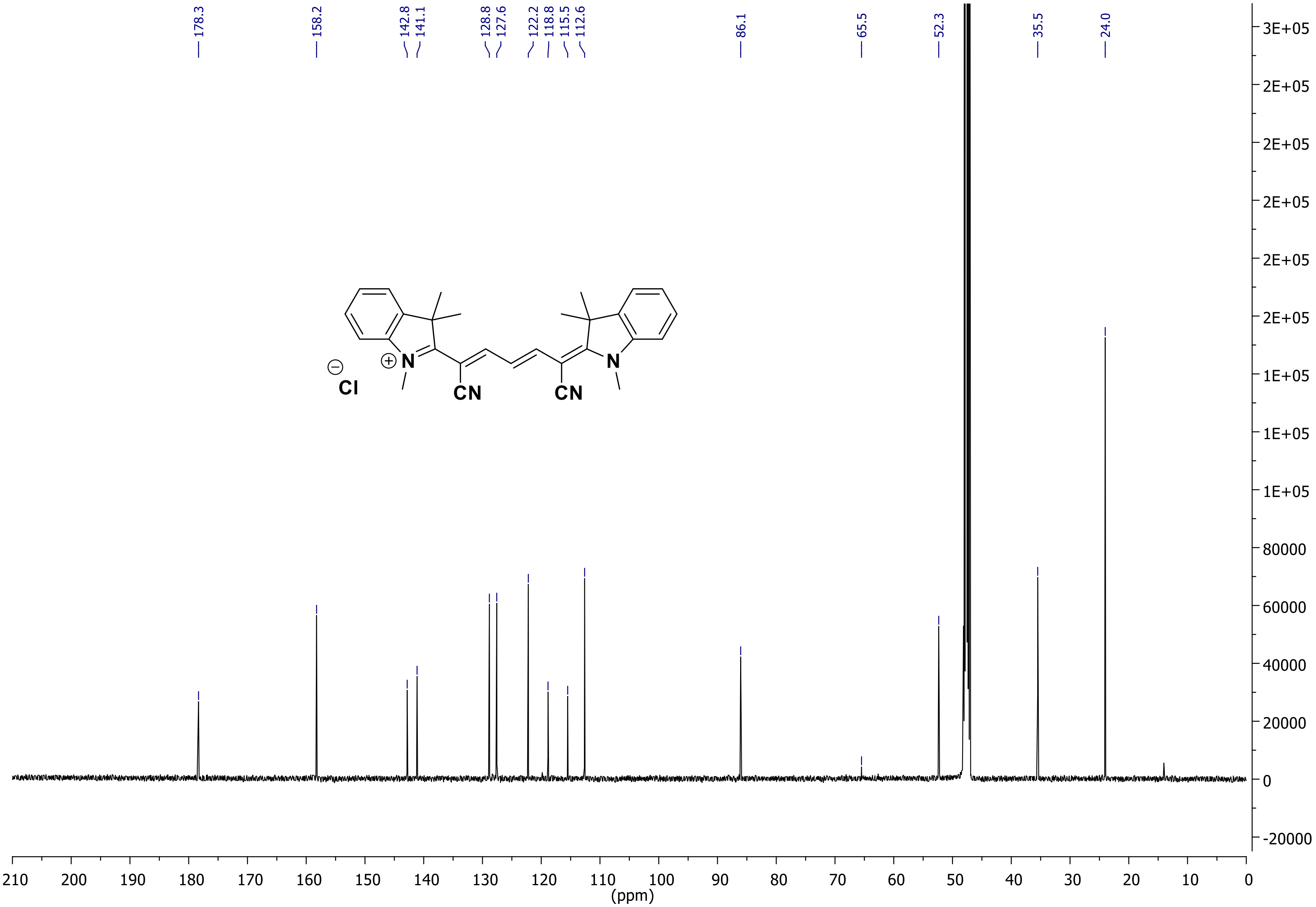

Figure S35. $^{13}C\{^{1}H\}$ (126 MHz, d_4-CD_3OD): **9**

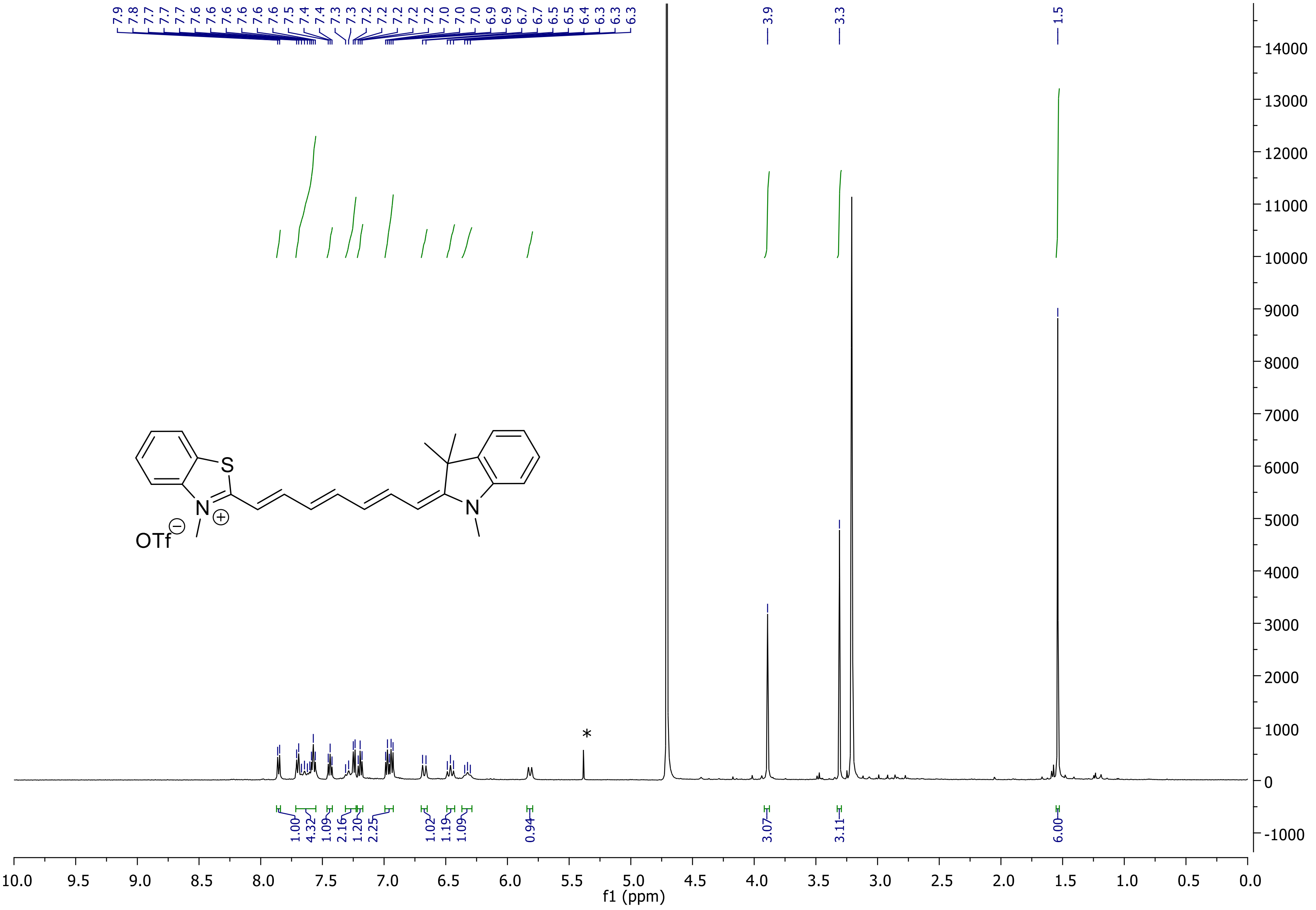

Figure S36. ^{1}H NMR (500 MHz, d_4-CD_3OD): **11** (* dichloromethane)

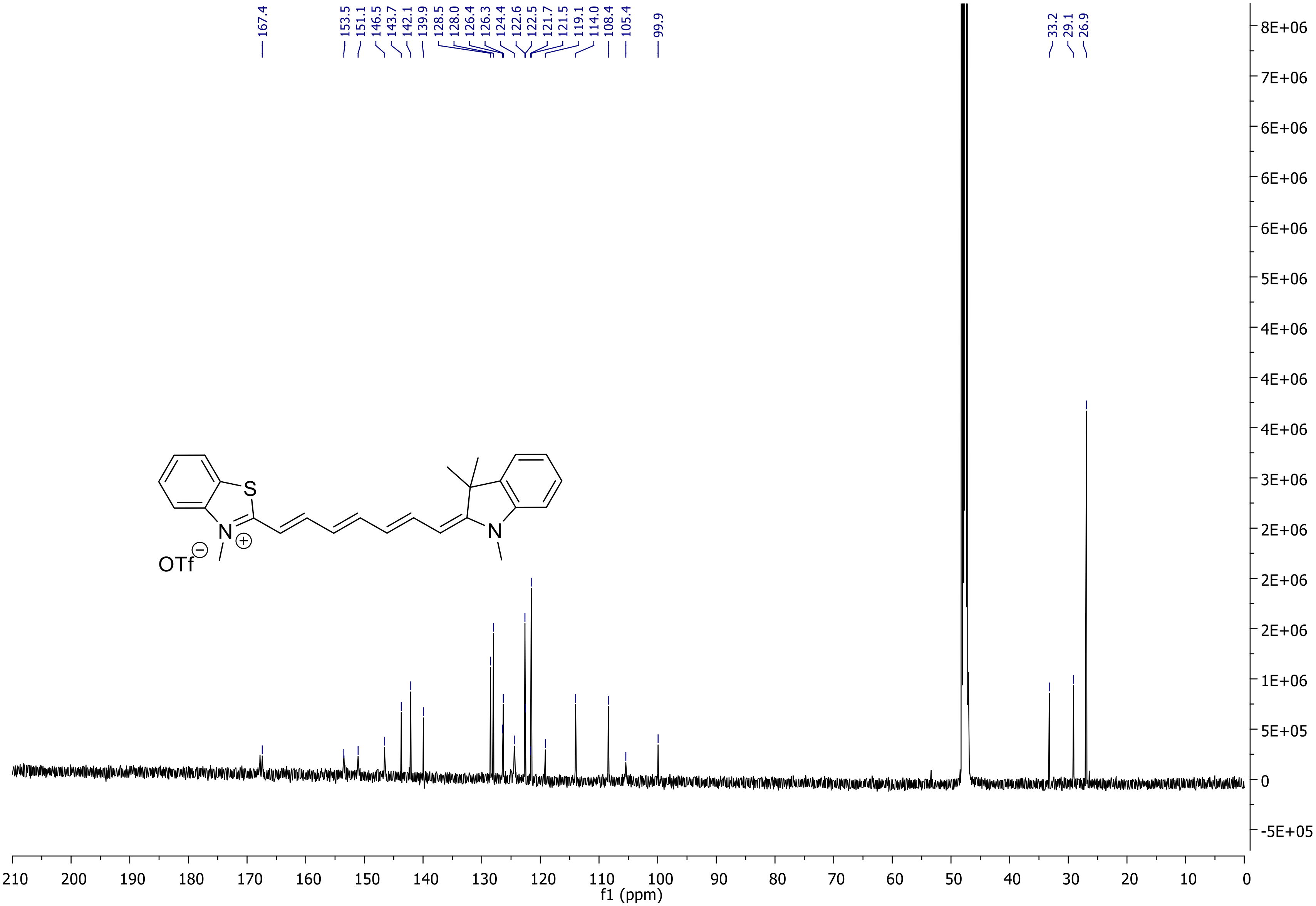

Figure S37. $^{13}C\{^{1}H\}$ NMR (126 MHz, d_4-CD_3OD): **11**

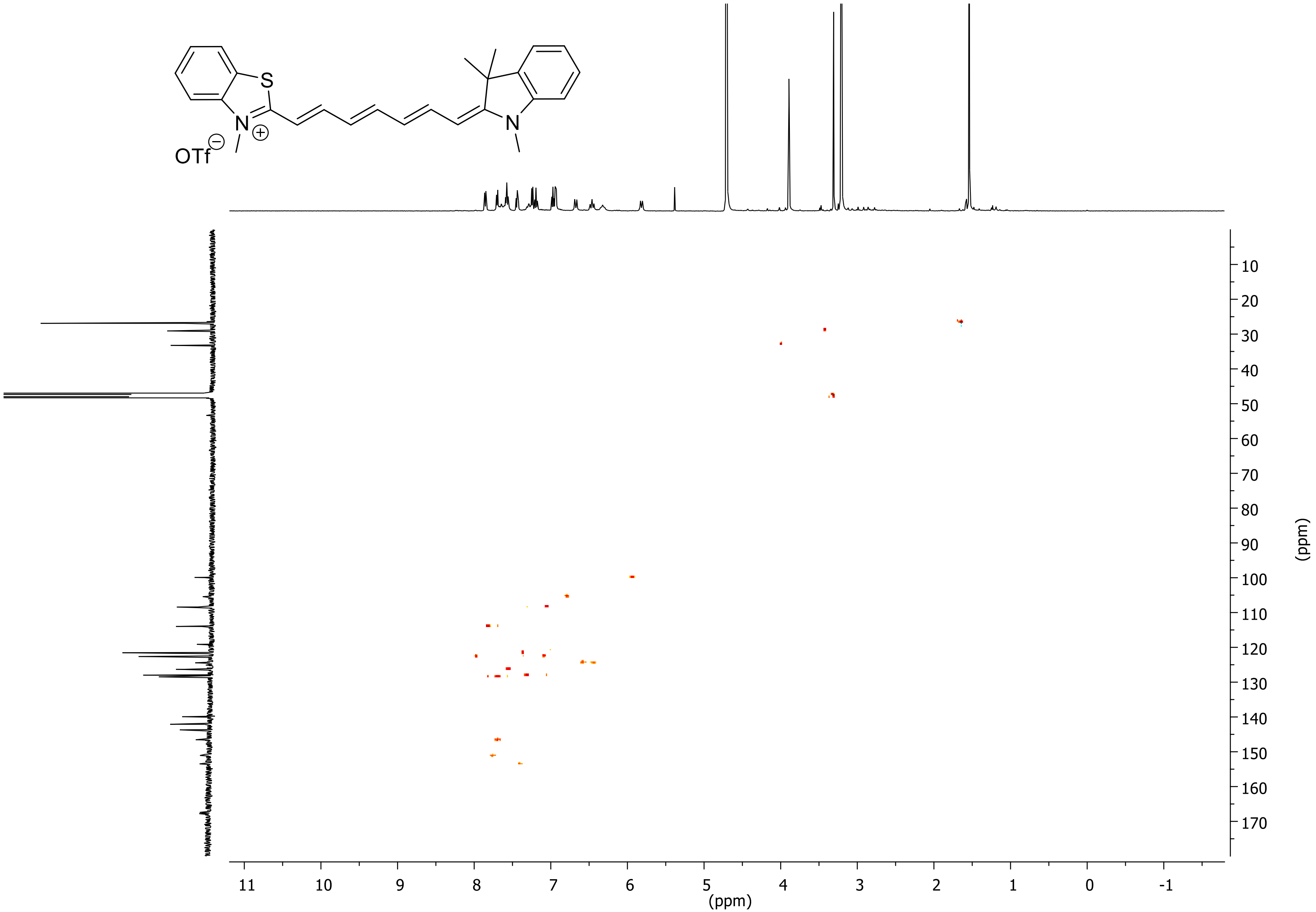

Figure S38. ^{1}H–$^{13}C\{^{1}H\}$ gHSQC (500 MHz, d_4-CD_3OD): **11**

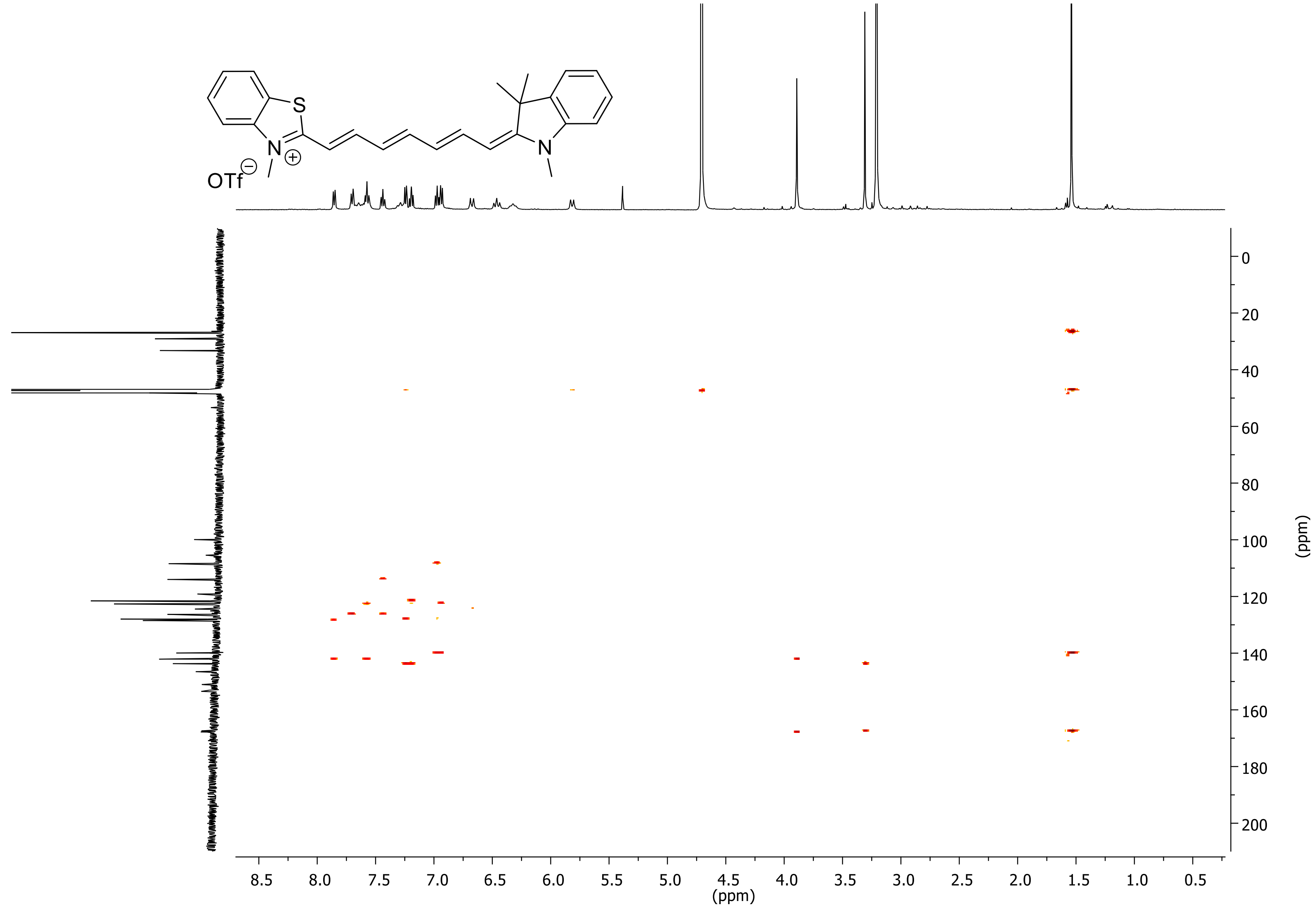

Figure S39. ^{1}H–$^{13}C\{^{1}H\}$ gHMBC (500 MHz, d_4-CD_3OD): **11**

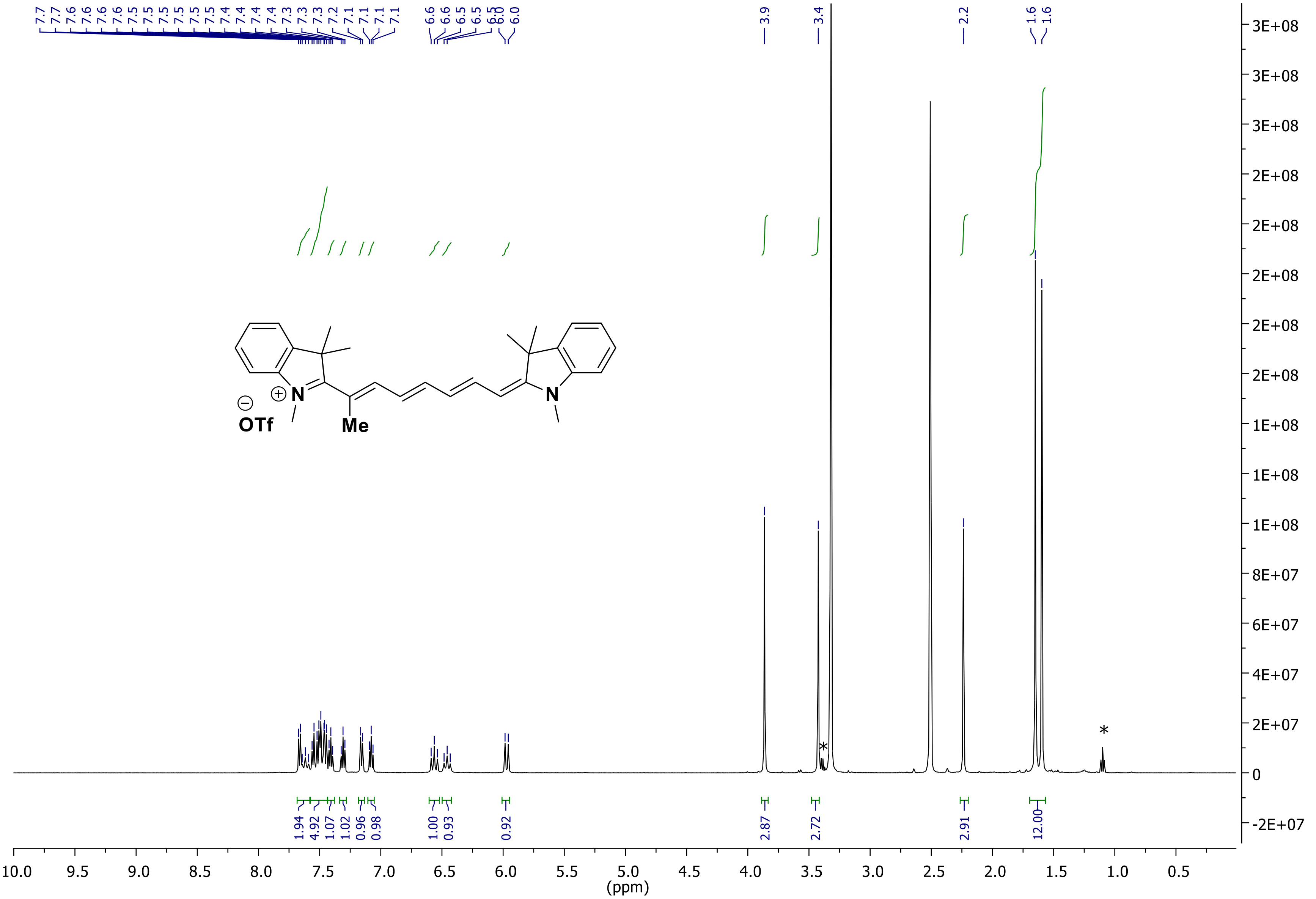

Figure S40. ^{1}H NMR (500 MHz, d_6-DMSO): **12** (*ethyl acetate (EtOAc))

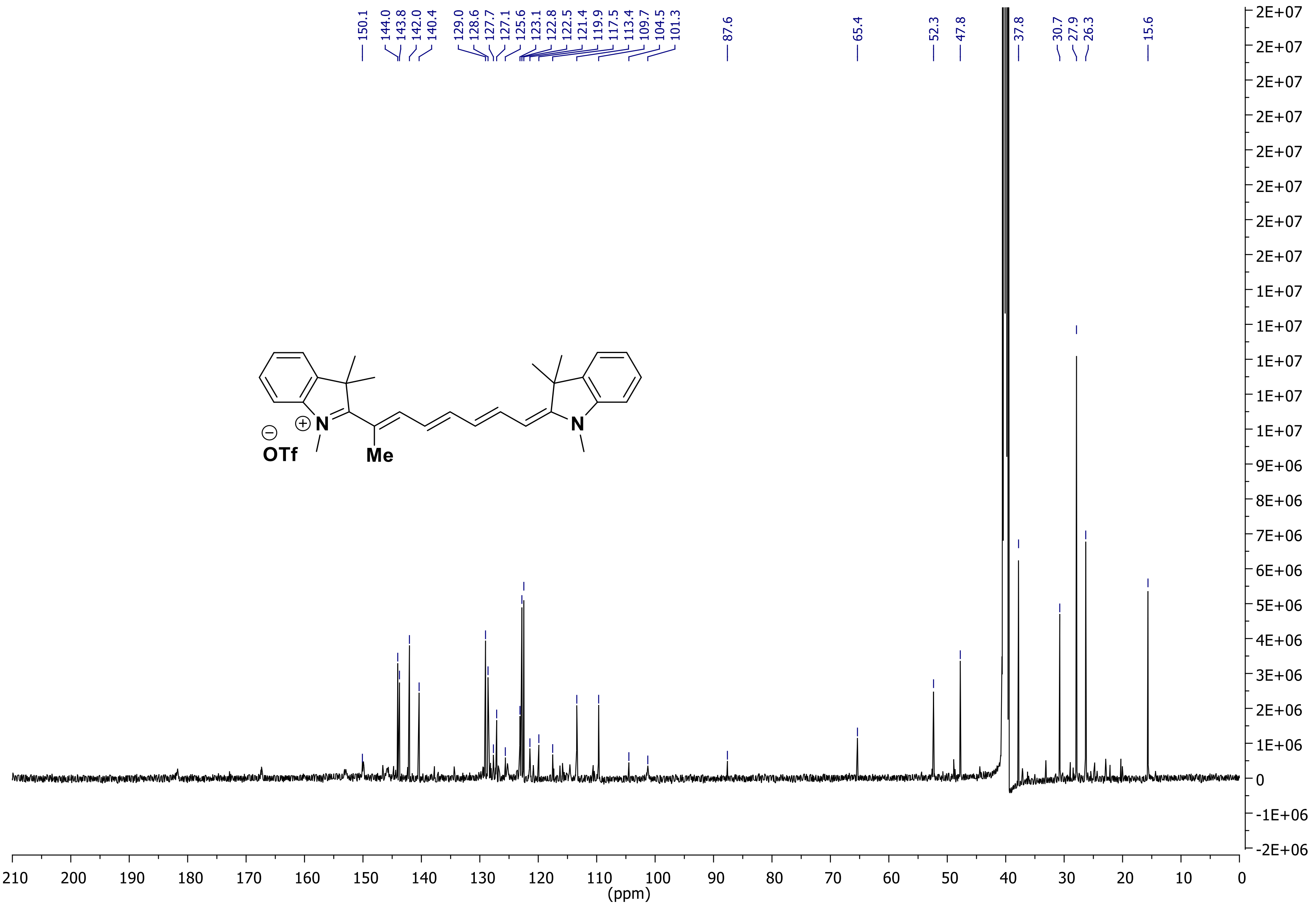

Figure S41. $^{13}C\{^1H\}$ NMR (126 MHz, d_6-DMSO): **12** (*ethyl acetate)

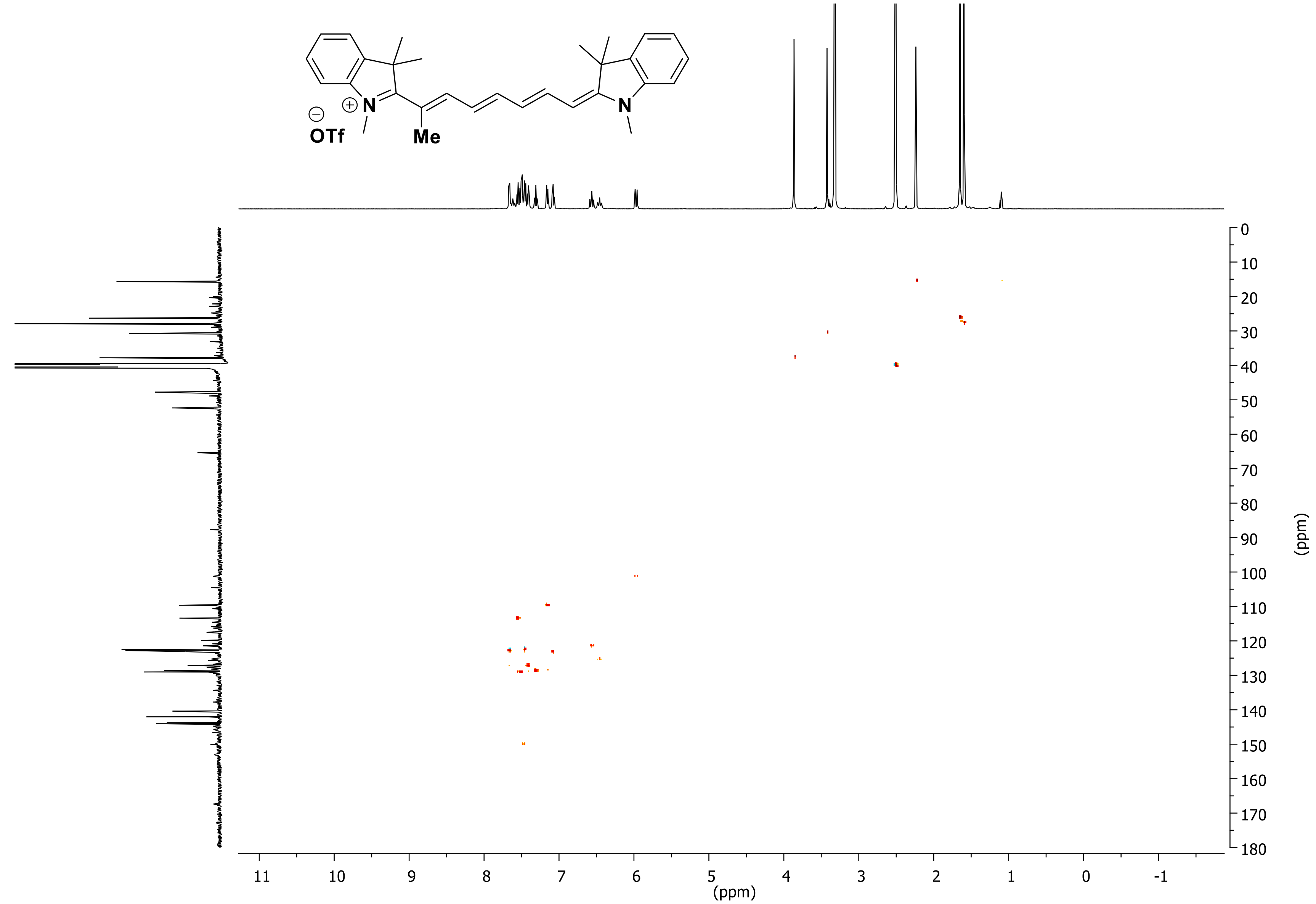

Figure S42. ^{1}H–$^{13}C\{^{1}H\}$ gHSQC (500 MHz, d_6–DMSO): **12**

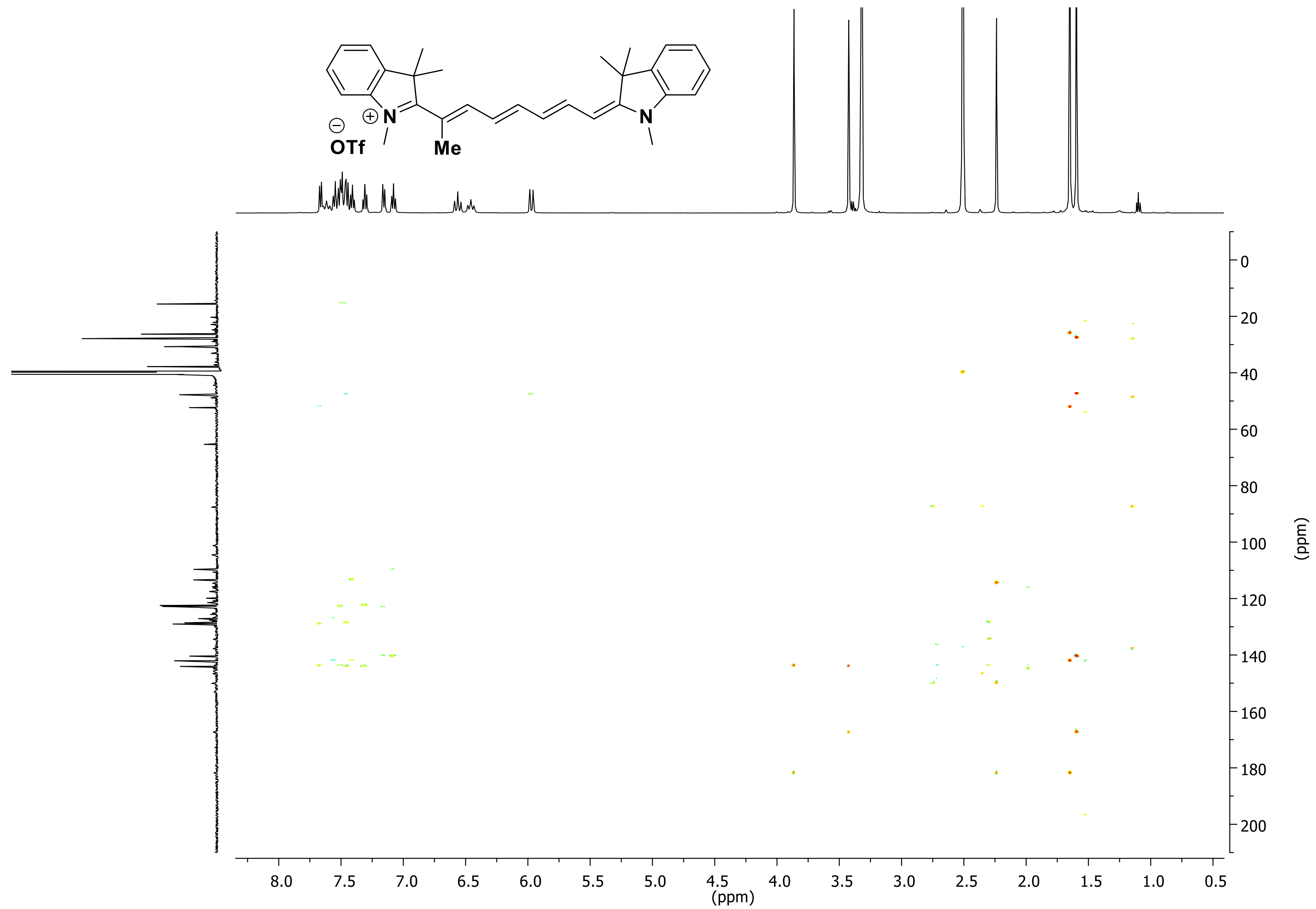

Figure S43. ^{1}H–$^{13}C\{^{1}H\}$ gHMBC (500 MHz, d_6–DMSO): **12**

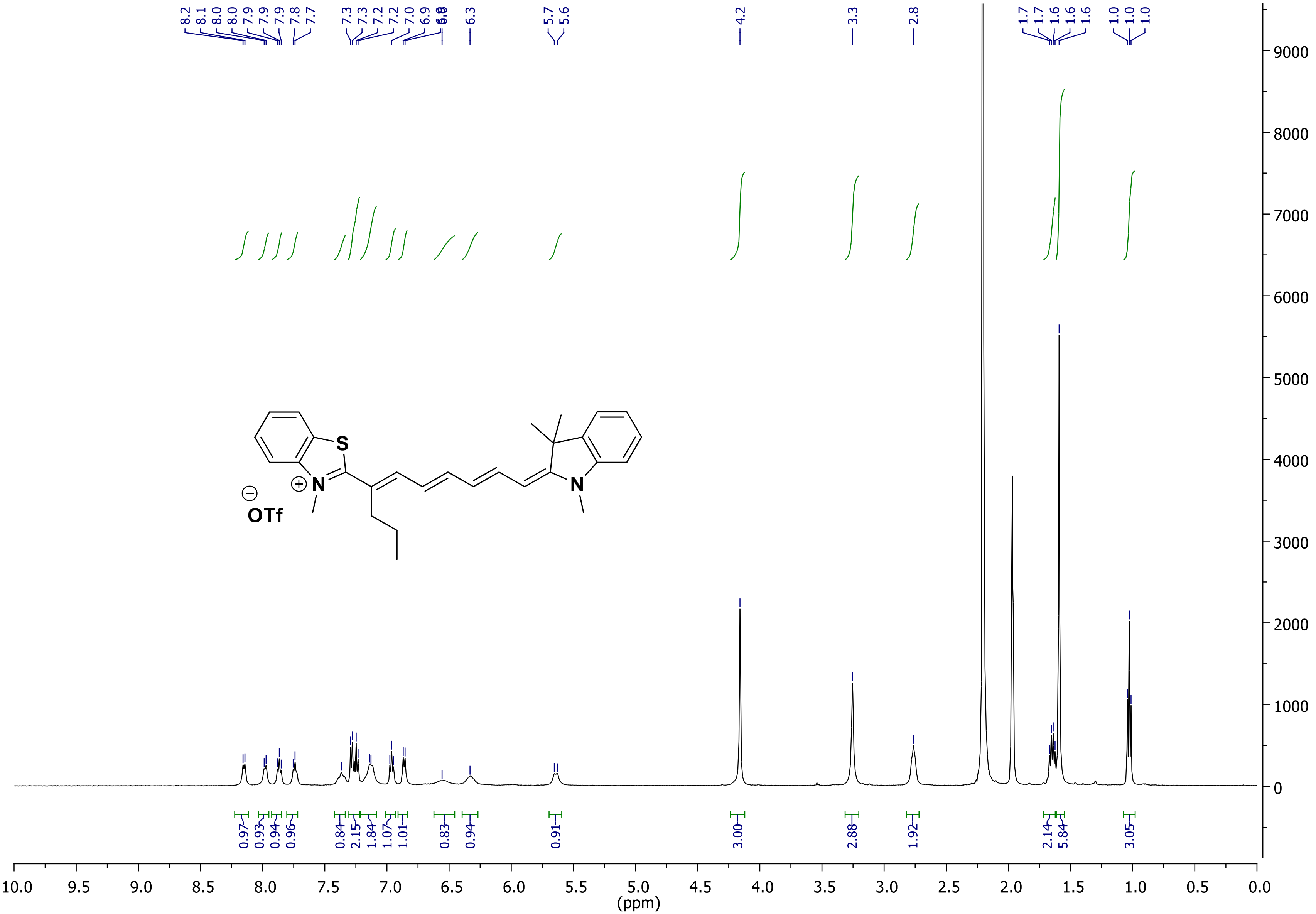

Figure S44. ^{1}H NMR (500 MHz, d_3-CD_3CN): **13**

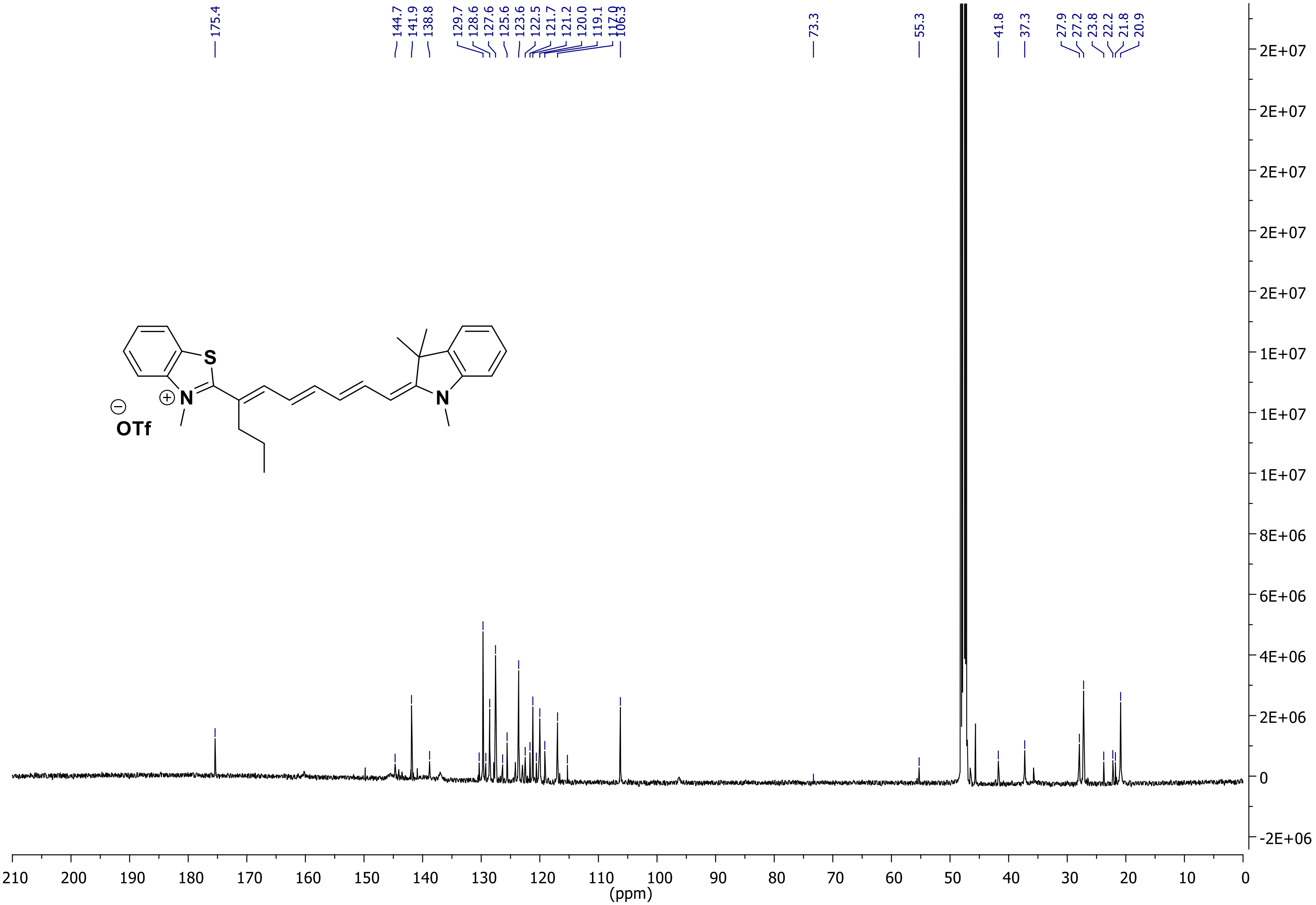

Figure S45. $^{13}C\{^{1}H\}$ NMR (126 MHz, d_3-CD_3CN): **13**

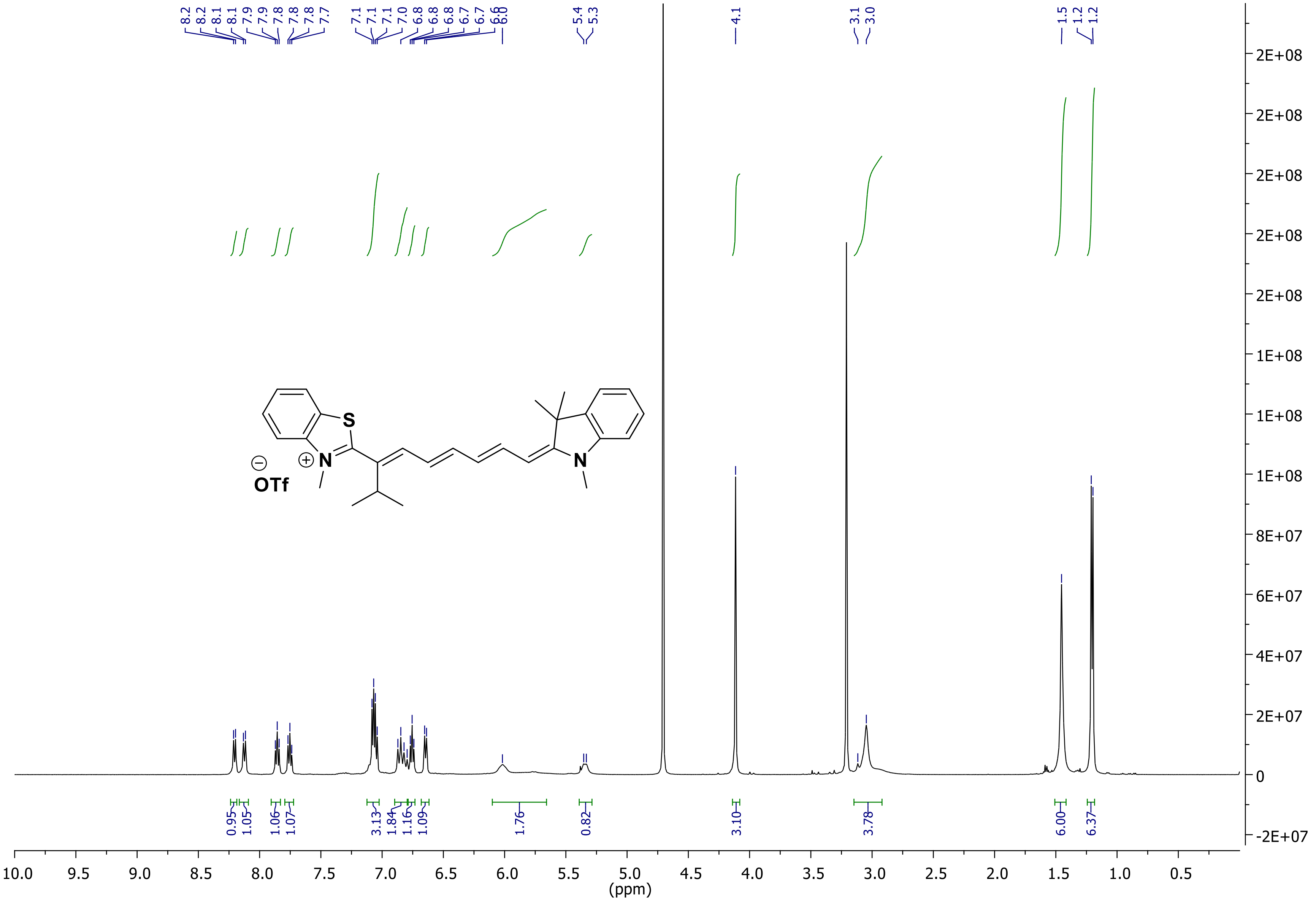

Figure S46. ^{1}H NMR (500 MHz, d_4-CD_3OD): **14**

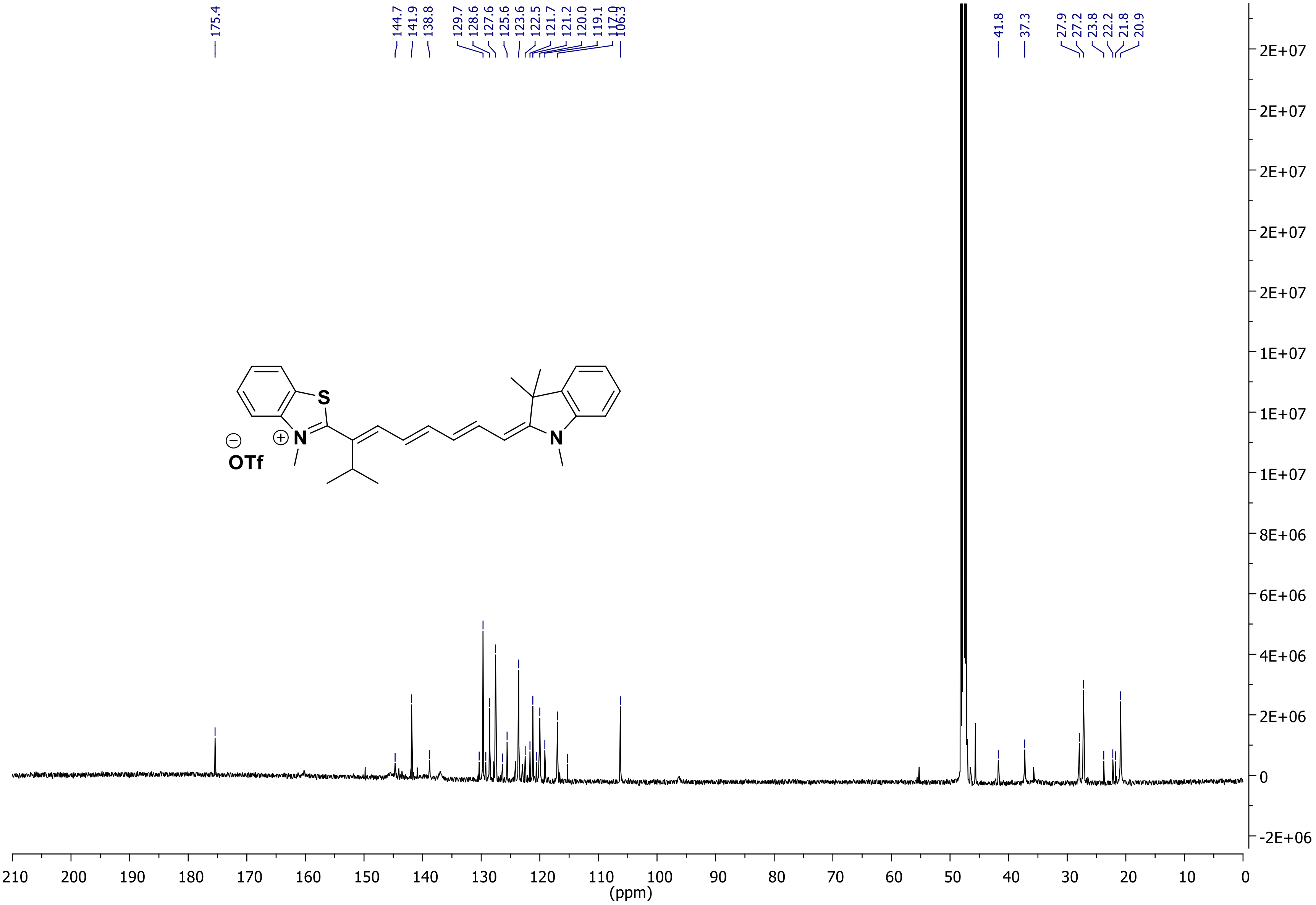

Figure S47. $^{13}C\{^1H\}$ NMR (126 MHz, d_4-CD_3OD): **14**

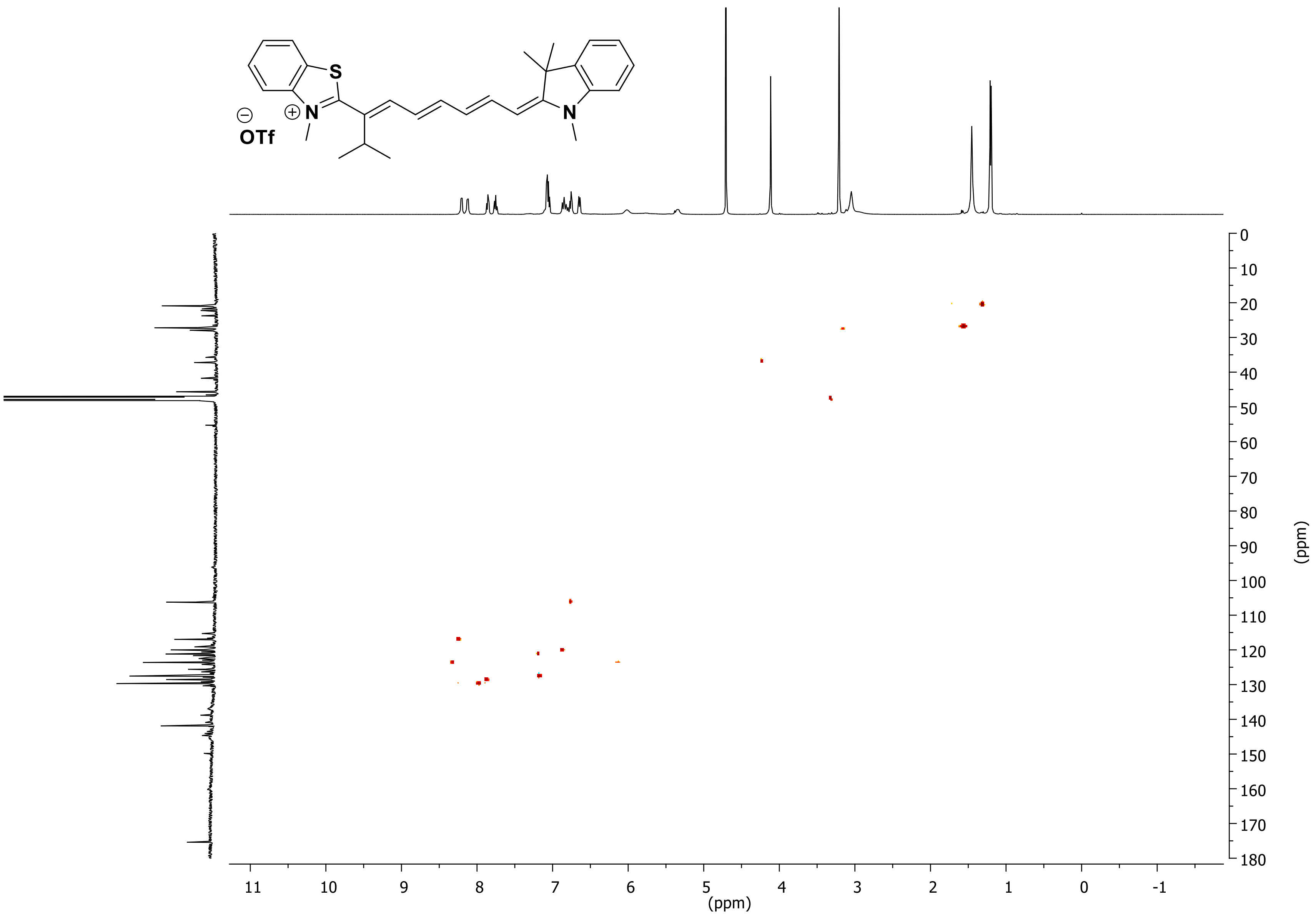

Figure S48. ^{1}H–$^{13}C\{^{1}H\}$ gHSQC (500 MHz, d_4-CD_3OD): **14**

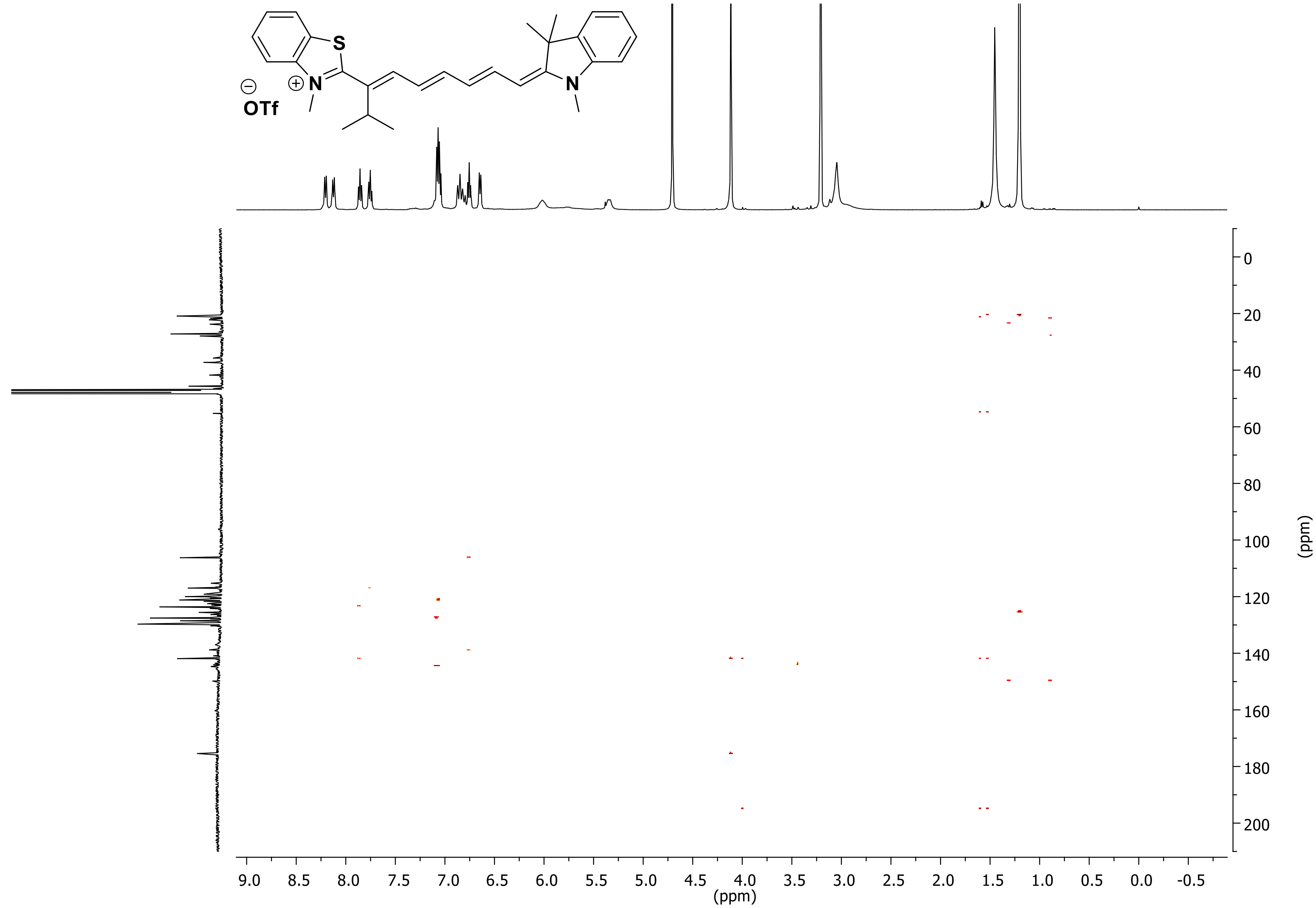

Figure S49. ^{1}H-$^{13}C\{^{1}H\}$ gHMBC (500 MHz, d_4-CD_3OD): **14**

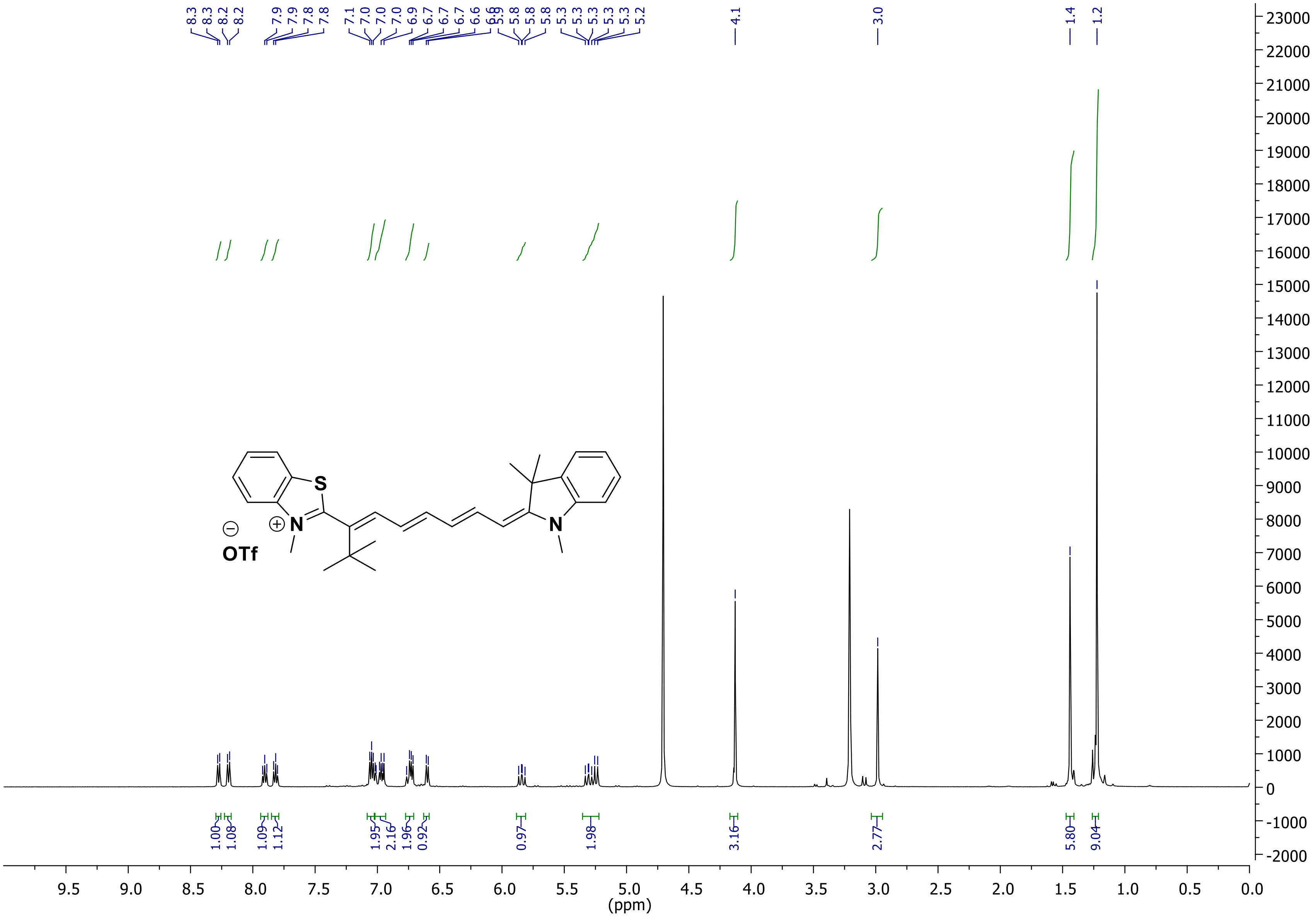

Figure S50. ^{1}H NMR (500 MHz, d_4-CD_3OD): **15**

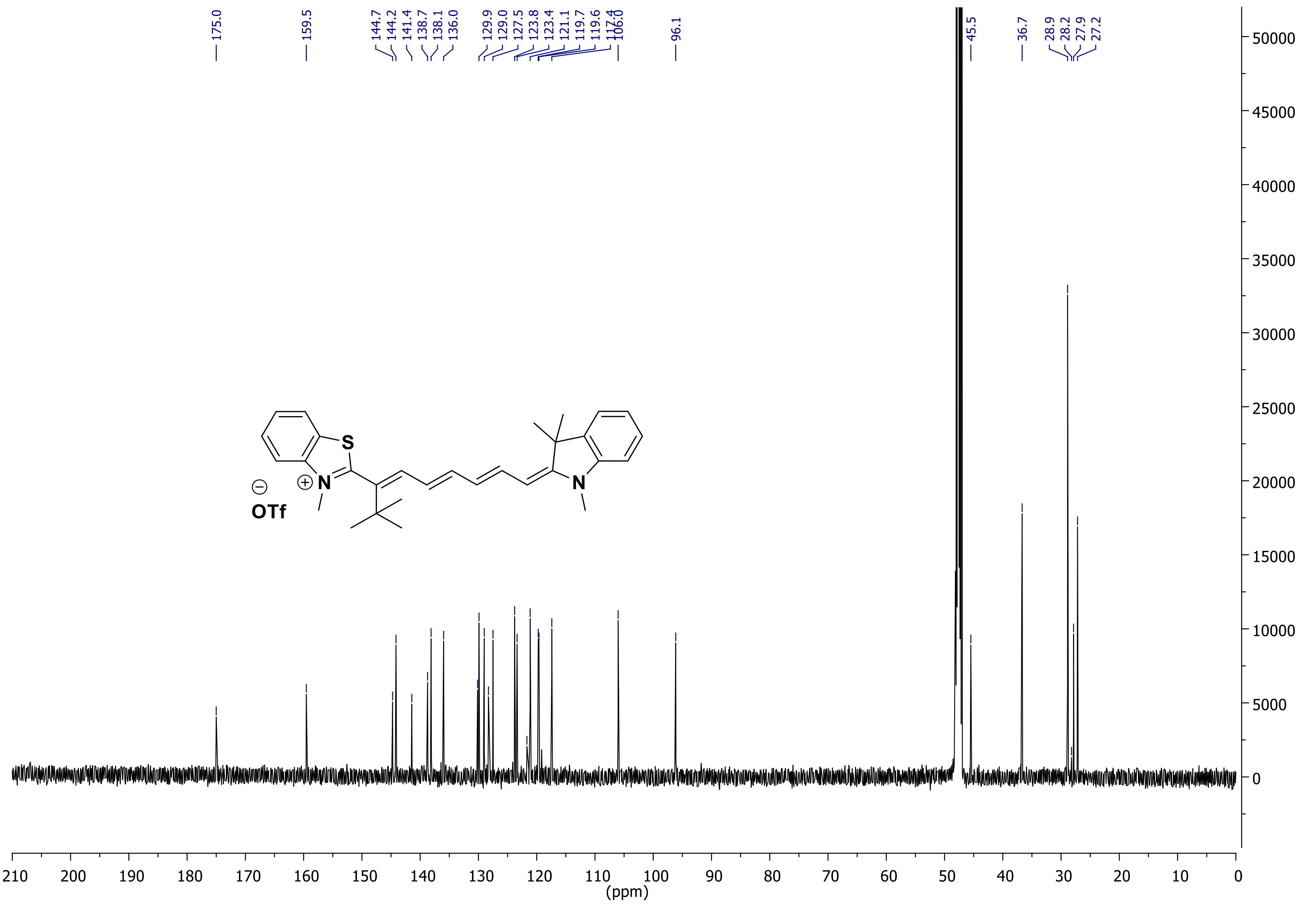

Figure S51. $^{13}C\{^{1}H\}$ NMR (126 MHz, d_4-CD_3OD): **15**

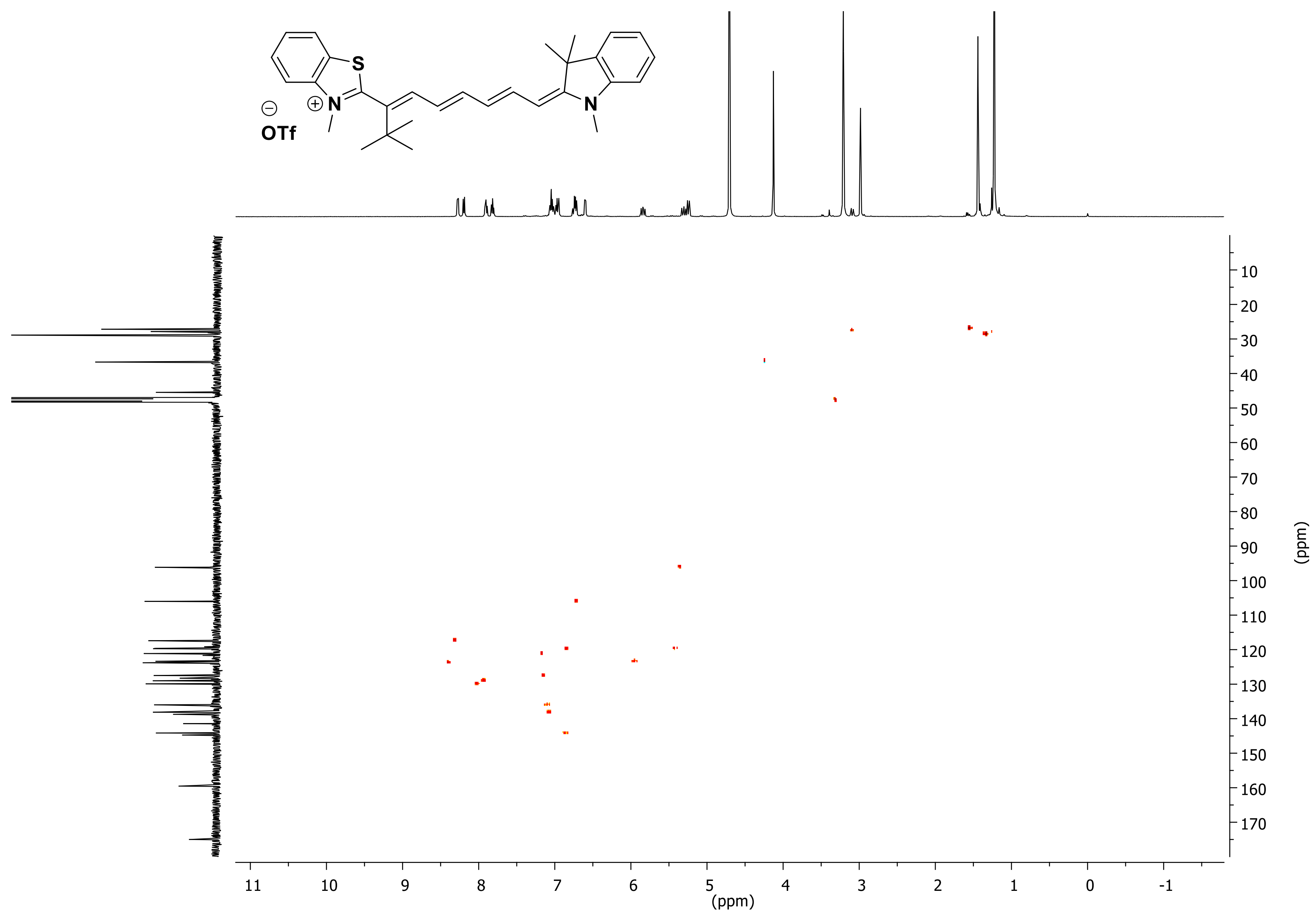

Figure S52. ^{1}H–$^{13}C\{^{1}H\}$ gHSQC (500 MHz, d_4-CD_3OD): **15**

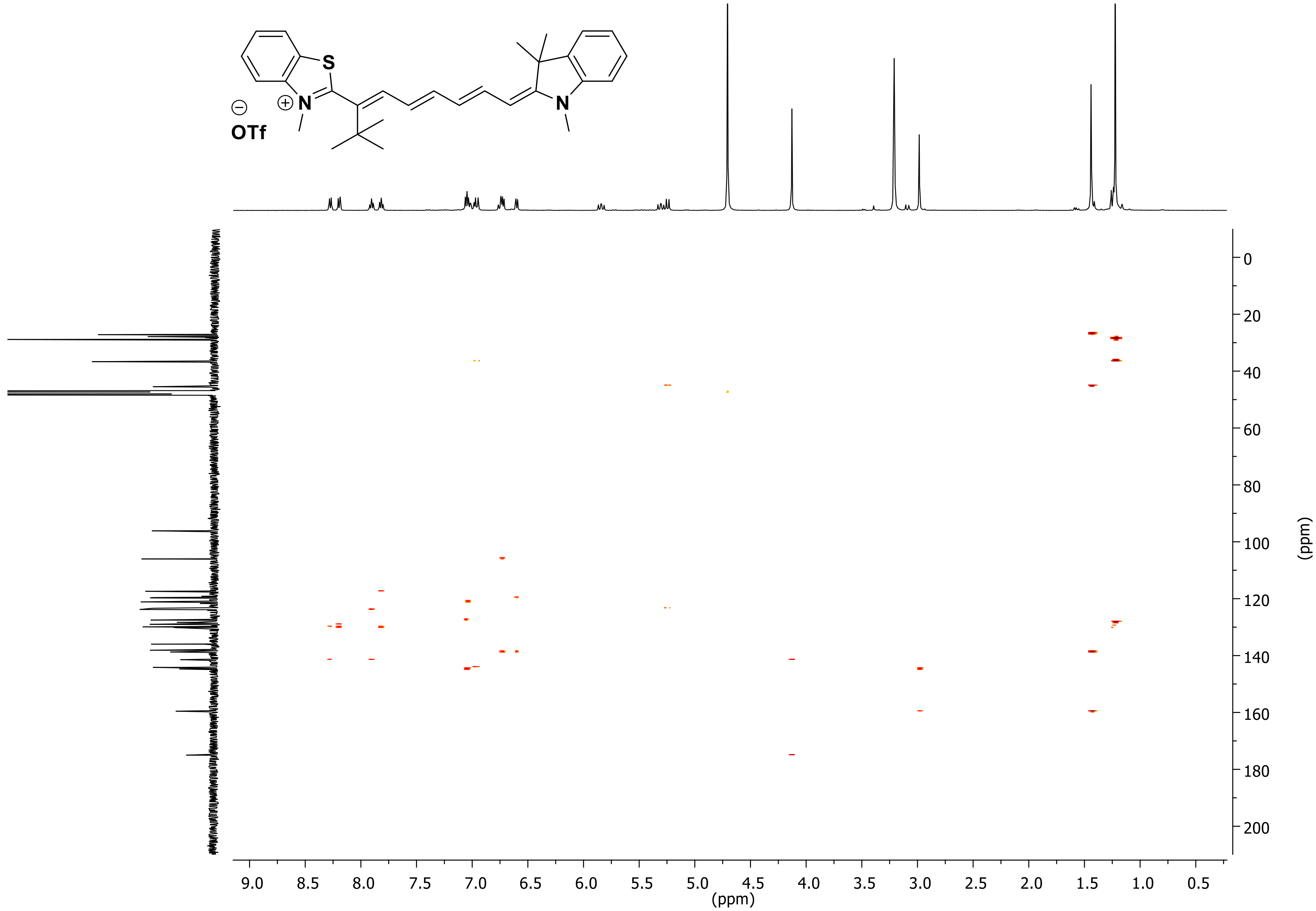

Figure S53. ^{1}H-$^{13}C\{^{1}H\}$ gHMBC (500 MHz, d_4-CD_3OD): **15**

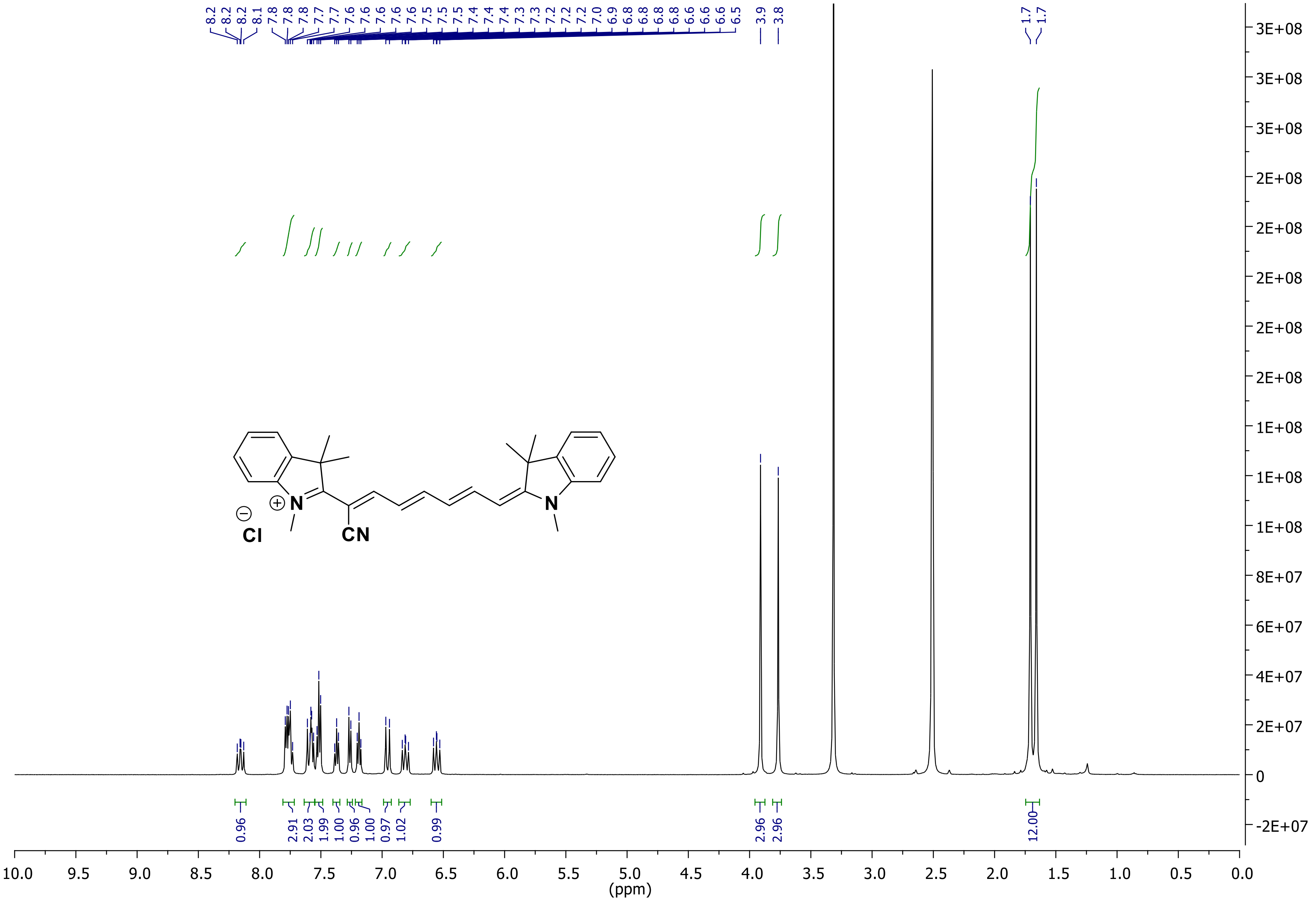

Figure S54. ^{1}H NMR (500 MHz, d_6-DMSO): **16**

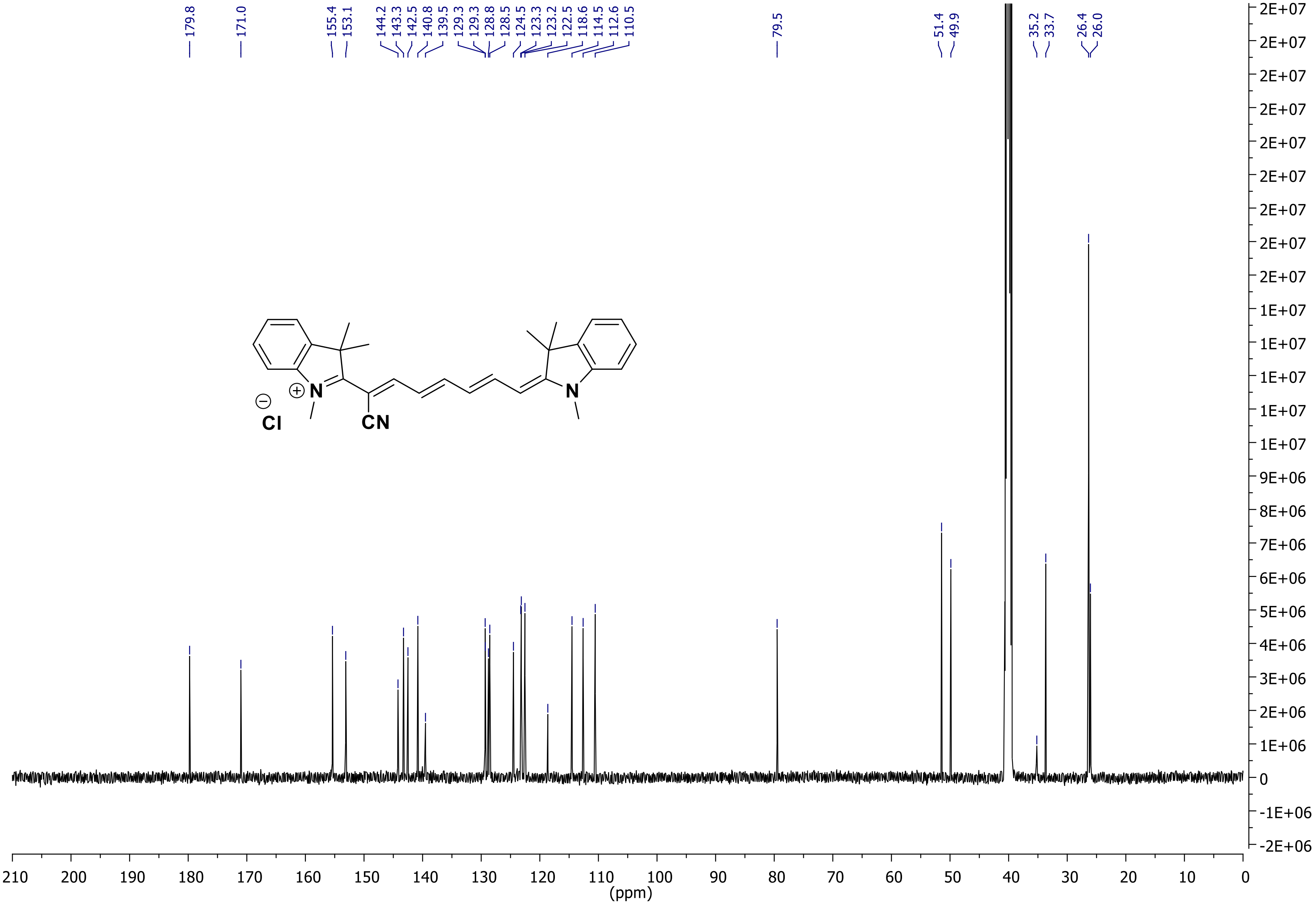

Figure S55. $^{13}C\{^{1}H\}$ NMR (126 MHz, d_6-DMSO): **16**

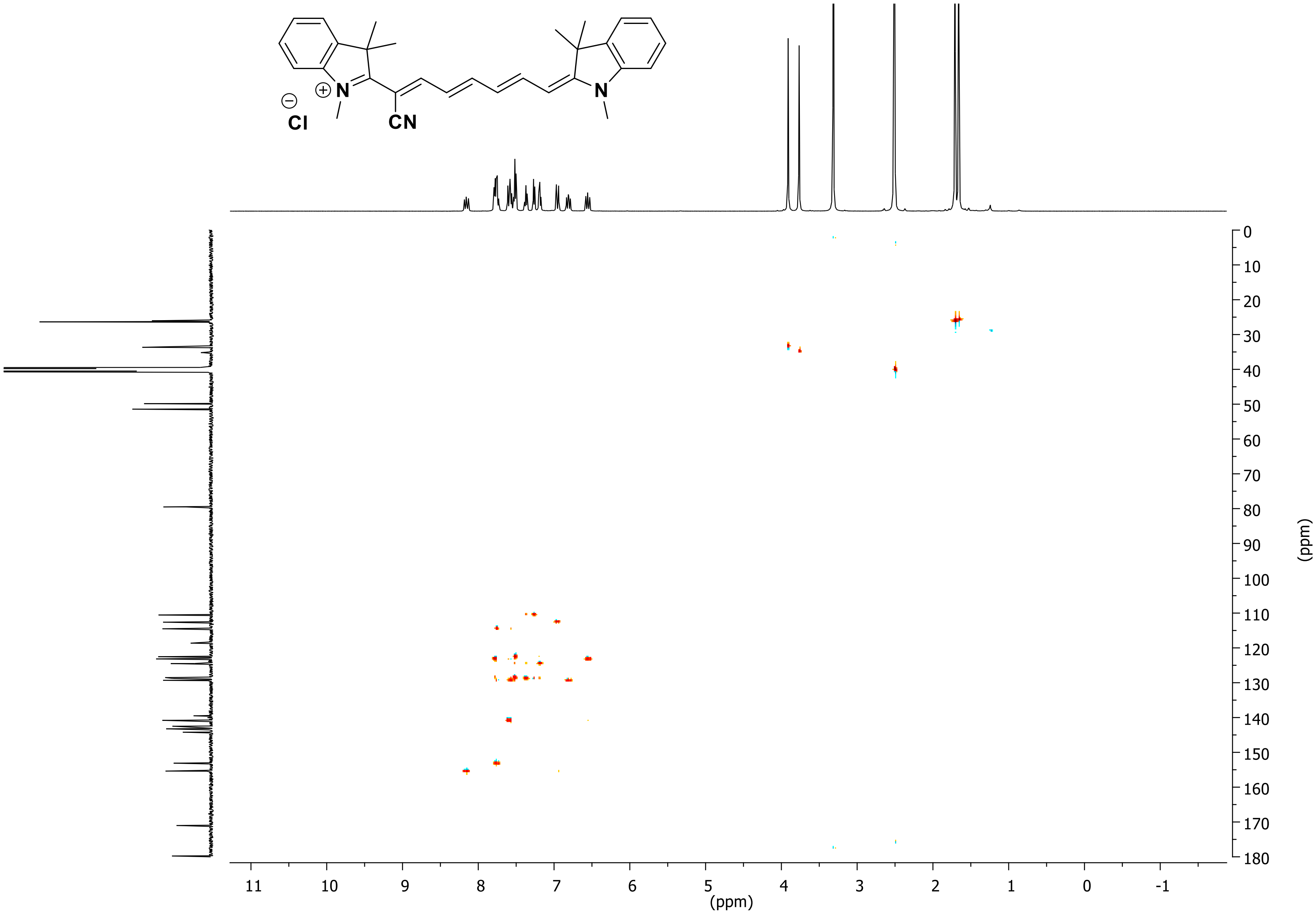

Figure S56. ^{1}H–$^{13}C\{^{1}H\}$ gHSQC (500 MHz, d_6–DMSO): **16**

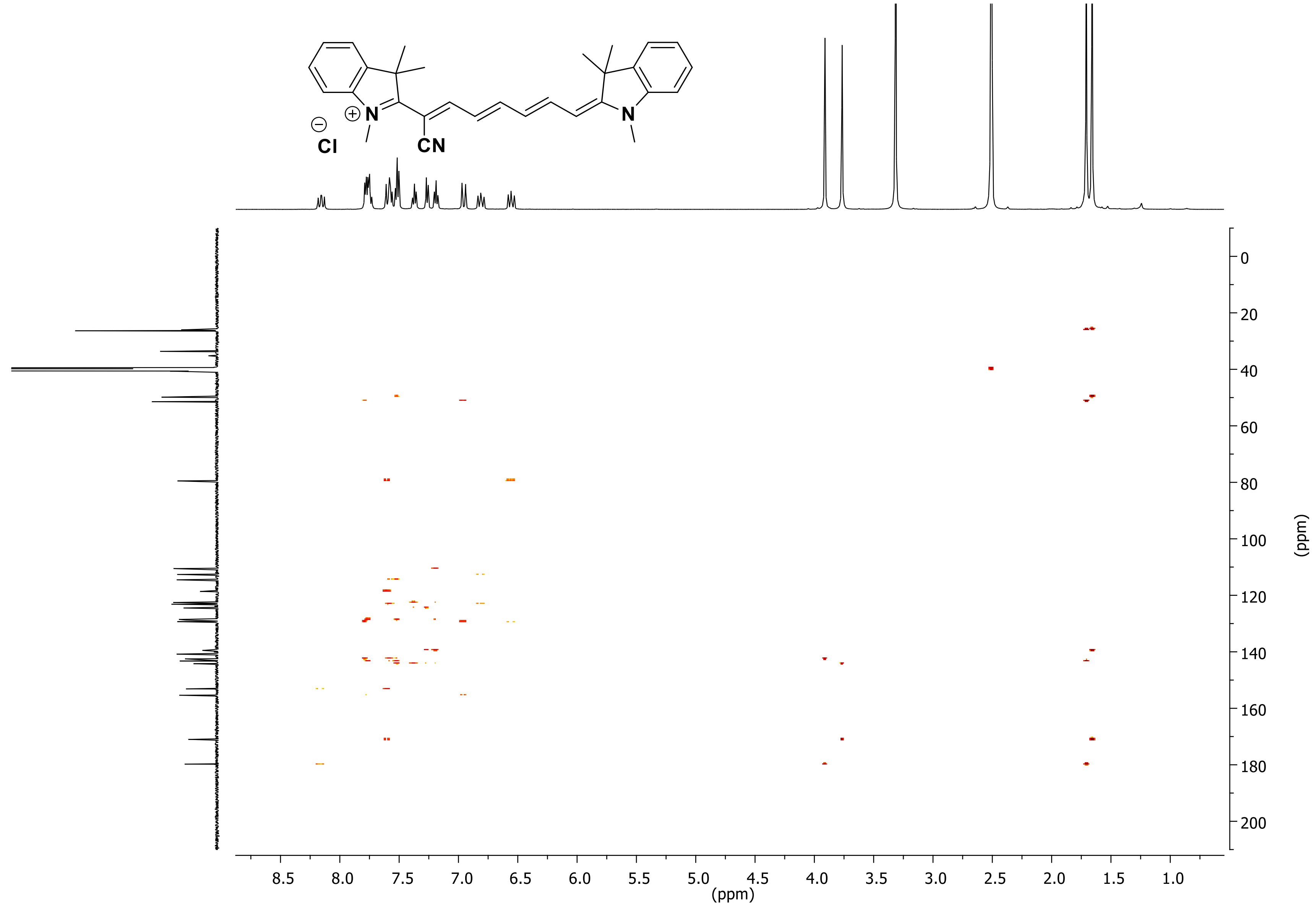

Figure S57. ^{1}H–$^{13}C\{^{1}H\}$ gHMBC (500 MHz, d_6–DMSO): **16**

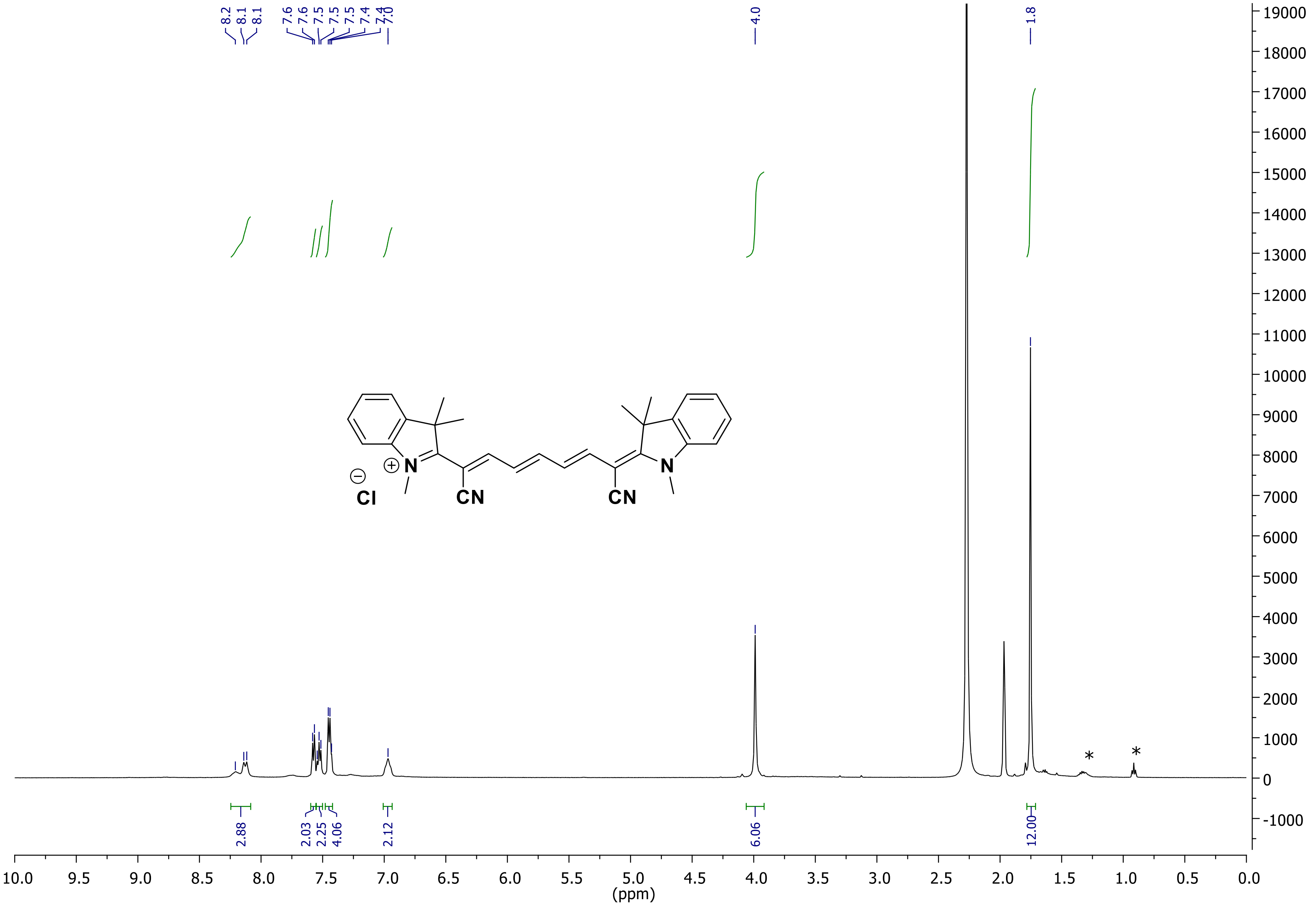

Figure S58. ^{1}H NMR (500 MHz, d_3-CD_3CN): **17** (*diethyl ether (Et_2O))

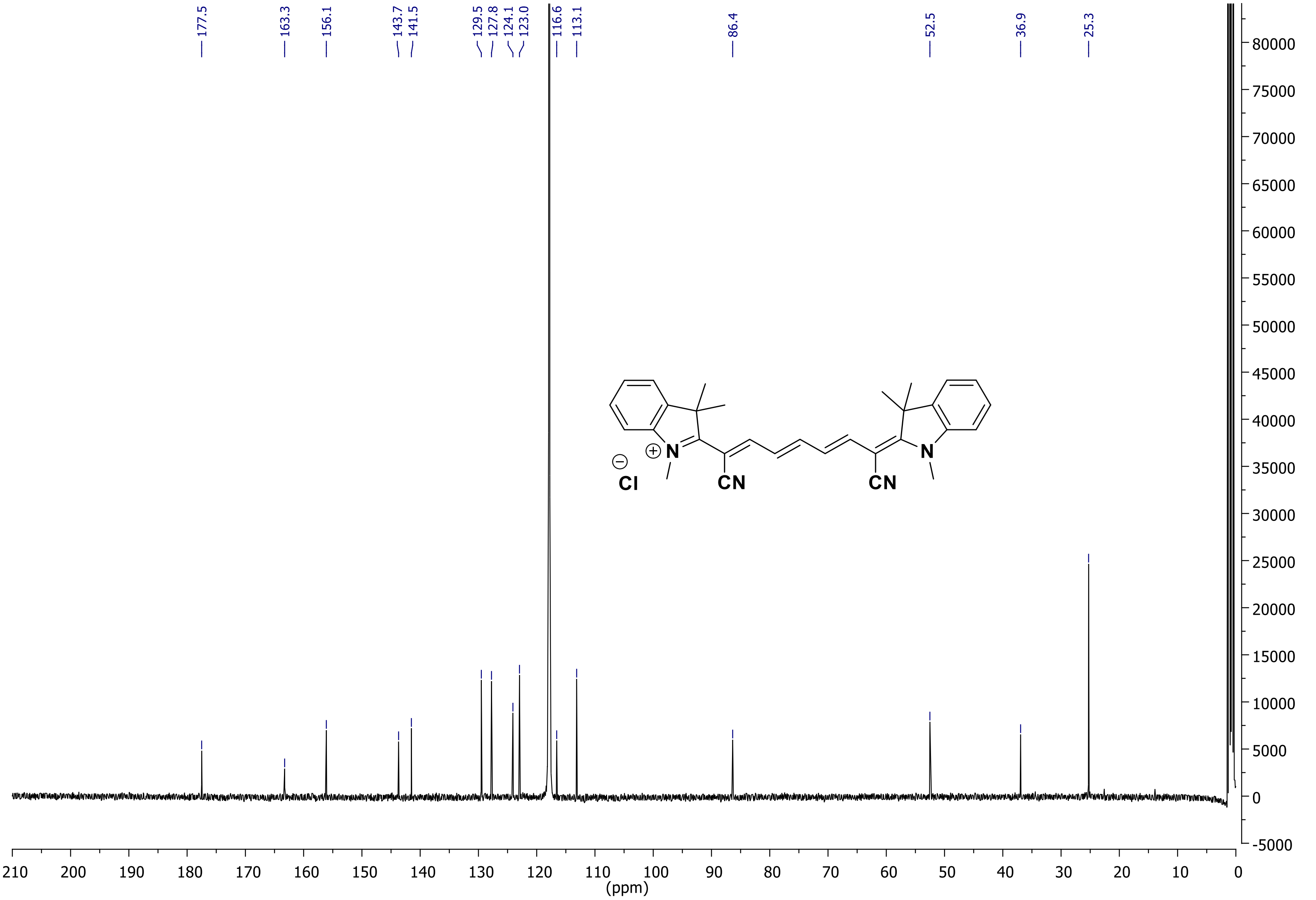

Figure S59. $^{13}C\{^1H\}$ NMR (126 MHz, d_3-CD_3CN): **17**

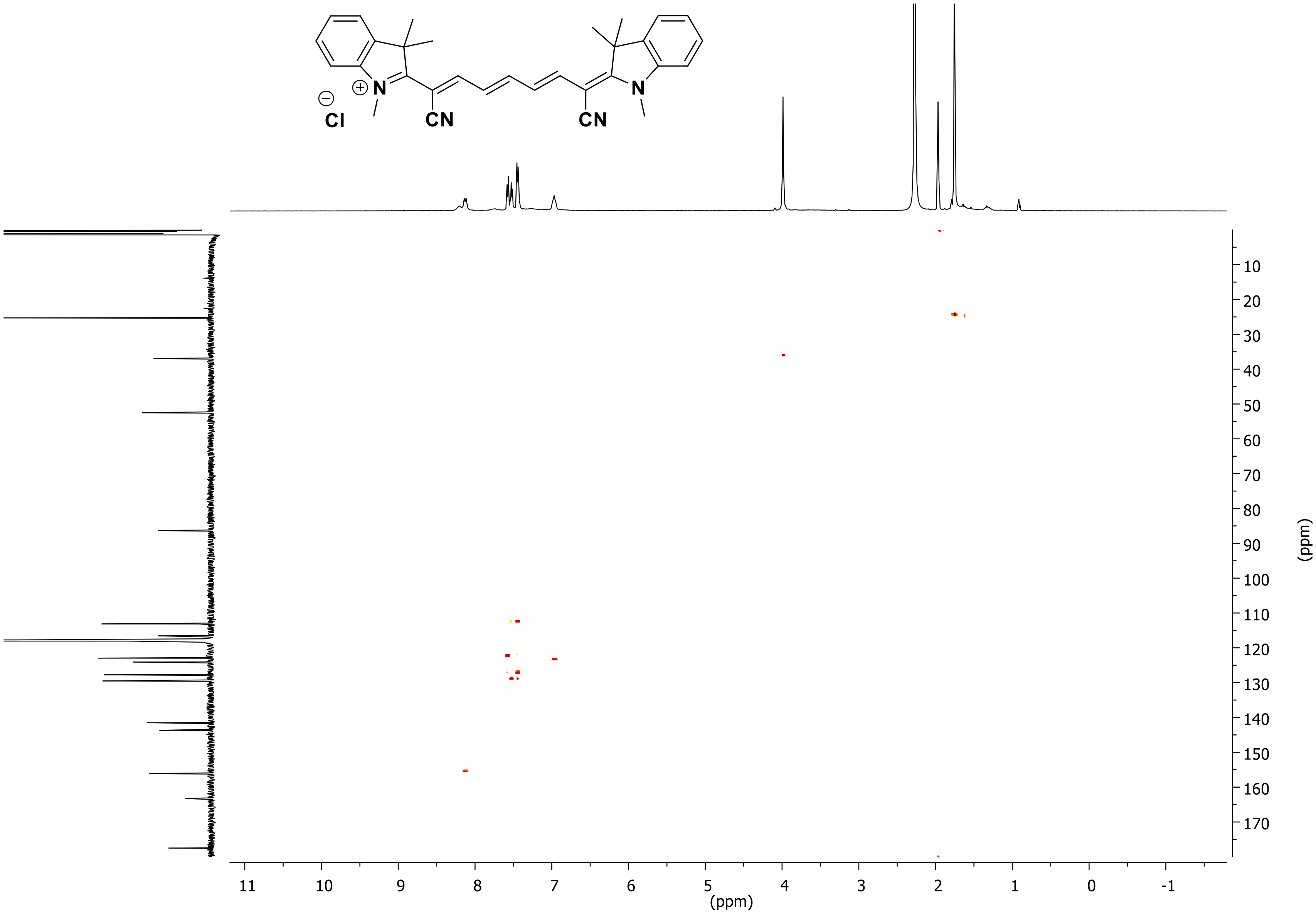

Figure S60. ^{1}H–$^{13}C\{^{1}H\}$ gHSQC (500 MHz, d_3-CD_3CN): **17**

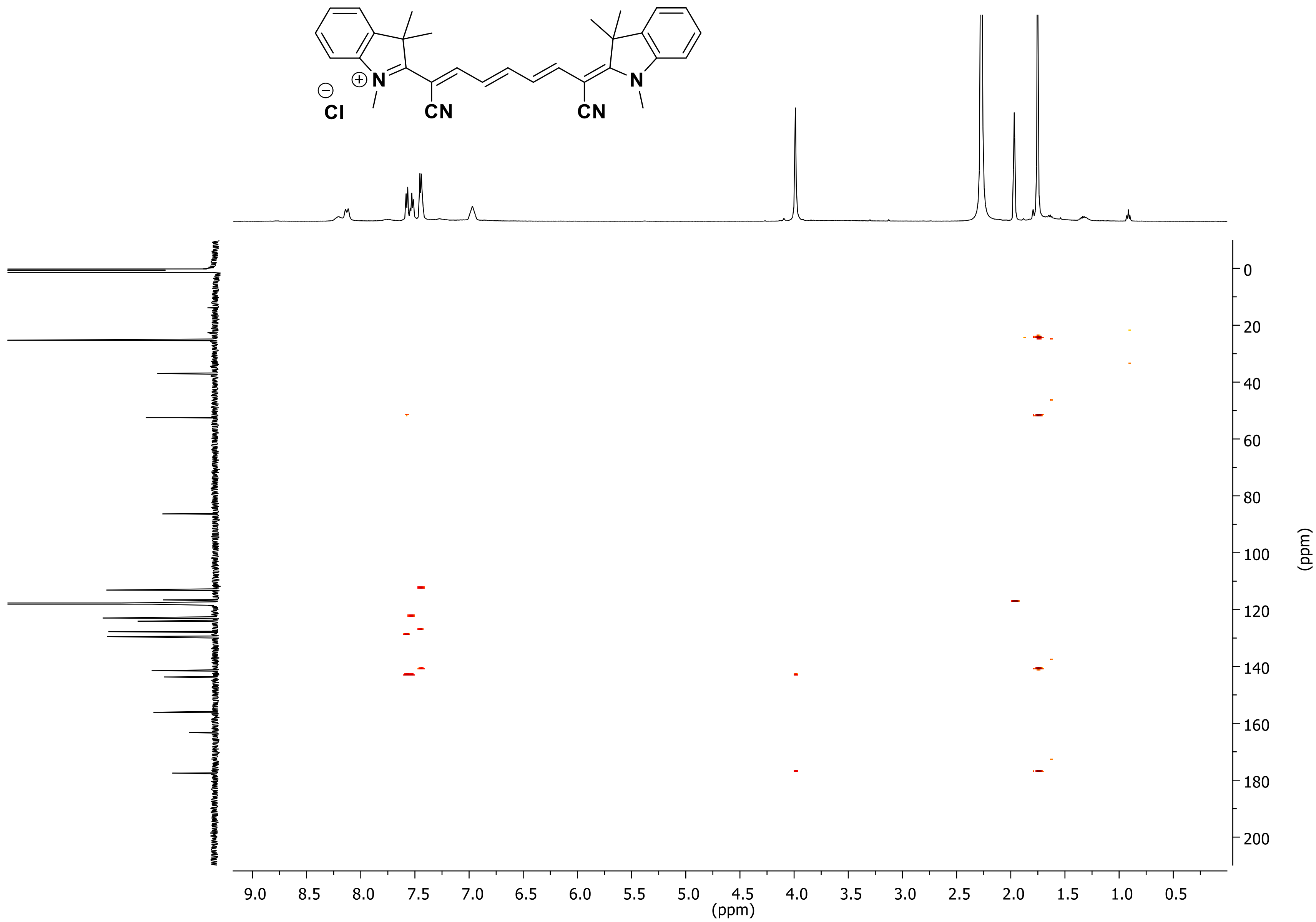

Figure S61. ^{1}H–$^{13}C\{^{1}H\}$ gHMBC (500 MHz, d_3-CD_3CN): **17**

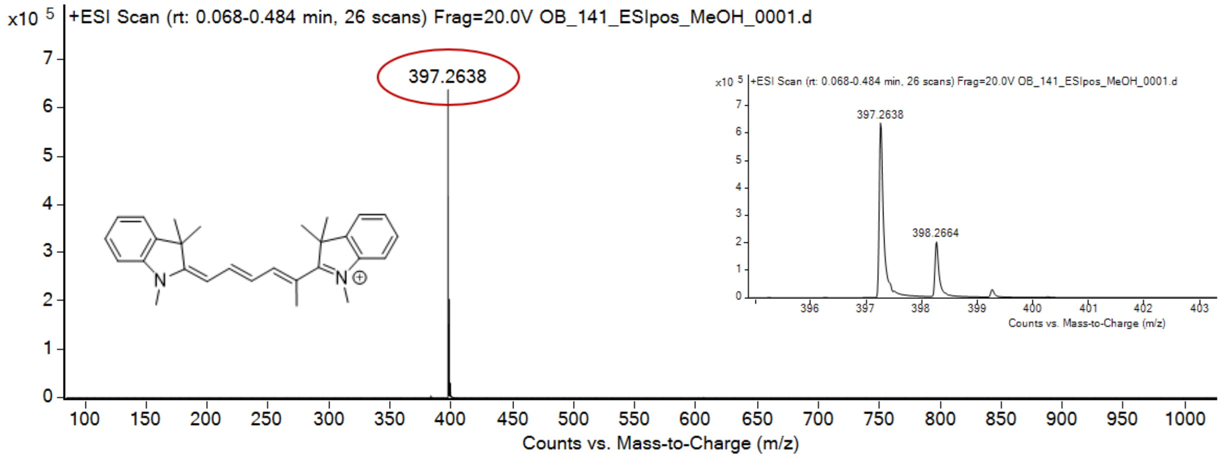

Figure S62. HRMS (ESI^+): **3** (m/z calcd for $C_{28}H_{33}N_2^+$ $[M - OTf^-]^+$ 397.2638, found 397.2638).

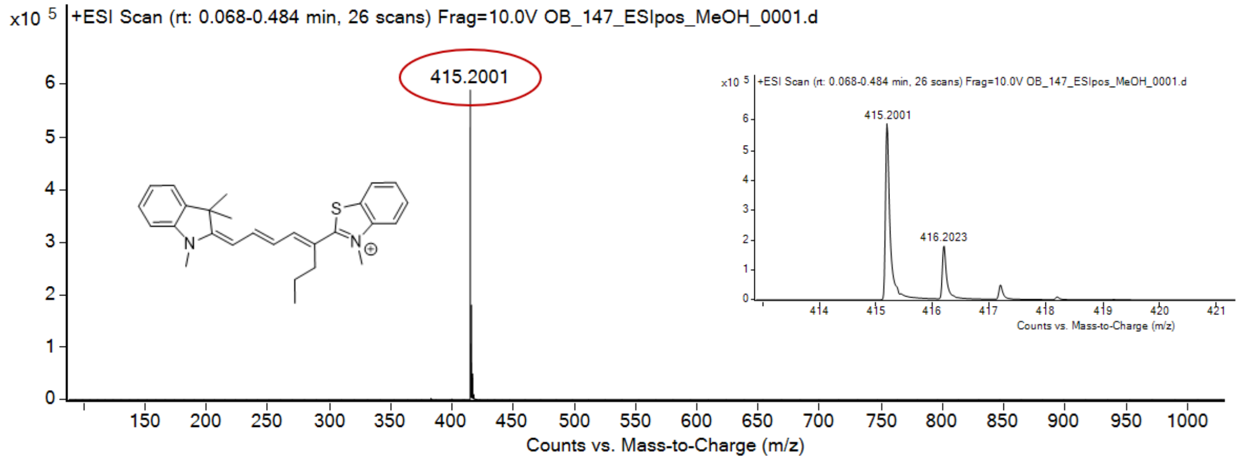

Figure S63. HRMS (ESI^+): **4** (m/z calcd for $C_{27}H_{31}N_2S^+$ $[M - OTf^-]^+$ 415.2002, found 415.2001).

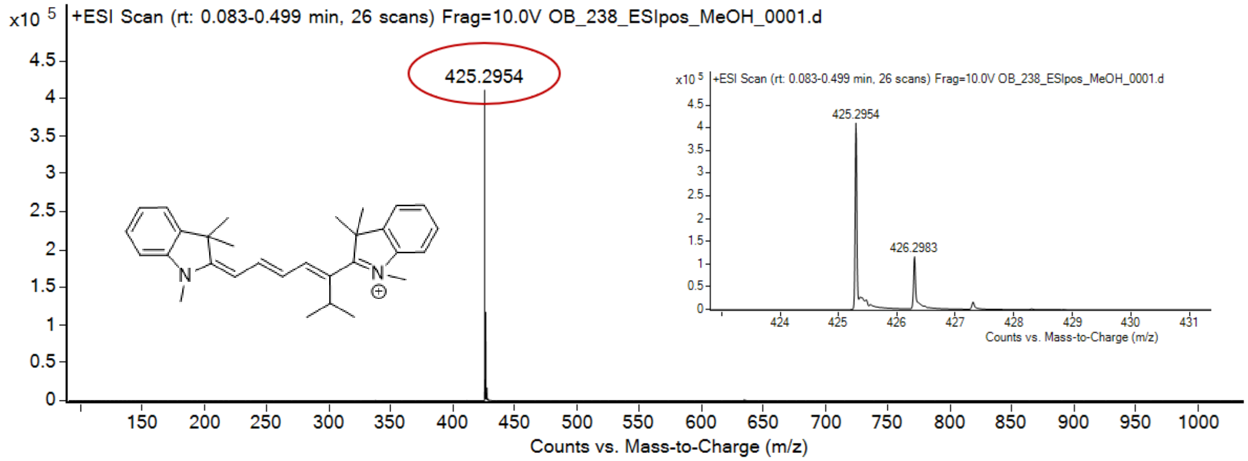

Figure S64. HRMS (ESI^+): **5** (m/z: calcd for $C_{30}H_{37}N_2^+$ $[M - OTf^-]^+$ 425.2951, found 425.2954).

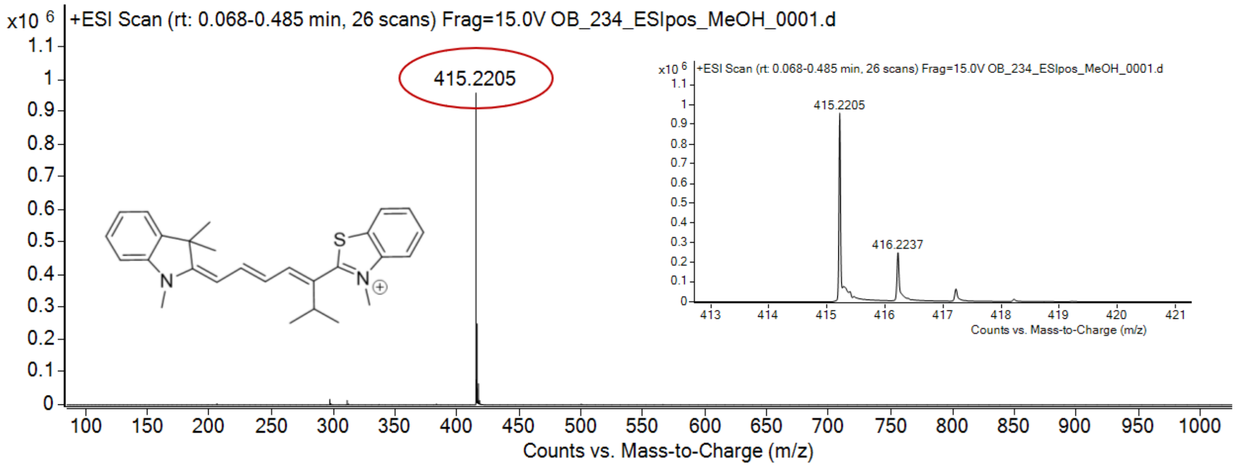

Figure S65. HRMS (ESI$^+$): **6** (*m/z*: calcd for $C_{27}H_{31}N_2S^+$ [M – OTf$^-$]$^+$ 415.2202, found 415.2205).

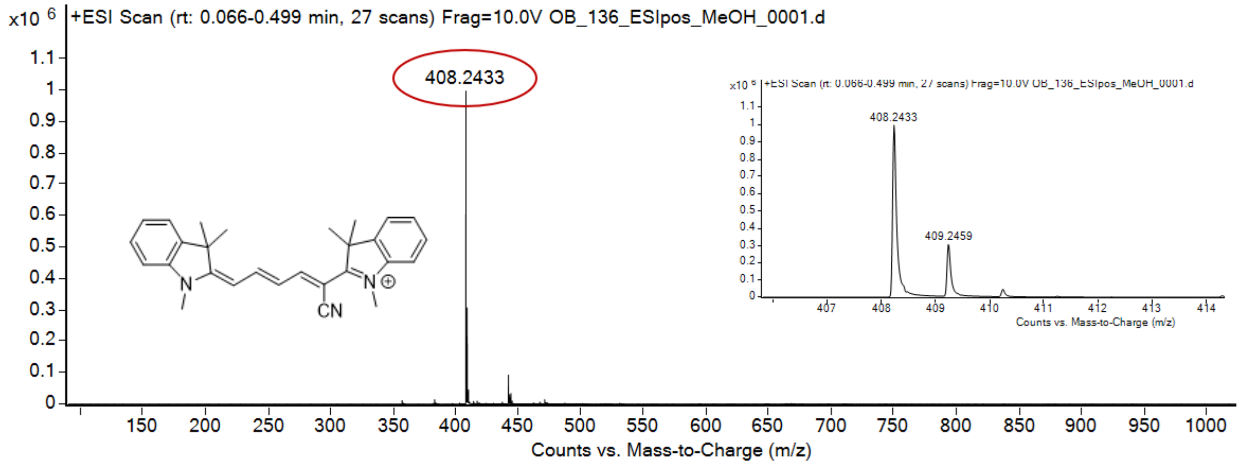

Figure S66. HRMS (ESI$^+$): **7** (*m/z*: calcd for $C_{28}H_{30}N_3^+$ [M – OTf$^-$]$^+$ 408.2434, found 408.2433).

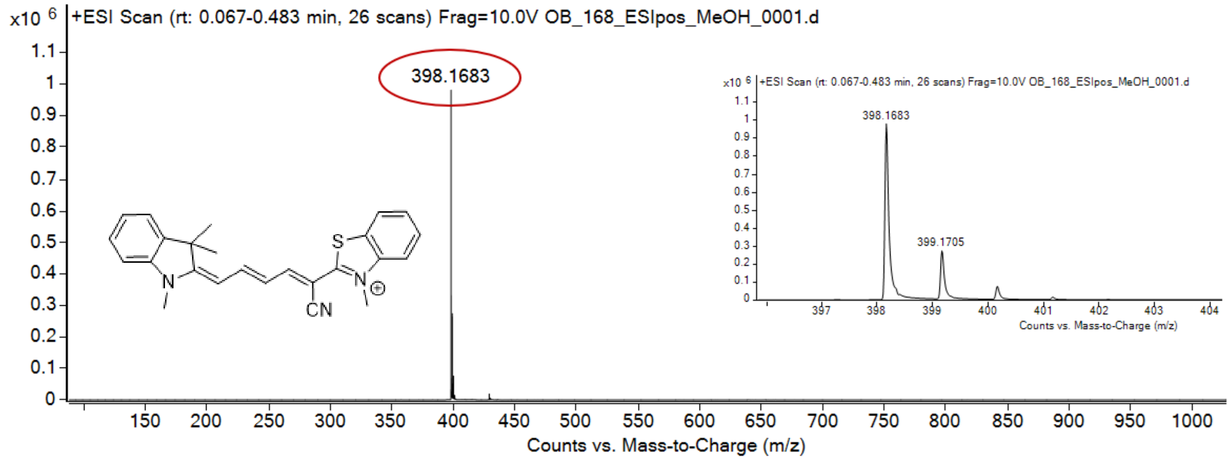

Figure S67. HRMS (ESI$^+$): **8** (*m/z*: calcd for $C_{25}H_{24}N_3S^+$ [M – OTf$^-$]$^+$ 398.1685, found 398.1683).

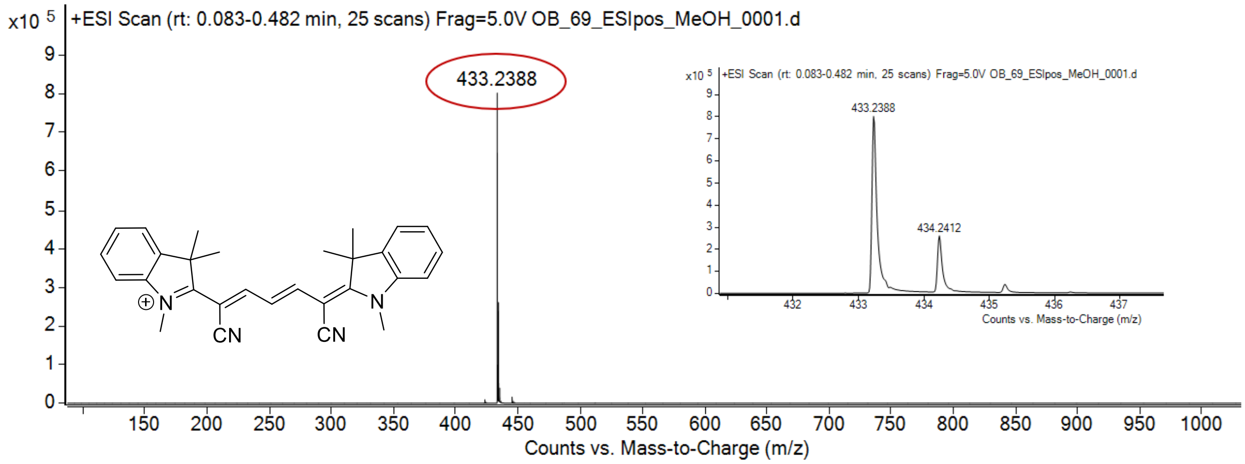

Figure S68. HRMS (ESI$^+$): **9** (*m/z*: calcd for $C_{29}H_{29}N_4^+$ $[M - OTf^-]^+$ 433.2387, found 433.2388).

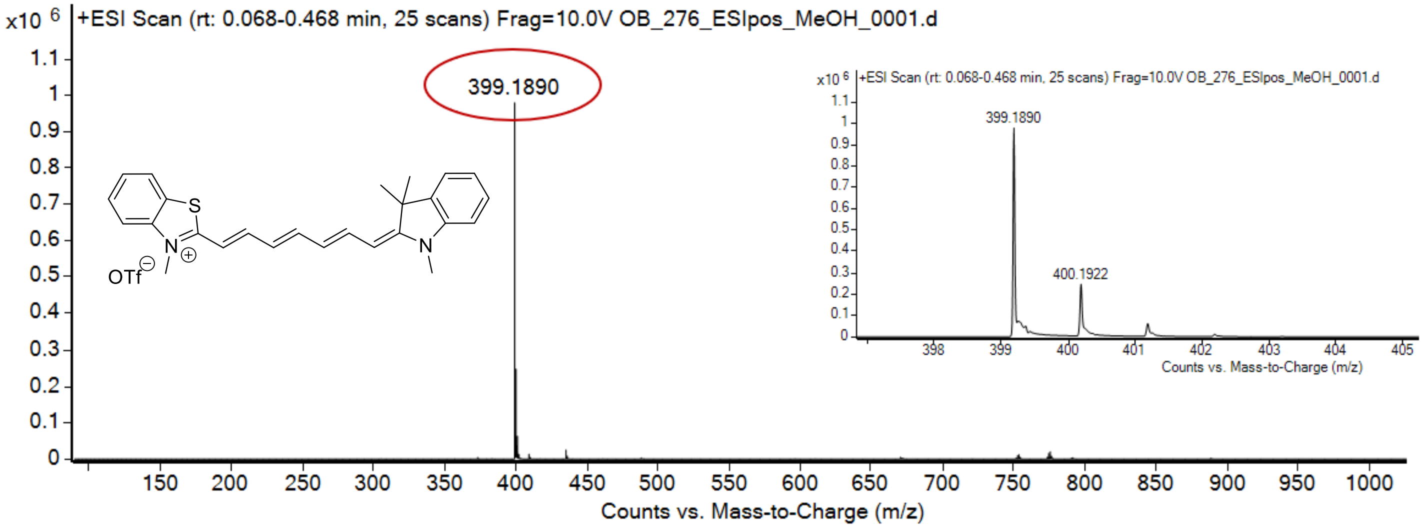

Figure S69. HRMS (ESI$^+$): **11** (*m/z*: calcd for $C_{26}H_{27}N_2S^+$ $[M - OTf^-]^+$ 399.1889, found 399.1890).

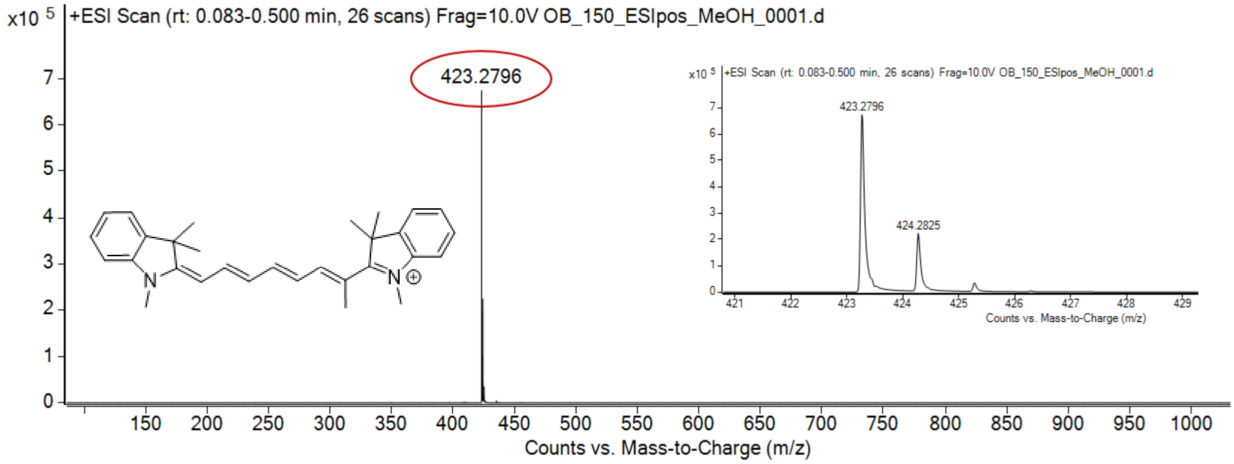

Figure S70. HRMS (ESI$^+$): **12** (*m/z*: calcd for $C_{30}H_{35}N_2^+$ $[M - OTf^-]^+$ 423.2795, found 423.2796).

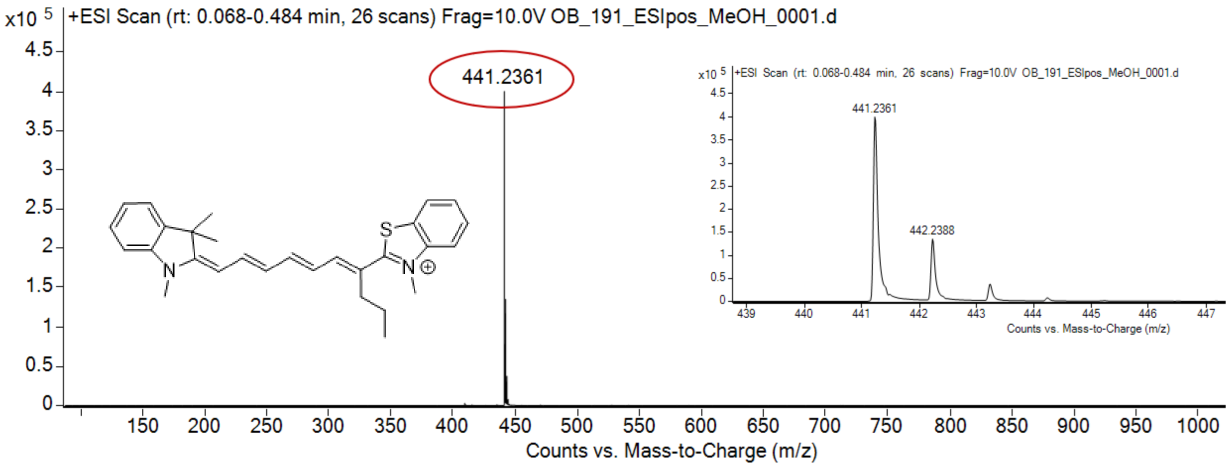

Figure S71. HRMS (ESI$^+$): **13** (*m/z*: calcd for $C_{29}H_{33}N_2S^+$ [M – OTf$^-$]$^+$ 441.2359, found 441.2361).

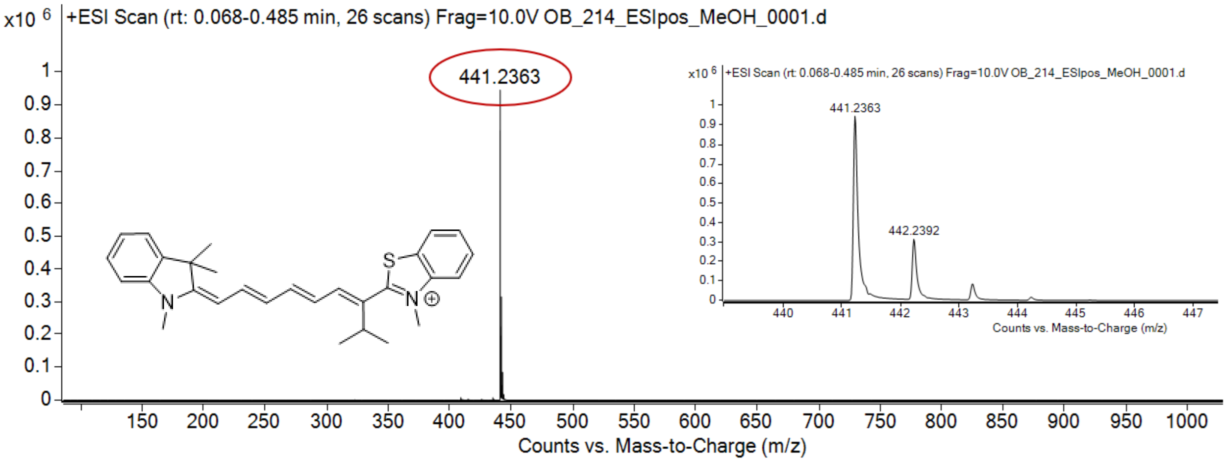

Figure S72. HRMS (ESI$^+$): **14** (*m/z*: calcd for $C_{29}H_{33}N_2S^+$ [M – OTf$^-$]$^+$ 441.2359, found 441.2363).

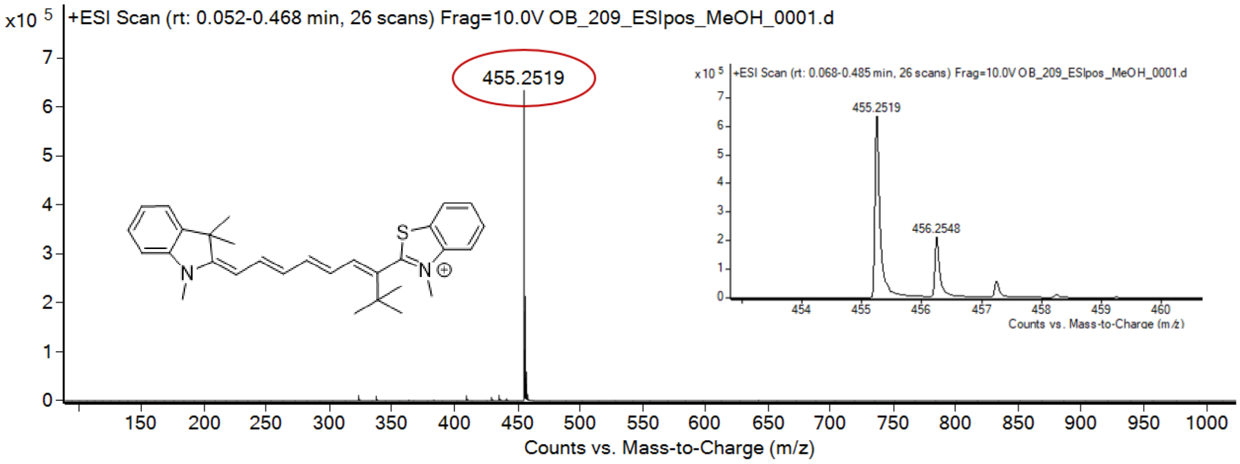

Figure S73. HRMS (ESI$^+$): **15** (*m/z* calcd for $C_{30}H_{35}N_2S^+$ [M – OTf$^-$]$^+$ 455.2515, found 455.2519).

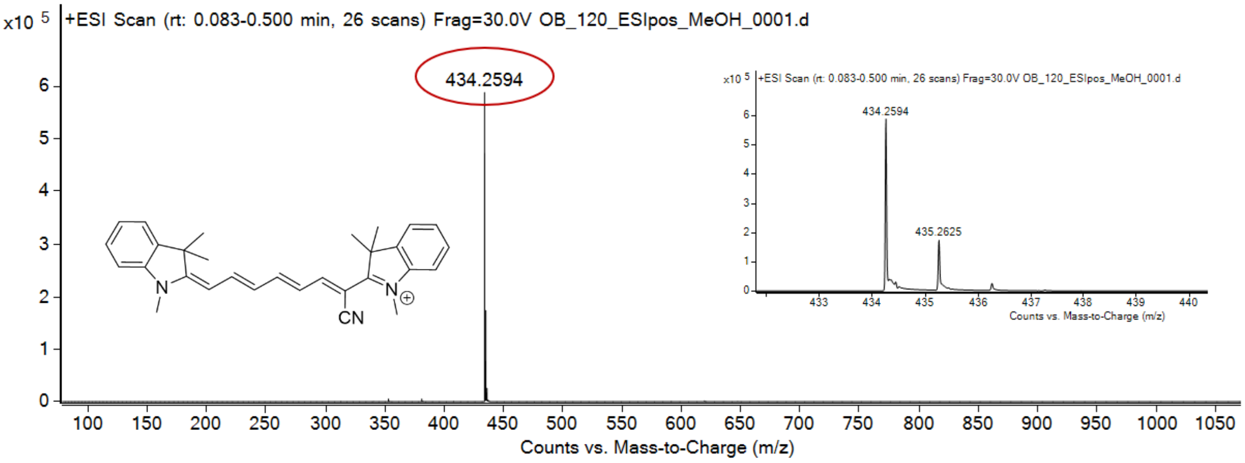

Figure S74. HRMS (ESI^+): **16** (*m/z*: calcd for $C_{30}H_{32}N_3^+$ $[M - OTf^-]^+$ 434.2591, found 434.2594).

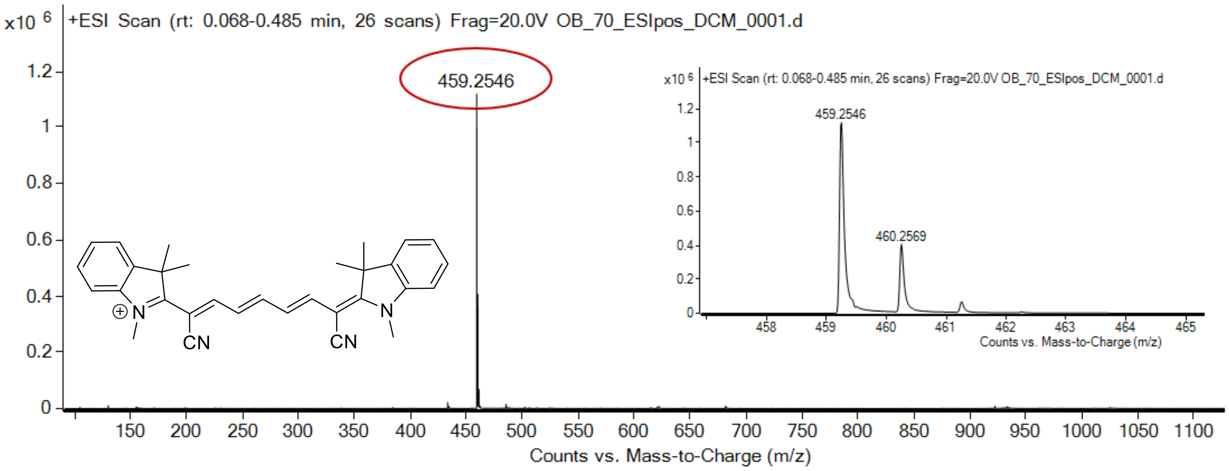

Figure S75. HRMS (ESI^+): **17** (*m/z*: calcd for $C_{31}H_{31}N_4^+$ $[M - OTf^-]^+$ 459.2543, found 459.2546).

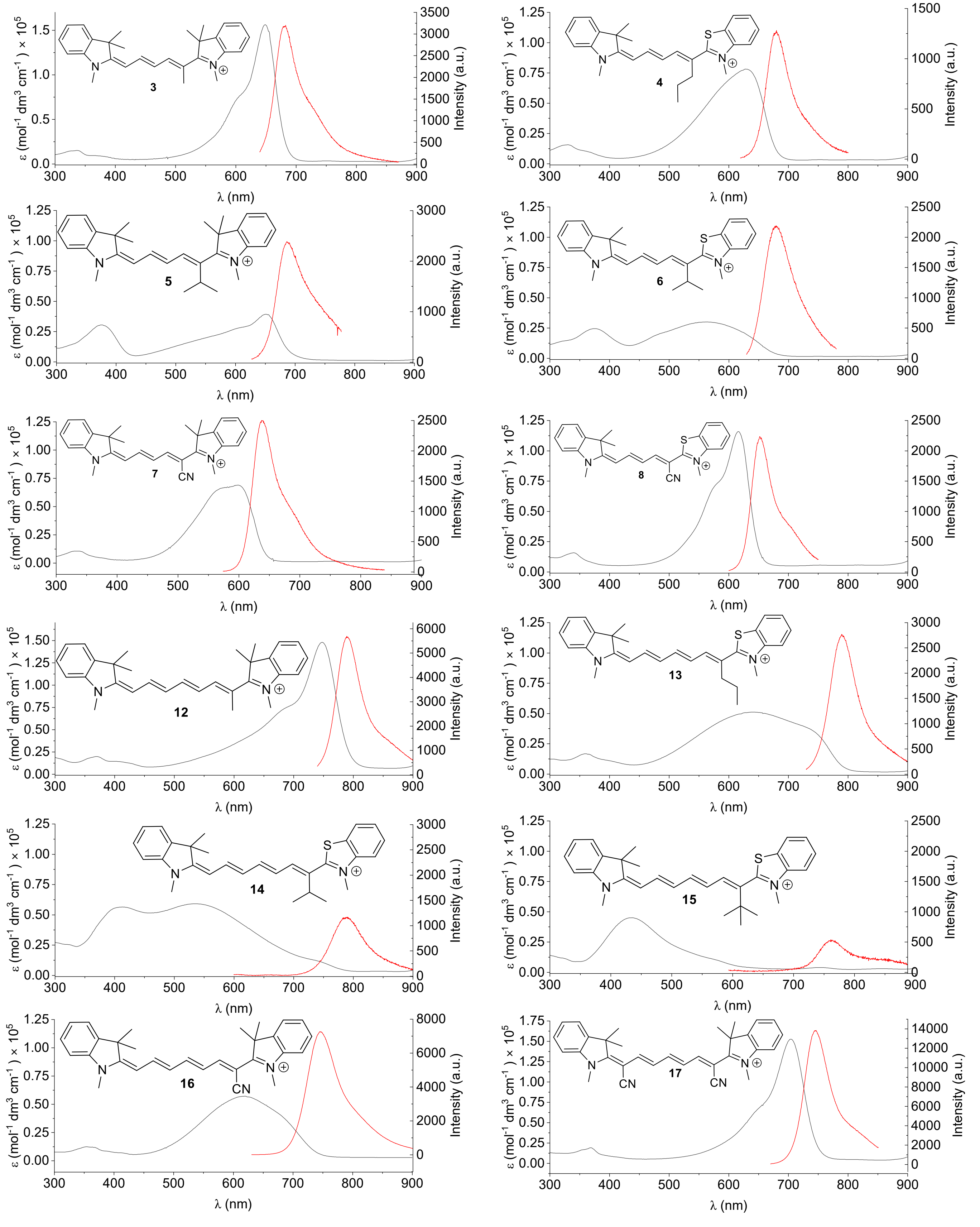

Figure S76: Absorption (black; left y-axes) and emission (red; right y-axes) spectra of **3**, **4**, **5**, **6**, **7**, **8**, **12**, **13**, **14**, **15**, **16**, and **17**. Measured in methanol

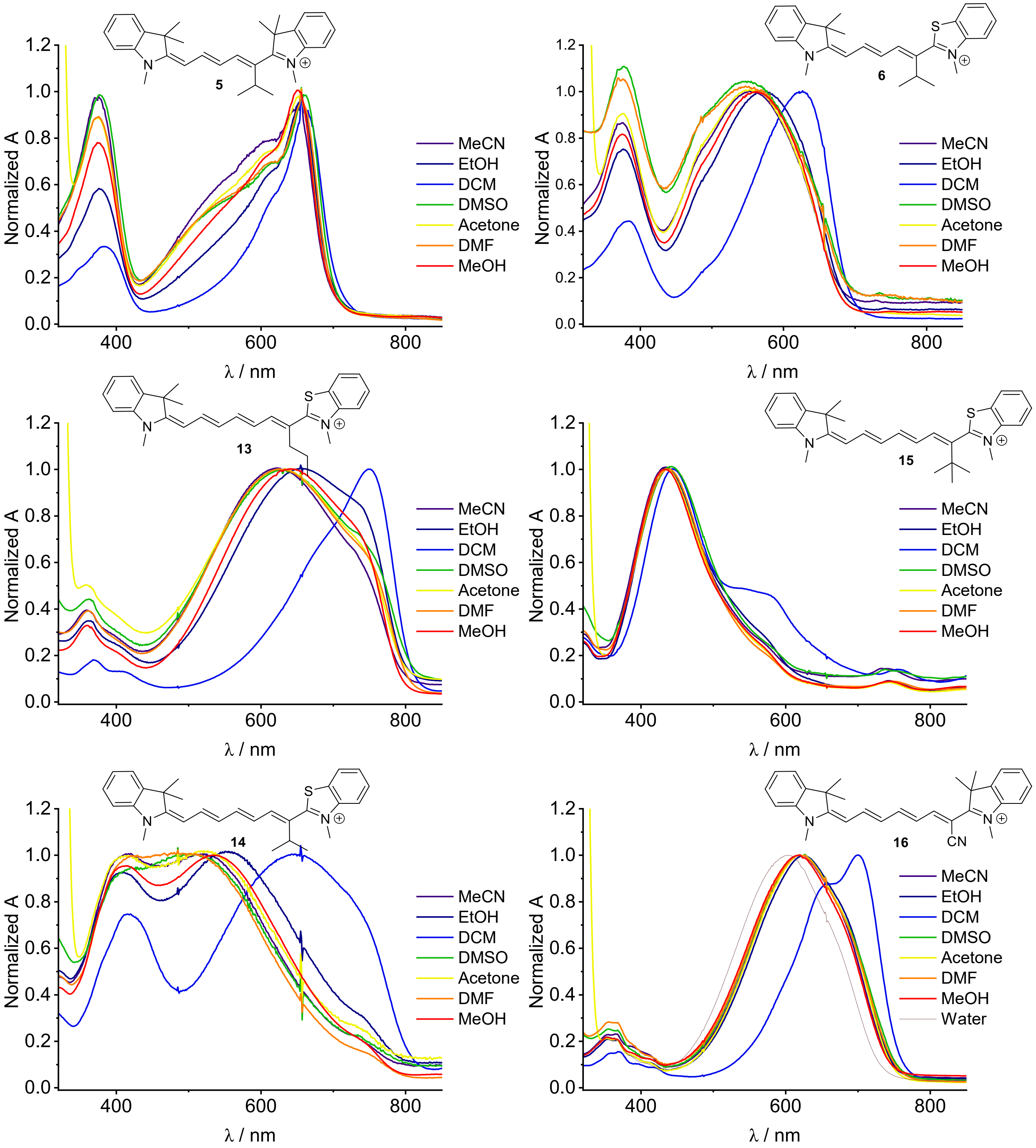

Figure S77. Absorption spectra of **5**, **6**, **13**, **15**, **14**, and **16** in different solvents.

Identification of Two Emissive Species of Compound 14

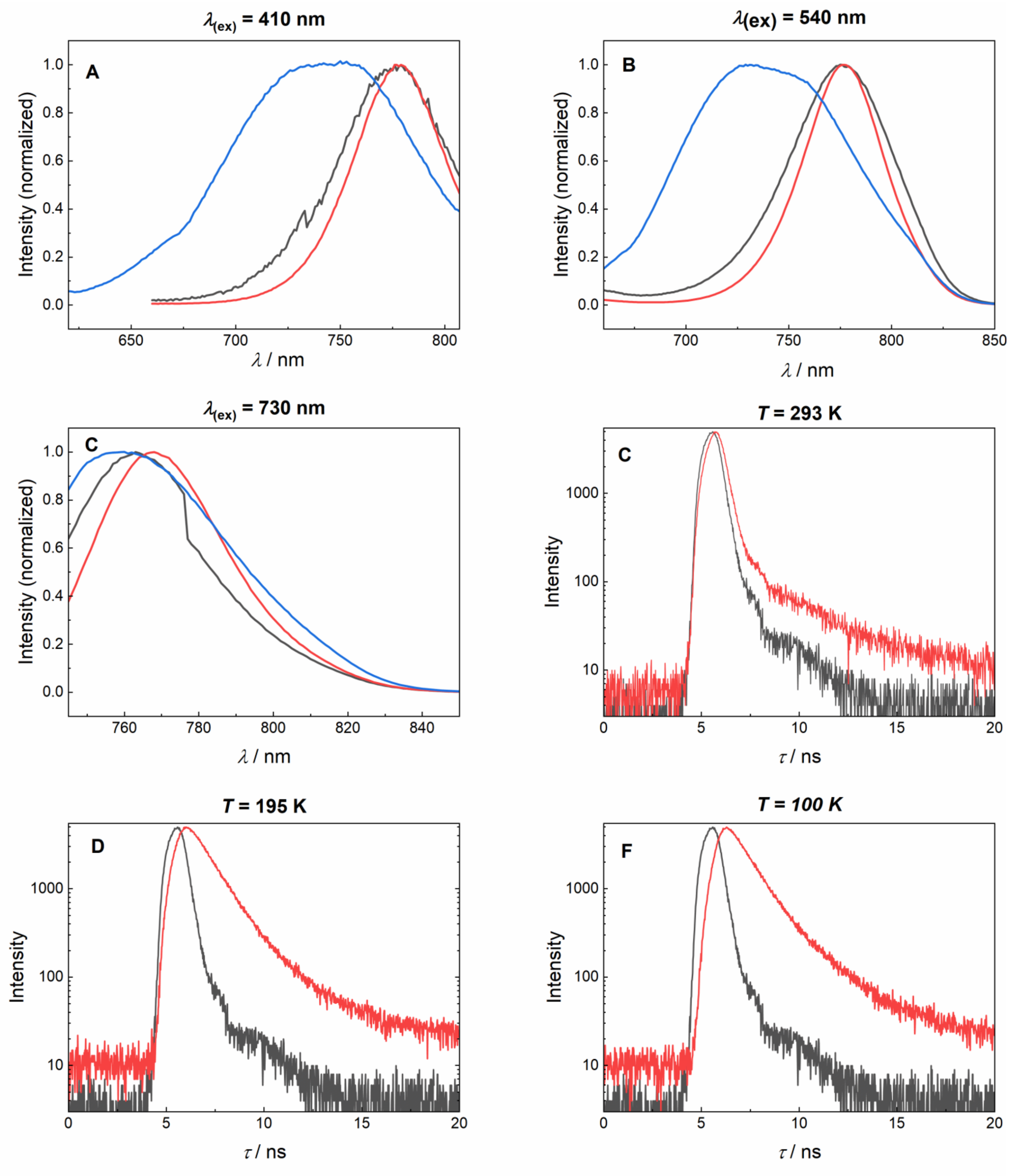

Figure S78A. Emission spectra of **14** at 293 K (black line), 195 K (red line), and 100 K (blue line) at (A) $\lambda_{(ex)}$ = 410 nm, (B) 540 nm, and (C) 730 nm. Fluorescence lifetime τ_f) (red line) of **14** measured with $\lambda_{(ex)}$ = 540 nm at different temperatures: (D) 293 K, (E) 195 K, and (F) 100 K in methanol. Instrument response function (IRF) is shown with a black line.

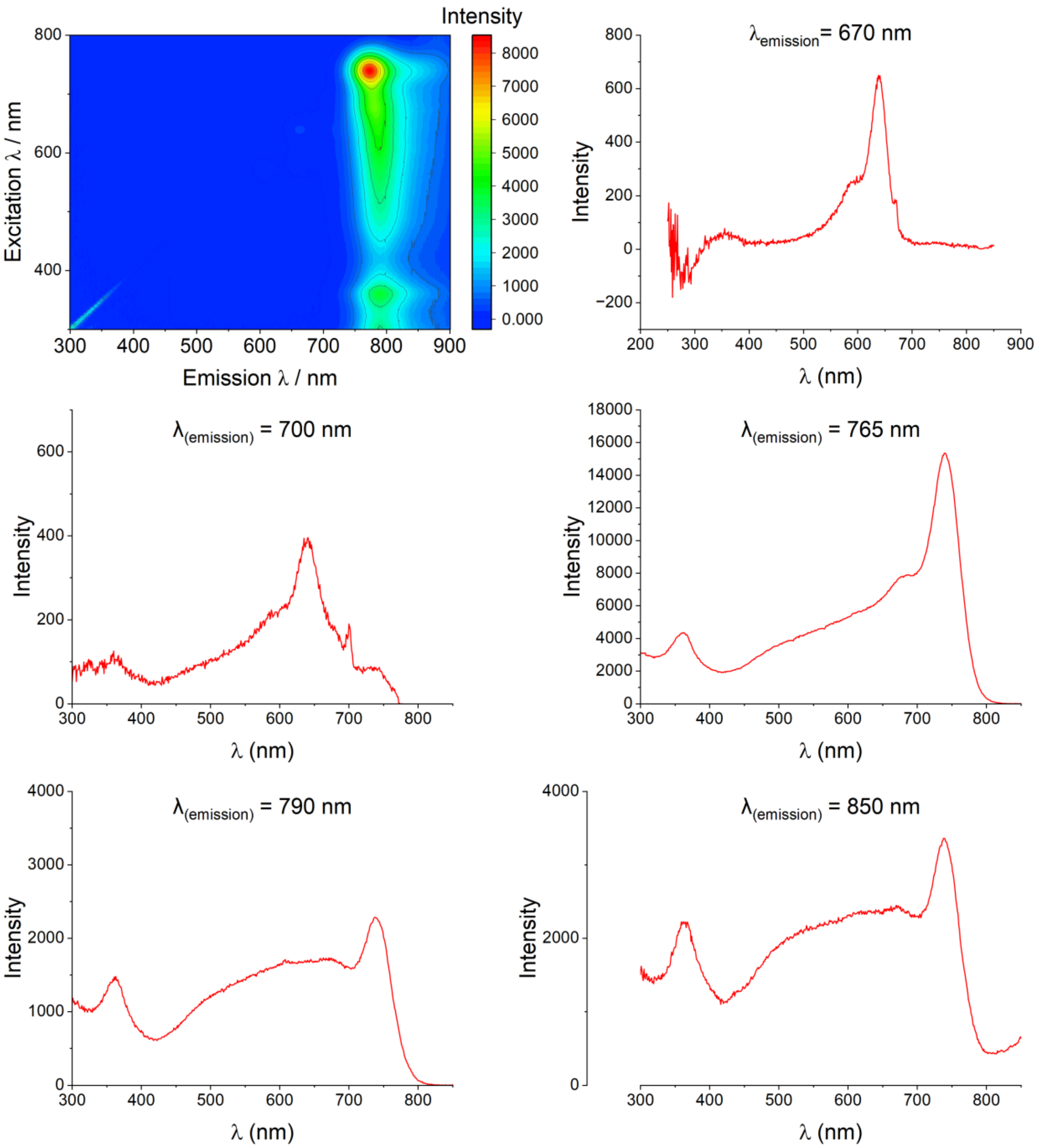

Figure S78B. Heat map (excitation-emission matrix) and excitation spectra of **14** obtained in methanol.

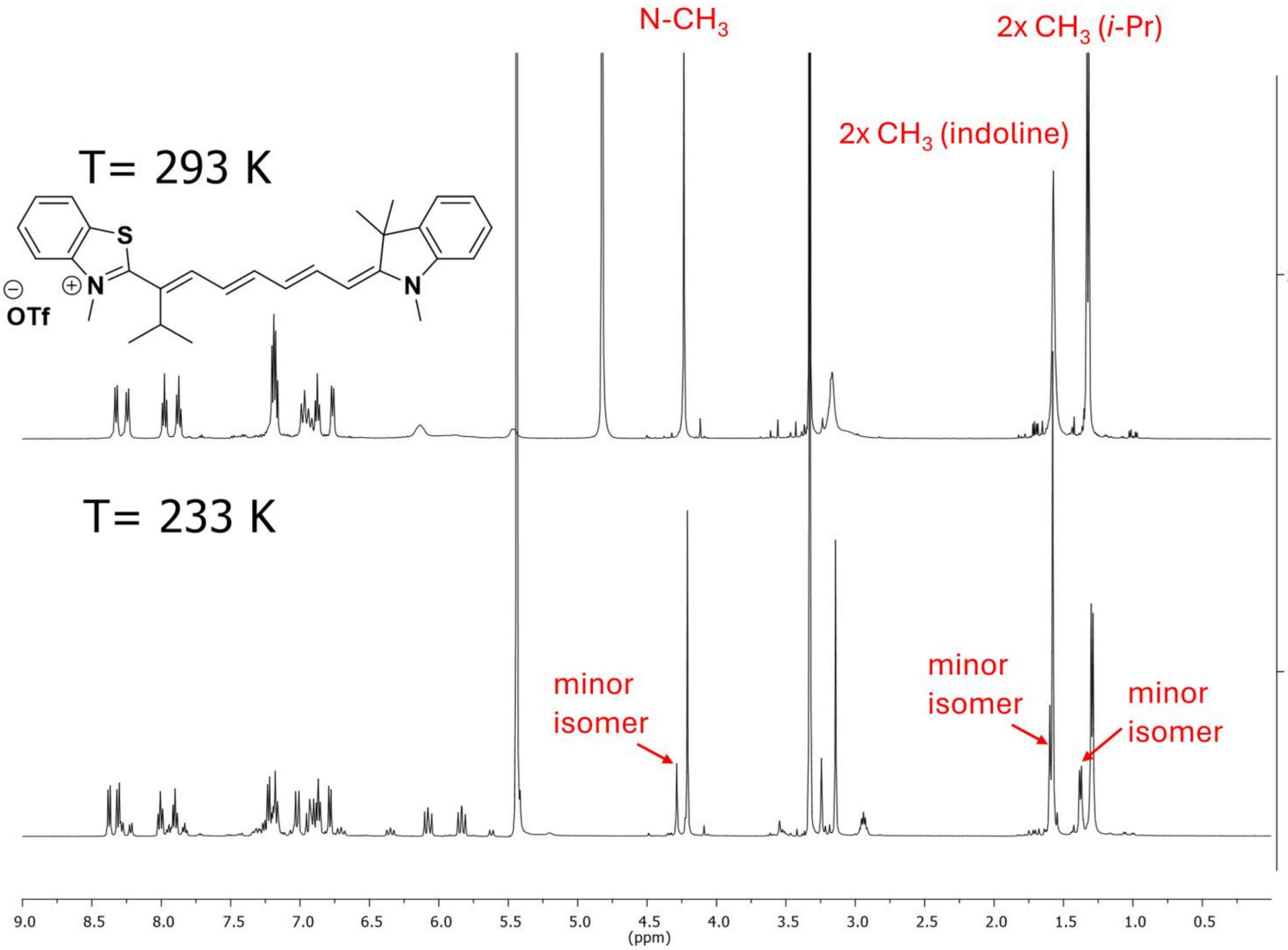

Figure S78C. ^{1}H NMR of **14** measured at 293 and 233 K in CD_3OD.

Table S1. Fluorescence lifetimes (τ_f) of **14** in methanol measured at different temperatures.

Temperature (K)	τ_1 /ns	τ_2 /ns
293	0.14 ± 0.01	3.70 ± 0.32
195	1.11	5.71
100	1.09	4.05

Quantum-Chemical Calculations

Methodology

Cyanine molecules represent peculiar systems from the point of view of theoretical modelling.[3] Jacquemin et al. recommended the use of DFT for geometry optimizations in the ground and excited states.[3-4] They also noted that the choice of basis set has minimal influence on the prototypical geometries of heptamethine cyanines.[4] Among several works, cyanine structures have previously been characterized at the IEF-PCM-CAM-B3LYP/6-31+G** level of theory.[1, 5]

However, the excitation energies of cyanines are consistently shifted to higher energies by the TDDFT method for a wide range of cyanine derivatives.[3] In contrast, ZINDO/S results correlate quite well with those calculated by the more advanced CASPT2 method, which is shown within their Supplementary Materials.[3]

Based on these findings, we employed the IEF-PCM-CAM-B3LYP/6-31+G** method for the ground state structure optimization and the IEF-PCM-ZINDO/S method to characterize the excited states. All structures are minimal structures as no imaginary frequencies were found after frequency analyses. For comparative purposes, we also used IEF-PCM-TD-CAM-B3LYP/6-31+G** and SC-NEVPT2/cc-pVDZ.[6-7] The state-specific effects were neglected for the solvation excited state calculations. As for the SC-NEVPT2 calculations, we employed the active space of 6 electrons in 10 orbitals (6/10) and 8 electrons in 14 orbitals (8/14) for pentamethine (Cy5) and heptamethine cyanine dyes (Cy7), respectively. The active spaces are recommended by Send et al. as an optimal cost/quality trade-off for these systems.[8] All DFT and ZINDO/S calculations were performed using the Gaussian 16 Revision A.03 quantum chemistry package.[9] The SC-NEVPT2 calculations were conducted using the ORCA 6.0.1 quantum chemistry package.[10]

The absorption spectra were evaluated from the ground state optimal minima using an empirical broadening scheme with a Gaussian broadening parameter of 0.15 eV. The Natural Transition Orbitals (NTOs) were modelled using the TheoDORE 3.2 program.[11]

The vibronic spectrum was calculated for the $S_0 \rightarrow S_1$ transition on IEF-PCM-TD-CAM-B3LYP/6-31+G** level of theory and modeled in the time-independent regime via FCClasses 3 package.[12] The spectrum was modeled in the Franck–Condon approximation (i.e., no HT effects were included) using the adiabatic Hessian method using Duschinsky rotations with the computed Hessians corresponding to the optimized structures in the S_0 and S_1 minima at the IEF-PCM-CAM-B3LYP/6-31+G** and IEF-PCM-TD-CAM-B3LYP/6-31+G** levels of theory, respectively. The transition dipole moments were calculated at the same level of theory. The computation was conducted using a Cartesian coordinate set. The resulting stick spectrum was broadened by Gaussian functions with HWHM = 0.05 eV.

Cartesian Coordinates and total SCF energies (in a.u.) for structures optimized at the IEF-PCM-CAM-B3LYP/6-31+G level of theory**

1 (E_{SCF} = −1156.29758160 a.u.)

C	7.318929	0.778178	0.011688
C	5.968623	0.461951	0.007717
C	5.512900	-0.852145	-0.007865
C	6.417697	-1.898778	-0.019253
C	7.784647	-1.606969	-0.015236
C	8.224032	-0.284492	-0.000107
N	4.853402	1.327404	0.017527
C	3.693509	0.641577	0.007300
C	3.998408	-0.860065	-0.009507
C	2.461803	1.288115	0.009172
C	1.214388	0.664698	0.002754
C	0.000013	1.343332	0.000051
C	-1.214402	0.664686	-0.002858
C	-2.461776	1.288099	-0.009100
C	-3.693515	0.641548	-0.007395
N	-4.853368	1.327387	-0.017654
C	-5.968618	0.461952	-0.007633
C	-5.512911	-0.852146	0.007896
C	-3.998424	-0.860084	0.009354
C	-6.417731	-1.898762	0.019369
C	-7.784672	-1.606919	0.015527
C	-8.224036	-0.284434	0.000500
C	-7.318913	0.778218	-0.011391
C	-4.945878	2.777665	-0.035506
C	-3.470492	-1.533650	1.290496
C	-3.468422	-1.565430	-1.253772
C	3.470639	-1.533541	-1.290772
C	3.468216	-1.565472	1.253487
C	4.945951	2.777669	0.035363
H	-6.078888	-2.930099	0.031312
H	-8.508084	-2.415035	0.024542
H	-9.287673	-0.070941	-0.001990
H	-7.675967	1.800877	-0.022644
H	-5.991580	3.071698	-0.055456
H	-4.453352	3.178125	-0.924506
H	-4.480410	3.199587	0.858308
H	-3.839020	-1.021881	2.182750
H	-3.818315	-2.569062	1.322188
H	-2.380150	-1.543555	1.324998
H	-3.834690	-1.075639	-2.159155
H	-2.378057	-1.578034	-1.286195
H	-3.817507	-2.600939	-1.260389
H	-2.465113	2.372673	-0.014883
H	-1.161198	-0.419546	0.000096
H	-0.000004	2.431090	0.000213
H	1.161207	-0.419535	-0.000519
H	2.465119	2.372689	0.015280
H	6.078831	-2.930107	-0.031253
H	8.508044	-2.415099	-0.024168
H	9.287673	-0.071020	0.002553
H	7.675996	1.800832	0.023067
H	3.818417	-2.568969	-1.322462
H	2.380301	-1.543392	-1.325448
H	3.839335	-1.021738	-2.182936
H	2.377844	-1.578050	1.285752
H	3.817270	-2.600992	1.260113
H	3.834355	-1.075746	2.158959
H	4.454817	3.178130	0.925148
H	4.479100	3.199630	-0.857702
H	5.991687	3.071693	0.053633

7 (E_{SCF} = −1248.49742756 a.u.)

C	-3.792293	0.542298	-0.038832
N	-4.944830	1.204473	-0.148310
C	-6.057685	0.329856	-0.074144
C	-5.586947	-0.966532	0.093276
C	-4.074262	-0.951167	0.132427
C	-6.481576	-2.017914	0.193956
C	-7.849033	-1.742102	0.124121
C	-8.302305	-0.434209	-0.044034
C	-7.408426	0.631844	-0.146973
H	-6.134918	-3.038092	0.324254
H	-8.565231	-2.552927	0.201264
H	-9.367492	-0.236650	-0.096266
H	-7.774208	1.642884	-0.277192
C	-5.059552	2.647202	-0.322162
H	-6.109451	2.916402	-0.387427
H	-4.559593	2.955467	-1.241981
H	-4.616122	3.164991	0.530120
C	-3.567857	-1.471240	1.492575
H	-3.962573	-0.870718	2.315436
H	-3.905152	-2.501430	1.628099
H	-2.478677	-1.463581	1.550998
C	-3.500696	-1.778963	-1.034876
H	-3.843302	-1.392887	-1.997722
H	-2.409947	-1.785474	-1.033369
H	-3.843087	-2.811933	-0.939403
C	-2.555502	1.212667	-0.084157
C	-1.321098	0.613585	0.015831
C	-0.099099	1.318972	-0.035693
C	1.097492	0.659339	0.067380
C	2.394950	1.251365	0.042401
H	-2.577043	2.289382	-0.209090

H	-1.251929	-0.462136	0.139409
H	-0.126644	2.397894	-0.158939
H	1.049811	-0.413334	0.198258
C	2.417500	2.666583	-0.149016
C	3.584918	0.506479	0.099128
C	3.711716	-1.015884	-0.097533
C	5.215963	-1.180181	-0.128356
C	5.817547	0.047832	0.101558
N	4.809556	1.027026	0.280101
C	5.995927	-2.306847	-0.321089
C	7.386545	-2.171635	-0.292076
C	7.973352	-0.927298	-0.068889
C	7.193317	0.212715	0.133222
H	5.544704	-3.278262	-0.496822
H	8.014251	-3.042147	-0.448801
H	9.054191	-0.836872	-0.054952
H	7.658746	1.177462	0.294924
C	3.092614	-1.485101	-1.425128
H	3.322897	-2.542952	-1.571811
H	2.007836	-1.374536	-1.438071
H	3.505679	-0.926113	-2.268001
C	3.150619	-1.803687	1.107537
H	2.064058	-1.752347	1.177033
H	3.425207	-2.855084	0.996278
H	3.574384	-1.438450	2.045904
C	5.142204	2.380691	0.706291
H	6.074234	2.339706	1.267409
H	4.365525	2.759883	1.366482
H	5.262081	3.051705	-0.146012
N	2.348385	3.806807	-0.353287

8 (E_{SCF} = −1528.80782979 a.u.)

C	-3.697315	0.501501	-0.052641
N	-4.857545	1.155931	-0.160956
C	-5.963207	0.277351	-0.054279
C	-5.483588	-1.013133	0.134738
C	-3.970399	-0.989725	0.154429
C	-6.370899	-2.066687	0.269580
C	-7.741036	-1.800447	0.211449
C	-8.203440	-0.498982	0.020907
C	-7.316759	0.569568	-0.116296
H	-6.016252	-3.081739	0.417617
H	-8.451473	-2.613375	0.315298
H	-9.270315	-0.308143	-0.022079
H	-7.690086	1.575514	-0.263801
C	-4.980852	2.593347	-0.362030
H	-6.032722	2.858011	-0.414785
H	-4.497589	2.885734	-1.296107
H	-4.525019	3.130658	0.471689
C	-3.445135	-1.477730	1.519089
H	-3.831494	-0.859976	2.333153
H	-3.776870	-2.505763	1.682475
H	-2.355292	-1.464818	1.563252
C	-3.407223	-1.840479	-1.000899
H	-3.765068	-1.478168	-1.967437
H	-2.316484	-1.839619	-1.014028
H	-3.741134	-2.873456	-0.878160
C	-2.467736	1.174511	-0.128107
C	-1.224794	0.583845	-0.029708
C	-0.011894	1.291973	-0.111716
C	1.194676	0.642842	-0.003826
C	2.480478	1.237304	-0.037035
H	-2.495332	2.248214	-0.276316
H	-1.148521	-0.488497	0.117988
H	-0.043325	2.368202	-0.258367
H	1.162192	-0.434003	0.145351
C	3.655304	0.445209	-0.019321
S	3.617623	-1.240198	-0.464561
C	5.343892	-1.376587	-0.226177
C	5.883123	-0.144960	0.143621
N	4.900961	0.847125	0.288860
C	6.145822	-2.500518	-0.388260
C	7.512196	-2.361012	-0.182492
C	8.057517	-1.123486	0.174675
C	7.254822	-0.002663	0.341813
H	5.716689	-3.454372	-0.672334
H	8.161377	-3.220419	-0.306660
H	9.127752	-1.031851	0.322208
H	7.693660	0.950886	0.607416
C	5.223973	2.148870	0.875549
H	6.076710	2.022450	1.538853
H	4.380947	2.496240	1.467925
H	5.462931	2.880376	0.102688
C	2.554325	2.646795	-0.209954
N	2.551104	3.794209	-0.388121

6 (E_{SCF} = −1554.47117387 a.u.)

C	3.750223	0.492156	0.032018
N	4.969844	1.104008	0.073772
C	6.017011	0.176139	-0.042160
C	5.478161	-1.102776	-0.168679
C	3.964478	-1.019380	-0.133107
C	6.311865	-2.198034	-0.299851
C	7.696852	-2.000038	-0.302471
C	8.220873	-0.715942	-0.174294
C	7.387514	0.397356	-0.041503
H	5.903831	-3.199417	-0.399718
H	8.363916	-2.849270	-0.404503

H	9.296300	-0.571069	-0.176955
H	7.813166	1.388840	0.057226
C	5.148408	2.533669	0.221300
H	6.209467	2.770036	0.216924
H	4.718358	2.880089	1.165511
H	4.670168	3.067794	-0.604640
C	3.372570	-1.540617	-1.455883
H	3.755018	-0.971118	-2.306536
H	3.653226	-2.588360	-1.592422
H	2.283000	-1.480321	-1.465107
C	3.417348	-1.810541	1.069639
H	3.831975	-1.432623	2.007488
H	2.328977	-1.757822	1.130004
H	3.698207	-2.862466	0.971645
C	2.576243	1.198780	0.131422
C	1.267897	0.653667	0.099819
C	0.117677	1.390563	0.212611
C	-1.156934	0.763676	0.175907
C	-2.395247	1.355843	0.222028
H	2.643081	2.275562	0.248537
H	1.152301	-0.419384	-0.020173
H	0.195147	2.467264	0.325928
H	-1.120874	-0.315142	0.042587
C	-2.569179	2.869773	0.301422
C	-3.538214	0.490931	0.081607
S	-4.886047	0.880897	-0.941303
C	-5.656171	-0.642440	-0.584808
C	-4.860412	-1.392130	0.280128
N	-3.697933	-0.708156	0.662431
C	-6.870712	-1.132630	-1.057256
C	-7.263514	-2.398070	-0.646863
C	-6.460366	-3.155559	0.215384
C	-5.252852	-2.665924	0.690173
H	-7.487001	-0.544643	-1.727292
H	-8.202895	-2.806248	-1.002565
H	-6.784903	-4.144920	0.517627
H	-4.635419	-3.263398	1.349209
C	-2.857101	-1.243351	1.737480
H	-3.510063	-1.650633	2.508745
H	-2.261907	-0.438145	2.158770
H	-2.201830	-2.029559	1.359623
H	-1.634812	3.239465	0.730075
C	-3.677229	3.308929	1.266461
C	-2.715569	3.538469	-1.074097
H	-3.641389	4.394069	1.396984
H	-4.675717	3.058441	0.898468
H	-3.551375	2.846224	2.249569
H	-2.684701	4.626545	-0.963580
H	-1.906268	3.240347	-1.746069
H	-3.665308	3.289425	-1.556504

5 (E_{SCF} = −1274.15456152 a.u.)

C	3.832614	0.482283	-0.021149
N	5.030215	1.103564	-0.116599
C	6.098184	0.188368	-0.025108
C	5.578002	-1.091433	0.143214
C	4.064509	-1.022242	0.163155
C	6.428981	-2.175660	0.260299
C	7.809152	-1.959054	0.206146
C	8.313699	-0.671389	0.036289
C	7.463040	0.429434	-0.082947
H	6.038196	-3.180059	0.391604
H	8.490554	-2.798130	0.296277
H	9.386534	-0.515162	-0.004892
H	7.871593	1.424026	-0.215168
C	5.192240	2.535525	-0.292327
H	6.250913	2.778681	-0.320388
H	4.732699	3.075050	0.539438
H	4.733495	2.859575	-1.229866
C	3.479105	-1.839711	-1.003936
H	3.851331	-1.474701	-1.964200
H	3.777807	-2.885540	-0.897054
H	2.388825	-1.801684	-1.020884
C	3.526270	-1.520545	1.518079
H	3.929286	-0.927063	2.342268
H	2.436990	-1.477353	1.563741
H	3.828352	-2.560417	1.665803
C	2.638961	1.182878	-0.090980
C	1.356084	0.627378	0.001329
C	0.180489	1.357388	-0.075325
C	-1.060114	0.714957	0.028048
C	-2.349131	1.248589	0.008032
H	2.694267	2.257288	-0.229710
H	1.253962	-0.444428	0.141284
H	0.251068	2.428768	-0.212825
H	-0.980142	-0.353112	0.183048
C	-2.609447	2.732581	-0.271207
C	-3.427766	0.338251	0.133801
C	-3.433235	-1.162361	-0.251320
C	-4.915143	-1.470316	-0.214530
C	-5.598934	-0.365437	0.267264
N	-4.675753	0.676610	0.513761
C	-5.607628	-2.627966	-0.524538
C	-6.995272	-2.644054	-0.357408
C	-7.665649	-1.521124	0.125381
C	-6.973021	-0.353803	0.451234
H	-5.092087	-3.509032	-0.893613
H	-7.555594	-3.538768	-0.606581
H	-8.743023	-1.549320	0.248669
H	-7.499333	0.518656	0.819967
C	-2.748967	-2.052796	0.815555
H	-3.000616	-3.096119	0.611622
H	-1.662088	-1.971252	0.803721

H	-3.105257	-1.807891	1.819074
C	-2.851518	-1.409939	-1.650520
H	-1.783811	-1.190061	-1.691753
H	-2.990460	-2.459479	-1.921482
H	-3.357532	-0.794403	-2.398209
C	-5.097601	1.843475	1.279854
H	-5.622195	2.569183	0.654672
H	-4.237071	2.309194	1.749747
H	-5.772828	1.500878	2.065561
H	-3.688840	2.871494	-0.309038
C	-2.073769	3.699152	0.795230
C	-2.127449	3.125689	-1.679160
H	-2.412825	4.716090	0.576688
H	-2.423401	3.436511	1.797618
H	-0.982924	3.717974	0.829508
H	-2.431764	4.154241	-1.895738
H	-1.042659	3.071323	-1.786733
H	-2.572773	2.475125	-2.437042

9 (E_{SCF} = −1340.69432574 a.u.)

C	7.177539	-0.719292	0.545409
C	5.865753	-0.441438	0.201893
C	5.098287	-1.411524	-0.424251
C	5.574046	-2.677781	-0.721244
C	6.895161	-2.951679	-0.365084
C	7.687049	-1.988008	0.258477
N	3.804468	-0.877008	-0.680160
C	3.689564	0.351702	-0.185238
C	5.042580	0.814637	0.364488
C	2.484216	1.103405	-0.130448
C	1.198661	0.537750	-0.093960
C	0.000004	1.233835	0.000014
C	-1.198650	0.537744	0.093981
C	-2.484207	1.103395	0.130470
C	-3.689558	0.351694	0.185247
N	-3.804482	-0.877010	0.680180
C	-5.098301	-1.411517	0.424251
C	-5.865749	-0.441429	-0.201911
C	-5.042563	0.814638	-0.364498
C	-7.177531	-0.719274	-0.545448
C	-7.687057	-1.987984	-0.258517
C	-6.895187	-2.951658	0.365064
C	-5.574076	-2.677768	0.721245
C	-2.847856	-1.609639	1.502864
C	-4.935056	1.242902	-1.837432
C	-5.640684	1.946559	0.501513
C	5.640699	1.946550	-0.501534
C	4.935096	1.242906	1.837423
C	2.847810	-1.609635	-1.502808
H	-4.959972	-3.433260	1.196352
H	-7.307299	-3.932540	0.575263
H	-8.710084	-2.227649	-0.527442
H	-7.800369	0.024364	-1.031993
H	-5.934087	1.470455	-2.216148
H	-4.321459	2.138567	-1.947151
H	-4.509116	0.446256	-2.451563
H	-6.659236	2.142814	0.159354
H	-5.068011	2.868928	0.408588
H	-5.683770	1.658700	1.554552
H	-2.183959	-0.907623	2.003112
H	-2.269563	-2.315938	0.903791
H	-3.405918	-2.158978	2.260080
C	-2.565016	2.521953	-0.022153
H	-1.131730	-0.543630	0.081849
H	0.000002	2.318930	0.000020
H	1.131747	-0.543625	-0.081842
C	2.565025	2.521963	0.022180
H	5.683769	1.658688	-1.554573
H	4.959928	-3.433271	-1.196335
H	7.307261	-3.932566	-0.575284
H	8.710079	-2.227680	0.527387
H	7.800391	0.024343	1.031940
H	3.405844	-2.158984	-2.260037
H	2.183903	-0.907616	-2.003039
H	2.269529	-2.315925	-0.903712
H	5.068036	2.868924	-0.408603
H	6.659257	2.142797	-0.159389
H	4.321506	2.138574	1.947147
H	5.934134	1.470454	2.216124
H	4.509160	0.446265	2.451563
N	2.560039	3.675743	0.136971
N	-2.560021	3.675729	-0.136989

10 (E_{SCF} = −1233.66023275 a.u.)

C	8.923278	-1.794853	0.014363
C	9.407192	-0.488277	-0.011903
C	8.538673	0.604507	-0.030098
C	7.178007	0.335232	-0.020943
C	6.678100	-0.962964	0.005570
C	7.546959	-2.039447	0.023304
C	5.163876	-0.919866	0.010526
C	4.616940	-1.564768	1.298472
C	4.606962	-1.617933	-1.245055
C	4.909352	0.591296	-0.017424
N	6.094043	1.237802	-0.035072
C	6.234460	2.683435	-0.064121

C	3.701731	1.277600	-0.021228
C	2.431659	0.695724	-0.009209
C	1.241938	1.412725	-0.007331
C	0.002914	0.775791	0.000049
C	-1.235438	1.414075	0.007164
C	-2.425915	0.698361	0.009196
C	-3.695376	1.281624	0.020876
C	-4.903765	0.596683	0.017137
N	-6.087762	1.244517	0.034348
C	-5.159994	-0.914207	-0.010304
C	-4.613424	-1.560333	-1.297778
C	-6.674260	-0.955607	-0.005771
C	-4.604279	-1.612338	1.245779
C	-6.226598	2.690305	0.062887
C	-7.172716	0.343154	0.020213
C	-8.533076	0.613955	0.028895
C	-9.402818	-0.477859	0.010792
C	-8.920363	-1.784986	-0.014935
C	-7.544314	-2.031123	-0.023419
H	9.618600	-2.627199	0.028196
H	10.477480	-0.311067	-0.018348
H	8.930499	1.614223	-0.050200
H	7.173396	-3.058605	0.044082
H	3.526935	-1.536928	1.335692
H	4.929132	-2.611244	1.338555
H	5.004903	-1.058131	2.185454
H	4.922710	-2.664152	-1.245397
H	3.516557	-1.596327	-1.273507
H	4.984868	-1.146786	-2.155581
H	7.289227	2.943150	-0.086408
H	5.754869	3.093737	-0.955928
H	5.783510	3.127971	0.826354
H	3.740470	2.361540	-0.034041
H	2.342899	-0.386202	-0.000659
H	1.277591	2.499997	-0.011789
H	0.002318	-0.314332	0.000253
H	-1.269907	2.501388	0.011218
H	-2.338306	-0.383663	0.001071
H	-3.732911	2.365608	0.033275
H	-3.523376	-1.533858	-1.334666
H	-4.926888	-2.606443	-1.337508
H	-5.000479	-1.053615	-2.185110
H	-3.513867	-1.591819	1.274637
H	-4.982051	-1.140417	2.155957
H	-4.921114	-2.658230	1.246434
H	-5.775288	3.134046	-0.827808
H	-7.281097	2.951155	0.085223
H	-5.746469	3.100399	0.954491
H	-8.923757	1.624125	0.048550
H	-10.472908	-0.299443	0.016872
H	-9.616616	-2.616556	-0.028709
H	-7.171874	-3.050702	-0.043779

16 (E_{SCF} = −1325.86123500 a.u.)

C	8.848024	-1.899212	0.123347
C	9.353391	-0.607638	-0.021192
C	8.502748	0.493797	-0.116802
C	7.140511	0.243633	-0.061258
C	6.618171	-1.035923	0.081671
C	7.470311	-2.122649	0.175762
C	5.106982	-0.962318	0.105302
C	4.565832	-1.483233	1.451755
C	4.514089	-1.747866	-1.081364
C	4.884803	0.542873	-0.046820
N	6.063114	1.161747	-0.132592
C	6.234888	2.601627	-0.281733
C	3.674458	1.259956	-0.102073
C	2.416670	0.708865	-0.018462
C	1.223514	1.461535	-0.082554
C	-0.007669	0.867749	0.006670
C	-1.247645	1.557742	-0.053911
C	-2.434567	0.892653	0.045670
C	-3.744404	1.478876	0.009165
C	-4.926774	0.733675	0.065765
N	-6.162171	1.251602	0.221562
C	-5.044847	-0.794529	-0.093347
C	-4.408874	-1.298189	-1.400165
C	-6.548238	-0.967695	-0.139428
C	-4.494970	-1.545858	1.139447
C	-6.505344	2.608349	0.624864
C	-7.159287	0.262908	0.052633
C	-8.536872	0.417828	0.062071
C	-9.307658	-0.731975	-0.121670
C	-8.711062	-1.977554	-0.306750
C	-7.318866	-2.103259	-0.314784
H	9.531381	-2.738334	0.195408
H	10.425897	-0.451097	-0.060595
H	8.908961	1.491479	-0.229109
H	7.083027	-3.130345	0.287665
H	3.477304	-1.434016	1.499114
H	4.861481	-2.527751	1.574248
H	4.974522	-0.911375	2.288074
H	4.810755	-2.795844	-0.997782
H	3.424213	-1.706766	-1.093176
H	4.885260	-1.362300	-2.033782
H	7.295188	2.833783	-0.310117
H	5.775108	2.940766	-1.211736
H	5.783998	3.122812	0.564427
H	3.738432	2.335594	-0.222620

H	2.301810	-0.363198	0.103782
H	1.293342	2.539669	-0.206675
H	-0.046797	-0.214219	0.131294
H	-1.228624	2.636636	-0.182308
H	-2.381677	-0.178967	0.183262
C	-3.769723	2.892293	-0.190729
H	-3.324385	-1.185177	-1.404629
H	-4.634828	-2.360334	-1.521534
H	-4.813777	-0.763062	-2.262371
H	-3.410004	-1.480785	1.223241
H	-4.935655	-1.159061	2.061433
H	-4.758404	-2.602633	1.053577
H	-6.607374	3.272659	-0.235241
H	-7.449302	2.573664	1.166588
H	-5.743168	2.995859	1.297669
H	-9.010571	1.383193	0.193659
H	-10.389180	-0.647719	-0.123676
H	-9.331109	-2.855883	-0.449940
H	-6.858913	-3.075603	-0.460785
N	-3.699013	4.031421	-0.402550

17 (E_{SCF} = −1418.05737639 a.u.)

C	8.583624	-2.389846	0.527792
C	9.206309	-1.228012	0.073404
C	8.461197	-0.104659	-0.286892
C	7.084126	-0.202544	-0.169060
C	6.446772	-1.344726	0.289360
C	7.192618	-2.457970	0.636390
C	4.950913	-1.118735	0.289912
C	4.366628	-1.319480	1.698504
C	4.305124	-2.074050	-0.740106
C	4.877960	0.351672	-0.156106
N	6.103140	0.779079	-0.467370
C	6.469801	2.032777	-1.118697
C	3.717340	1.163118	-0.168409
C	2.409521	0.639367	-0.097507
C	1.235227	1.369732	-0.051894
C	-0.000020	0.724809	-0.000400
C	-1.235339	1.369753	0.051510
C	-2.409574	0.639429	0.096894
C	-3.717476	1.163211	0.168281
C	-4.878056	0.351872	0.155888
N	-6.103384	0.779313	0.466815
C	-4.950878	-1.118713	-0.289555
C	-4.366280	-1.320248	-1.697888
C	-6.446726	-1.344784	-0.289241
C	-4.305376	-2.073377	0.741254
C	-6.470142	2.033261	1.117587
C	-7.084229	-0.202484	0.168699
C	-8.461339	-0.104722	0.286243
C	-9.206288	-1.228272	-0.073790
C	-8.583437	-2.390202	-0.527685
C	-7.192402	-2.458210	-0.636034
H	9.185646	-3.248885	0.803336
H	10.287734	-1.190680	-0.000090
H	8.952737	0.797273	-0.630763
H	6.715572	-3.366052	0.990759
H	3.289116	-1.151371	1.721671
H	4.553125	-2.346322	2.020874
H	4.836794	-0.644695	2.417589
H	4.567155	-3.100251	-0.473578
H	3.217419	-2.007138	-0.753876
H	4.678745	-1.878058	-1.747768
H	7.352041	1.849073	-1.729446
H	5.664025	2.356975	-1.772368
H	6.689871	2.810389	-0.385694
C	3.823744	2.588683	-0.142773
H	2.306774	-0.437774	-0.098032
H	1.264039	2.455383	-0.056287
H	-0.000041	-0.364559	-0.000736
H	-1.264076	2.455403	0.056477
H	-2.306835	-0.437712	0.096726
C	-3.823593	2.588828	0.143422
H	-3.288721	-1.152452	-1.720943
H	-4.552968	-2.347183	-2.019850
H	-4.836111	-0.645671	-2.417387
H	-3.217722	-2.005875	0.755862
H	-4.679846	-1.877140	1.748552
H	-4.566732	-3.099805	0.474936
H	-6.688688	2.811037	0.384308
H	-7.353331	1.850184	1.727130
H	-5.665048	2.356931	1.772387
H	-8.953069	0.797274	0.629676
H	-10.287729	-1.190999	-0.000488
H	-9.185329	-3.249394	-0.803039
H	-6.715199	-3.366357	-0.990026
N	-3.836464	3.746300	0.072218
N	3.837014	3.746106	-0.070880

14 (E_{SCF} = −1631.83428615a.u.)

C	-9.006863	-1.120050	1.449338
C	-9.198248	-0.894548	0.079429
C	-8.210158	-0.312880	-0.699706
C	-7.015416	0.043733	-0.074439
C	-6.828650	-0.174207	1.289786
C	-7.820189	-0.762542	2.070968
S	-5.259872	0.404039	1.780878
C	-4.887083	0.898841	0.163143

N	-5.888787	0.619261	-0.679133
C	-5.825356	0.748046	-2.138584
C	-3.648368	1.563352	-0.181297
C	-3.741592	2.683867	-0.960622
C	-2.686234	3.550034	-1.376741
C	-2.938702	4.672099	-2.111752
C	-1.952349	5.590502	-2.574462
C	-2.266913	6.699260	-3.305540
C	-1.312215	7.640678	-3.788461
C	-1.579217	8.768418	-4.517105
N	-0.587434	9.615552	-4.941088
C	-2.930101	9.320262	-4.996551
C	-3.631443	8.355054	-5.970013
C	-3.851676	9.682042	-3.817212
C	0.817686	9.404010	-4.669116
C	-1.109012	10.699214	-5.659595
C	-0.440459	11.766307	-6.245597
C	-1.208726	12.723653	-6.912999
C	-2.594958	12.614054	-6.988723
C	-3.247201	11.530314	-6.389698
C	-2.496705	10.577293	-5.726675
H	-9.798093	-1.576831	2.033106
H	-10.137203	-1.177549	-0.382802
H	-8.372179	-0.135680	-1.755514
H	-7.668165	-0.931424	3.130644
H	-6.245028	-0.155796	-2.578308
H	-6.395382	1.617176	-2.469655
H	-4.787827	0.848633	-2.443498
C	-2.325741	1.069252	0.396567
H	-4.733774	3.000562	-1.275379
H	-1.660196	3.324688	-1.103467
H	-3.974452	4.893337	-2.371794
H	-0.910914	5.389319	-2.329163
H	-3.318121	6.860869	-3.525173
H	-0.275346	7.430388	-3.545371
H	-3.943384	7.434316	-5.473928
H	-2.972299	8.090881	-6.800755
H	-4.523652	8.834795	-6.381420
H	-4.748437	10.181777	-4.193408
H	-4.167438	8.796356	-3.263124
H	-3.349225	10.360055	-3.122805
H	1.008663	9.407963	-3.591615
H	1.150374	8.447640	-5.083521
H	1.401445	10.199086	-5.126715
H	0.637077	11.869548	-6.196174
H	-0.709528	13.567049	-7.379134
H	-3.170624	13.369855	-7.512198
H	-4.328316	11.444881	-6.448055
H	-1.556392	1.487403	-0.256480
C	-2.043054	1.603941	1.808821
C	-2.163255	-0.453918	0.323424
H	-1.143367	-0.728484	0.606507
H	-2.841745	-0.978397	1.001708
H	-2.343048	-0.822882	-0.690470
H	-1.024466	1.339748	2.108128
H	-2.138861	2.692370	1.845708
H	-2.719668	1.178161	2.555568

15 (E_{SCF} = −1671.11811314 a.u.)

C	-9.174405	-2.160298	-0.153101
C	-9.692560	-0.870198	-0.075868
C	-8.854749	0.246102	-0.006823
C	-7.482917	0.025348	-0.018072
C	-6.950380	-1.262412	-0.095057
C	-7.788670	-2.359253	-0.162887
C	-5.434842	-1.185143	-0.088361
C	-4.867447	-1.770334	-1.394591
C	-4.870511	-1.925723	1.137889
C	-5.208919	0.331983	0.004241
N	-6.438029	0.952816	0.039466
C	-6.605500	2.385809	0.124917
C	-4.038369	1.031448	0.048480
C	-2.714400	0.485202	0.017585
C	-1.577700	1.228904	0.064682
C	-0.267198	0.647422	0.029053
C	0.898180	1.341923	0.064094
C	2.169666	0.663789	0.013757
C	3.431336	1.161551	0.053167
C	4.488030	0.136885	-0.054730
N	5.267802	-0.293498	0.927421
S	4.828041	-0.643060	-1.544273
C	6.089414	-1.593664	-0.807200
C	5.137900	0.125180	2.325047
C	6.199591	-1.267650	0.545369
C	7.141753	-1.889450	1.365159
C	7.963926	-2.841793	0.787252
C	7.853475	-3.172732	-0.572126
C	6.917023	-2.555734	-1.384965
H	-9.844171	-3.011956	-0.205881
H	-10.767716	-0.721892	-0.068681
H	-9.277735	1.242037	0.052830
H	-7.383384	-3.365148	-0.223262
H	-3.778048	-1.710446	-1.425557
H	-5.149791	-2.823457	-1.478315
H	-5.264485	-1.240449	-2.264152
H	-5.154576	-2.980708	1.092365
H	-3.781027	-1.872410	1.177803
H	-5.268404	-1.505075	2.064878
H	-7.665118	2.630246	0.136100

H	-6.150210	2.775556	1.040922
H	-6.144592	2.881937	-0.735112
H	-4.098626	2.113596	0.113934
H	-2.596337	-0.592489	-0.048151
H	-1.649745	2.313552	0.130176
H	-0.218414	-0.440351	-0.033969
H	0.857152	2.422892	0.127188
H	2.080518	-0.420099	-0.052377
C	3.849953	2.650266	0.076025
H	5.003769	-0.761618	2.944709
H	6.034781	0.662889	2.634058
H	4.265128	0.764545	2.418975
H	7.233330	-1.637398	2.414433
H	8.707636	-3.340139	1.398586
H	8.512595	-3.923073	-0.994020
H	6.829998	-2.809388	-2.434906
C	3.320889	3.333218	-1.201508
C	3.302471	3.370233	1.322403
C	5.380176	2.810040	0.076284
H	5.625309	3.874326	0.033583
H	5.844656	2.411875	0.981726
H	5.844893	2.333796	-0.792012
H	3.587297	4.426171	1.286438
H	2.215615	3.320794	1.393520
H	3.716852	2.948215	2.242152
H	3.612869	4.387948	-1.203180
H	3.742313	2.864395	-2.095588
H	2.233849	3.284600	-1.278454

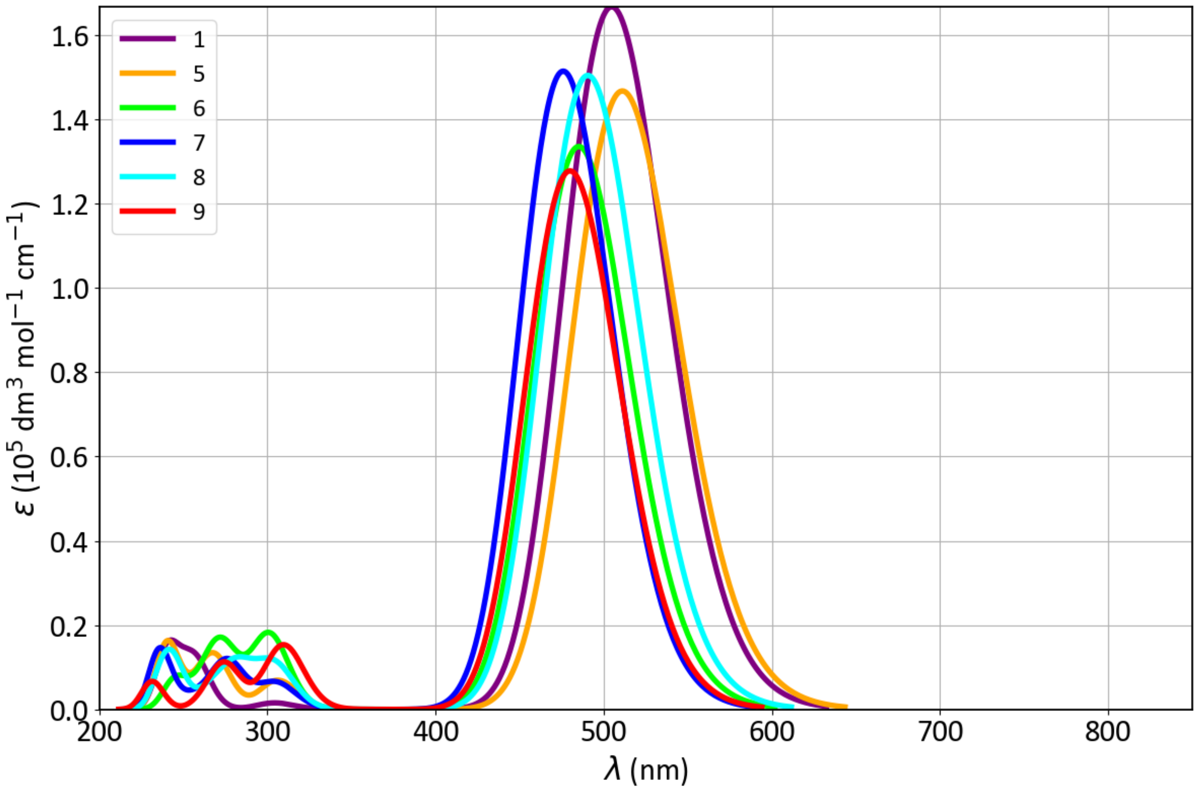

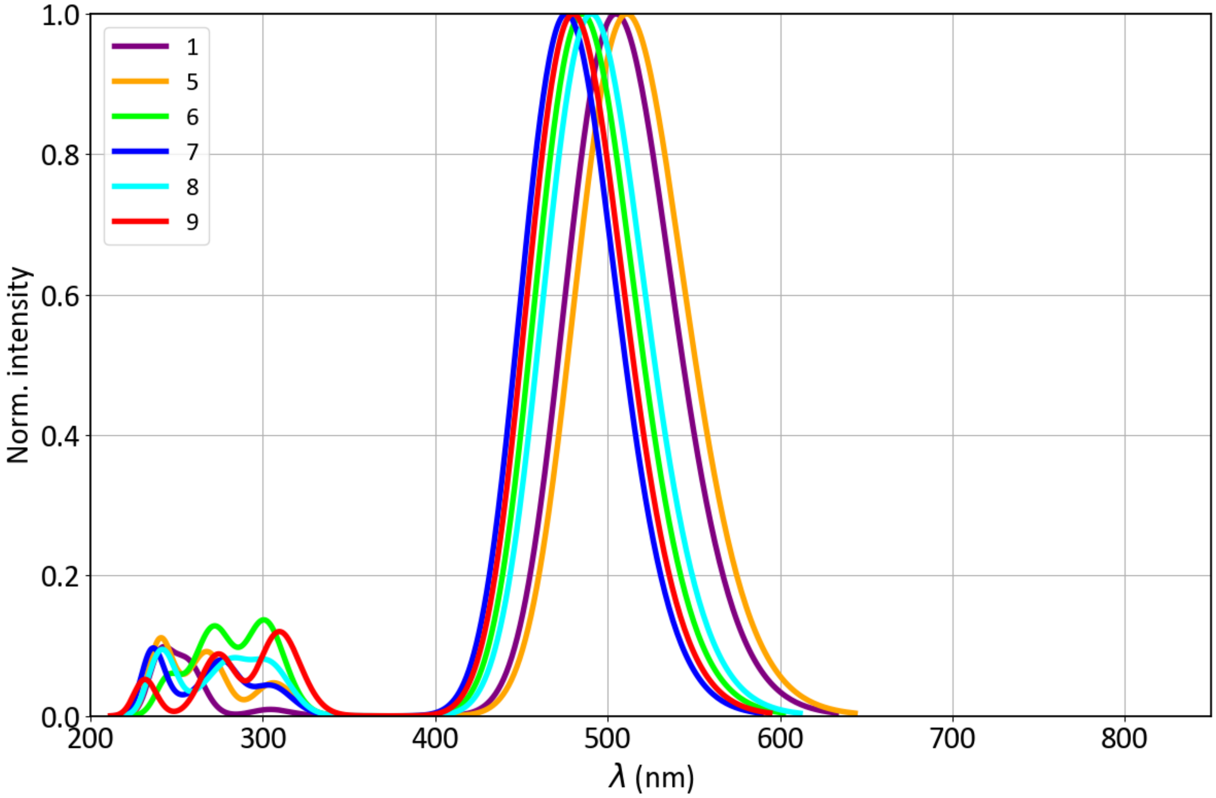

Figure S79. Calculated absorption spectra of **1**, **5**, **6**, **7**, **8**, and **9** (top: absolute intensity; bottom: normalized intensity) using IEF-PCM-TD-CAM-B3LYP/6-31+G**. These are purely electronic spectra as vibronic effects were not taken into account.

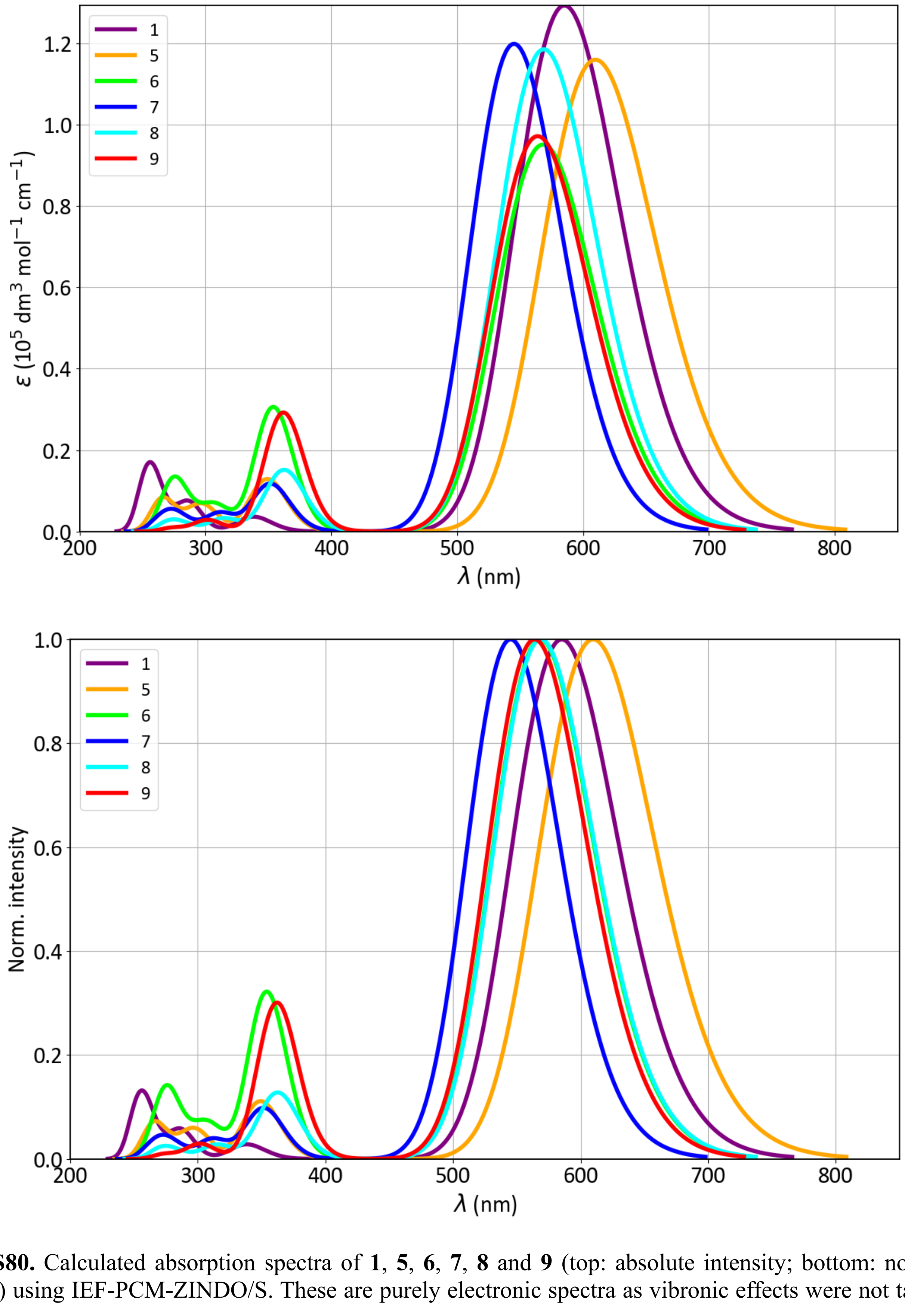

Figure S80. Calculated absorption spectra of **1**, **5**, **6**, **7**, **8** and **9** (top: absolute intensity; bottom: normalized intensity) using IEF-PCM-ZINDO/S. These are purely electronic spectra as vibronic effects were not taken into account.

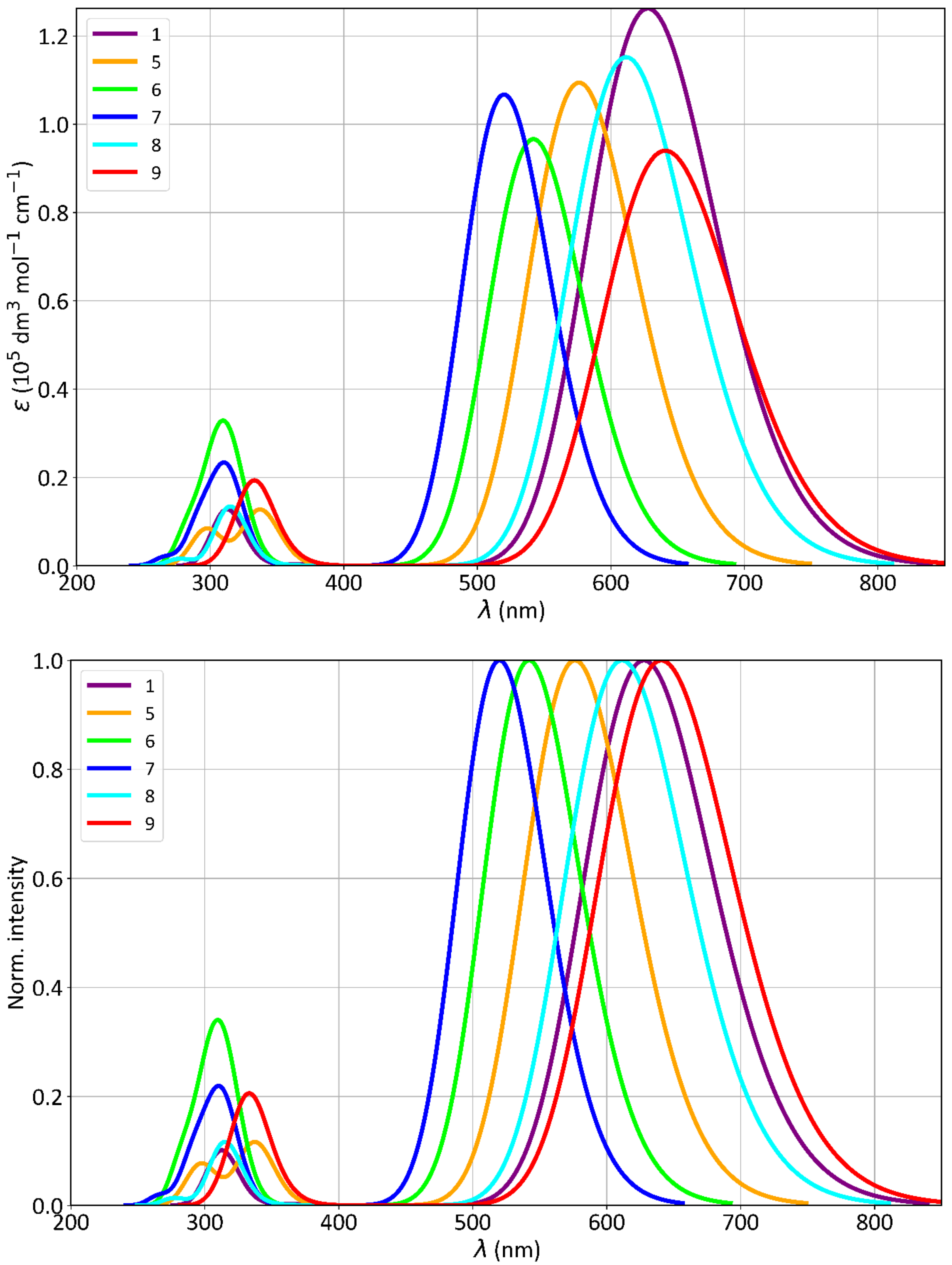

Figure S81. Calculated absorption spectra of **1**, **5**, **6**, **7**, **8** and **9** (top: absolute intensity; bottom: normalized intensity) using SC-NEVPT2(6/10)/cc-pVDZ. These are purely electronic spectra as vibronic effects were not taken into account.

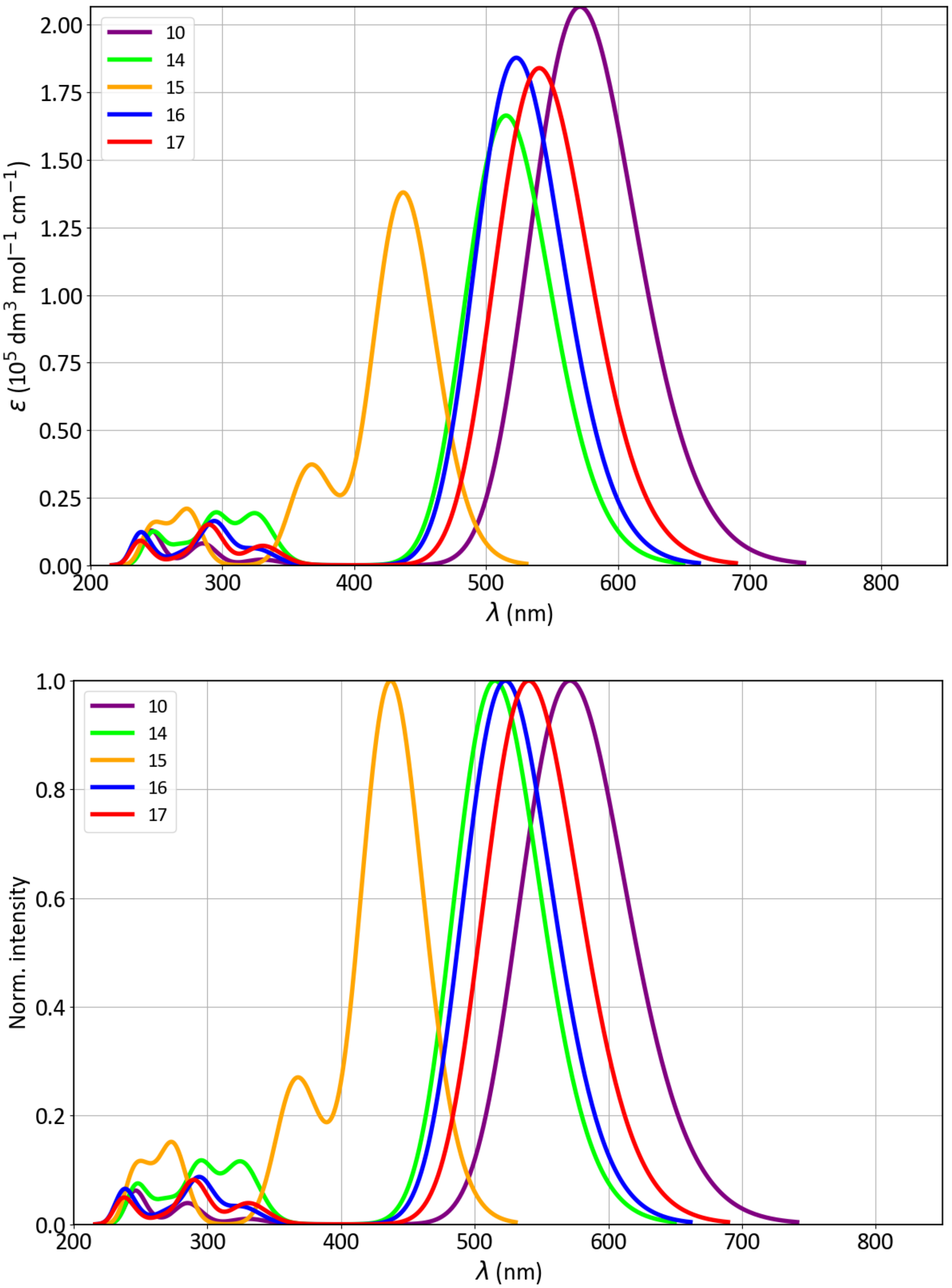

Figure S82. Calculated absorption spectra of **10**, **14**, **15**, 1**6** and **17** (top: absolute intensity; bottom: normalized intensity) using IEF-PCM-TD-CAM-B3LYP/6-31+G**. These are purely electronic spectra as vibronic effects were not taken into account.

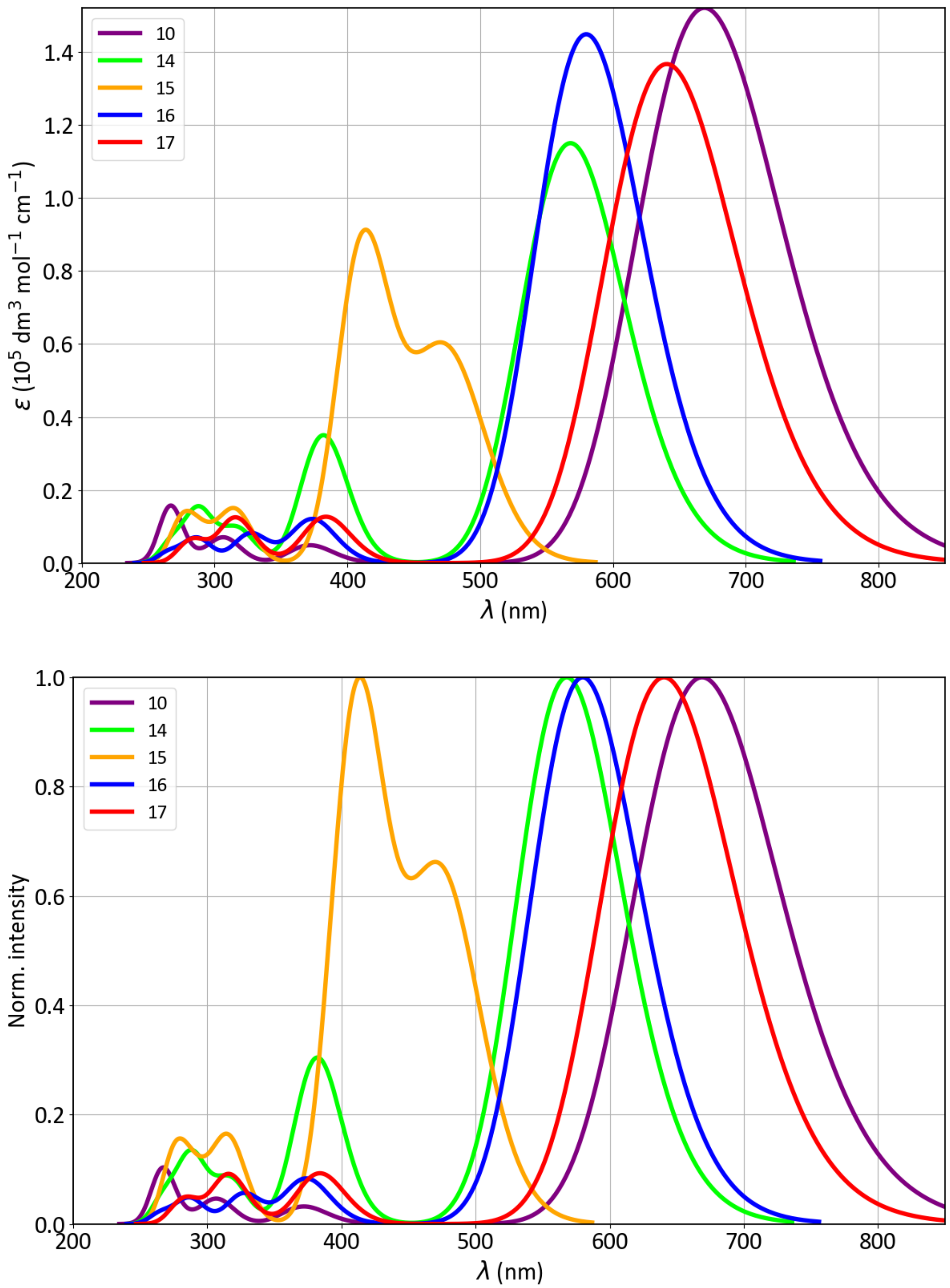

Figure S83. Calculated absorption spectra of **10**, **14**, **15**, **16** and **17** (top: absolute intensity; bottom: normalized intensity) using IEF-PCM-ZINDO/S. These are purely electronic spectra as vibronic effects were not taken into account.

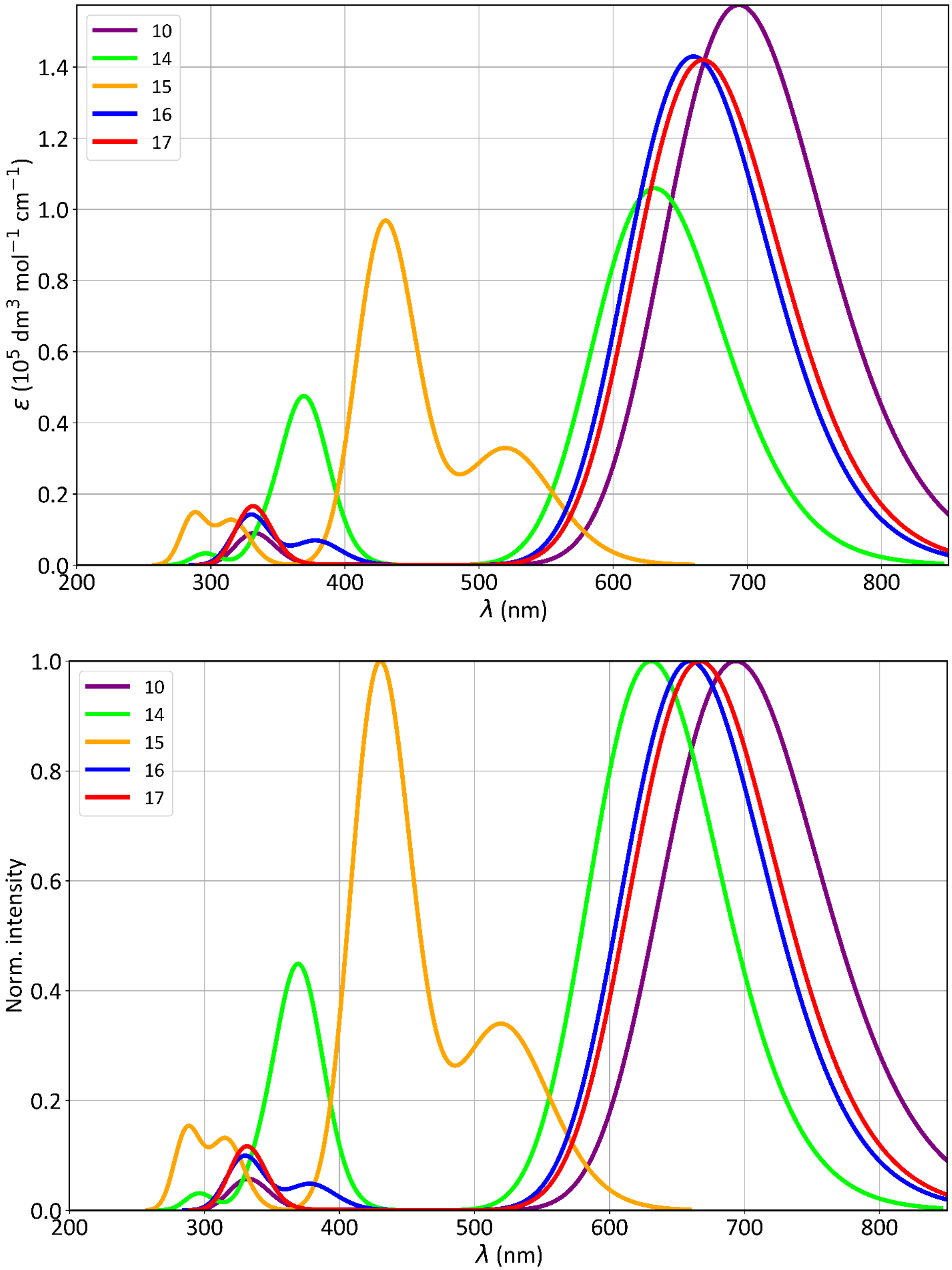

Figure S84. Calculated absorption spectra of **10**, **14**, **15**, **16** and **17** (top: absolute intensity; bottom: normalized intensity) using SC-NEVPT2(8/14)/cc-pVDZ. These are purely electronic spectra as vibronic effects were not taken into account.

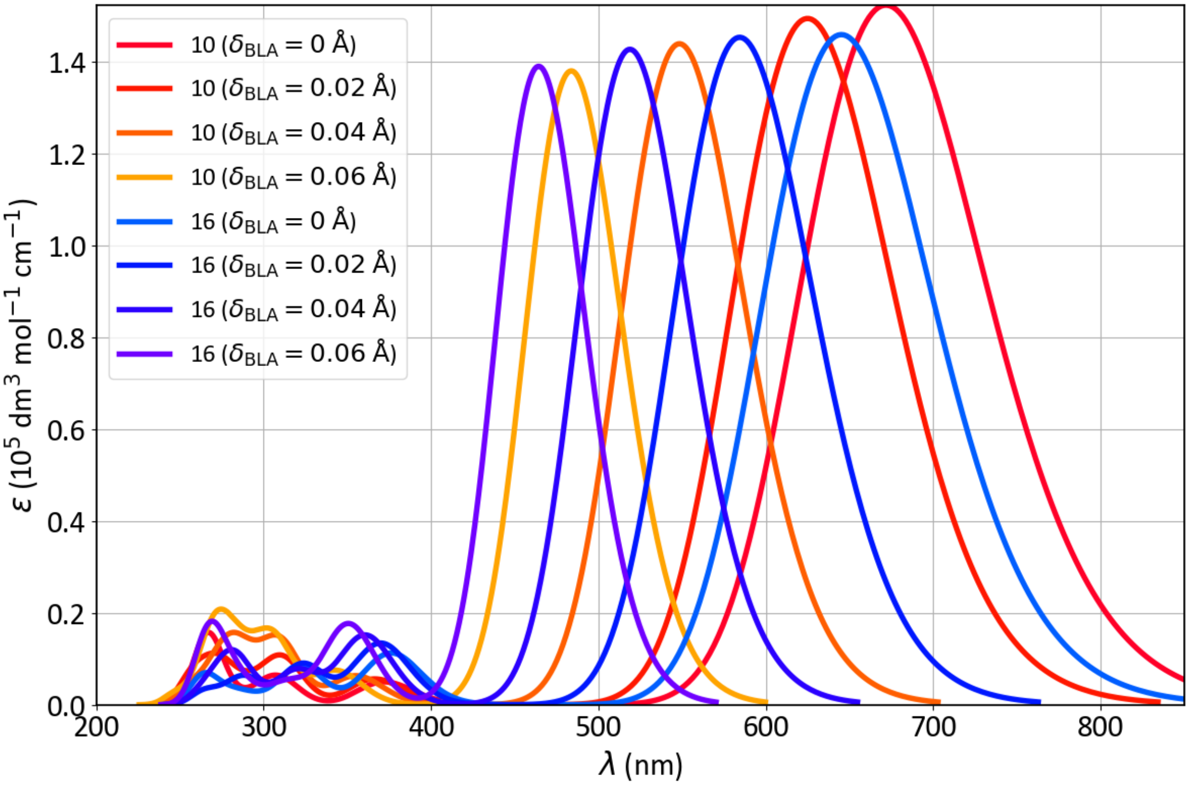

Figure S85. Calculated BLA spectral trend of **10** (red-ish) and **16** (blue-ish) using IEF-PCM-ZINDO/S.

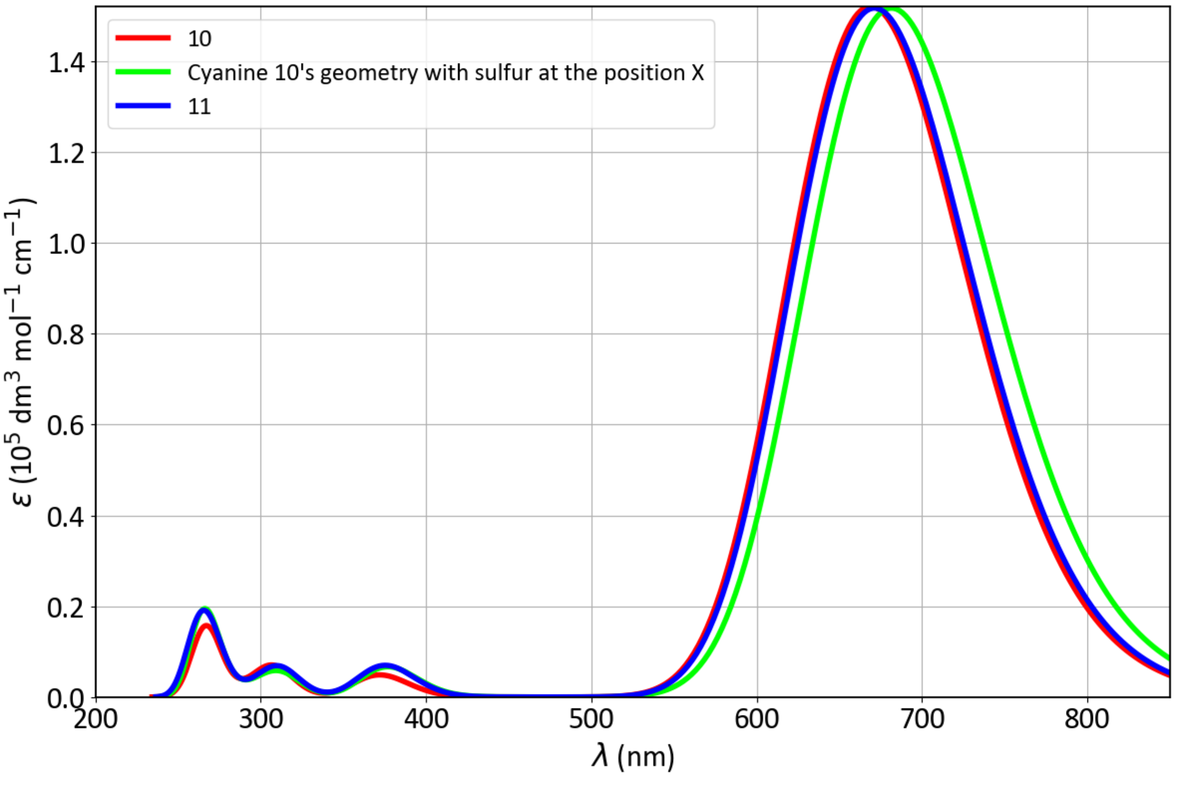

Figure S86. Calculated absorption spectra of **10** (red), **10** with sulfur at the position X (green) and **11** (blue) using IEF-PCM-ZINDO/S.

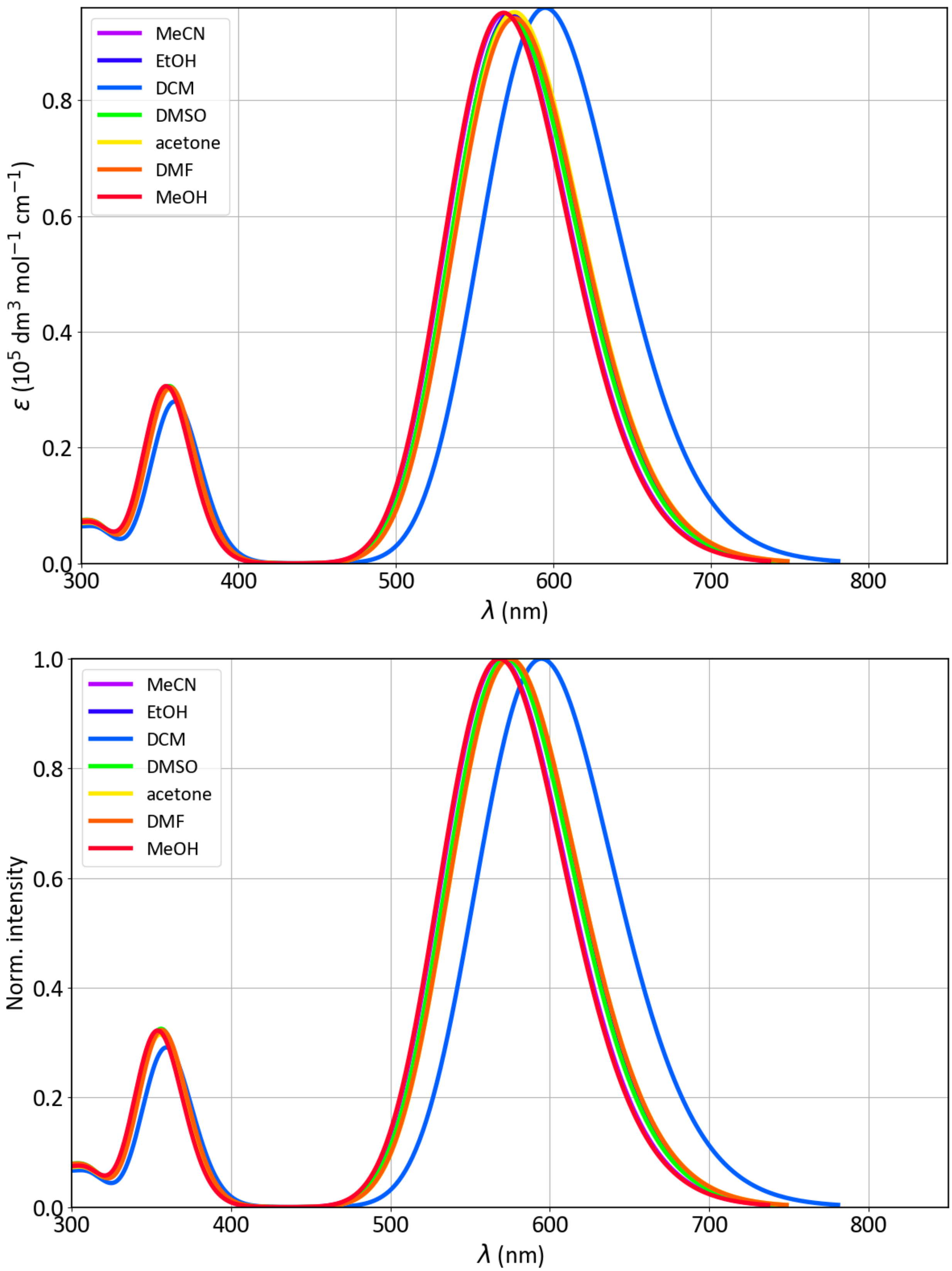

Figure S87. Calculated absorption spectra of **6** in different solvents (top: absolute intensity; bottom: normalized intensity) using IEF-PCM-ZINDO/S.

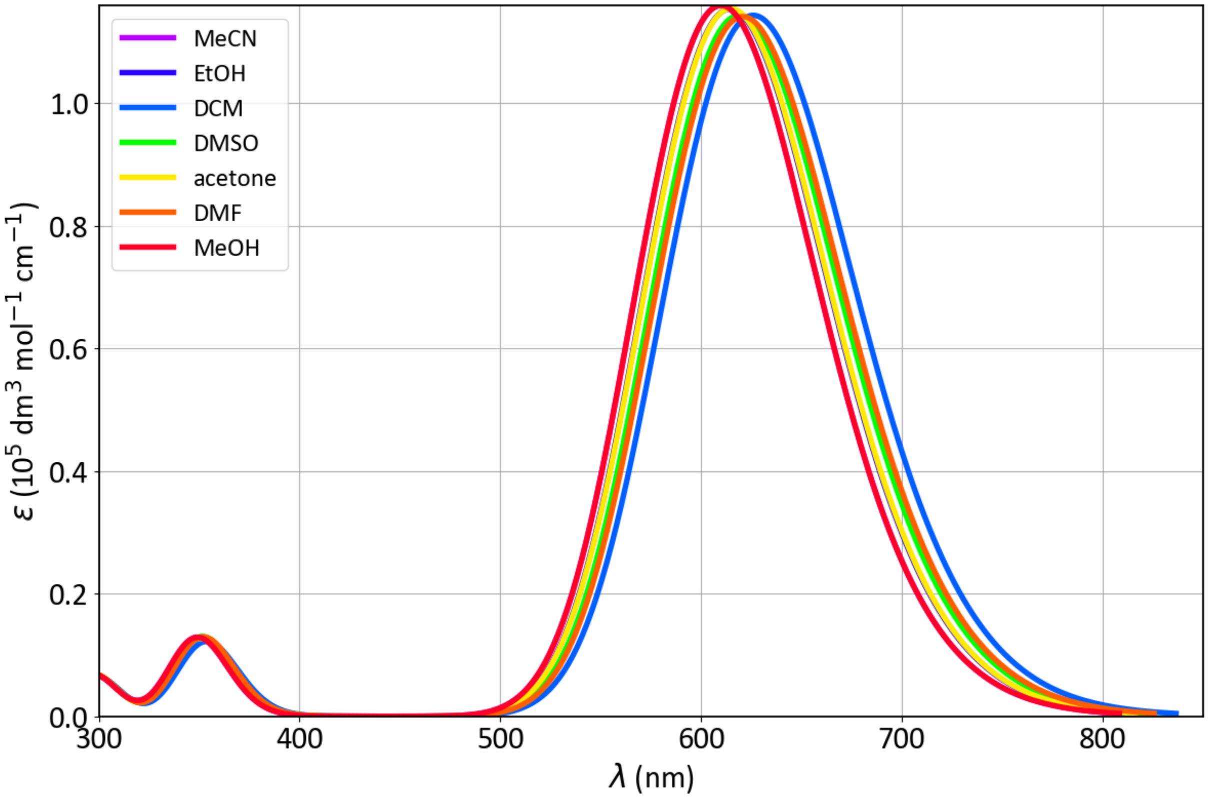

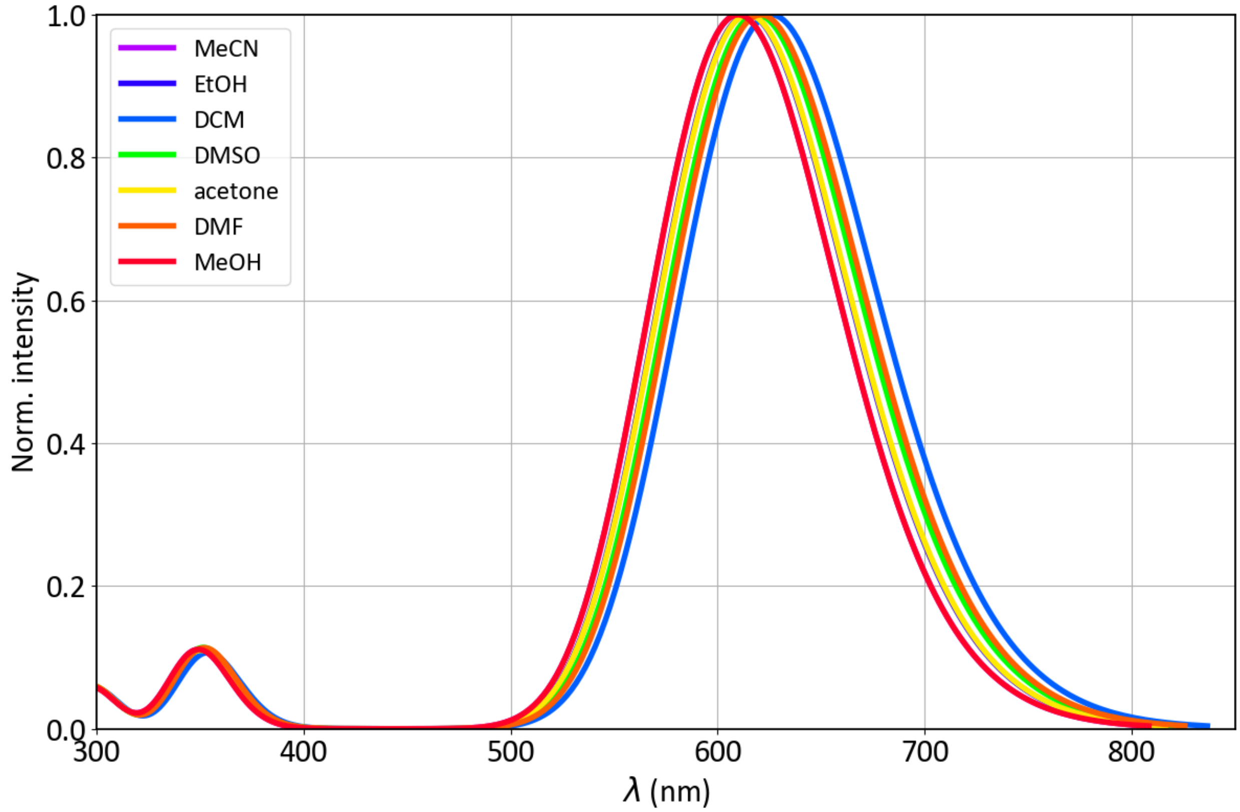

Figure S88. Calculated absorption spectra of **5** in different solvents (top: absolute intensity; bottom: normalized intensity) using IEF-PCM-ZINDO/S.

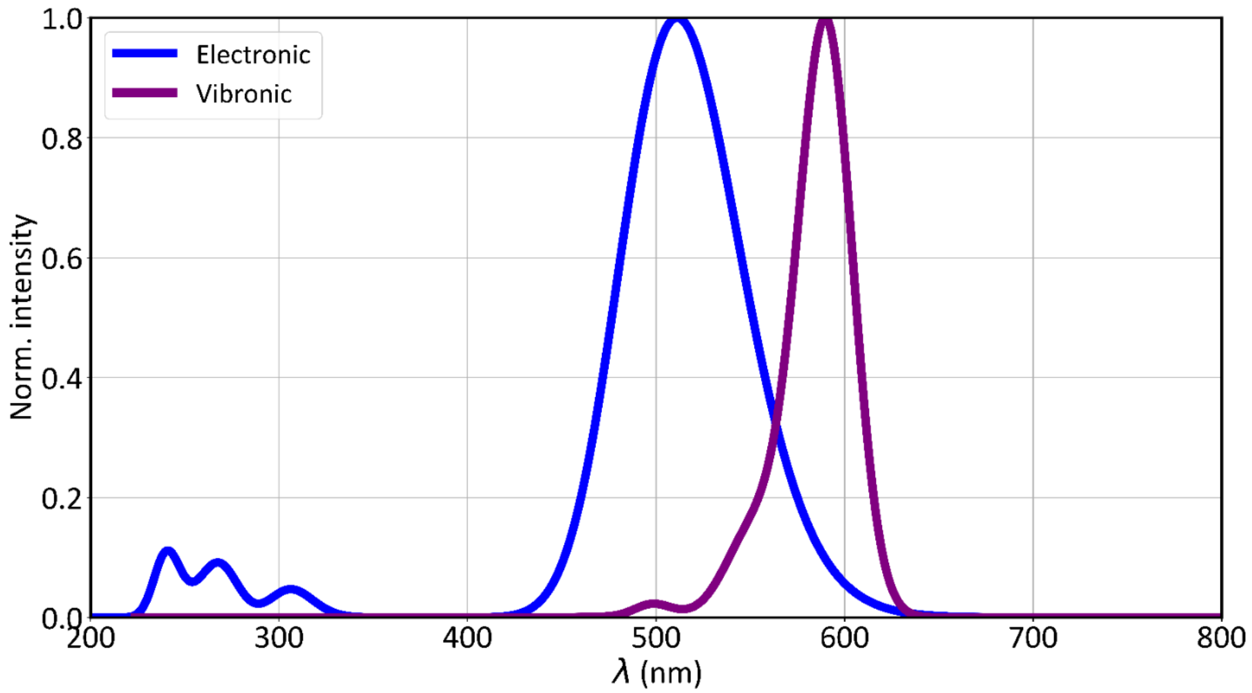

Figure S89. Calculated absorption spectra of **5** in methanol (purple: vibronic spectrum of the $S_0 \rightarrow S_1$ transition; blue: absorption spectrum (10 excited states) without including vibronic effects) using IEF-PCM-TD-CAM-B3LYP/6-31+G**. The difference in spectra indicates a structural difference between the ground and the first excited state.

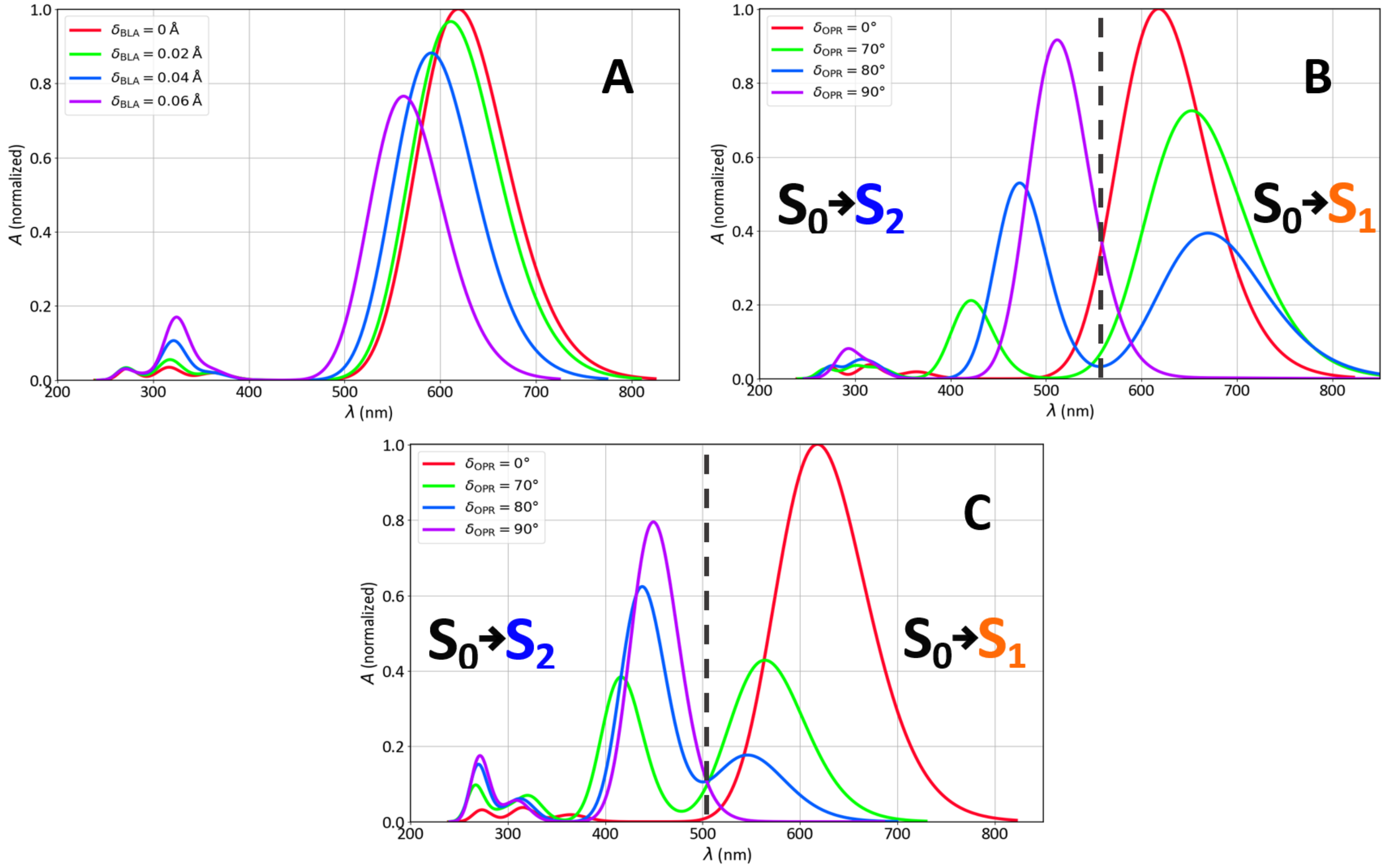

Figure S90. (A) Calculated UV/vis spectra of **10** in the planar conformation for different degrees of BLA: non-alternated (red), moderately alternated (green: BLA = 0.02 Å), and highly alternated (blue: BLA = 0.04 Å; violet: BLA = 0.06 Å). (B) Calculated UV/vis spectra of fully symmetric Cy7 (**10**) as a function of OPR of the terminal heterocyclic group. The spectra correspond to different rotational states: planar orientation (red: OPR = 0°), highly rotated (green: OPR = 70 °), near-perpendicular orientation (blue: OPR = 80°), and fully perpendicular orientation (violet: OPR = 90 °). (C) Calculated UV/vis spectra of fully symmetric Cy7 (**10**) as a function of OPR, incorporating OPR-induced BLA through constrained optimization. The color scheme follows the same convention as that in panel B. The spectra were calculated via ZINDO/S approach in the gas phase.

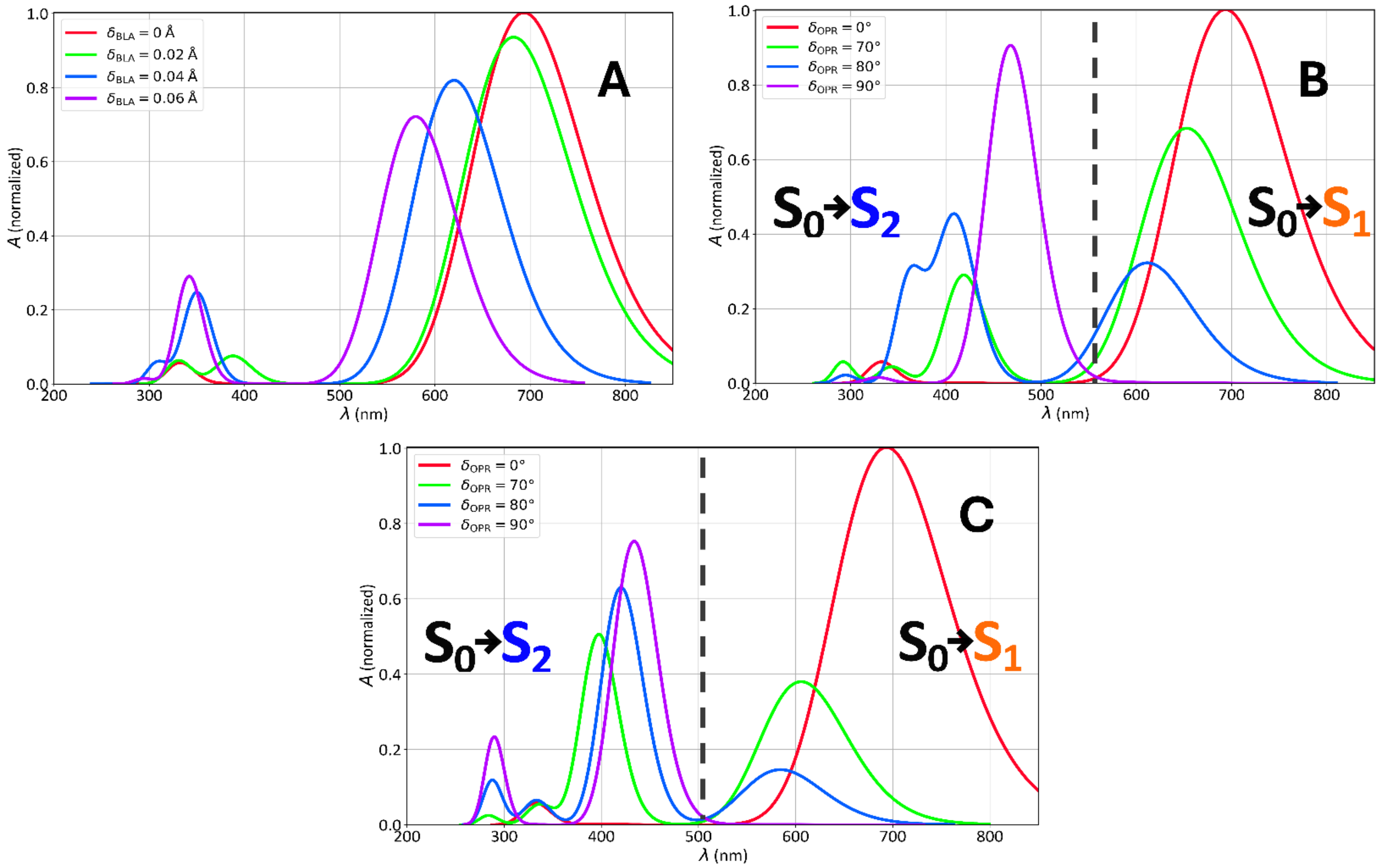

Figure S91. (A) Calculated UV/vis spectra of **10** in the planar conformation for different degrees of BLA: non-alternated (red), moderately alternated (green: BLA = 0.02 Å), and highly alternated (blue: BLA = 0.04 Å; violet: BLA = 0.06 Å). (B) Calculated UV/vis spectra of fully symmetric Cy7 (**10**) as a function of OPR of the terminal heterocyclic group. The spectra correspond to different rotational states: planar orientation (red: OPR = 0°), highly rotated orientation (green: OPR = 70 °), near-perpendicular orientation (blue: OPR = 80°), and fully perpendicular orientation (violet: OPR = 90 °). (C) Calculated UV/vis spectra of fully symmetric Cy7 (**10**) as a function of OPR, incorporating OPR-induced BLA through constrained optimization. The color scheme follows the same convention as that in panel B. The spectra were calculated via SC-NEVPT2(8/14)/cc-pVDZ approach.

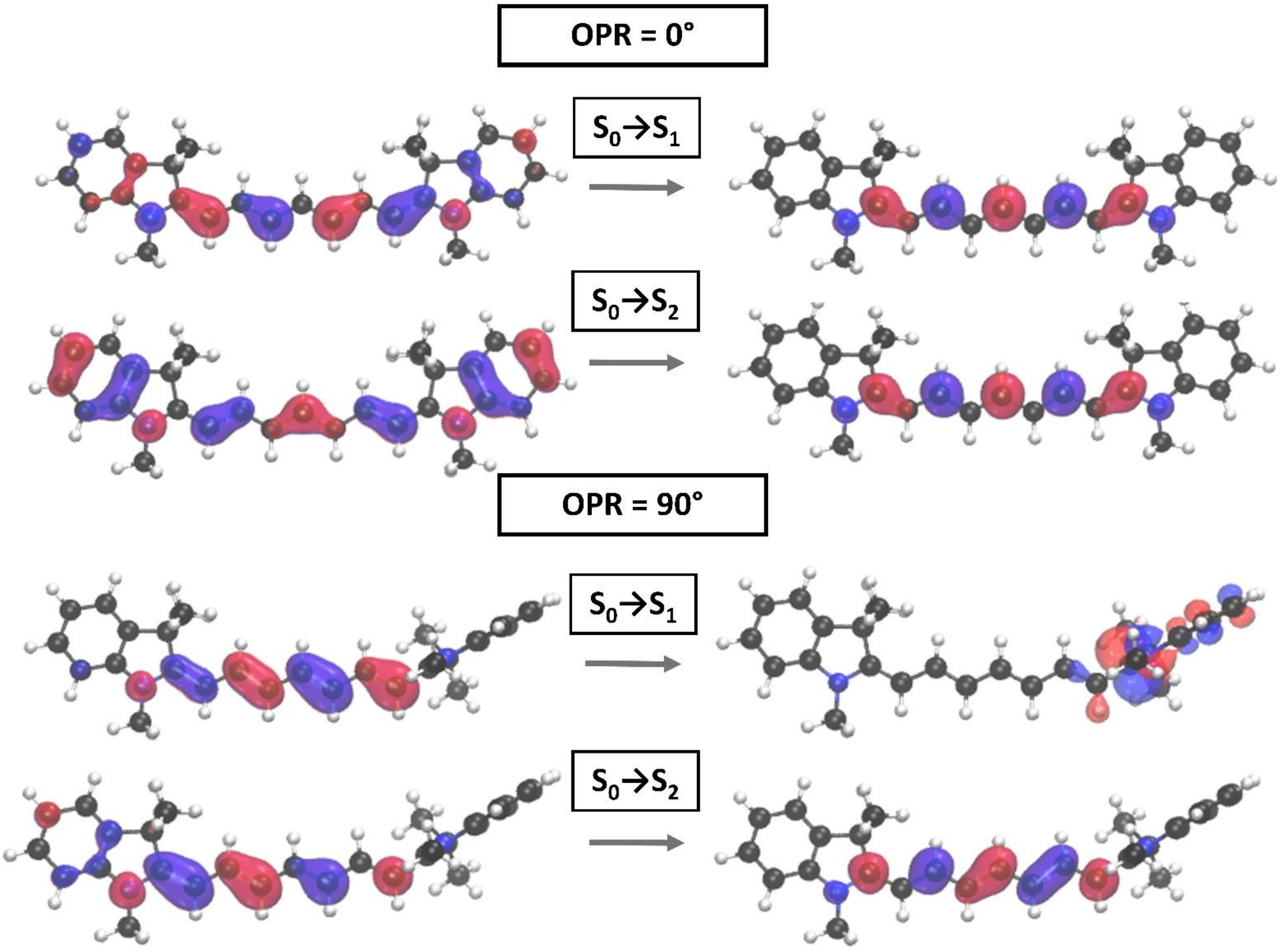

Figure S92. Natural Transition Orbitals (NTOs) characterizing the $S_0 \rightarrow S_1$ and $S_0 \rightarrow S_2$ excitations for OPR = 0° (top) and OPR = 90° (bottom). The remaining coordinates were optimized. The orbitals were visualized using a contour threshold of 0.03. The orbitals were calculated in the gas phase on ZINDO/S level of theory. By comparing the orbitals with the main text **Figure 2** (calculated in methanol via IEF-PCM approach), the charge transfer character now corresponds to the $S_0 \rightarrow S_1$ excitation instead of the $S_0 \rightarrow S_2$. This difference is reflected in the different OPR trends shown in **Figure S90**.

Table S2. Comparison of computed vertical transition energies ($E_{exc.}$) in eV and corresponding oscillator strengths (f) for the $S_0 \rightarrow S_1$ transition using different electronic structure methods.

Structure	IEF-PCM-ZINDO/S		IEF-PCM-TD-CAM-B3LYP/6-31+G**		SC-NEVPT2/cc-pVDZ[a]	
	$E_{exc.}$ (eV)	f	$E_{exc.}$ (eV)	f	$E_{exc.}$ (eV)	f
**1**	2.118	1.694	2.456	2.184	1.975	1.653
**5** ($S_0 \rightarrow S_1$)	2.033	1.519	2.426	1.922	2.151	1.433
**5** ($S_0 \rightarrow S_2$)	3.550	0.169	4.047	0.091	3.674	0.166
**6** ($S_0 \rightarrow S_1$)	2.182	1.246	2.555	1.750	2.287	1.266
**6** ($S_0 \rightarrow S_2$)	3.505	0.401	4.114	0.233	3.923	0.273
**7**	2.274	1.570	2.605	1.984	2.384	1.398
**8**	2.179	1.553	2.527	1.971	2.028	1.509
**9**	2.198	1.273	2.583	1.674	1.935	1.231
**10**	1.854	1.993	2.170	2.707	1.788	2.062
**14** ($S_0 \rightarrow S_1$)	2.183	1.507	2.406	2.180	1.966	1.388
**14** ($S_0 \rightarrow S_2$)	3.245	0.459	3.807	0.245	3.339	0.583
**15** ($S_0 \rightarrow S_1$)	2.608	0.747	2.836	1.806	2.380	0.426
**15** ($S_0 \rightarrow S_2$)	3.004	1.172	3.374	0.486	2.882	1.267
**16**	2.138	1.898	2.371	2.460	1.879	1.873
**17**	1.936	1.791	2.294	2.410	1.859	1.860

[a] The chosen active spaces were 6/10 (6 electrons in 10 orbitals) and 8/14 (8 electrons in 14 orbitals) for structures **1**–**9** and **10**–**17**, respectively.

References

1. Stackova, L.; Muchova, E.; Russo, M.; Slavicek, P.; Stacko, P.; Klan, P., Deciphering the Structure–Property Relations in Substituted Heptamethine Cyanines. *J. Org. Chem.* **2020,** *85*, 9776-9790.
2. Wilkinson, F.; Helman, W. P.; Ross, A. B., Quantum Yields for the Photosensitized Formation of the Lowest Electronically Excited Singlet State of Molecular Oxygen in Solution. *J. Phys. Chem. Ref. Data* **1993,** *22*, 113-262.
3. Le Guennic, B.; Jacquemin, D., Taking Up the Cyanine Challenge with Quantum Tools. *Acc. Chem. Res.* **2015,** *48*, 530-537.
4. Jacquemin, D.; Chibani, S.; Le Guennic, B.; Mennucci, B., Solvent Effects on Cyanine Derivatives: A PCM Investigation. *J. Phys. Chem. A* **2014,** *118*, 5343-5348.
5. Scalmani, G.; Frisch, M. J., Continuous Surface Charge Polarizable Continuum Models of Solvation. I. General Formalism. *J. Chem. Phys.* **2010,** *132*, 114110.
6. Ridley, J.; Zerner, M., An Intermediate Neglect of Differential Overlap Technique for Spectroscopy: Pyrrole and the Azines. *Theor. Chim. Acta* **1973,** *32*, 111-134.
7. Angeli, C.; Cimiraglia, R.; Malrieu, J.-P., *N*-Electron Valence State Perturbation Theory: A Fast Implementation of the Strongly Contracted Variant. *Chem. Phys. Lett.* **2001,** *350*, 297-305.
8. Send, R.; Valsson, O.; Filippi, C., Electronic Excitations of Simple Cyanine Dyes: Reconciling Density Functional and Wave Function Methods. *J. Chem. Theory Comput.* **2011,** *7*, 444-455.
9. Frisch, M. J.; Trucks, G. W.; Schlegel, H. B.; Scuseria, G. E.; Robb, M. A.; Cheeseman, J. R.; Scalmani, G.; Barone, V.; Petersson, G. A.; Nakatsuji, H.; Li, X.; Caricato, M.; Marenich, A. V.; Bloino, J.; Janesko, B. G.; Gomperts, R.; Mennucci, B.; Hratchian, H. P.; Ortiz, J. V.; Izmaylov, A. F.; Sonnenberg, J. L.; Williams; Ding, F.; Lipparini, F.; Egidi, F.; Goings, J.; Peng, B.; Petrone, A.; Henderson, T.; Ranasinghe, D.; Zakrzewski, V. G.; Gao, J.; Rega, N.; Zheng, G.; Liang, W.; Hada, M.; Ehara, M.; Toyota, K.; Fukuda, R.; Hasegawa, J.; Ishida, M.; Nakajima, T.; Honda, Y.; Kitao, O.; Nakai, H.; Vreven, T.; Throssell, K.; Montgomery Jr., J. A.; Peralta, J. E.; Ogliaro, F.; Bearpark, M. J.; Heyd, J. J.; Brothers, E. N.; Kudin, K. N.; Staroverov, V. N.; Keith, T. A.; Kobayashi, R.; Normand, J.; Raghavachari, K.; Rendell, A. P.; Burant, J. C.; Iyengar, S. S.; Tomasi, J.; Cossi, M.; Millam, J. M.; Klene, M.; Adamo, C.; Cammi, R.; Ochterski, J. W.; Martin, R. L.; Morokuma, K.; Farkas, O.; Foresman, J. B.; Fox, D. J. *Gaussian 16 Rev. D.01*, Wallingford, CT, 2016.
10. Neese, F., The ORCA Program System. *WIREs Comput. Mol. Sci.* **2012,** *2*, 73-78.
11. Plasser, F., TheoDORE: A Toolbox for a Detailed and Automated Analysis of Electronic Excited State Computations. *J. Chem. Phys.* **2020,** *152*.
12. Cerezo, J.; Santoro, F., FCclasses3: Vibrationally-Resolved Spectra Simulated at the Edge of the Harmonic Approximation. *J. Comput. Chem.* **2023,** *44*, 626-643.
